# Supplementary material for: Potential Biochemical Pesticide—Synthesis of Neofuranocoumarin and Inhibition the Proliferation of Spodoptera frugiperda Cells through Activating the Mitochondrial Pathway
Source: Toxins (Basel). 2022 Sep 29;14(10):677. doi: 10.3390/toxins14100677 (PMC9612269; doi:10.3390/toxins14100677)
Supplement: Supplementary file 1 [file toxins-14-00677-s001.zip › Figure S1.Spectrogram picture of compound.pdf]

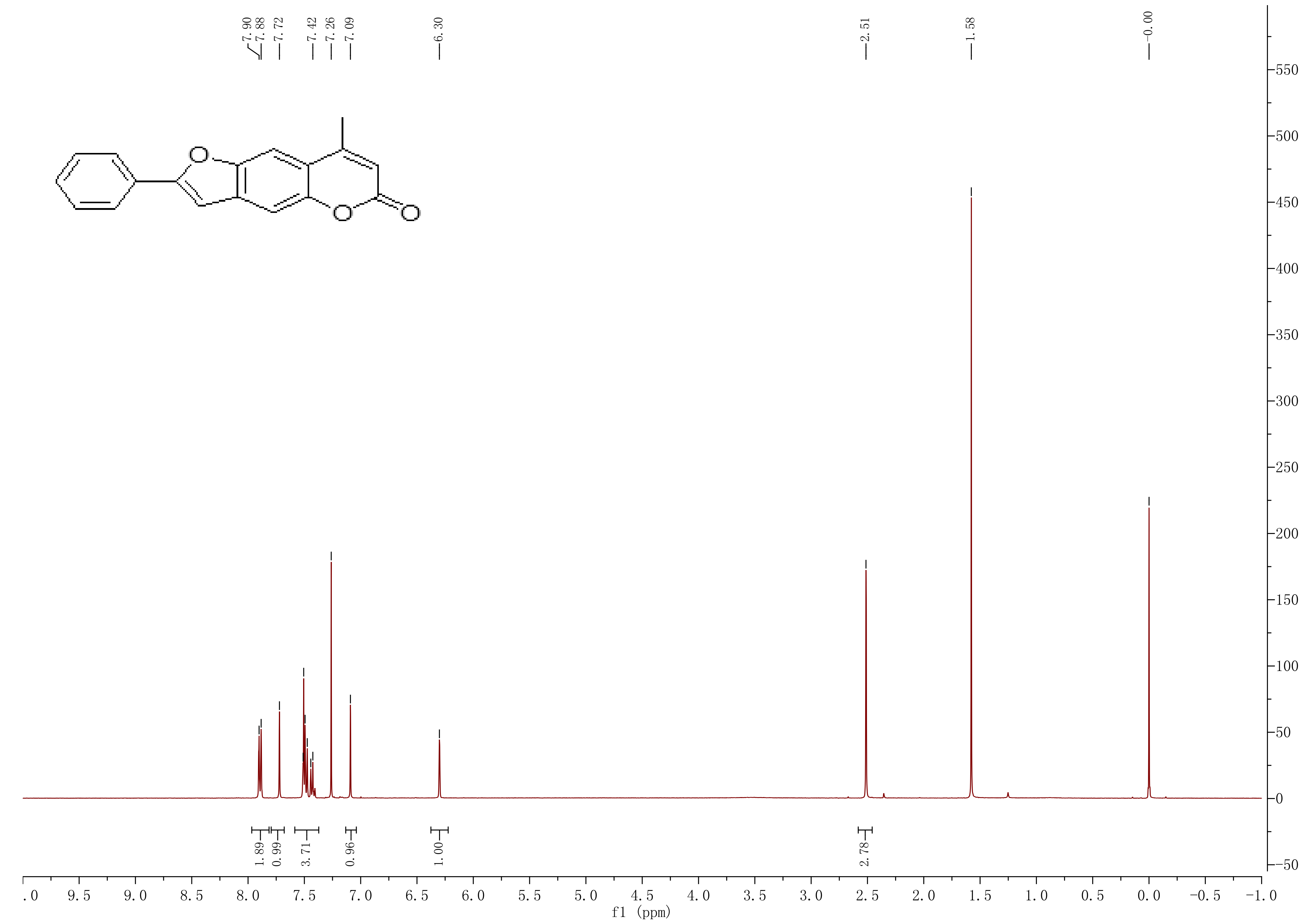

7.90
7.88
7.72
7.42
7.26
7.09
6.30
2.51
1.58
-0.00
1.89
0.99
3.71
0.96
1.00
2.78
f1 (ppm)

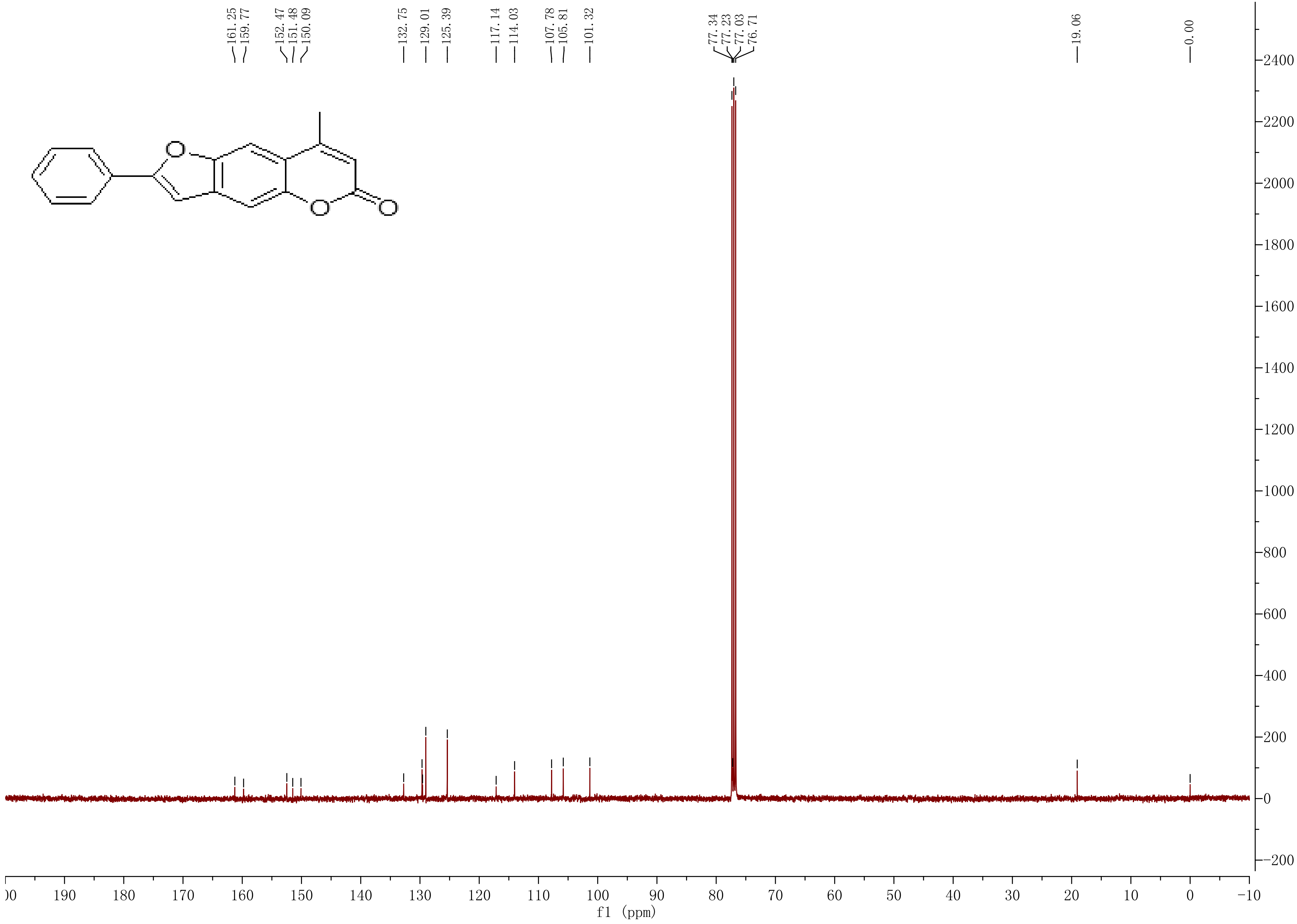
161.25
159.77
152.47
151.48
150.09
132.75
129.01
125.39
117.14
114.03
107.78
105.81
101.32
77.34
77.23
77.03
76.71
19.06
0.00
f1 (ppm)

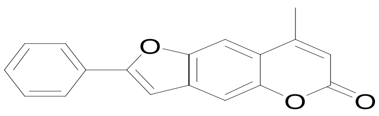

O
O
O

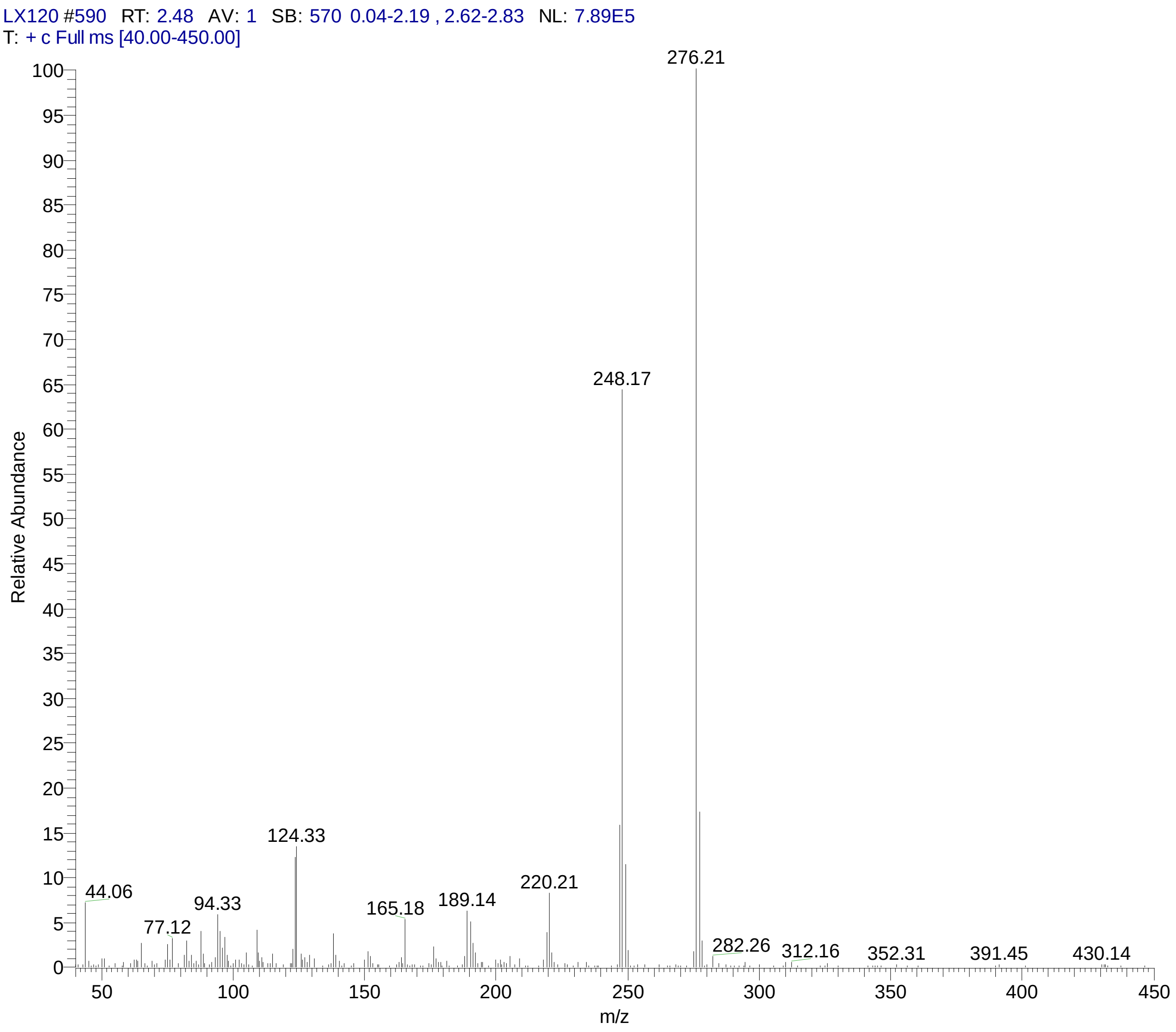

LX120 #590 RT: 2.48 AV: 1 SB: 570 0.04-2.19 , 2.62-2.83 NL: 7.89E5
T: + c Full ms [40.00-450.00]
Relative Abundance
m/z
276.21
248.17
124.33
44.06
77.12
94.33
165.18
189.14
220.21
282.26
312.16
352.31
391.45
430.14

MS of I1

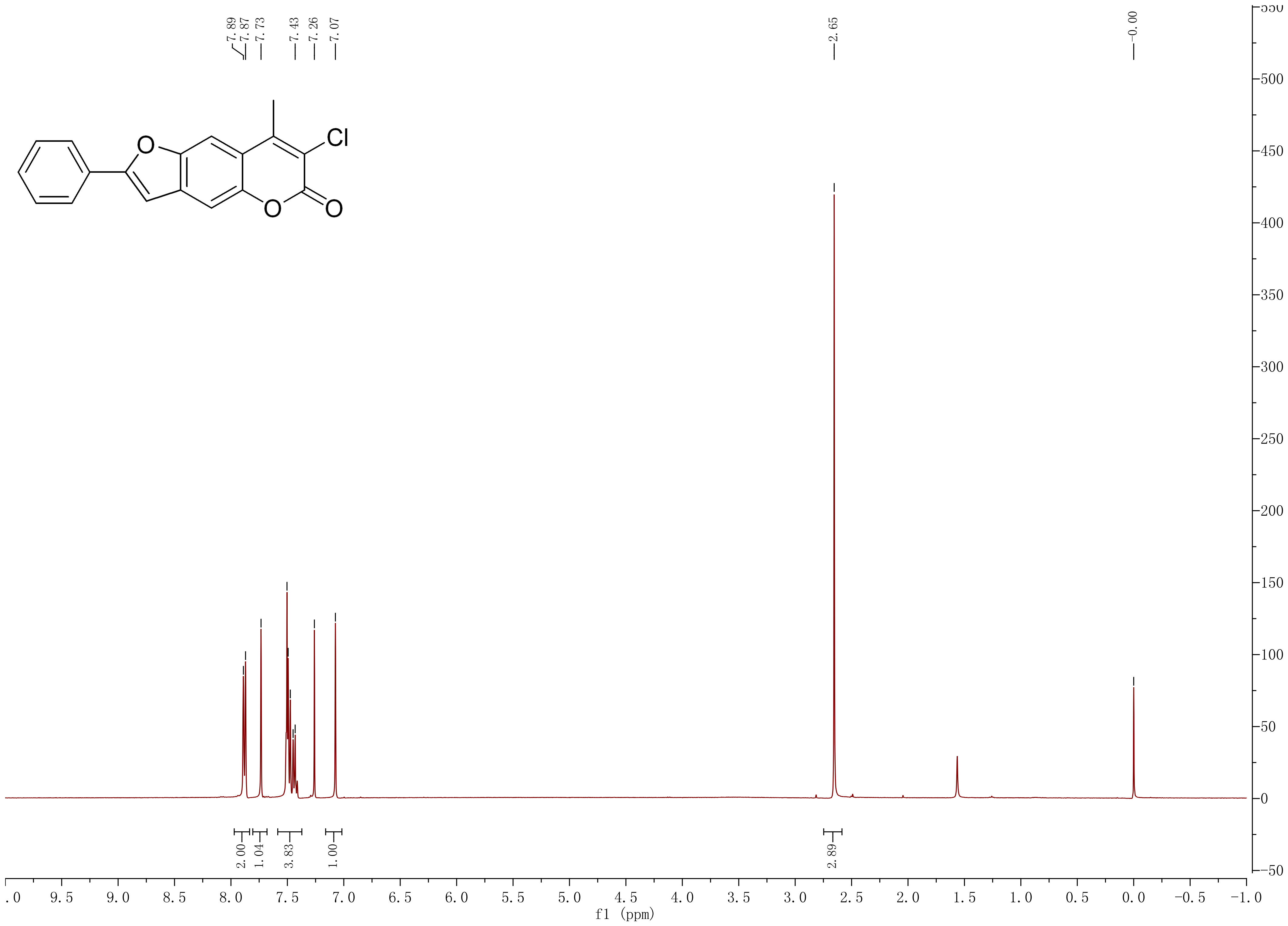

7.89
7.87
7.73
7.43
7.26
7.07
2.65
-0.00
O
Cl
O
O
2.00
1.04
3.83
1.00
2.89
.0
9.5
9.0
8.5
8.0
7.5
7.0
6.5
6.0
5.5
5.0
4.5
4.0
3.5
3.0
2.5
2.0
1.5
1.0
0.5
0.0
-0.5
-1.0
f1 (ppm)
550
500
450
400
350
300
250
200
150
100
50
0
-50

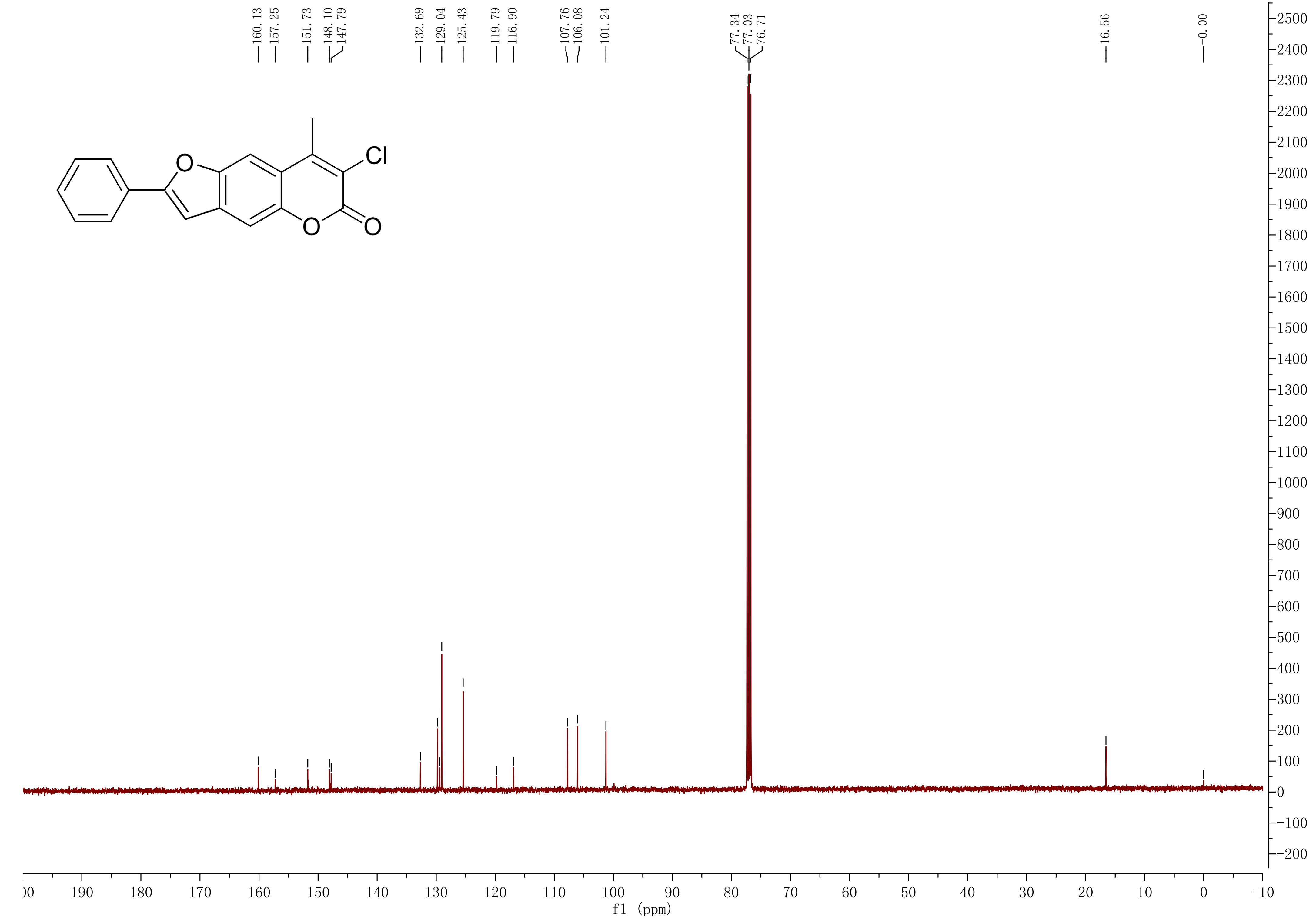

160.13
157.25
151.73
148.10
147.79
132.69
129.04
125.43
119.79
116.90
107.76
106.08
101.24
77.34
77.03
76.71
16.56
-0.00
Cl
O
O
O
f1 (ppm)

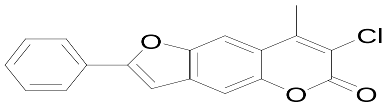

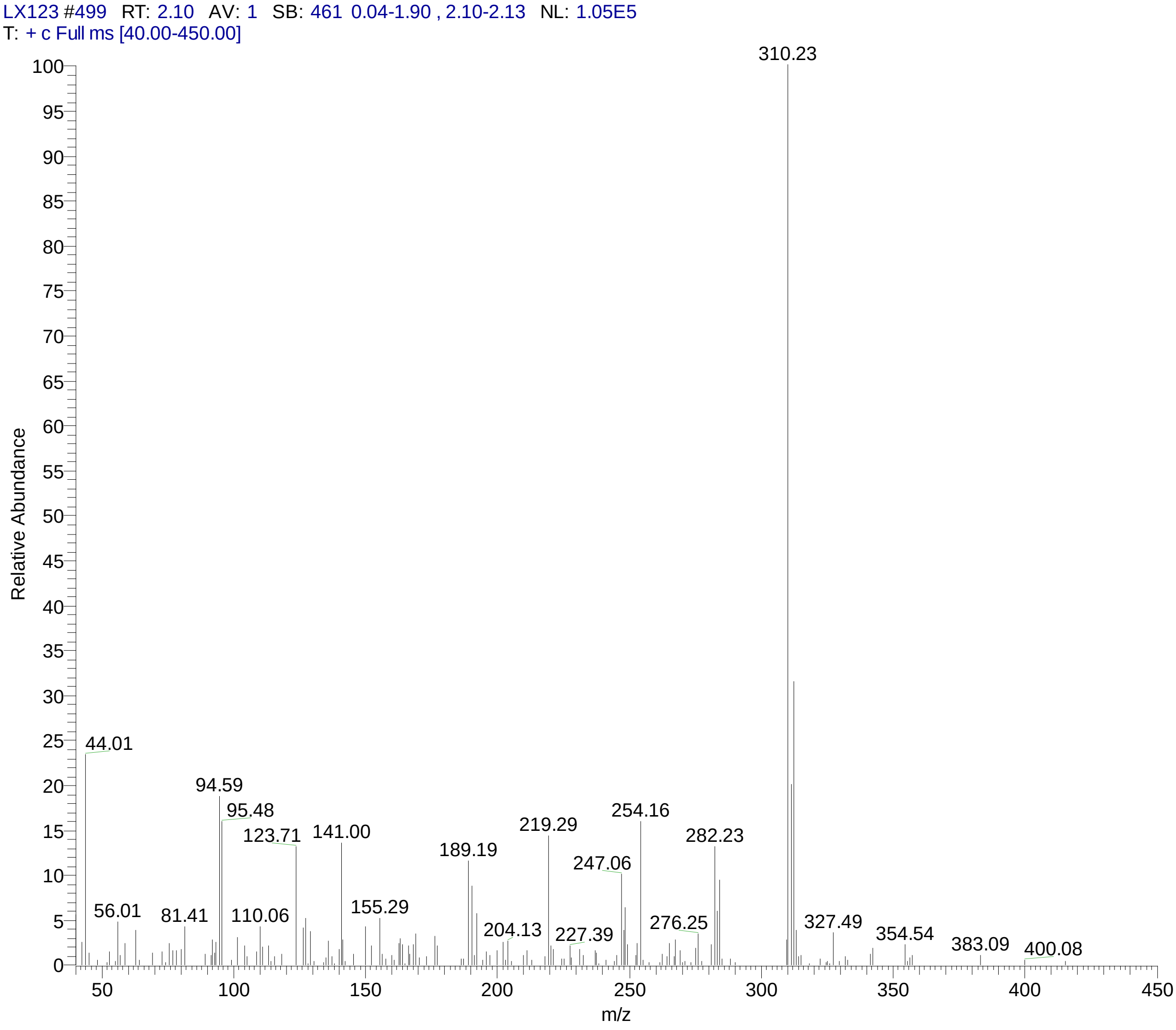

LX123 #499 RT: 2.10 AV: 1 SB: 461 0.04-1.90 , 2.10-2.13 NL: 1.05E5
T: + c Full ms [40.00-450.00]
Relative Abundance
m/z
310.23
44.01
94.59
95.48
123.71
141.00
189.19
219.29
254.16
247.06
282.23
56.01
81.41
110.06
155.29
204.13
227.39
276.25
327.49
354.54
383.09
400.08

MS of I2

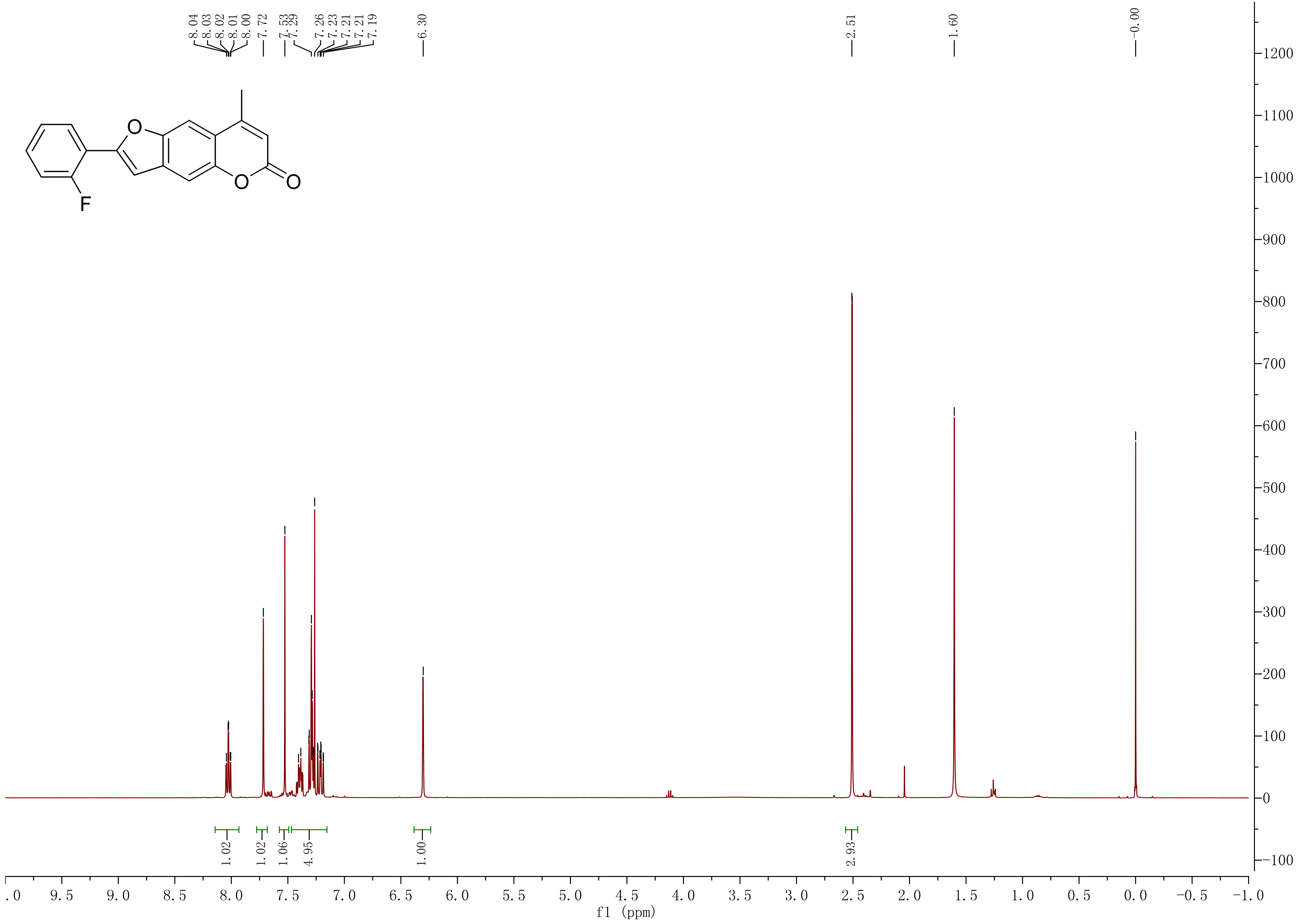
8.04
8.03
8.02
8.01
8.00
7.72
7.53
7.29
7.26
7.23
7.21
7.21
7.19
6.30
2.51
1.60
-0.00
O
O
O
F
1.02
1.02
1.06
4.95
1.00
2.93
f1 (ppm)

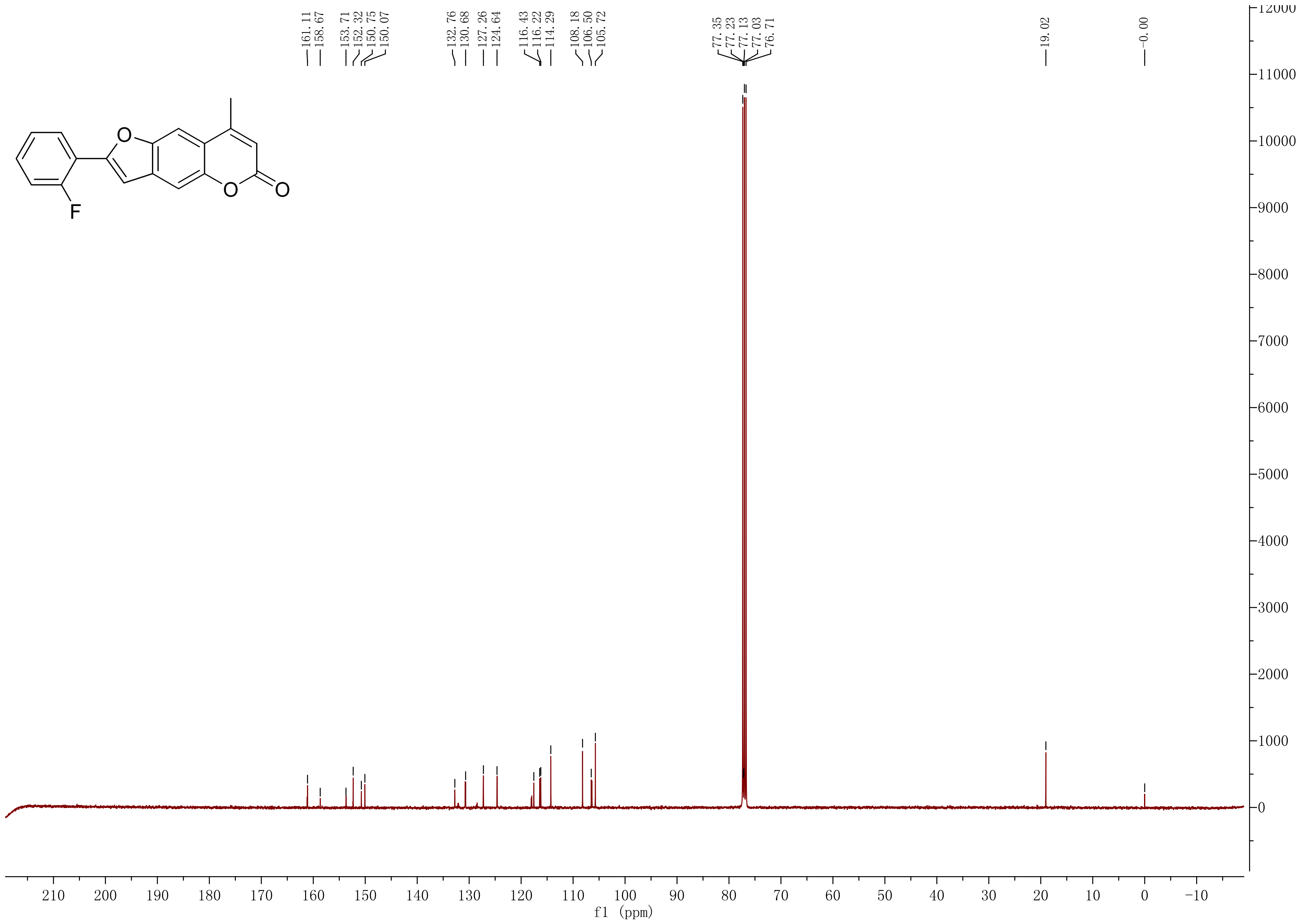

161.11
158.67
153.71
152.32
150.75
150.07
132.76
130.68
127.26
124.64
116.43
116.22
114.29
108.18
106.50
105.72
77.35
77.23
77.13
77.03
76.71
19.02
-0.00
O
O
O
F
f1 (ppm)

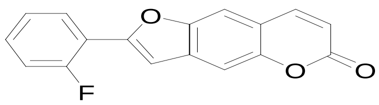

O
O
O
F

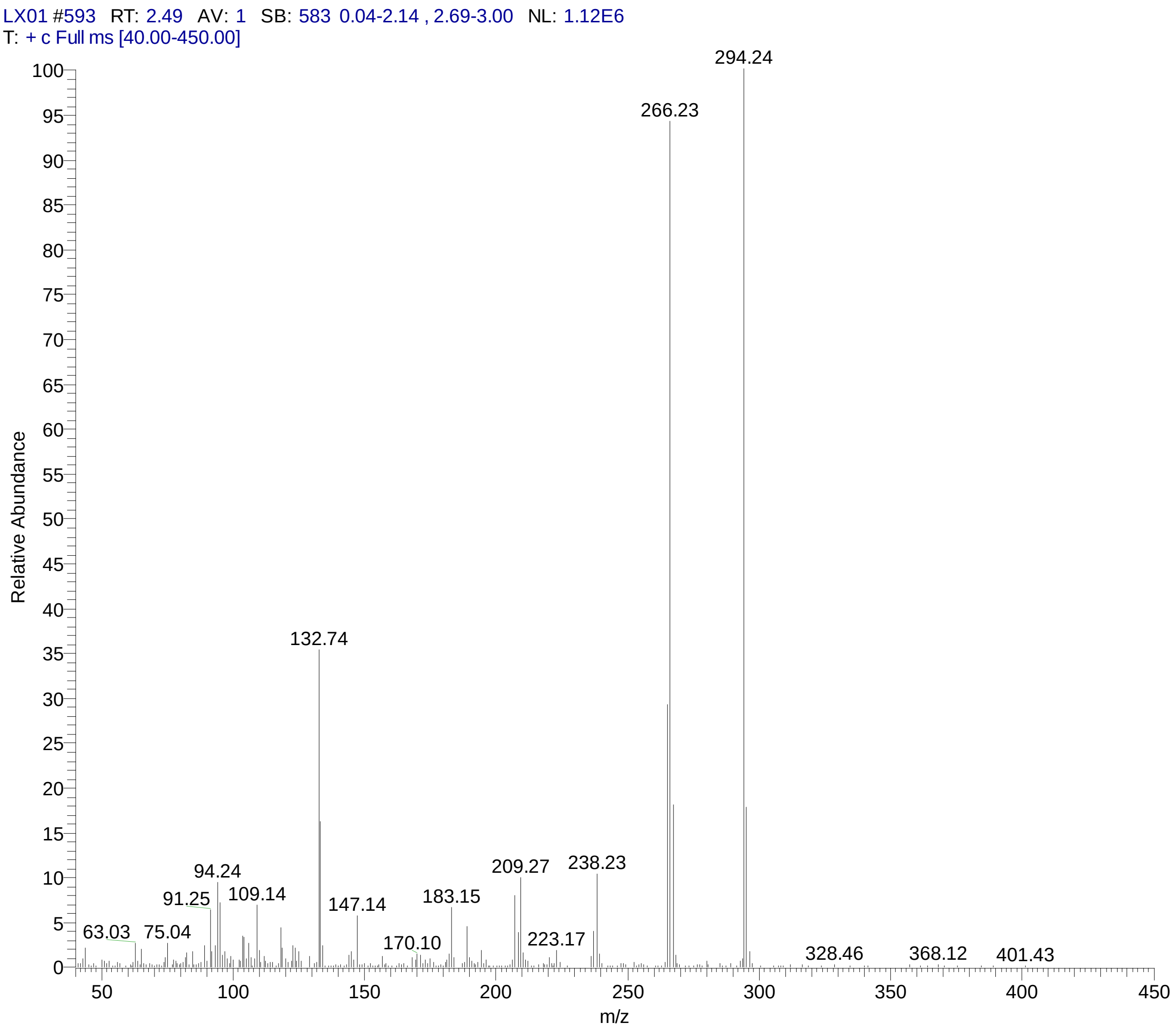

LX01 #593 RT: 2.49 AV: 1 SB: 583 0.04-2.14 , 2.69-3.00 NL: 1.12E6
T: + c Full ms [40.00-450.00]
294.24
266.23
132.74
94.24
91.25
109.14
147.14
183.15
209.27
238.23
63.03
75.04
170.10
223.17
328.46
368.12
401.43
Relative Abundance
m/z

MS of I3

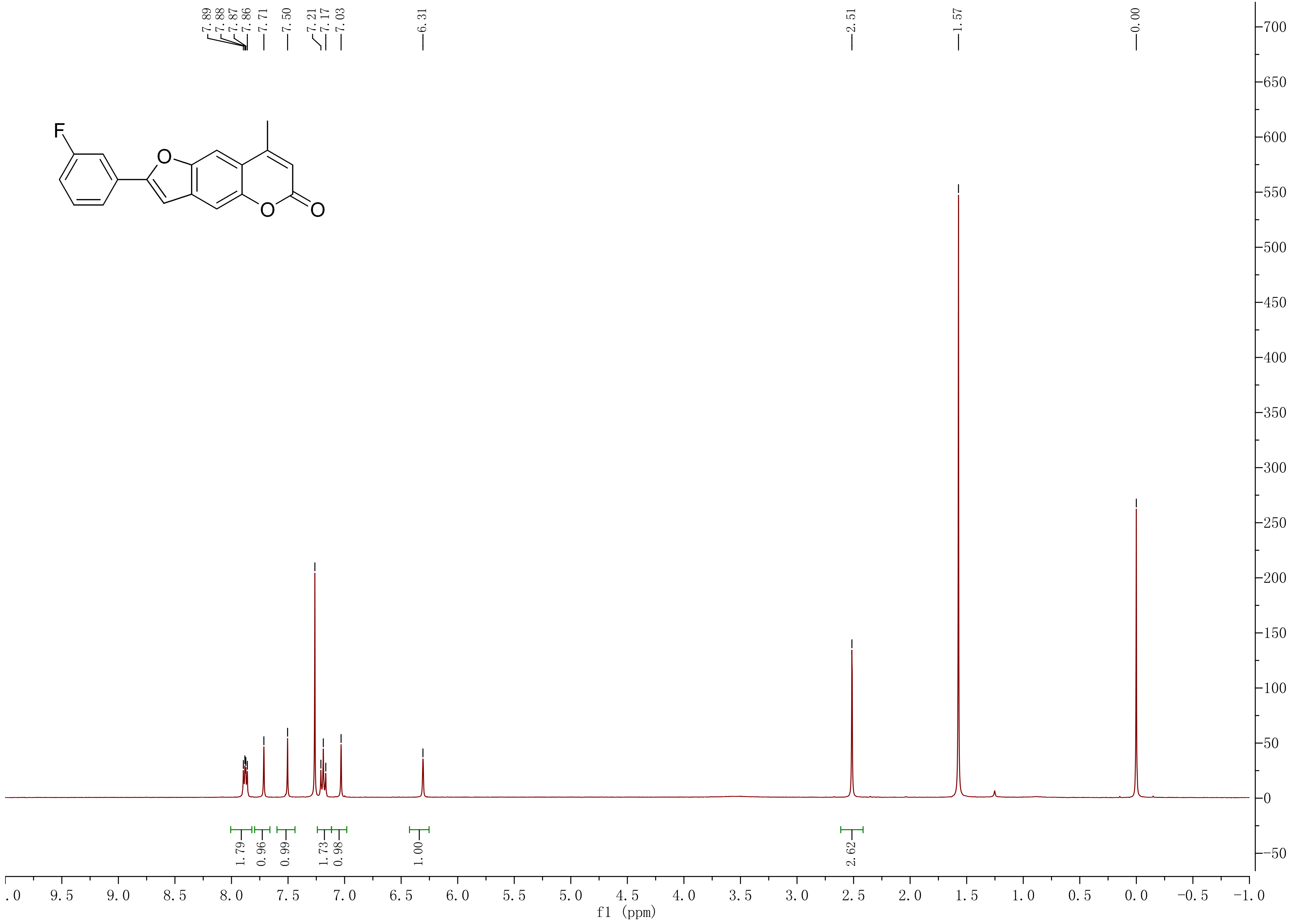
7.89
7.88
7.87
7.86
7.71
7.50
7.21
7.17
7.03
6.31
2.51
1.57
0.00
F
O
O
O
1.79
0.96
0.99
1.73
0.98
1.00
2.62
f1 (ppm)

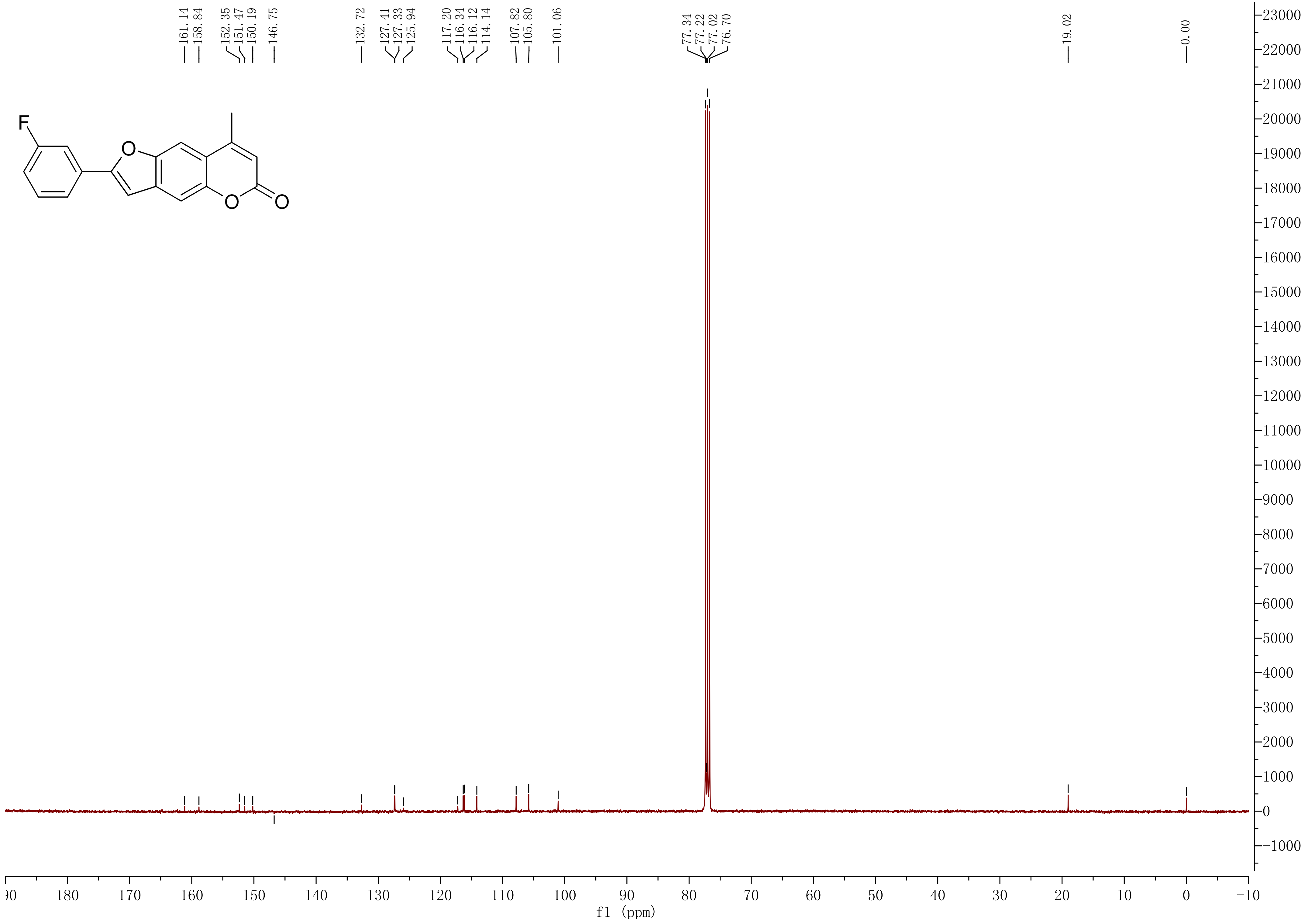
161.14
158.84
152.35
151.47
150.19
146.75
132.72
127.41
127.33
125.94
117.20
116.34
116.12
114.14
107.82
105.80
101.06
77.34
77.22
77.02
76.70
19.02
0.00
F
O
O
O
f1 (ppm)

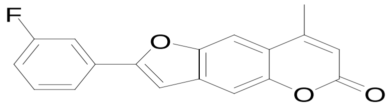
F
O
O
O

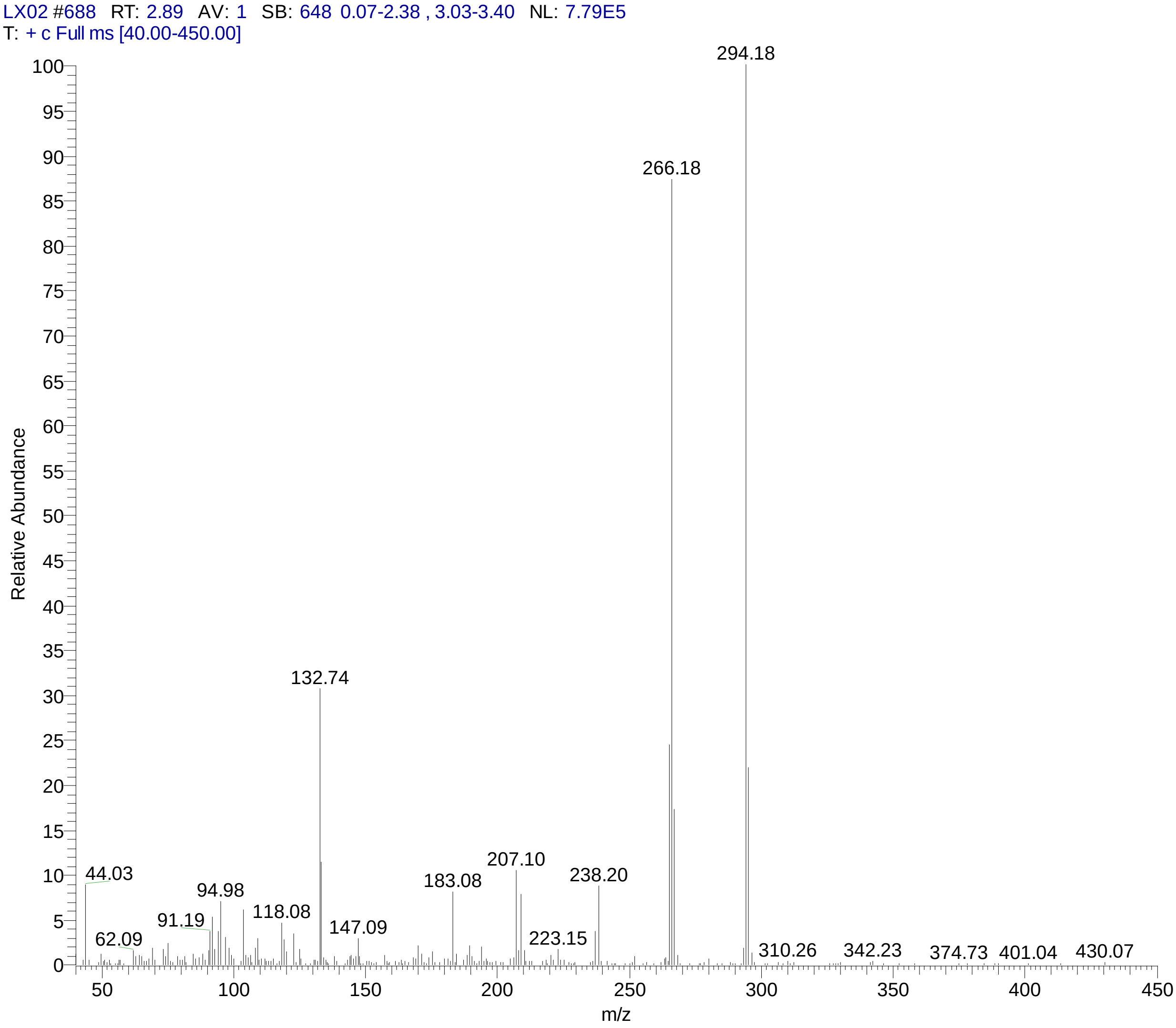
LX02 #688 RT: 2.89 AV: 1 SB: 648 0.07-2.38 , 3.03-3.40 NL: 7.79E5
T: + c Full ms [40.00-450.00]
294.18
266.18
132.74
44.03
62.09
91.19
94.98
118.08
147.09
183.08
207.10
223.15
238.20
310.26
342.23
374.73
401.04
430.07
Relative Abundance
m/z

MS of I4

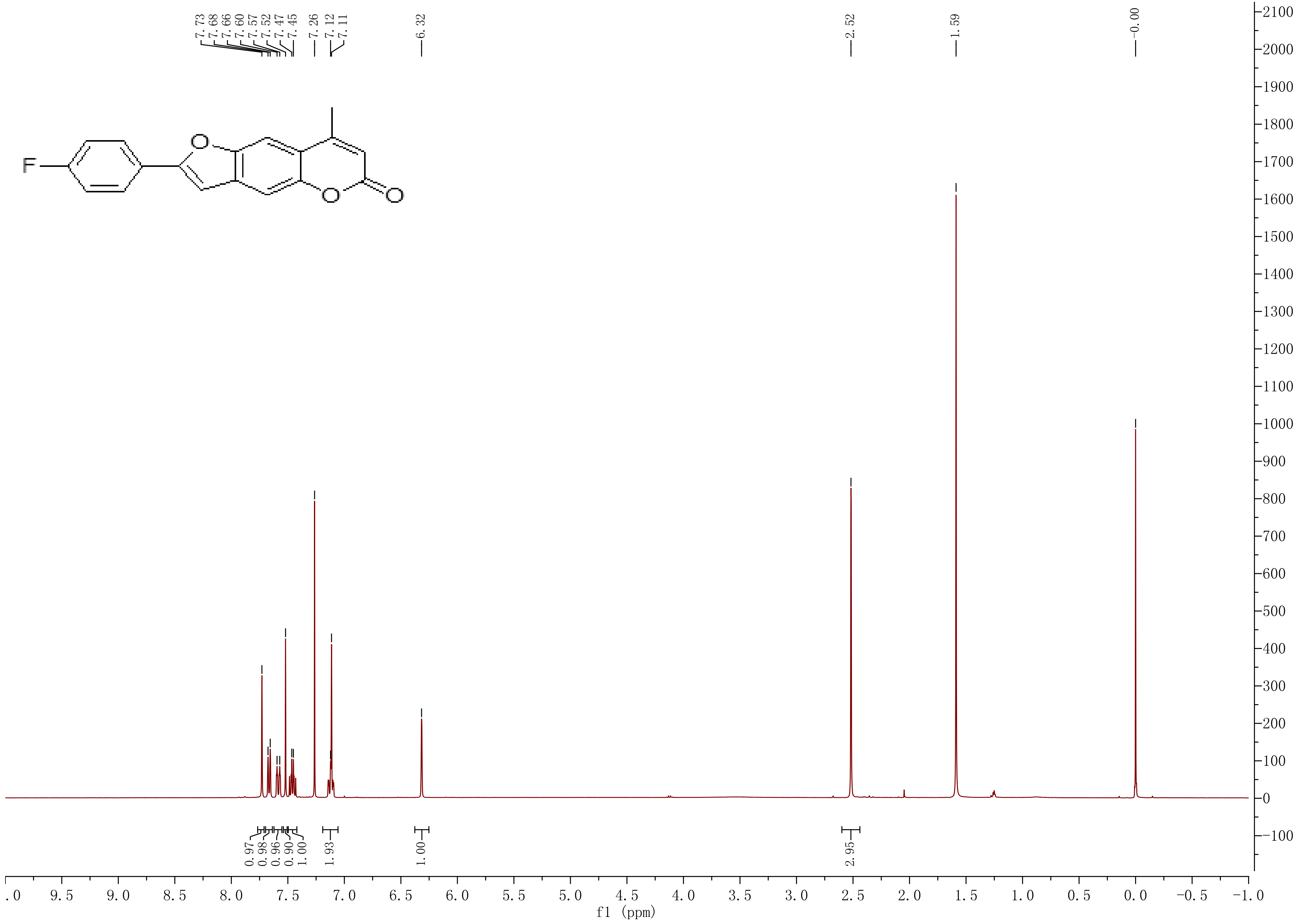

7.73 7.68 7.66 7.60 7.57 7.52 7.47 7.45 7.26 7.12 7.11
6.32
2.52
1.59
-0.00
F
O
0.97 0.98 0.96 0.90 1.00
1.93
1.00
2.95
f1 (ppm)

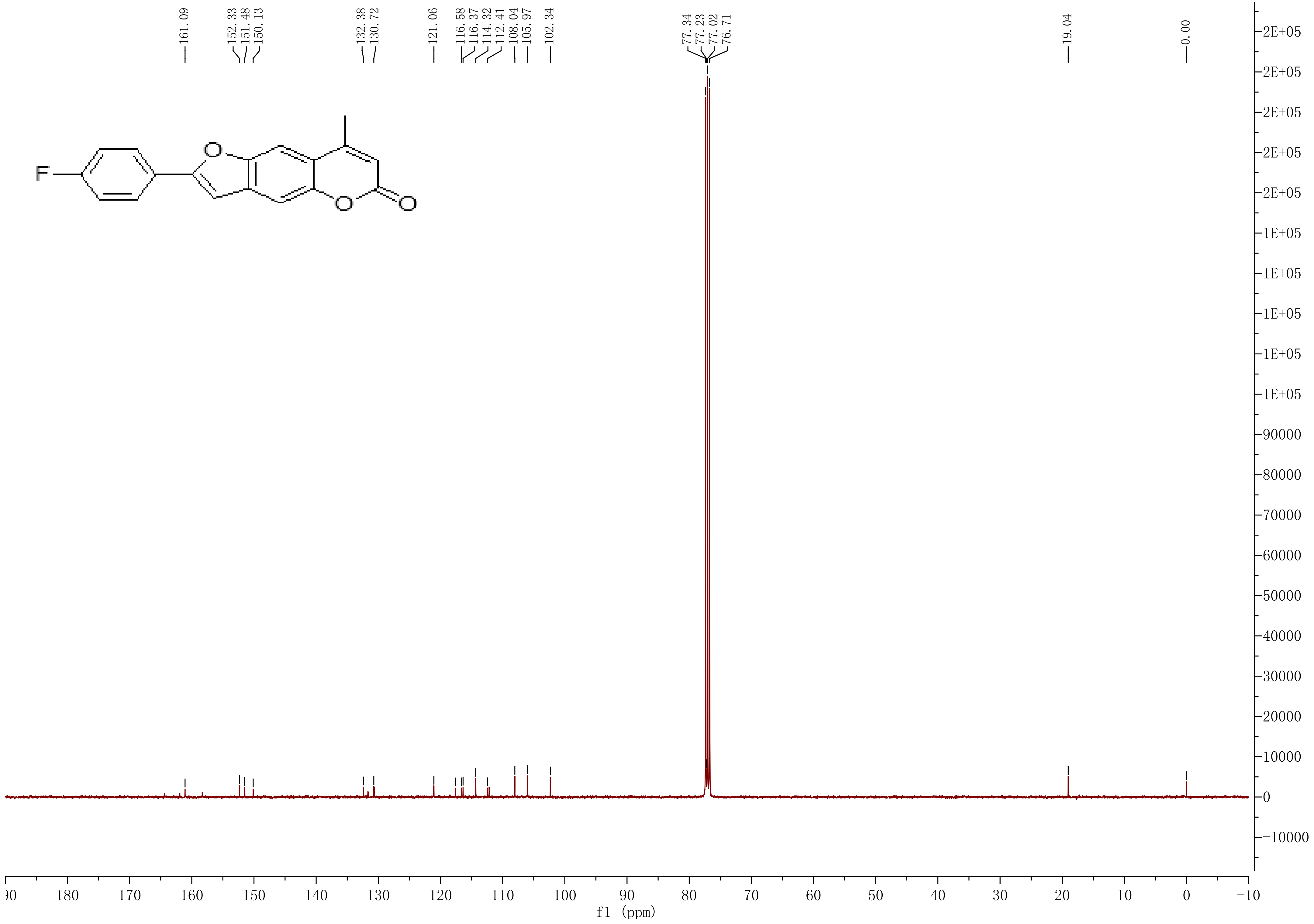

161.09
152.33
151.48
150.13
132.38
130.72
121.06
116.58
116.37
114.32
112.41
108.04
105.97
102.34
77.34
77.23
77.02
76.71
19.04
0.00
F
O
O
O
f1 (ppm)

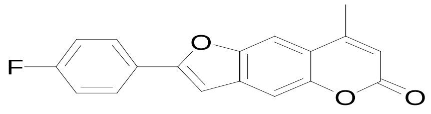
F
O
O
O

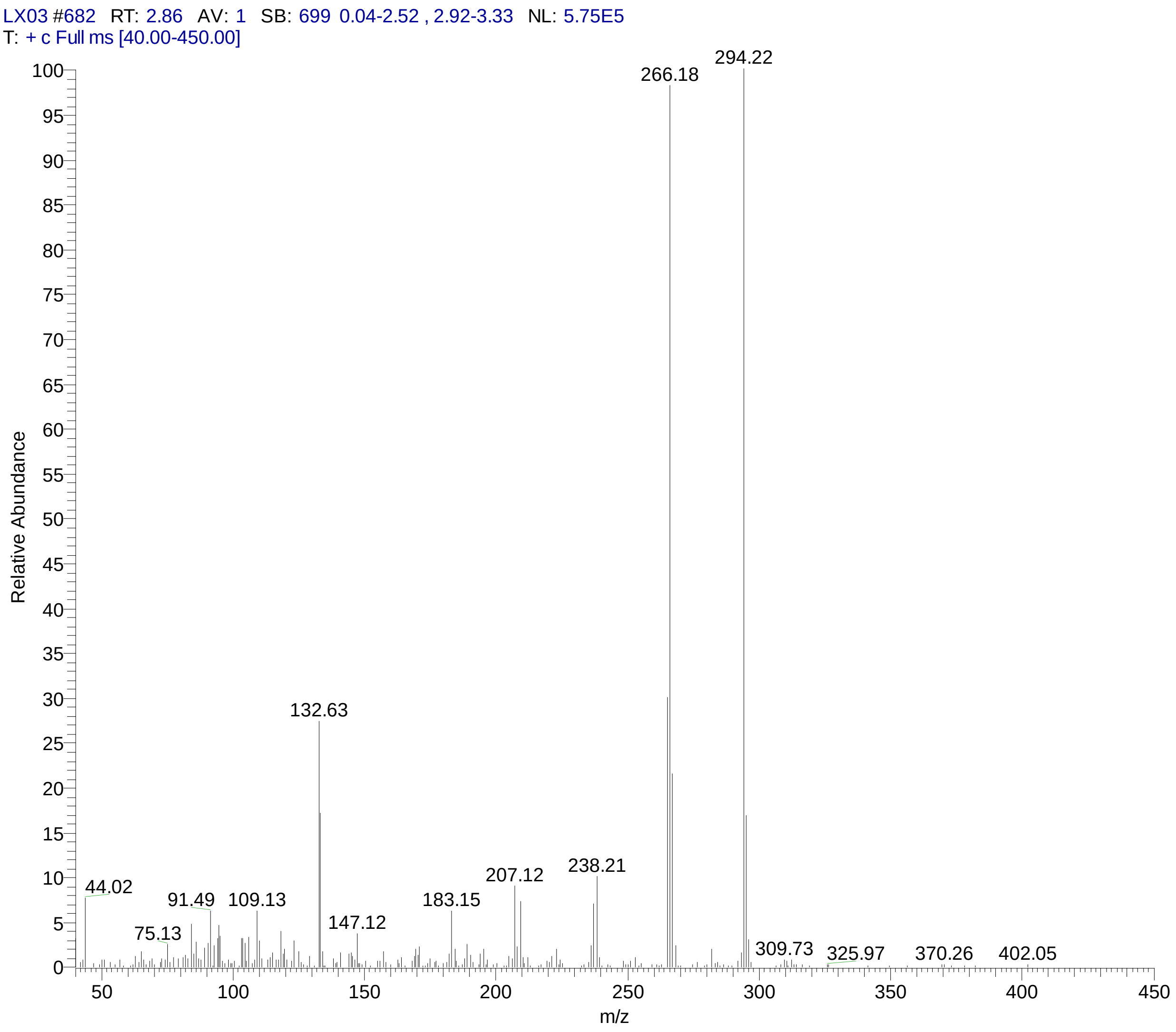
LX03 #682 RT: 2.86 AV: 1 SB: 699 0.04-2.52 , 2.92-3.33 NL: 5.75E5
T: + c Full ms [40.00-450.00]
Relative Abundance
m/z
294.22
266.18
132.63
44.02
75.13
91.49
109.13
147.12
183.15
207.12
238.21
309.73
325.97
370.26
402.05

MS of I5

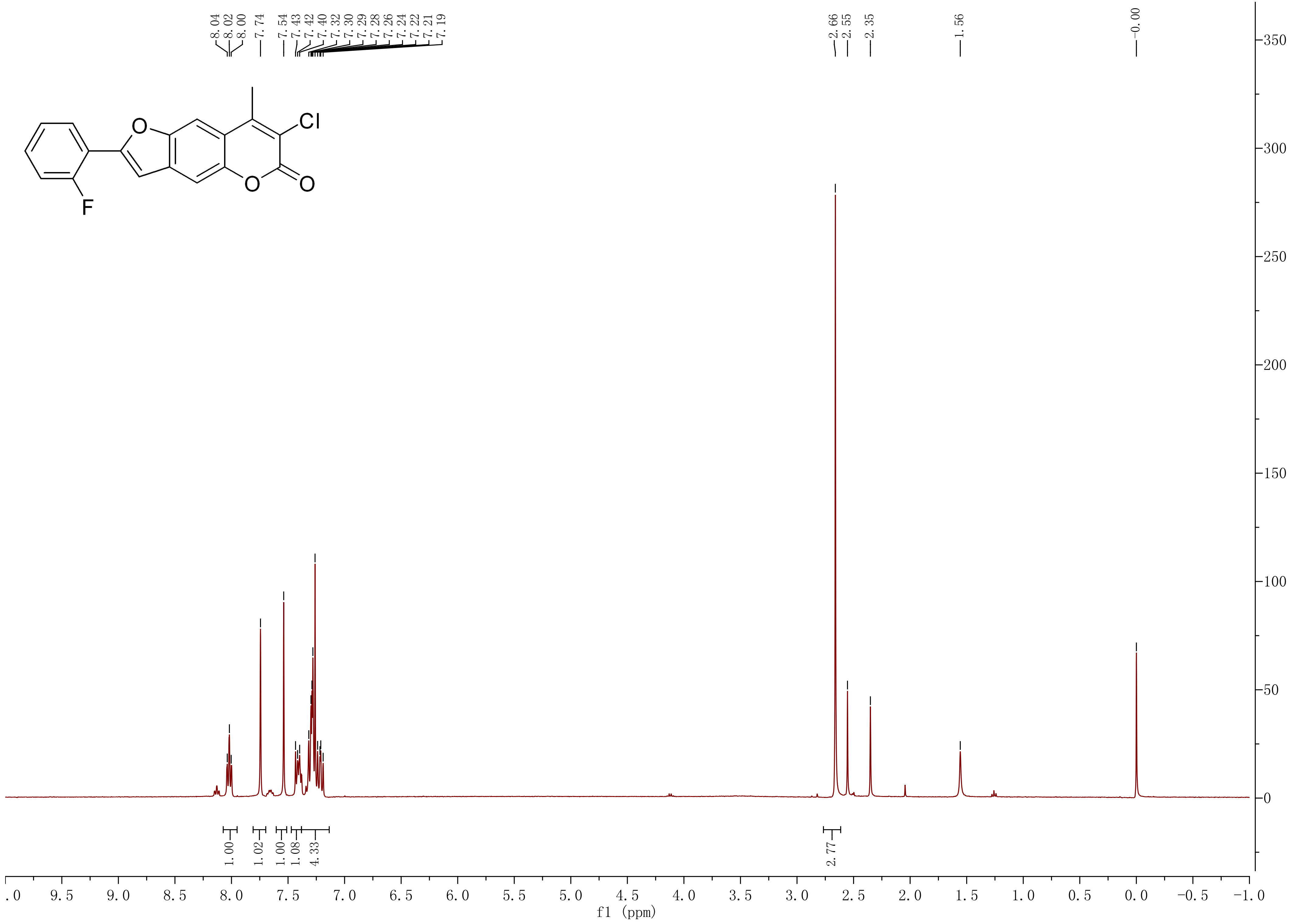
8.04
8.02
8.00
7.74
7.54
7.43
7.42
7.40
7.32
7.30
7.29
7.28
7.26
7.24
7.22
7.21
7.19
2.66
2.55
2.35
1.56
-0.00
Cl
O
O
O
F
1.00
1.02
1.00
1.08
4.33
2.77
f1 (ppm)

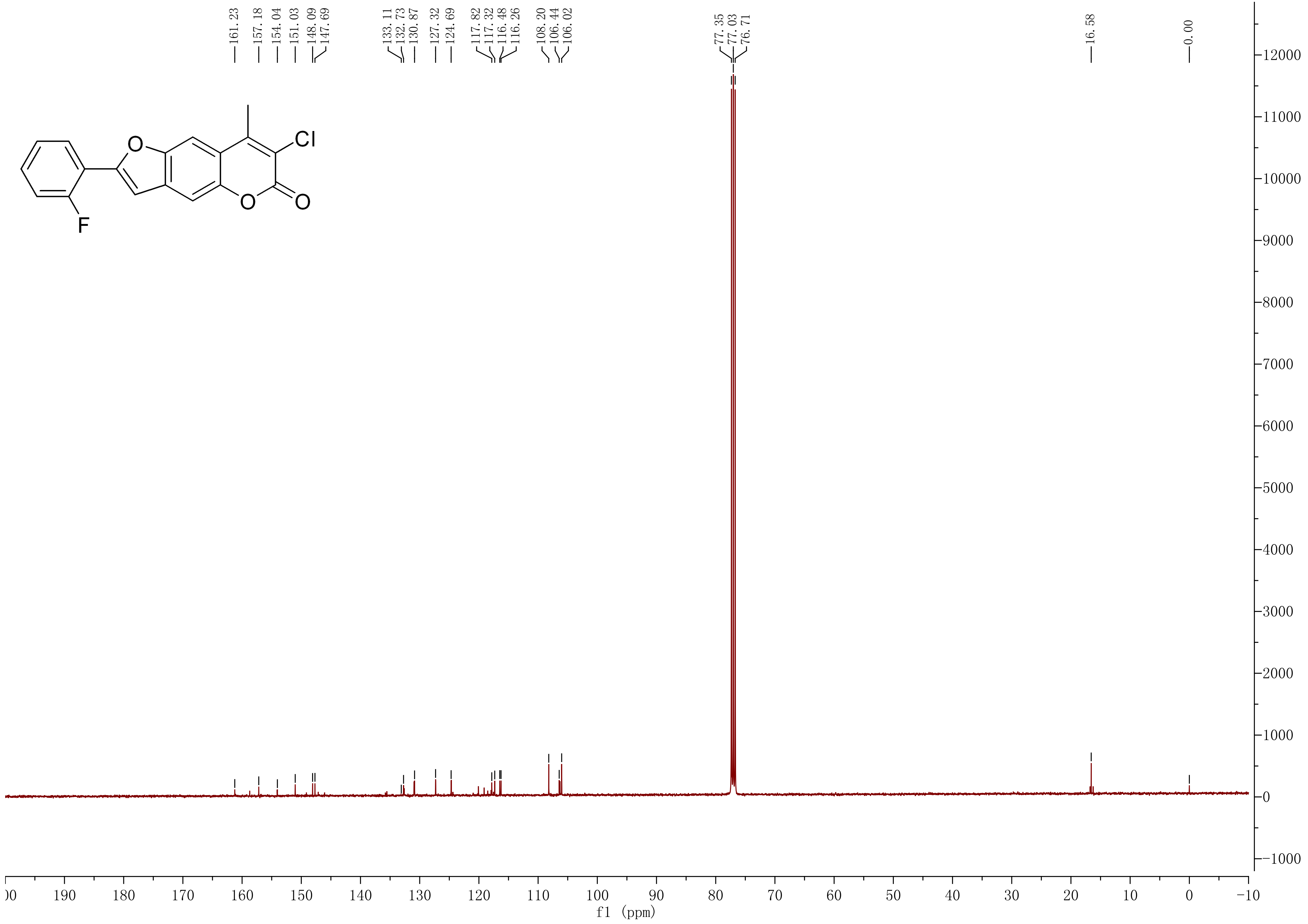
161.23
157.18
154.04
151.03
148.09
147.69
133.11
132.73
130.87
127.32
124.69
117.82
117.32
116.48
116.26
108.20
106.44
106.02
77.35
77.03
76.71
16.58
0.00
O
Cl
O
O
F
f1 (ppm)
190
180
170
160
150
140
130
120
110
100
90
80
70
60
50
40
30
20
10
0
−10
12000
11000
10000
9000
8000
7000
6000
5000
4000
3000
2000
1000
0
−1000

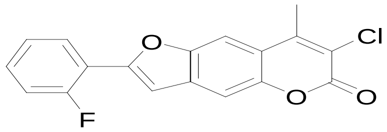

O
Cl
O
O
F

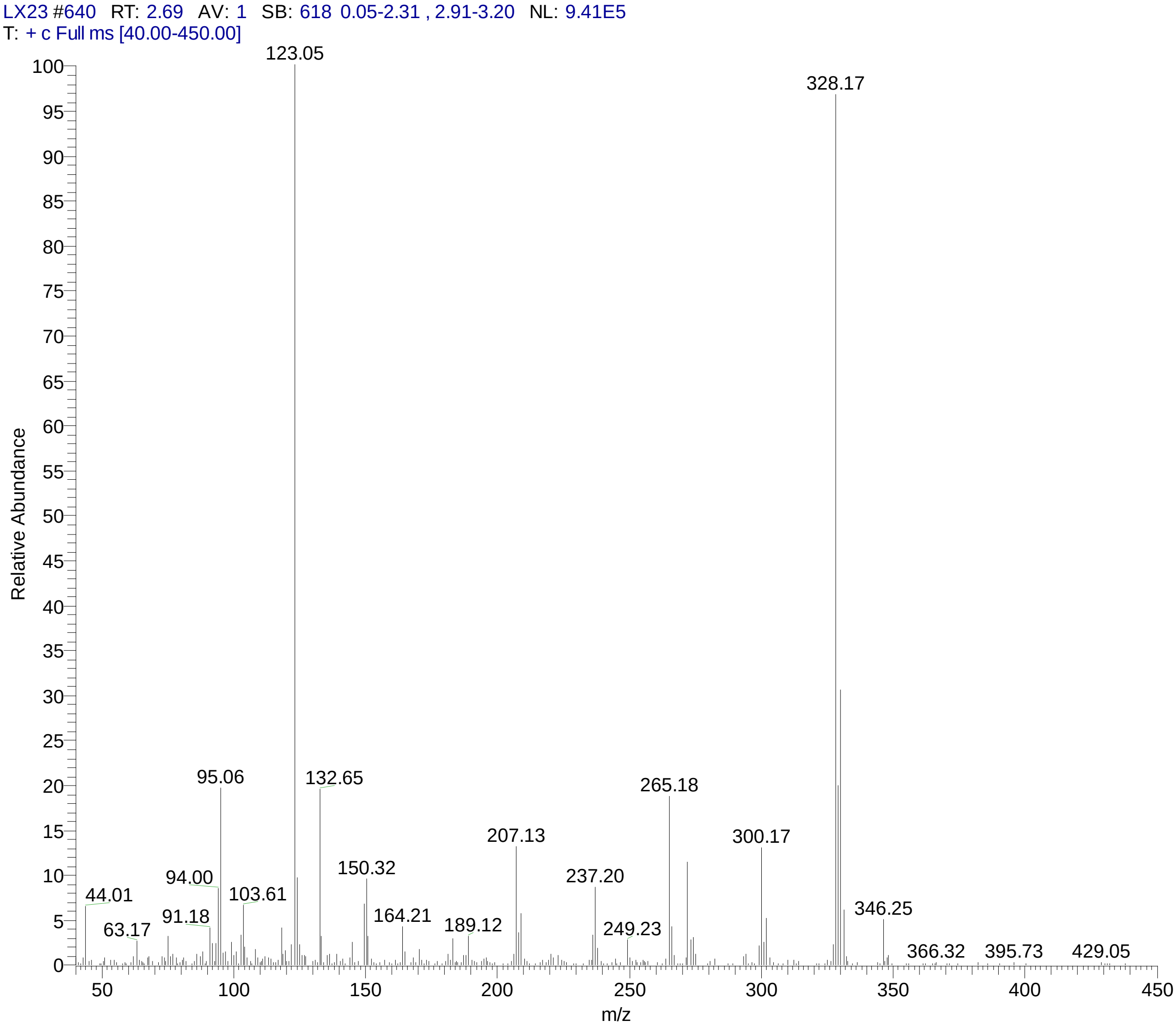

LX23 #640 RT: 2.69 AV: 1 SB: 618 0.05-2.31 , 2.91-3.20 NL: 9.41E5
T: + c Full ms [40.00-450.00]
Relative Abundance
m/z
123.05
328.17
95.06
132.65
265.18
207.13
300.17
150.32
237.20
94.00
44.01
103.61
346.25
91.18
164.21
63.17
189.12
249.23
366.32
395.73
429.05

MS of I6

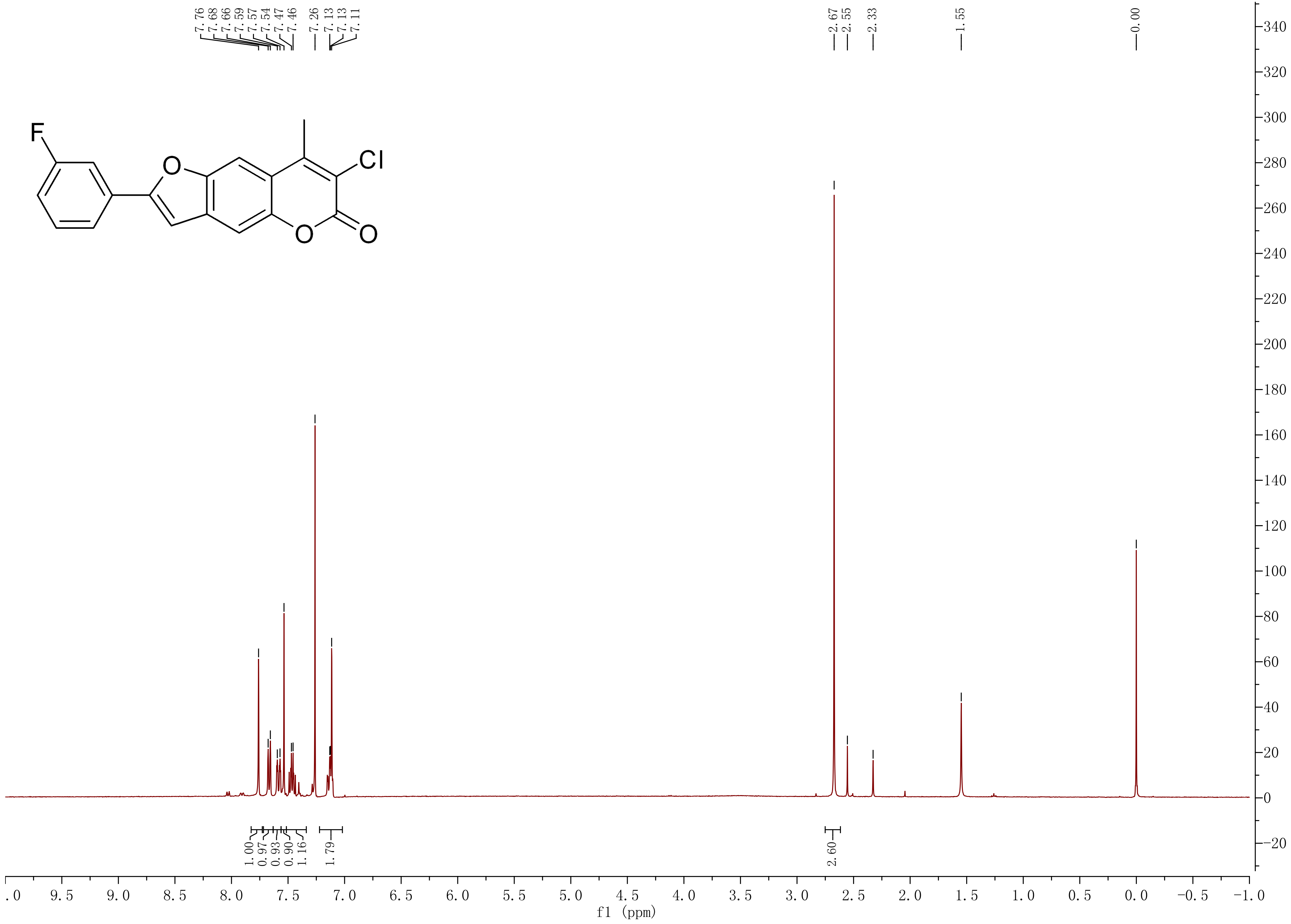
7.76
7.68
7.66
7.59
7.57
7.54
7.47
7.46
7.26
7.13
7.13
7.11
2.67
2.55
2.33
1.55
0.00
F
O
Cl
O
O
1.00
0.97
0.93
0.90
1.16
1.79
2.60
f1 (ppm)

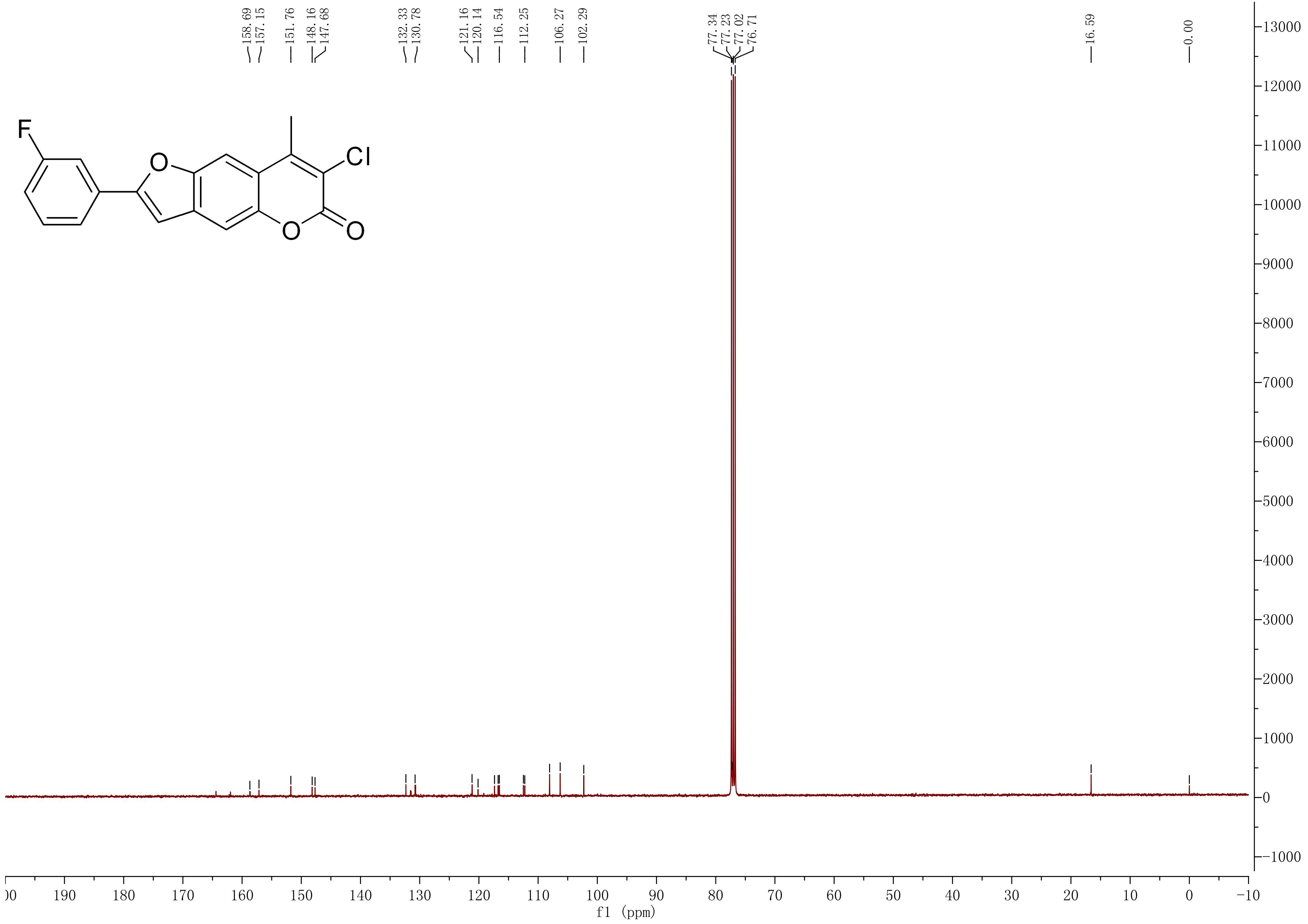
158.69
157.15
151.76
148.16
147.68
132.33
130.78
121.16
120.14
116.54
112.25
106.27
102.29
77.34
77.23
77.02
76.71
16.59
0.00
F
O
O
O
Cl
f1 (ppm)

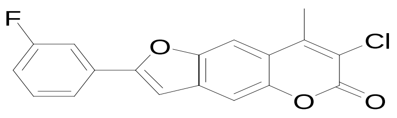
F
O
Cl
O
O

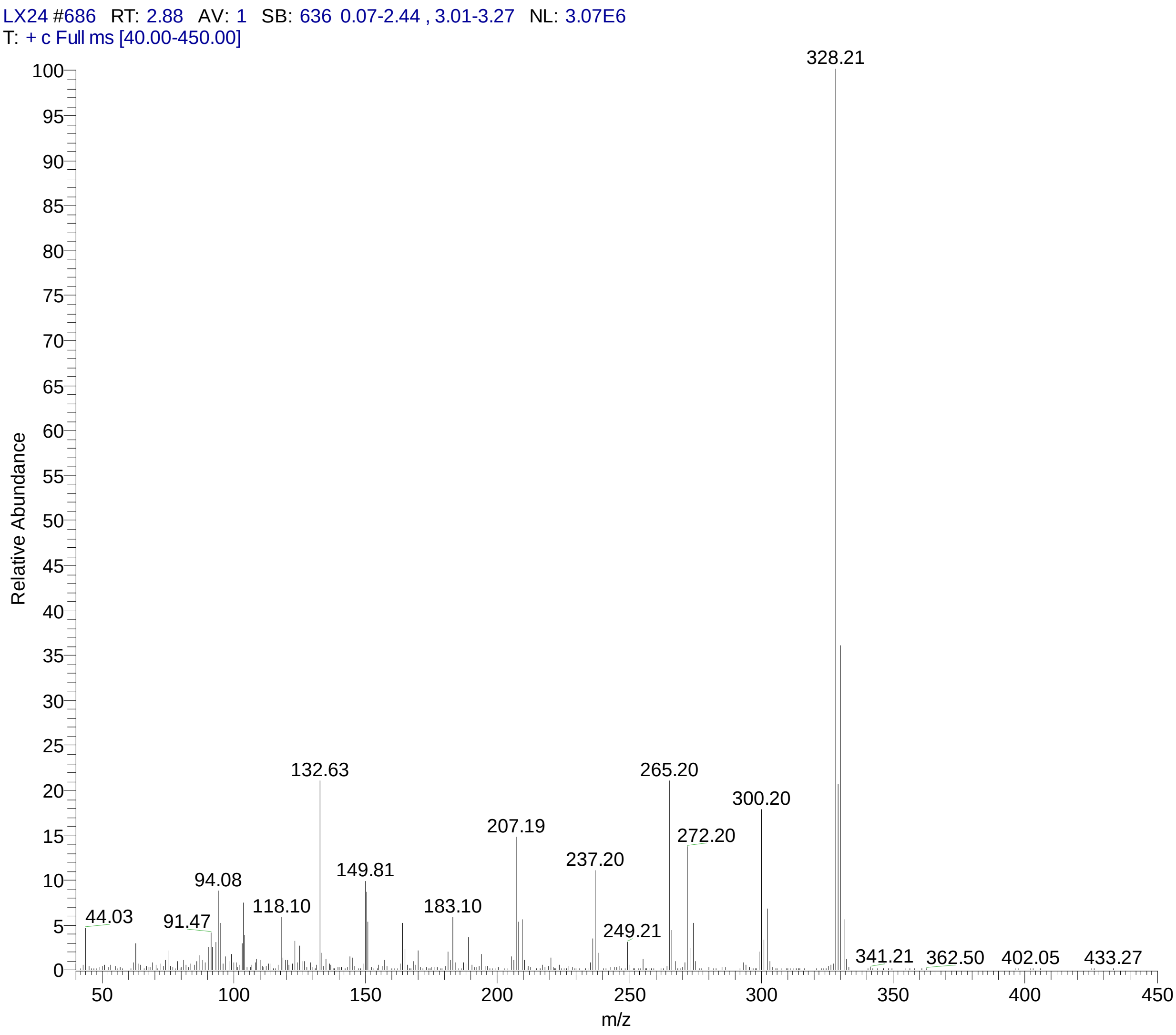
LX24 #686 RT: 2.88 AV: 1 SB: 636 0.07-2.44 , 3.01-3.27 NL: 3.07E6
T: + c Full ms [40.00-450.00]
Relative Abundance
m/z
328.21
132.63
265.20
300.20
207.19
272.20
237.20
149.81
94.08
118.10
183.10
249.21
44.03
91.47
341.21
362.50
402.05
433.27

MS of I7

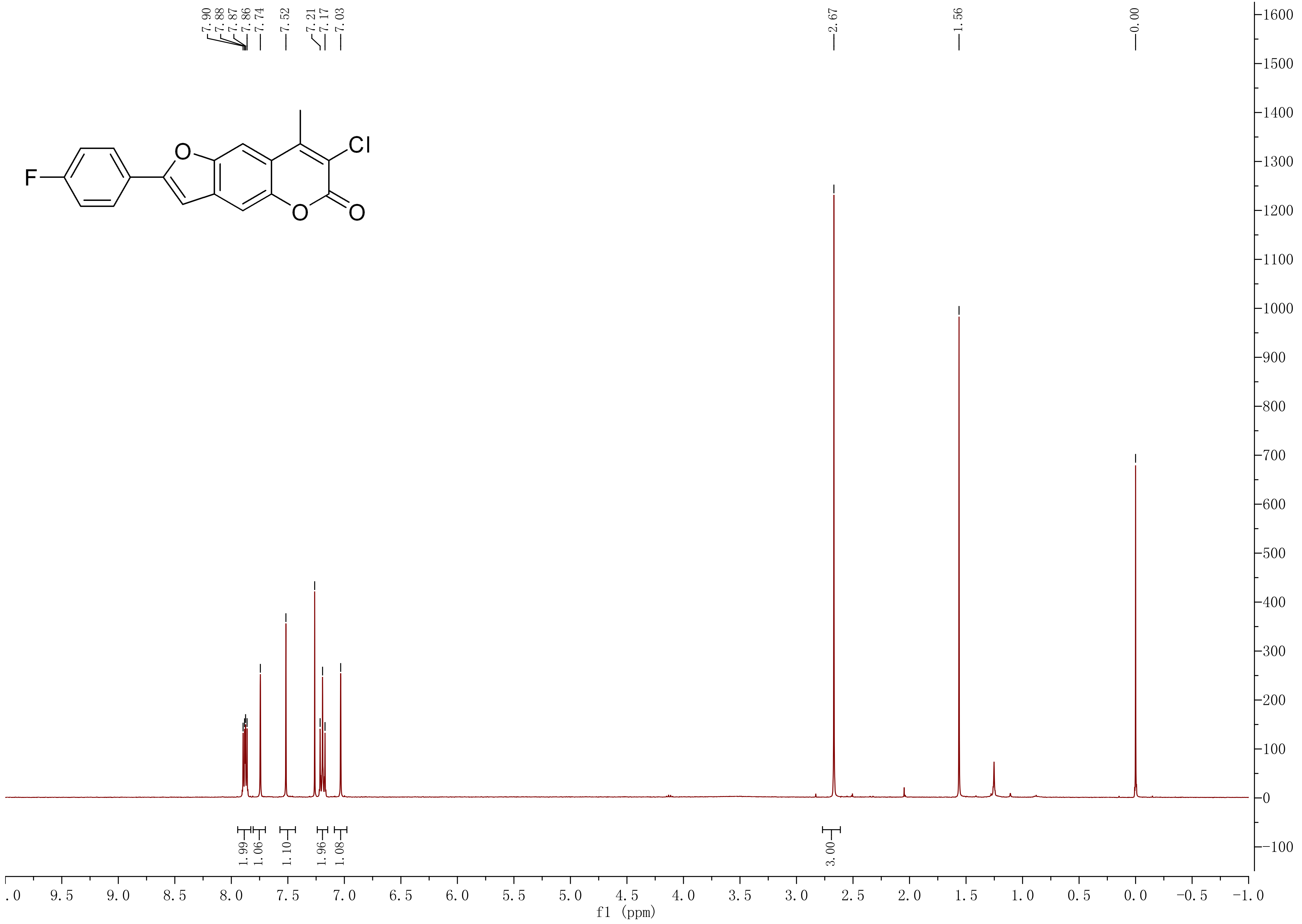

7.90
7.88
7.87
7.86
7.74
7.52
7.21
7.17
7.03
2.67
1.56
0.00
F
O
Cl
O
O
1.99
1.06
1.10
1.96
1.08
3.00
1600
1500
1400
1300
1200
1100
1000
900
800
700
600
500
400
300
200
100
0
−100
.0
9.5
9.0
8.5
8.0
7.5
7.0
6.5
6.0
5.5
5.0
4.5
4.0
3.5
3.0
2.5
2.0
1.5
1.0
0.5
0.0
−0.5
−1.0
f1 (ppm)

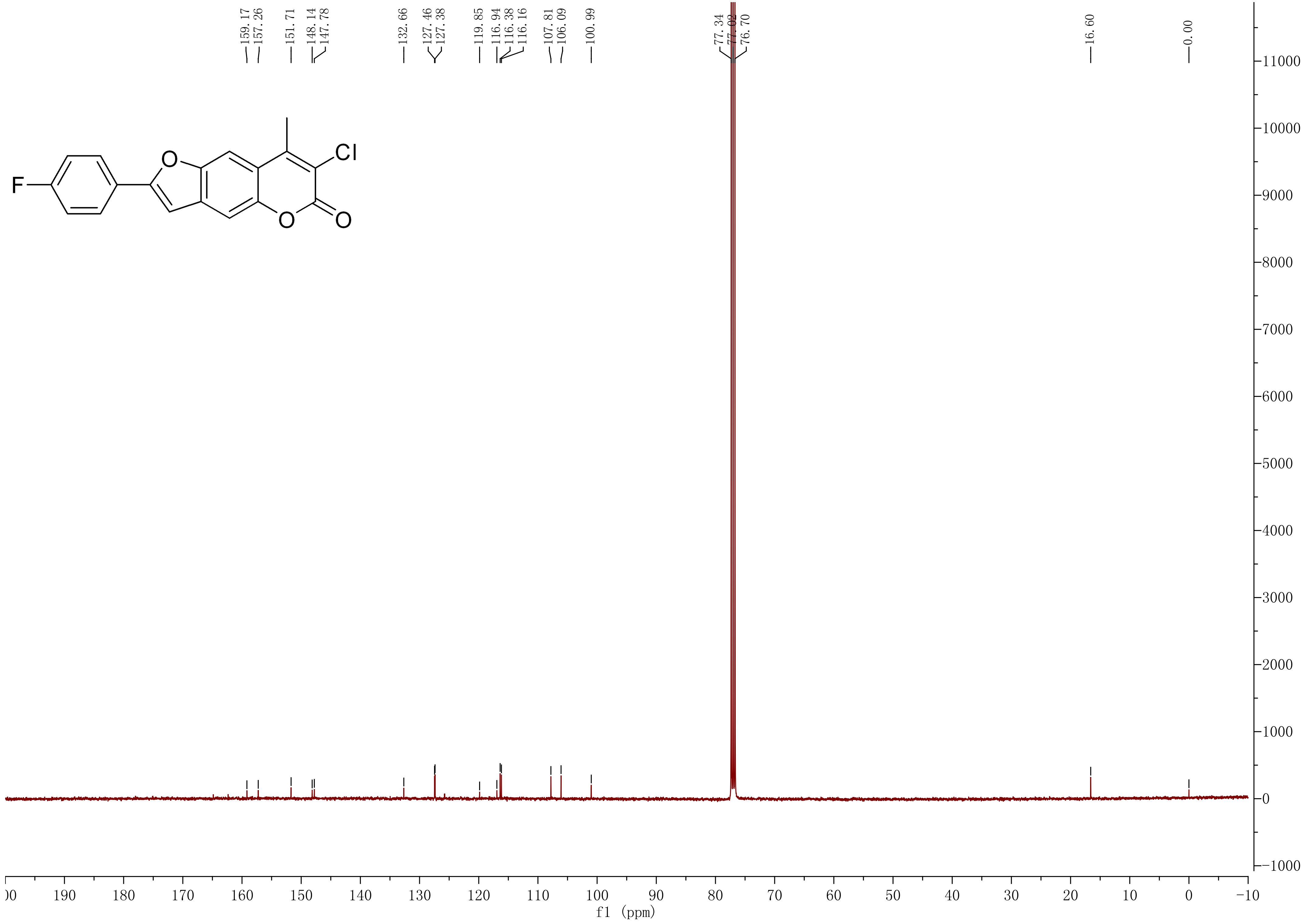

159.17
157.26
151.71
148.14
147.78
132.66
127.46
127.38
119.85
116.94
116.38
116.16
107.81
106.09
100.99
77.34
77.02
76.70
16.60
0.00
F
O
Cl
O
O
f1 (ppm)

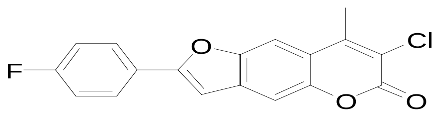
O
F
Cl
O
O

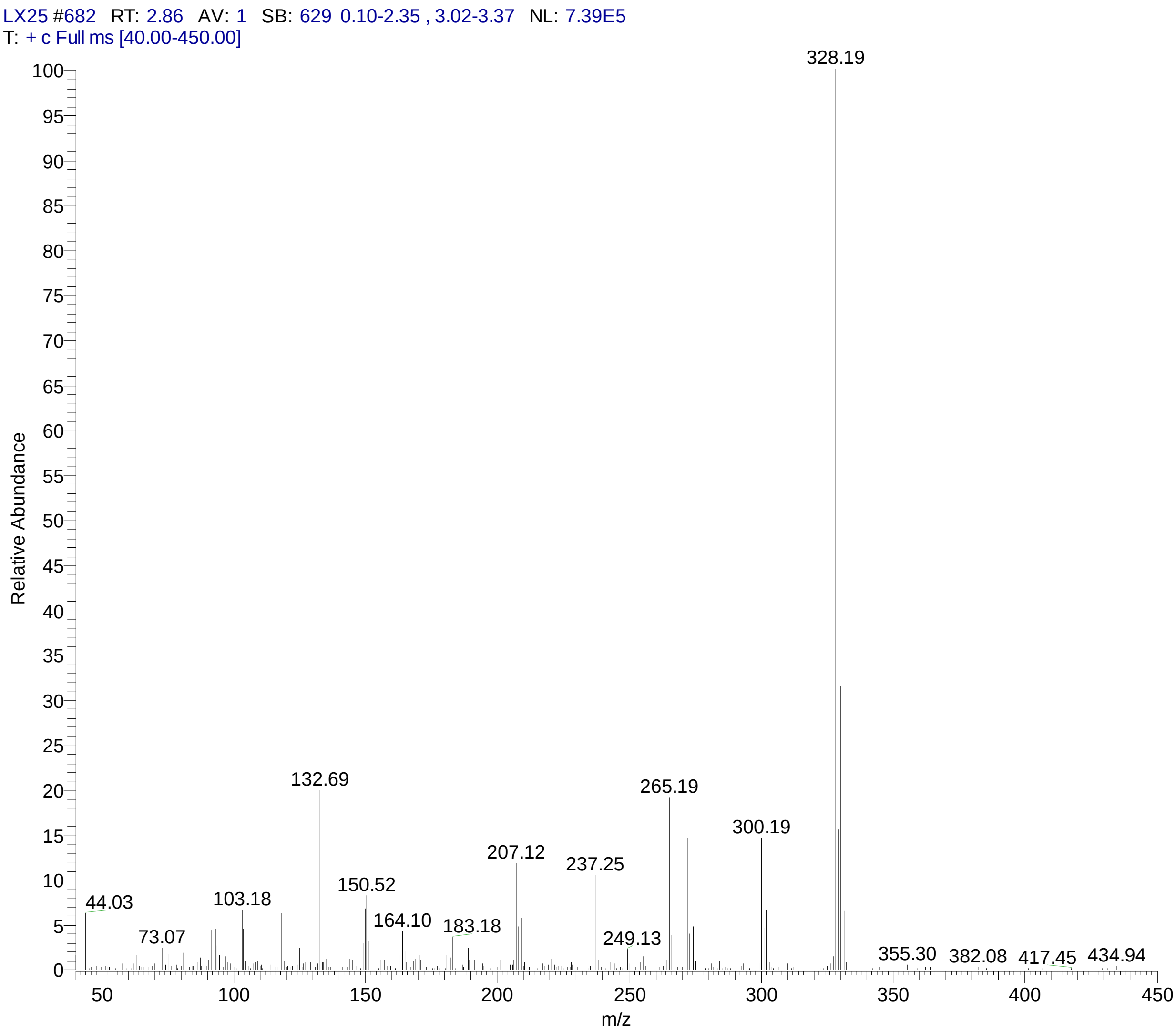
LX25 #682 RT: 2.86 AV: 1 SB: 629 0.10-2.35 , 3.02-3.37 NL: 7.39E5
T: + c Full ms [40.00-450.00]
Relative Abundance
m/z
328.19
132.69
265.19
300.19
207.12
237.25
150.52
103.18
44.03
164.10
183.18
73.07
249.13
355.30
382.08
417.45
434.94

MS of I8

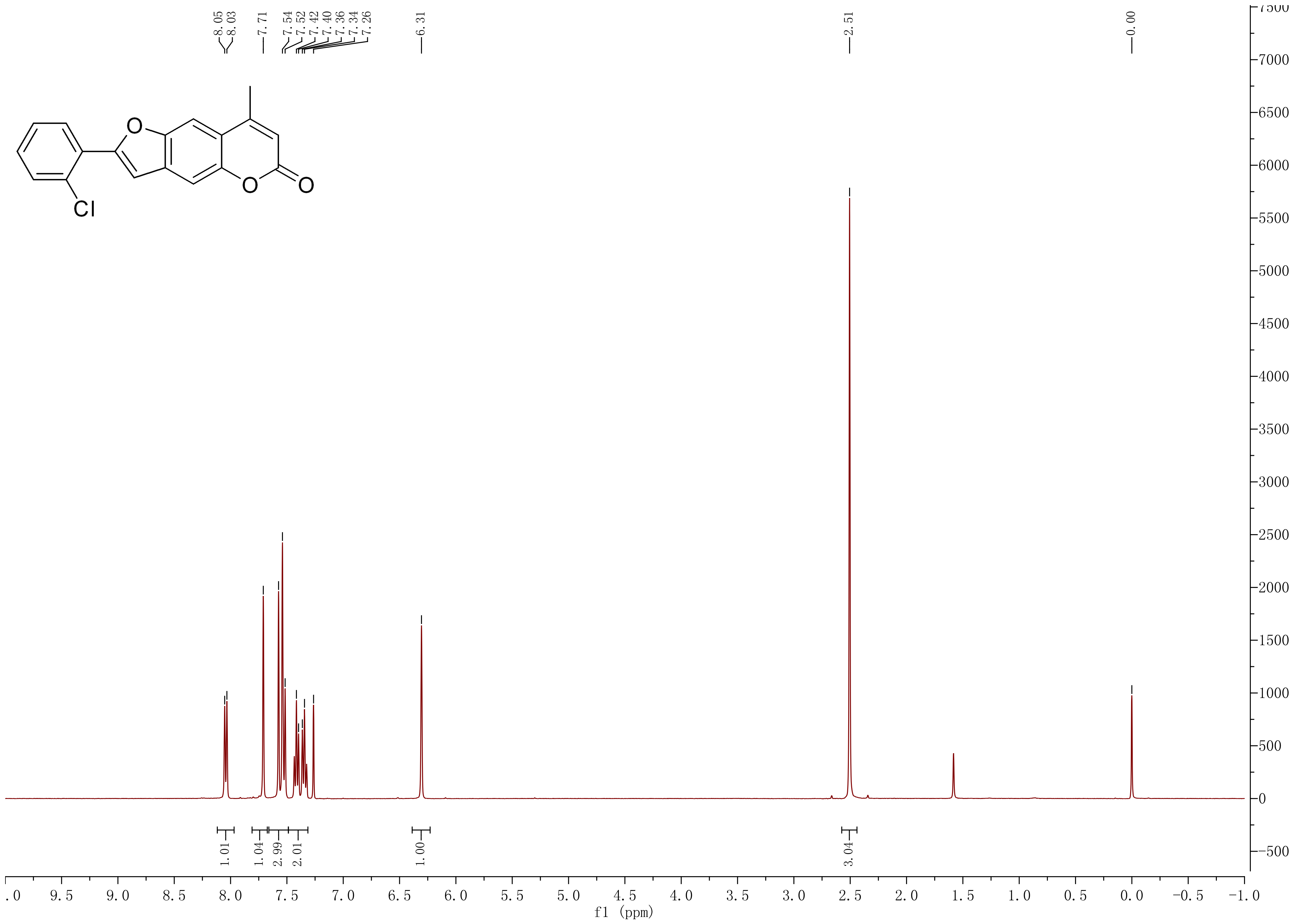

8.05
8.03
7.71
7.54
7.52
7.42
7.40
7.36
7.34
7.26
6.31
2.51
0.00
Cl
O
O
O
1.01
1.04
2.99
2.01
1.00
3.04
f1 (ppm)

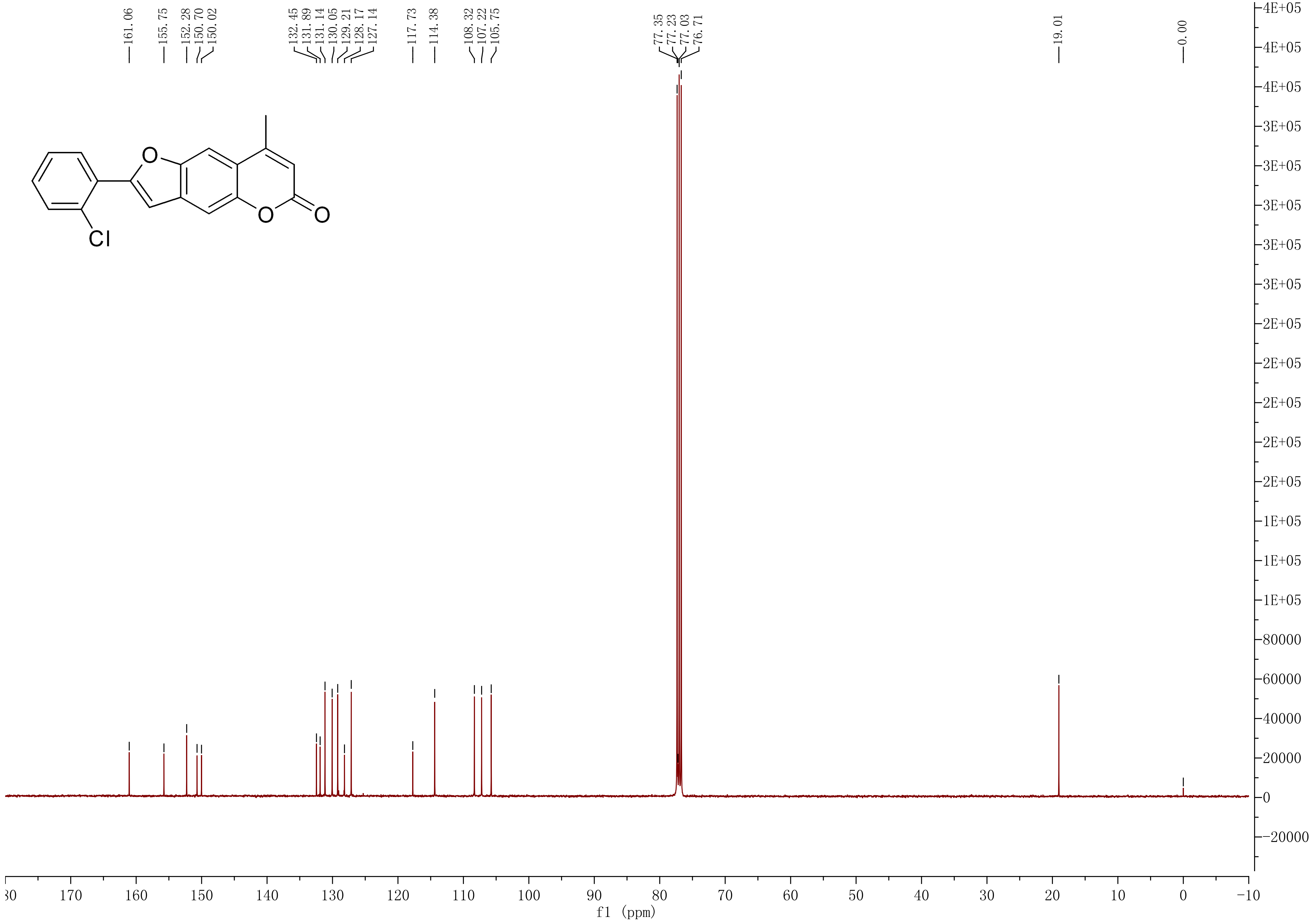

161.06
155.75
152.28
150.70
150.02
132.45
131.89
131.14
130.05
129.21
128.17
127.14
117.73
114.38
108.32
107.22
105.75
77.35
77.23
77.03
76.71
19.01
0.00
O
O
O
Cl
4E+05
3E+05
2E+05
1E+05
80000
60000
40000
20000
0
-20000
170
160
150
140
130
120
110
100
90
80
70
60
50
40
30
20
10
0
-10
f1 (ppm)

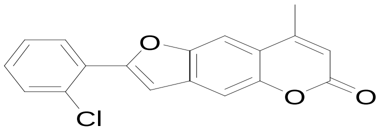

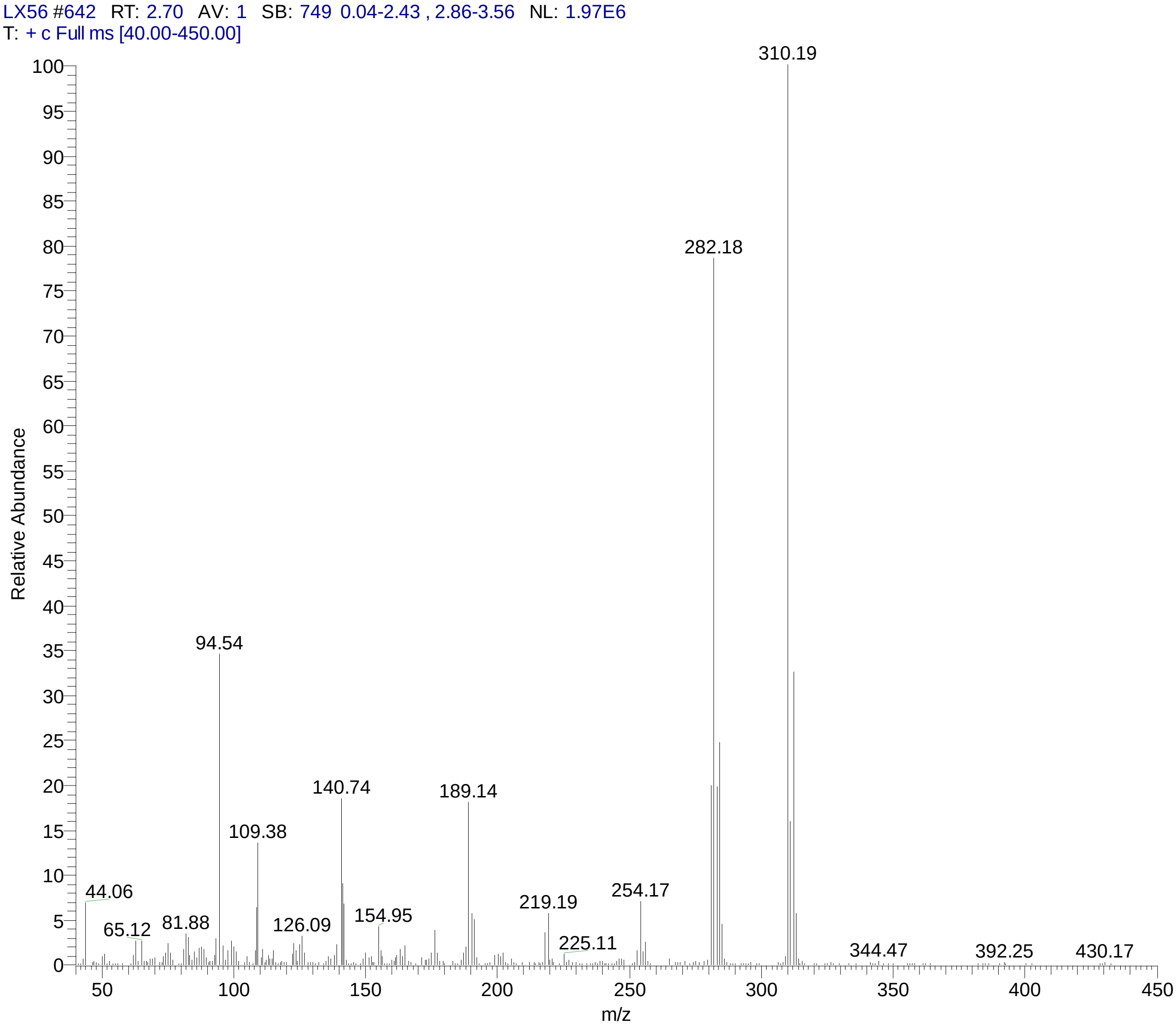

LX56 #642 RT: 2.70 AV: 1 SB: 749 0.04-2.43 , 2.86-3.56 NL: 1.97E6
T: + c Full ms [40.00-450.00]
310.19
282.18
94.54
140.74
189.14
109.38
44.06
65.12
81.88
126.09
154.95
219.19
225.11
254.17
344.47
392.25
430.17
Relative Abundance
m/z

MS of I9

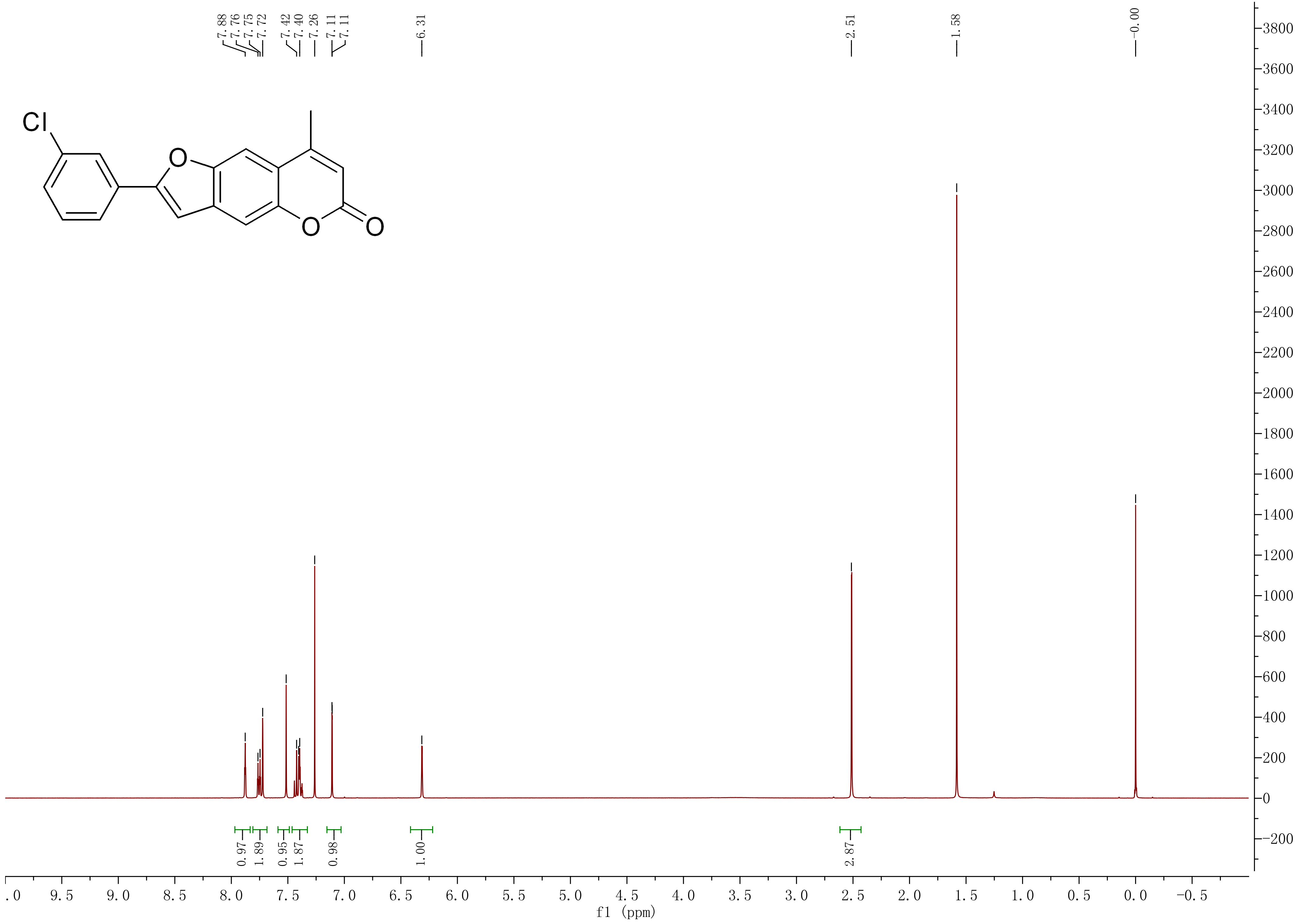

7.88
7.76
7.75
7.72
7.42
7.40
7.26
7.11
7.11
6.31
2.51
1.58
-0.00
Cl
O
O
O
0.97
1.89
0.95
1.87
0.98
1.00
2.87
f1 (ppm)

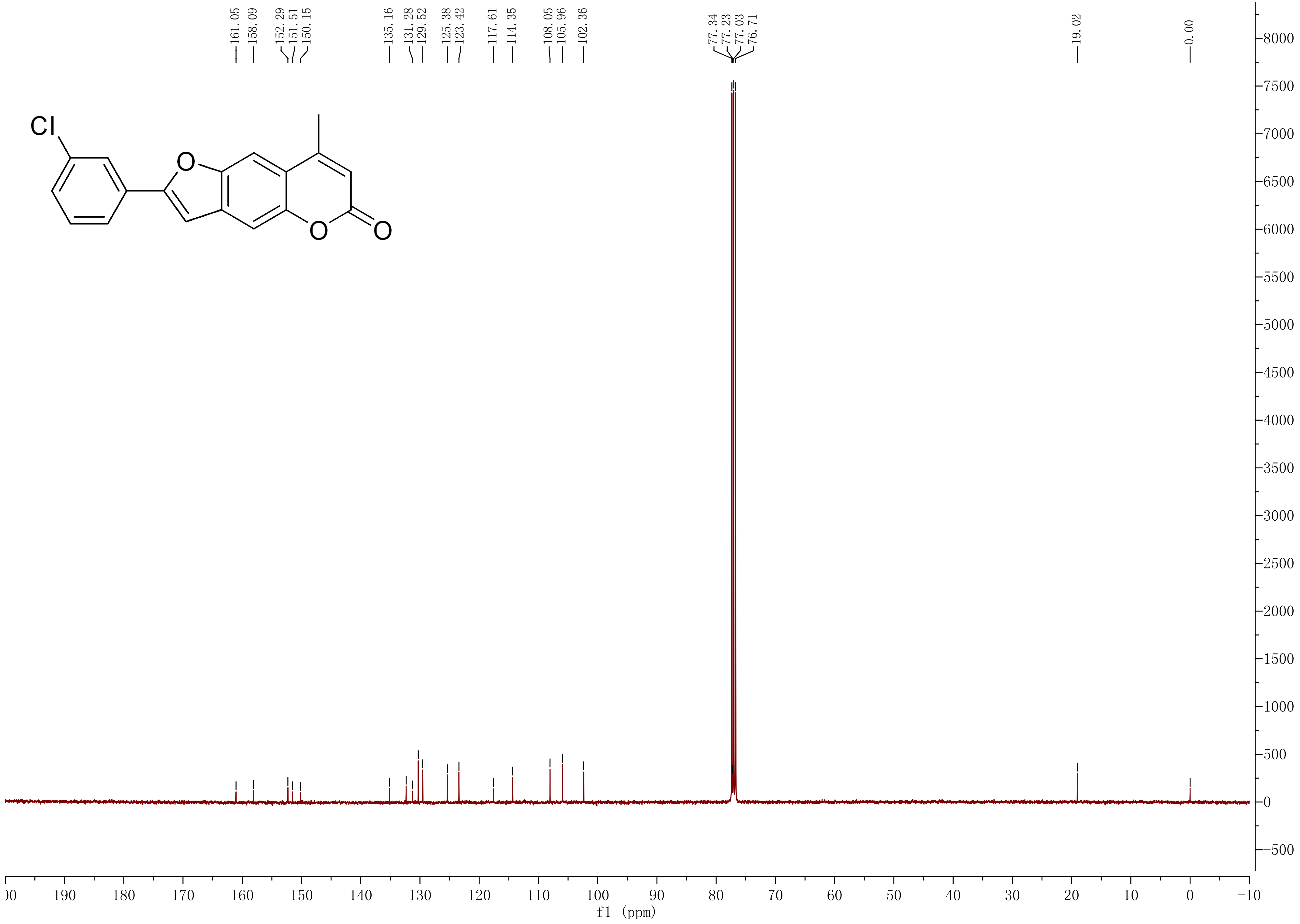

161.05
158.09
152.29
151.51
150.15
135.16
131.28
129.52
125.38
123.42
117.61
114.35
108.05
105.96
102.36
77.34
77.23
77.03
76.71
19.02
0.00
f1 (ppm)

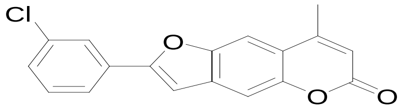
Cl
O
O
O

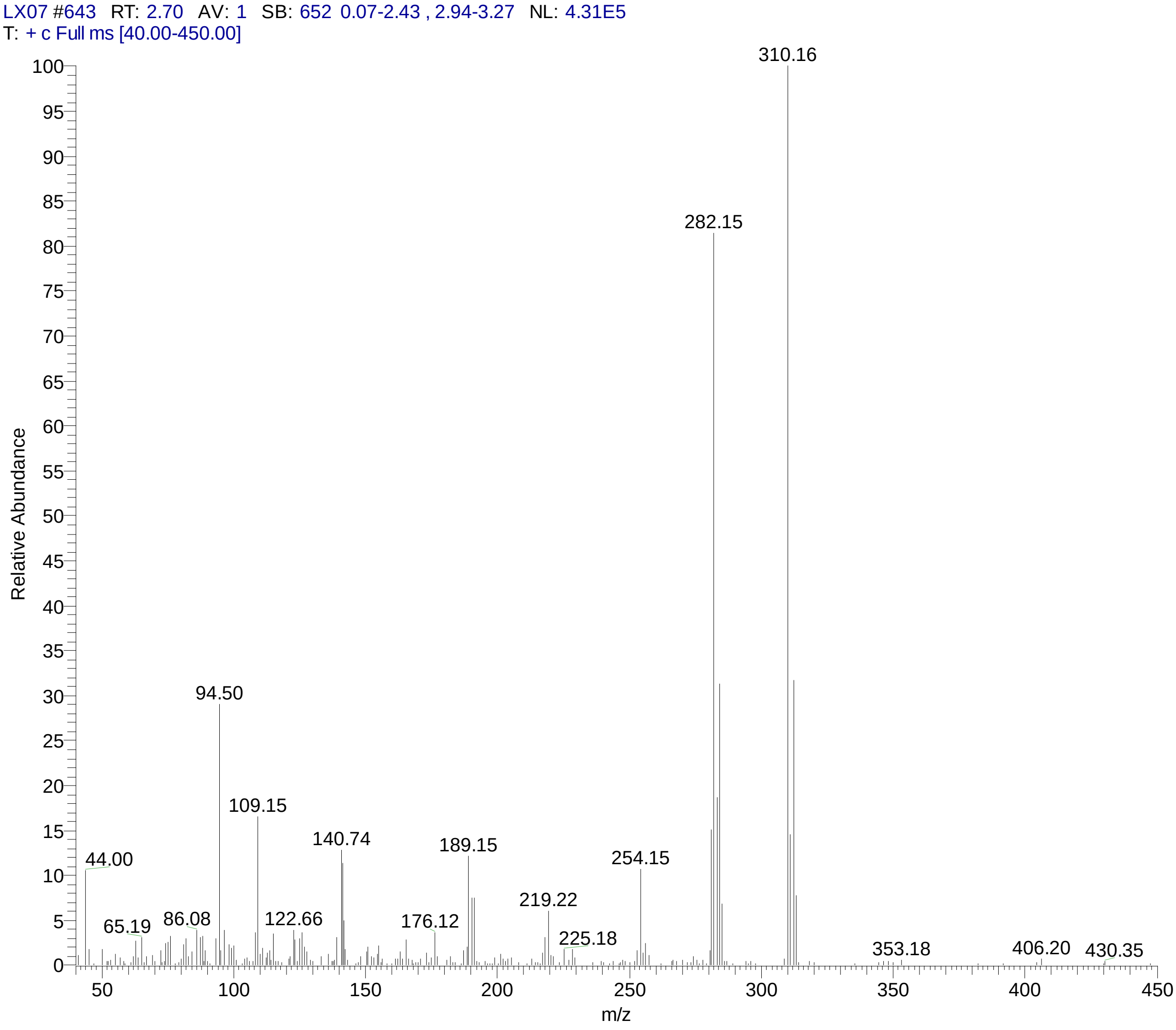
LX07 #643 RT: 2.70 AV: 1 SB: 652 0.07-2.43 , 2.94-3.27 NL: 4.31E5
T: + c Full ms [40.00-450.00]
Relative Abundance
m/z
310.16
282.15
94.50
109.15
140.74
189.15
254.15
219.22
44.00
65.19
86.08
122.66
176.12
225.18
353.18
406.20
430.35

MS of I10

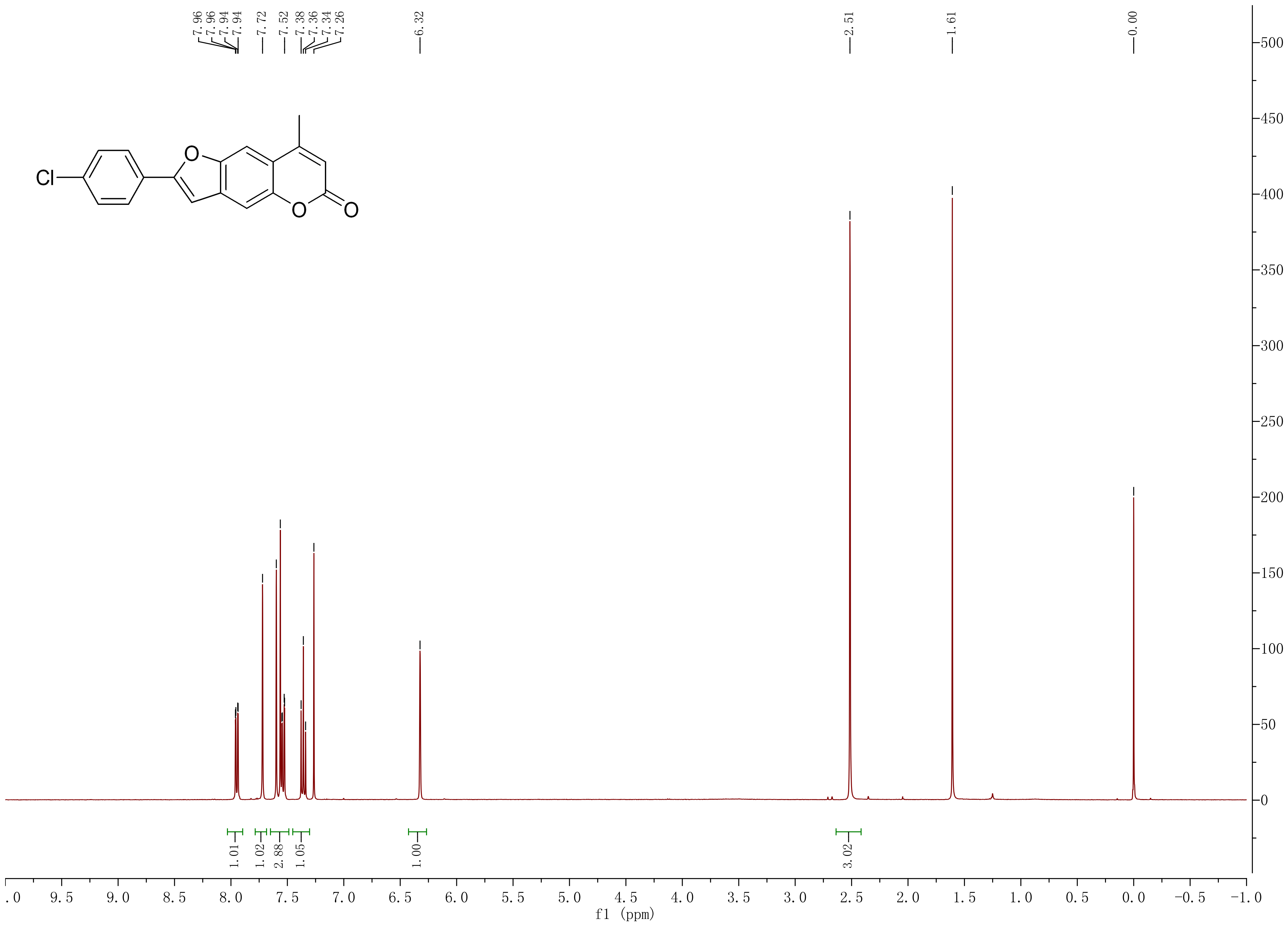

7.96
7.96
7.94
7.94
7.72
7.52
7.38
7.36
7.34
7.26
6.32
2.51
1.61
0.00
Cl
O
O
O
1.01
1.02
2.88
1.05
1.00
3.02
f1 (ppm)

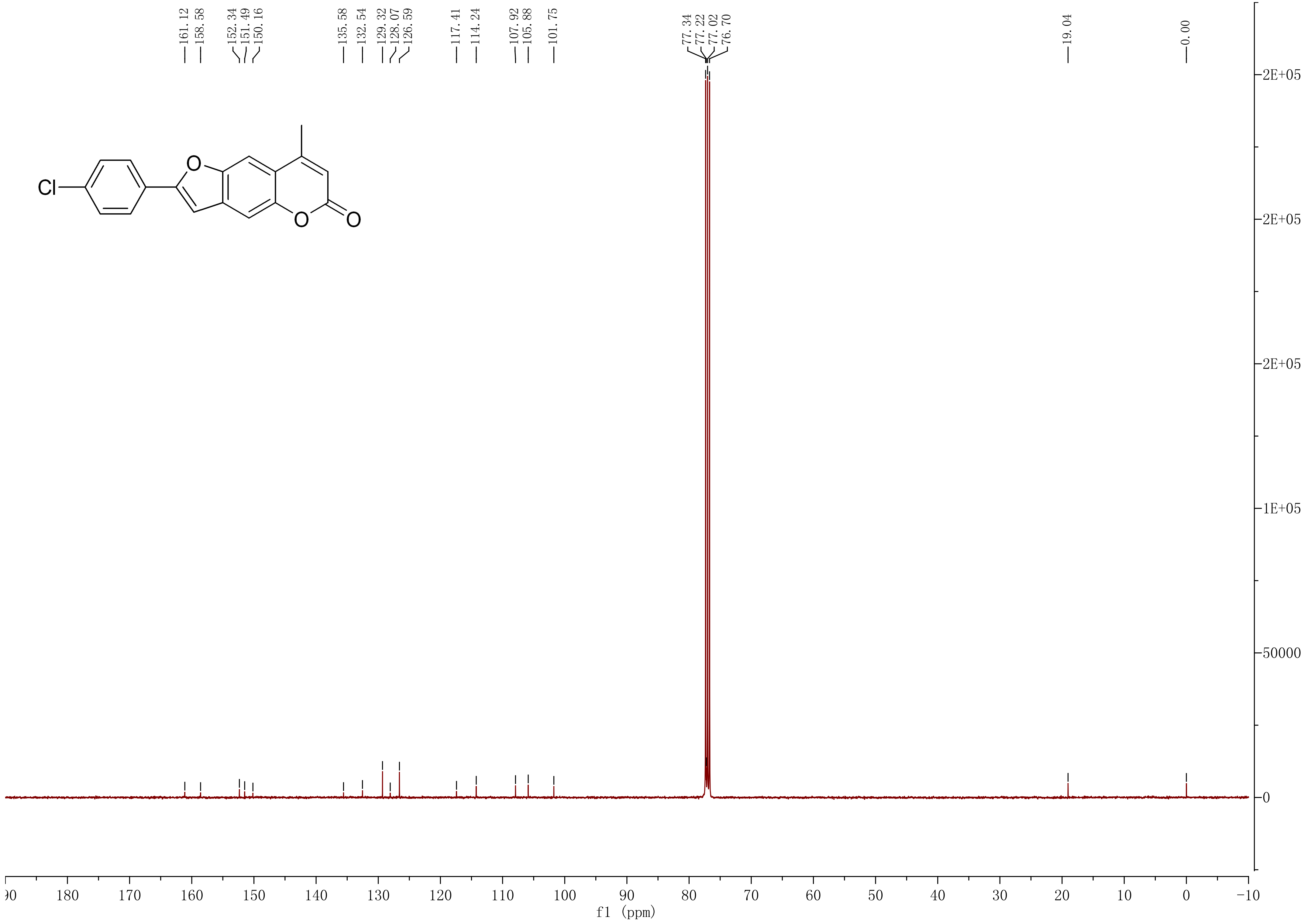
161.12
158.58
152.34
151.49
150.16
135.58
132.54
129.32
128.07
126.59
117.41
114.24
107.92
105.88
101.75
77.34
77.22
77.02
76.70
19.04
0.00
Cl
O
O
O
2E+05
2E+05
2E+05
1E+05
50000
0
180
170
160
150
140
130
120
110
100
90
80
70
60
50
40
30
20
10
0
−10
f1 (ppm)

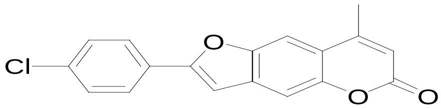

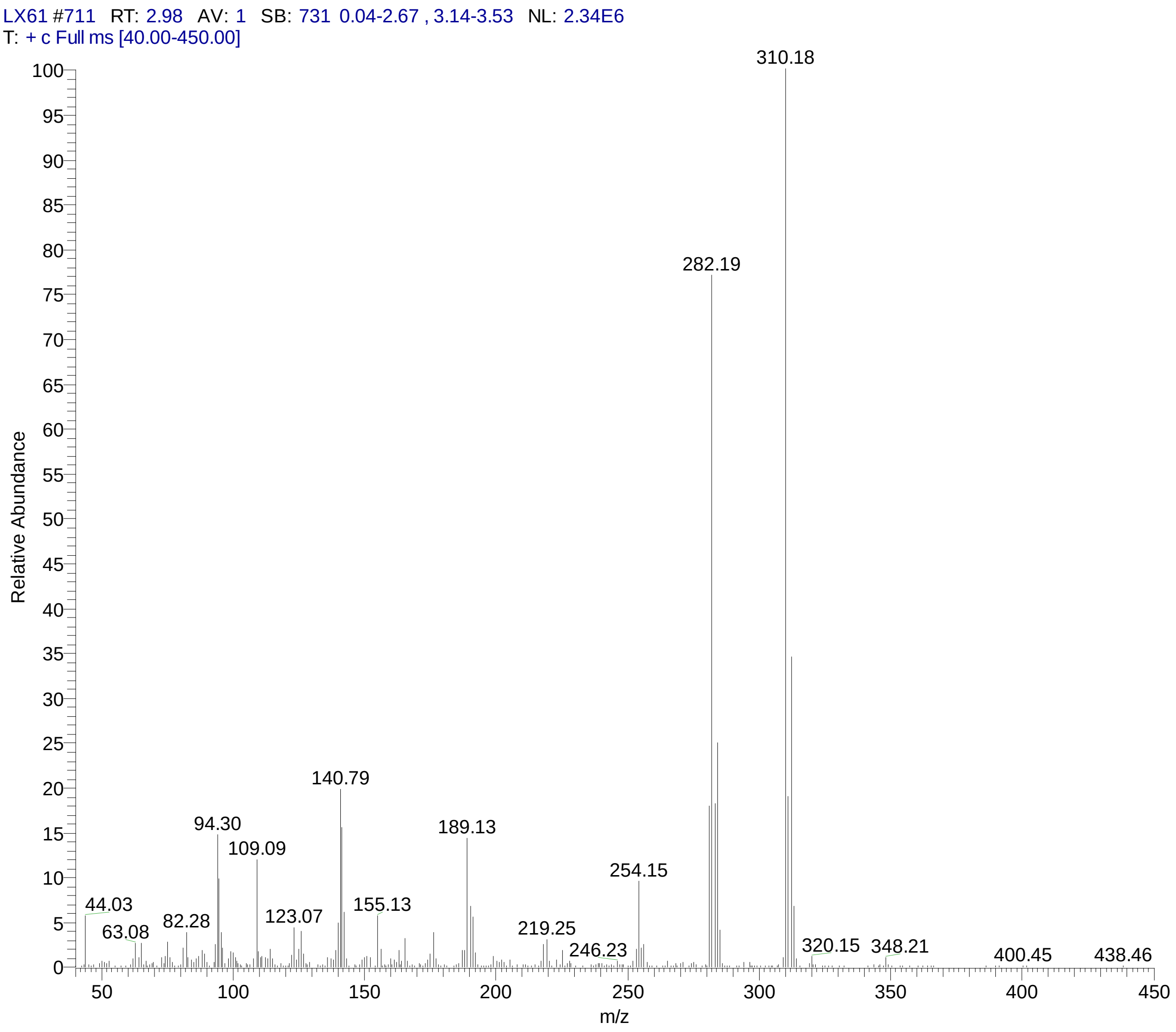
LX61 #711 RT: 2.98 AV: 1 SB: 731 0.04-2.67 , 3.14-3.53 NL: 2.34E6
T: + c Full ms [40.00-450.00]
Relative Abundance
m/z
310.18
282.19
140.79
94.30
109.09
189.13
254.15
44.03
63.08
82.28
123.07
155.13
219.25
246.23
320.15
348.21
400.45
438.46

MS of I11

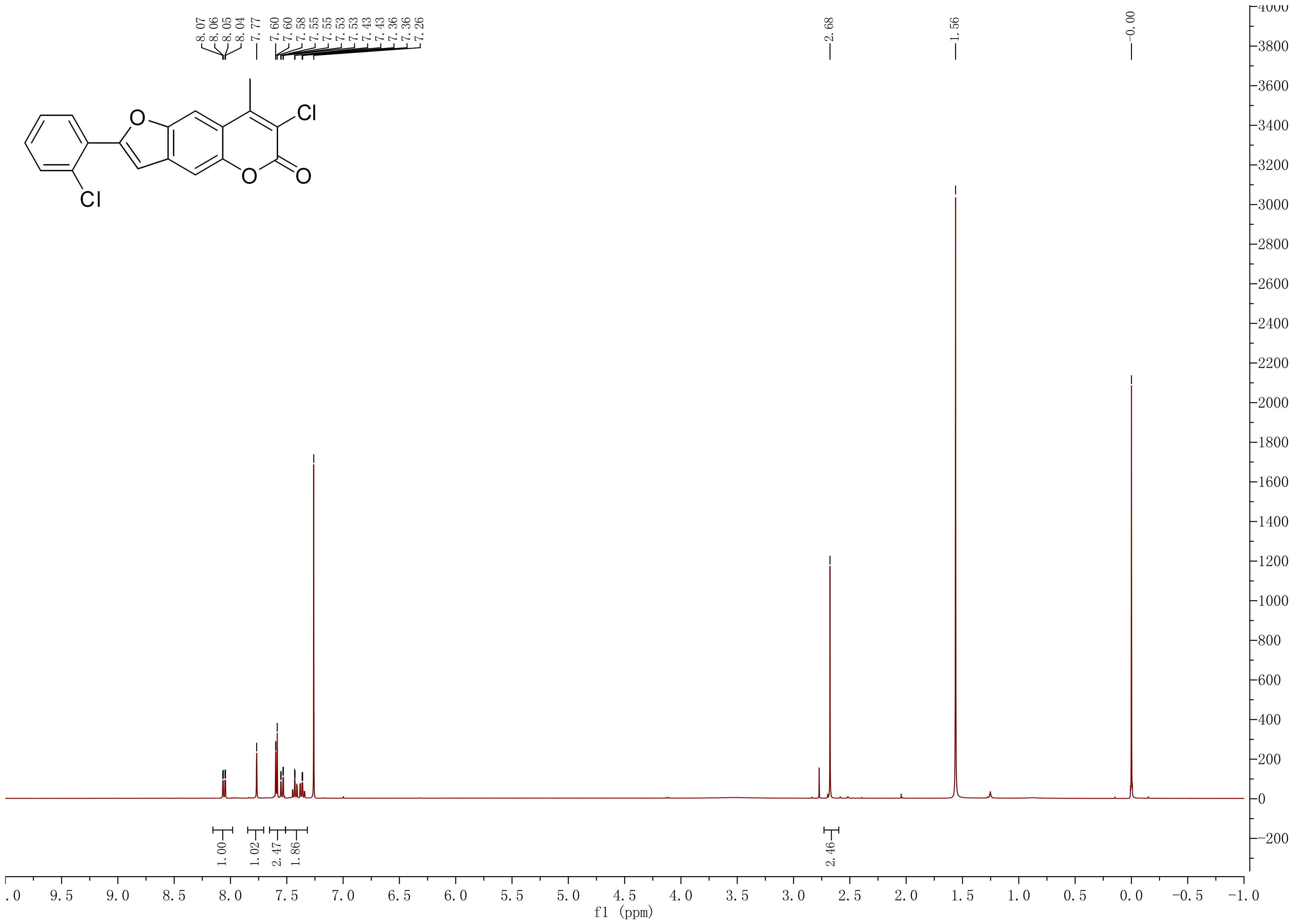
8.07
8.06
8.05
8.04
7.77
7.60
7.60
7.58
7.55
7.55
7.53
7.53
7.43
7.43
7.36
7.36
7.26
2.68
1.56
-0.00
Cl
O
O
O
Cl
1.00
1.02
2.47
1.86
2.46
f1 (ppm)

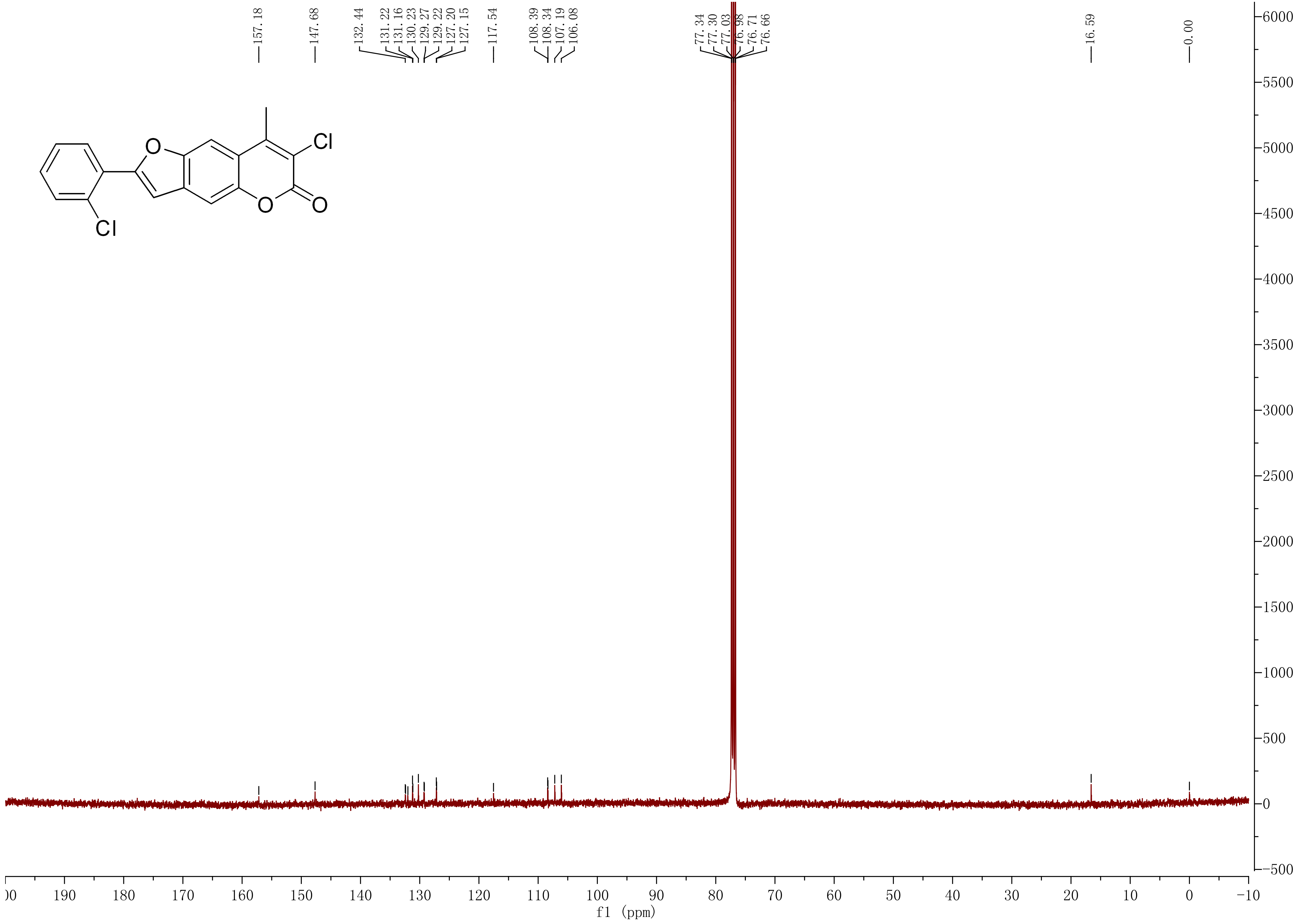
157.18
147.68
132.44
131.22
131.16
130.23
129.27
129.22
127.20
127.15
117.54
108.39
108.34
107.19
106.08
77.34
77.30
77.03
76.98
76.71
76.66
16.59
0.00
O
Cl
O
O
Cl
f1 (ppm)

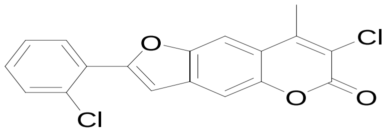
O
Cl
O
O
Cl

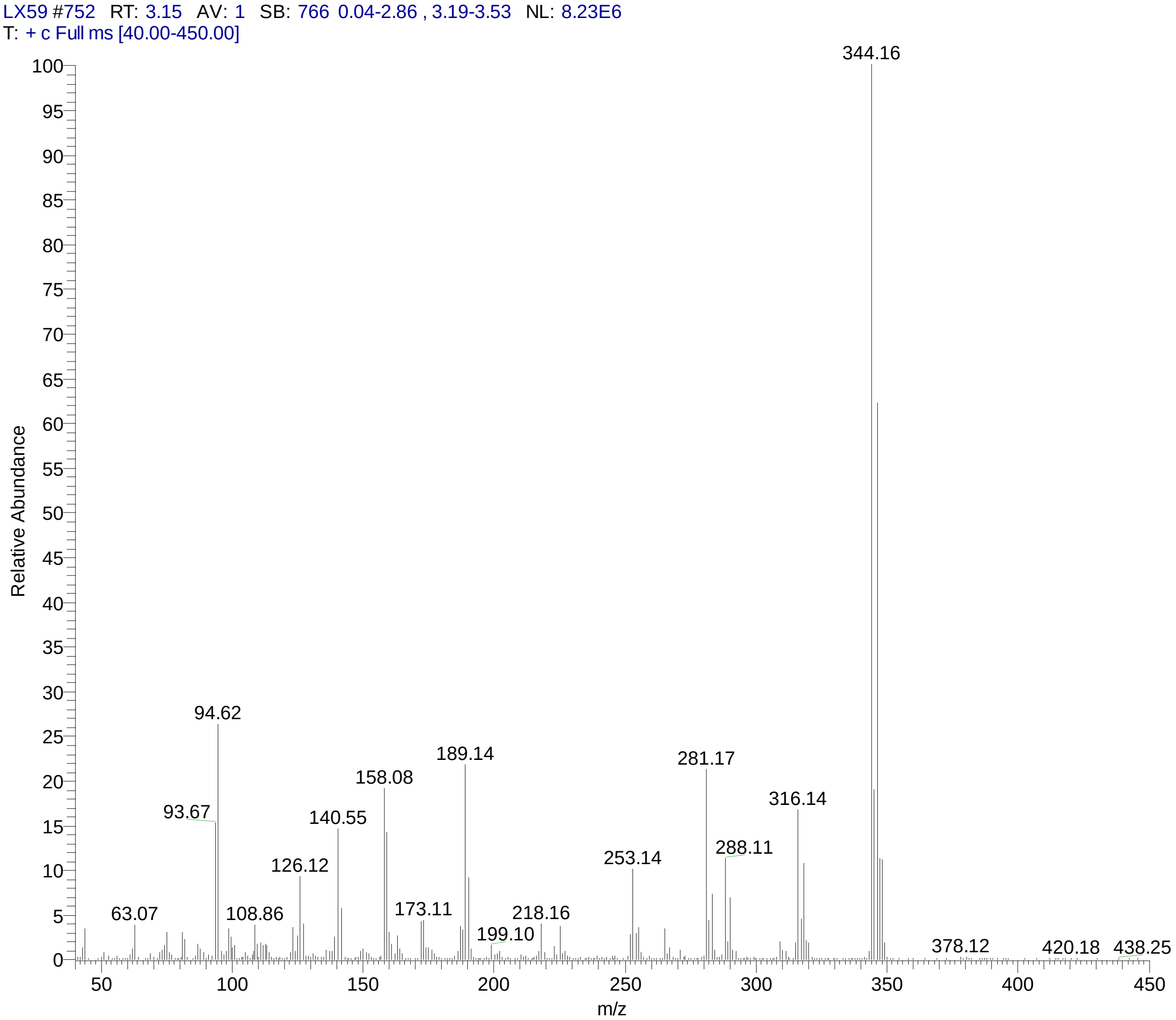
LX59 #752 RT: 3.15 AV: 1 SB: 766 0.04-2.86 , 3.19-3.53 NL: 8.23E6
T: + c Full ms [40.00-450.00]
Relative Abundance
m/z
344.16
94.62
93.67
189.14
158.08
140.55
126.12
63.07
108.86
173.11
199.10
218.16
253.14
281.17
288.11
316.14
378.12
420.18
438.25

MS of I12

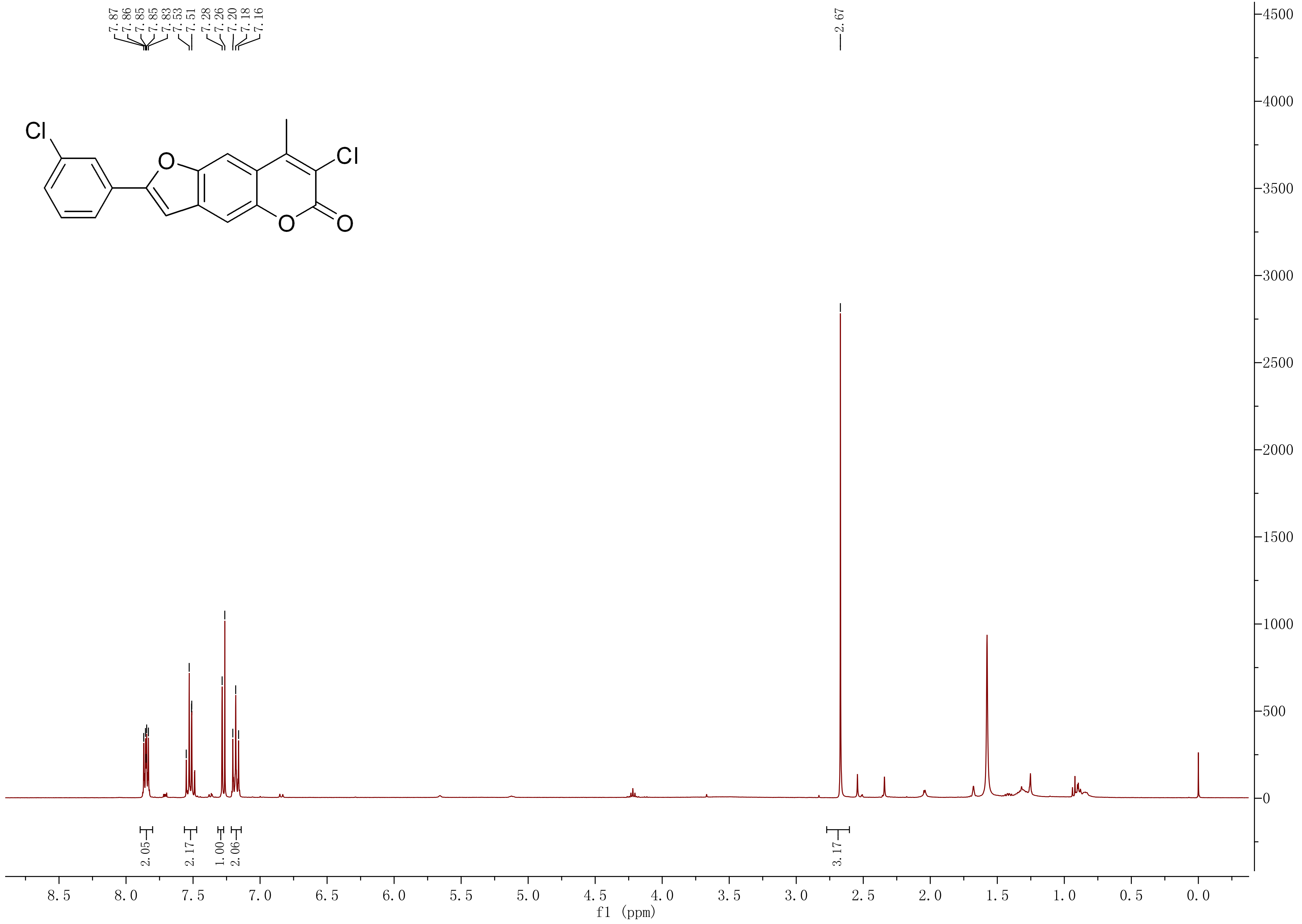
7.87
7.86
7.85
7.85
7.83
7.53
7.51
7.28
7.26
7.20
7.18
7.16
2.67
Cl
O
Cl
O
O
2.05
2.17
1.00
2.06
3.17
8.5
8.0
7.5
7.0
6.5
6.0
5.5
5.0
4.5
4.0
3.5
3.0
2.5
2.0
1.5
1.0
0.5
0.0
f1 (ppm)
4500
4000
3500
3000
2500
2000
1500
1000
500
0

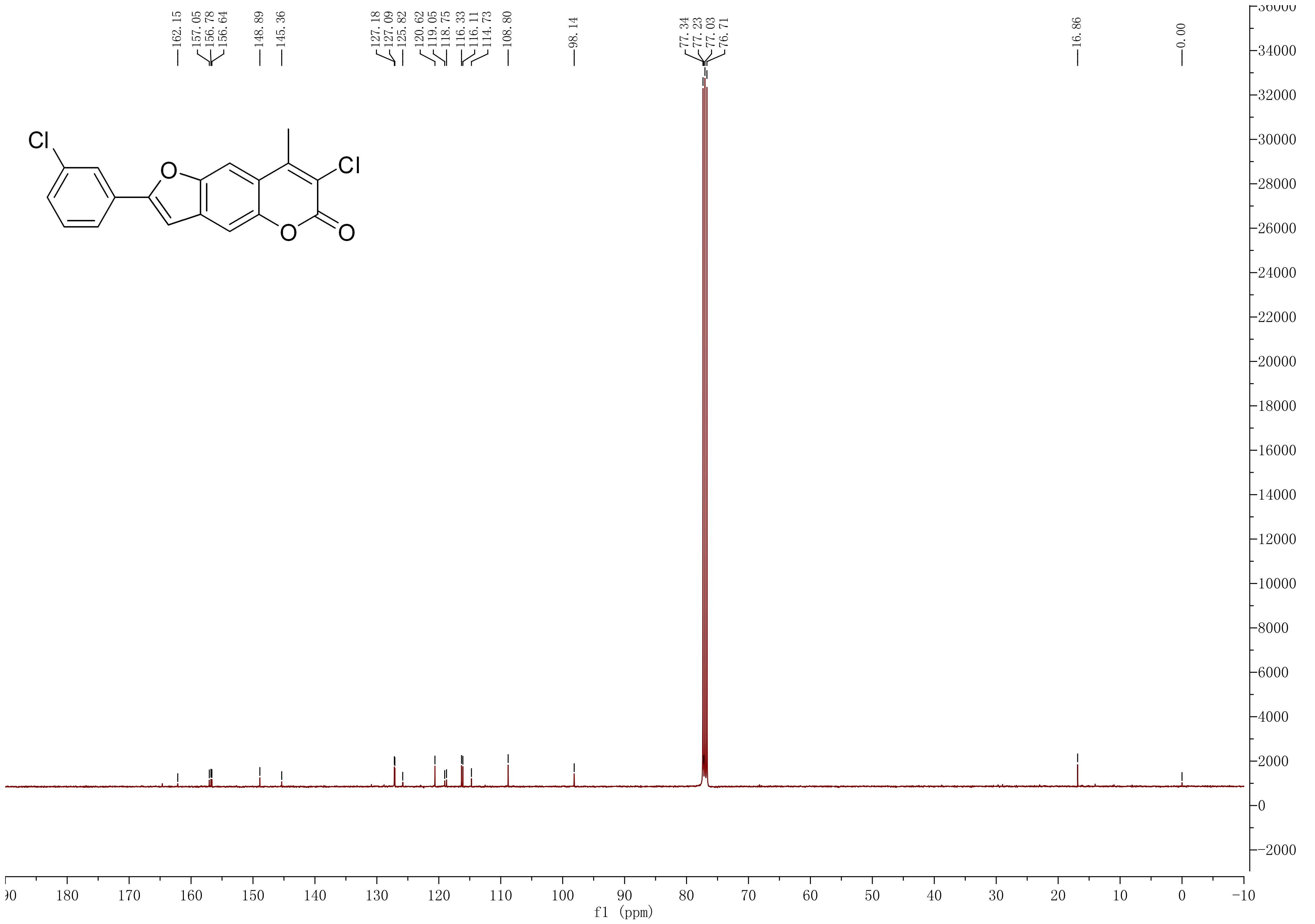
162.15
157.05
156.78
156.64
148.89
145.36
127.18
127.09
125.82
120.62
119.05
118.75
116.33
116.11
114.73
108.80
98.14
77.34
77.23
77.03
76.71
16.86
0.00
f1 (ppm)

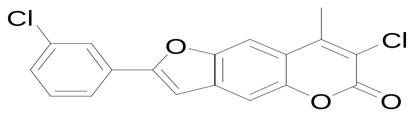

Cl
O
Cl
O
O

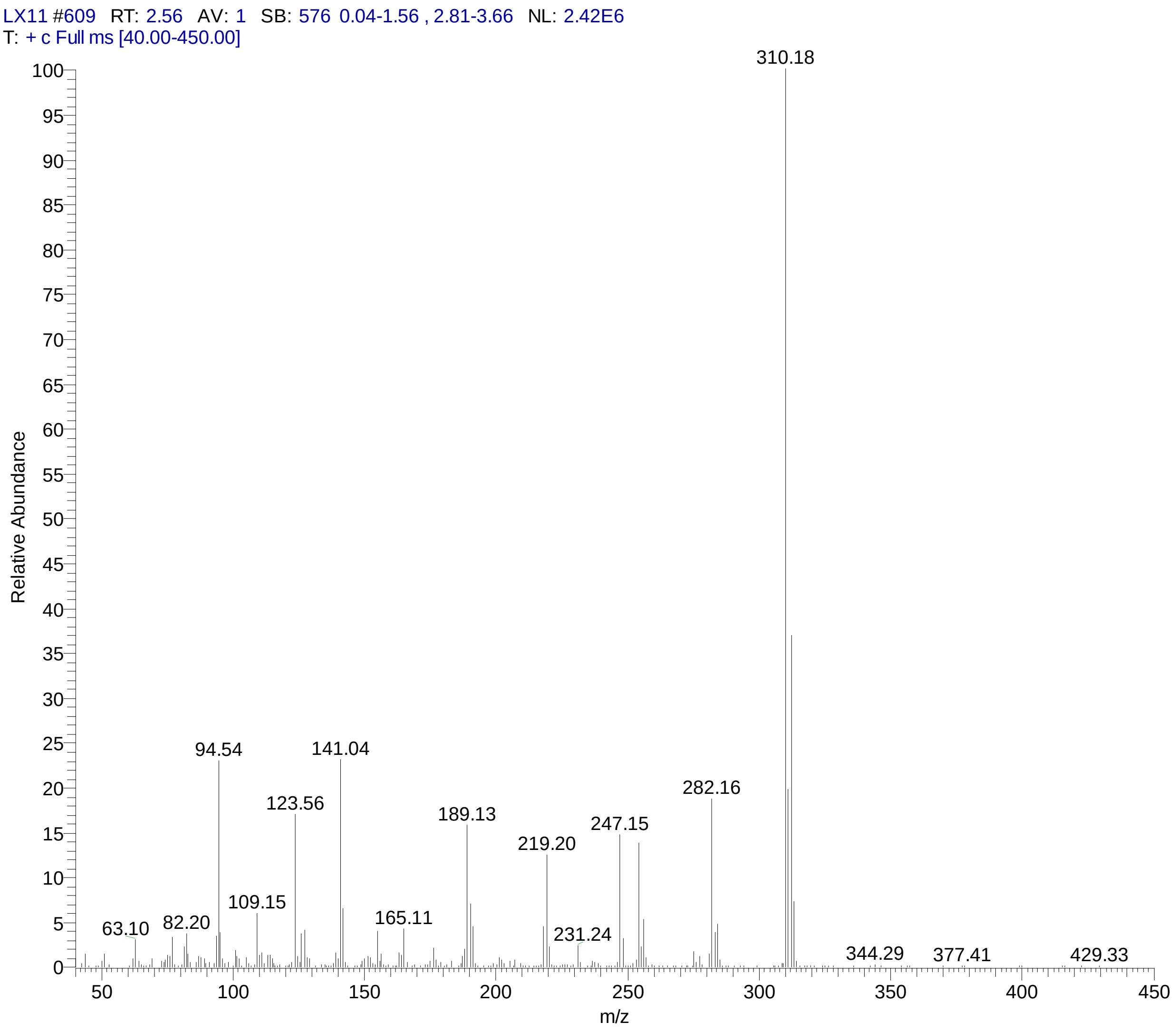

LX11 #609 RT: 2.56 AV: 1 SB: 576 0.04-1.56 , 2.81-3.66 NL: 2.42E6
T: + c Full ms [40.00-450.00]
Relative Abundance
m/z
310.18
282.16
247.15
219.20
231.24
189.13
165.11
141.04
123.56
109.15
94.54
82.20
63.10
344.29
377.41
429.33

MS of I13

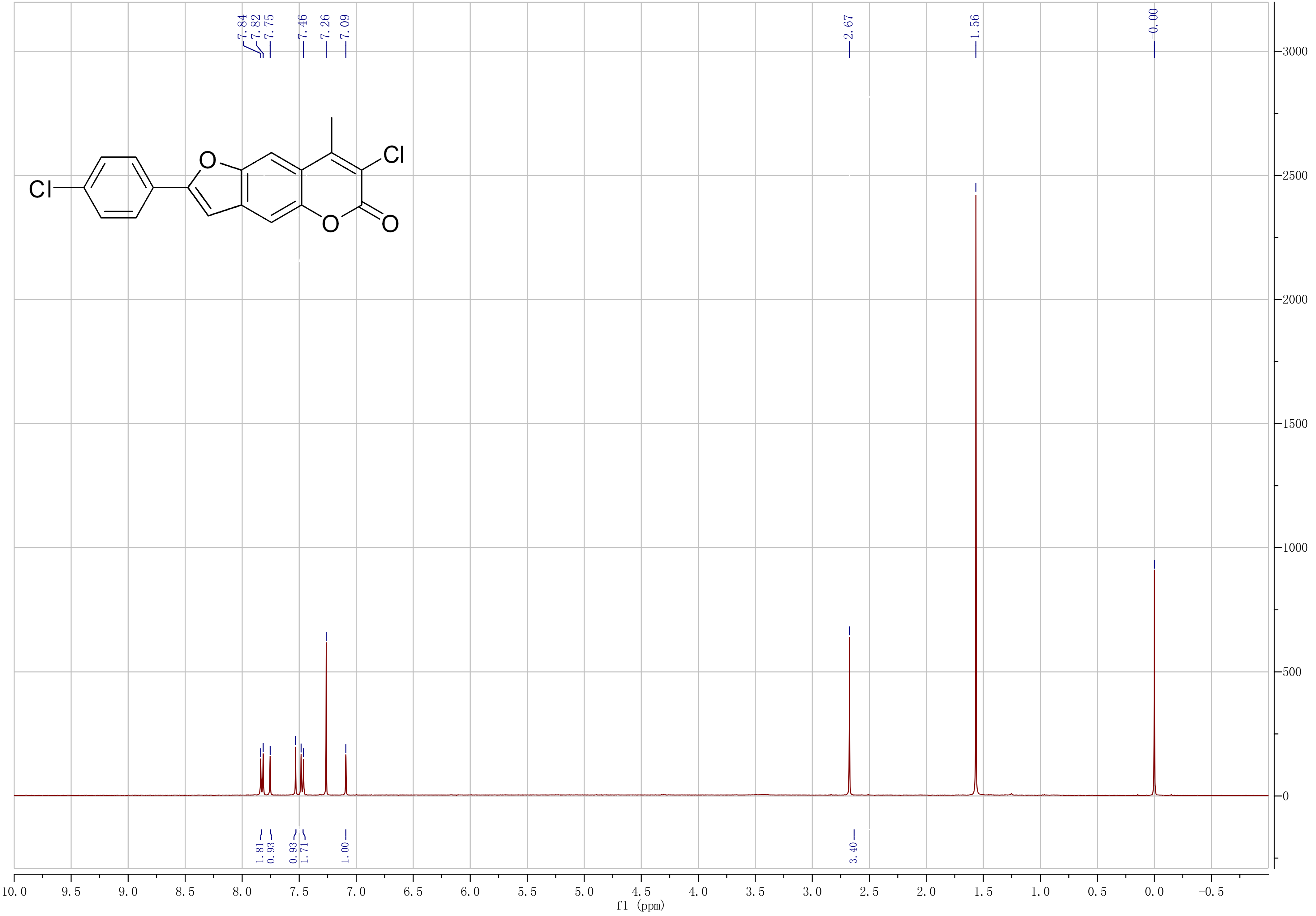

7.84
7.82
7.75
7.46
7.26
7.09
2.67
1.56
-0.00
Cl
O
Cl
O
O
1.81
0.93
0.93
1.71
1.00
3.40
10.0
9.5
9.0
8.5
8.0
7.5
7.0
6.5
6.0
5.5
5.0
4.5
4.0
3.5
3.0
2.5
2.0
1.5
1.0
0.5
0.0
-0.5
f1 (ppm)
3000
2500
2000
1500
1000
500
0

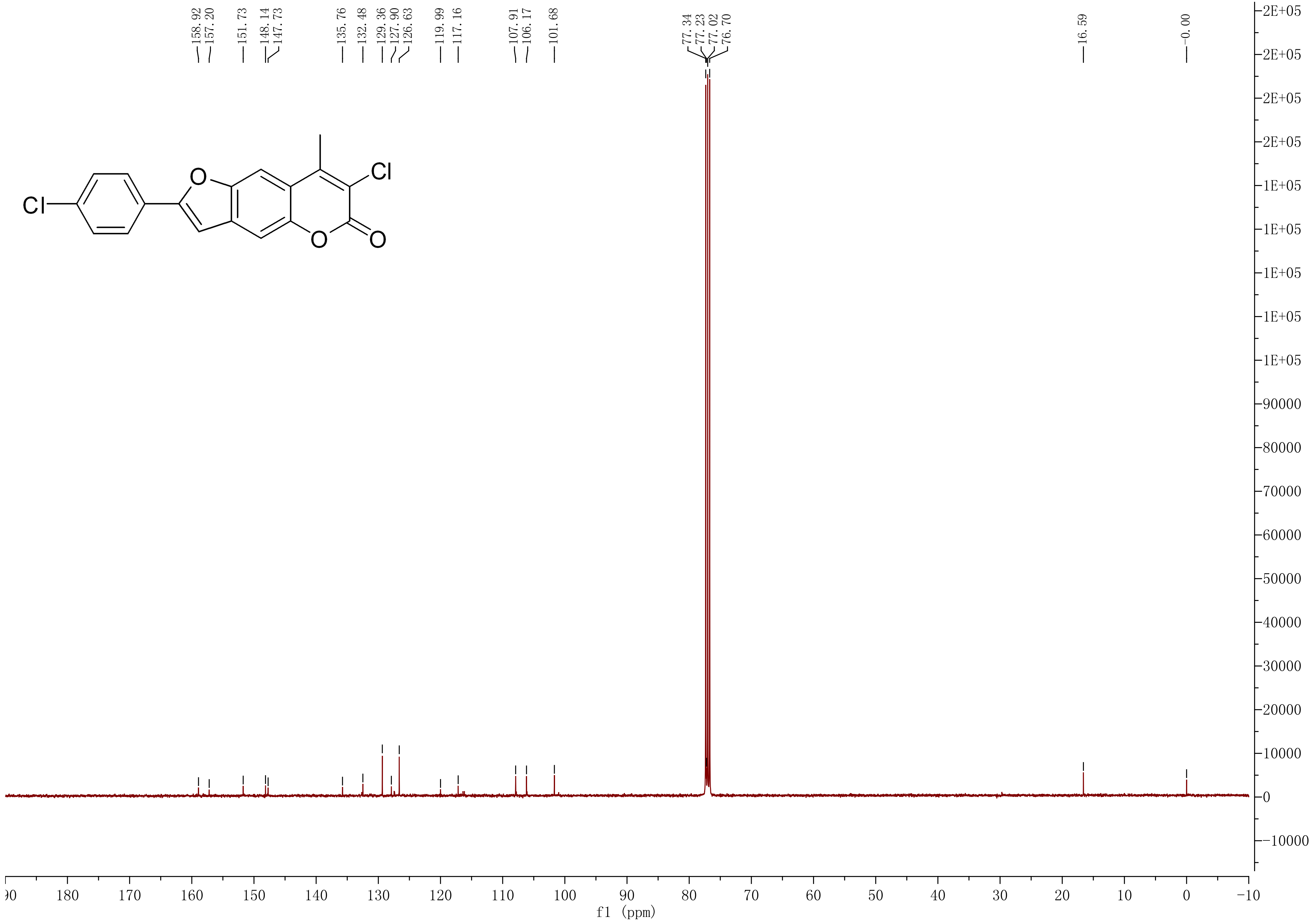

158.92
157.20
151.73
148.14
147.73
135.76
132.48
129.36
127.90
126.63
119.99
117.16
107.91
106.17
101.68
77.34
77.23
77.02
76.70
16.59
-0.00
Cl
O
Cl
O
O
f1 (ppm)

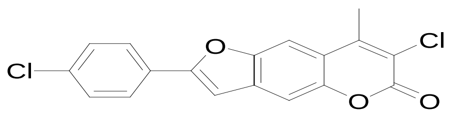

Cl
O
Cl
O
O

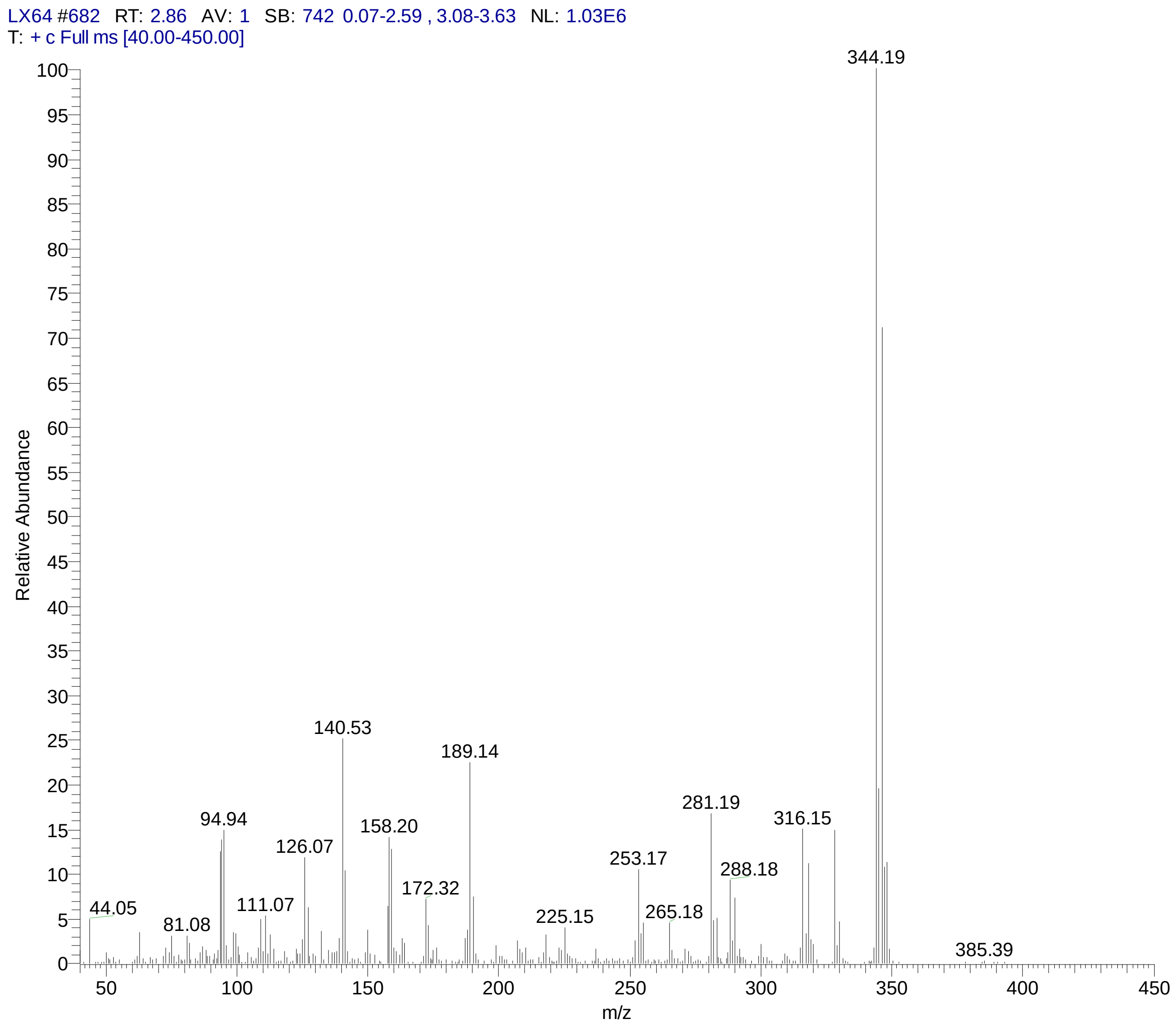

LX64 #682 RT: 2.86 AV: 1 SB: 742 0.07-2.59 , 3.08-3.63 NL: 1.03E6
T: + c Full ms [40.00-450.00]
344.19
140.53
189.14
94.94
158.20
126.07
281.19
316.15
253.17
288.18
172.32
111.07
225.15
265.18
44.05
81.08
385.39
Relative Abundance
m/z

MS of I14

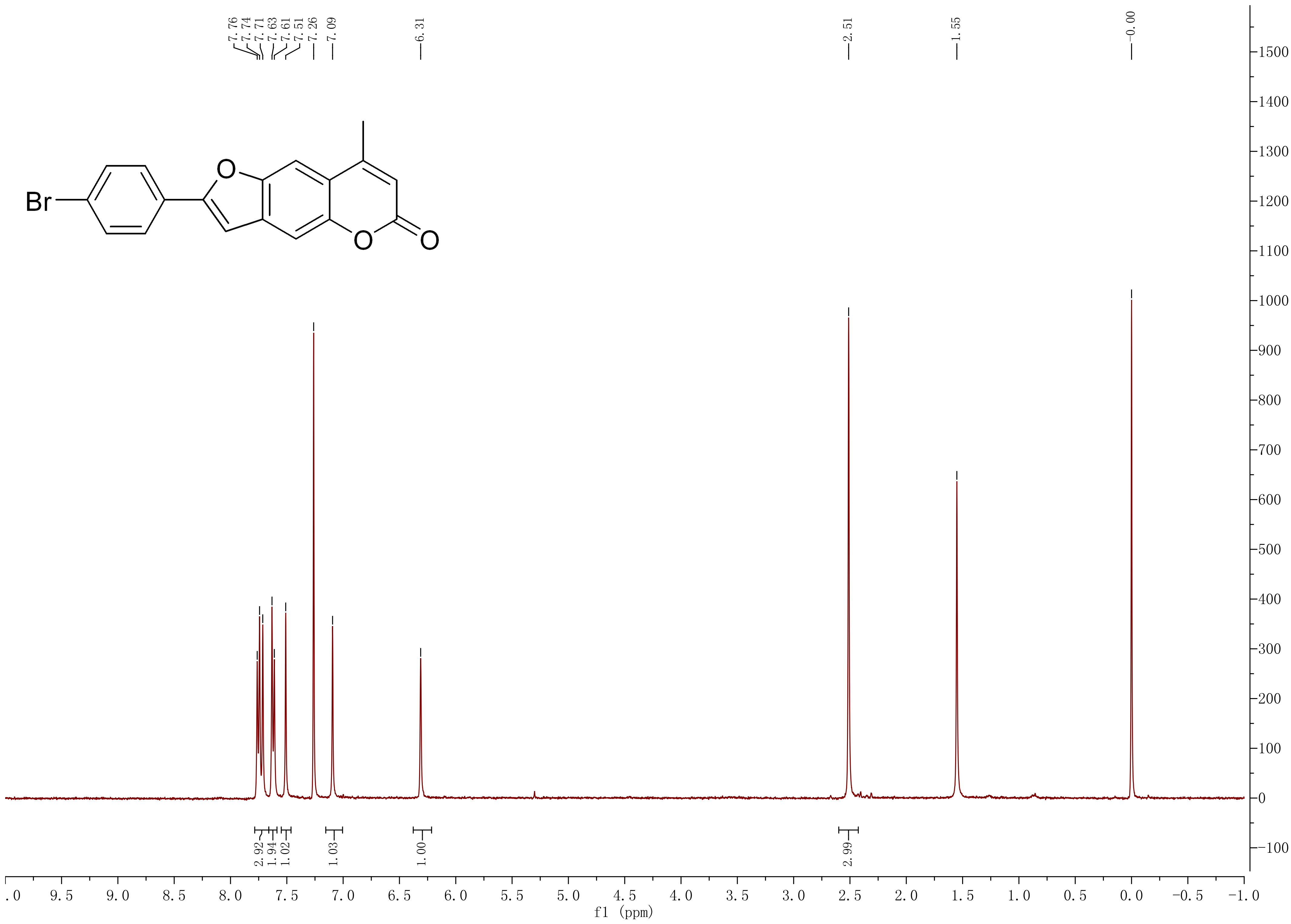
7.76
7.74
7.71
7.63
7.61
7.51
7.26
7.09
6.31
2.51
1.55
-0.00
Br
O
O
O
2.92
1.94
1.02
1.03
1.00
2.99
f1 (ppm)

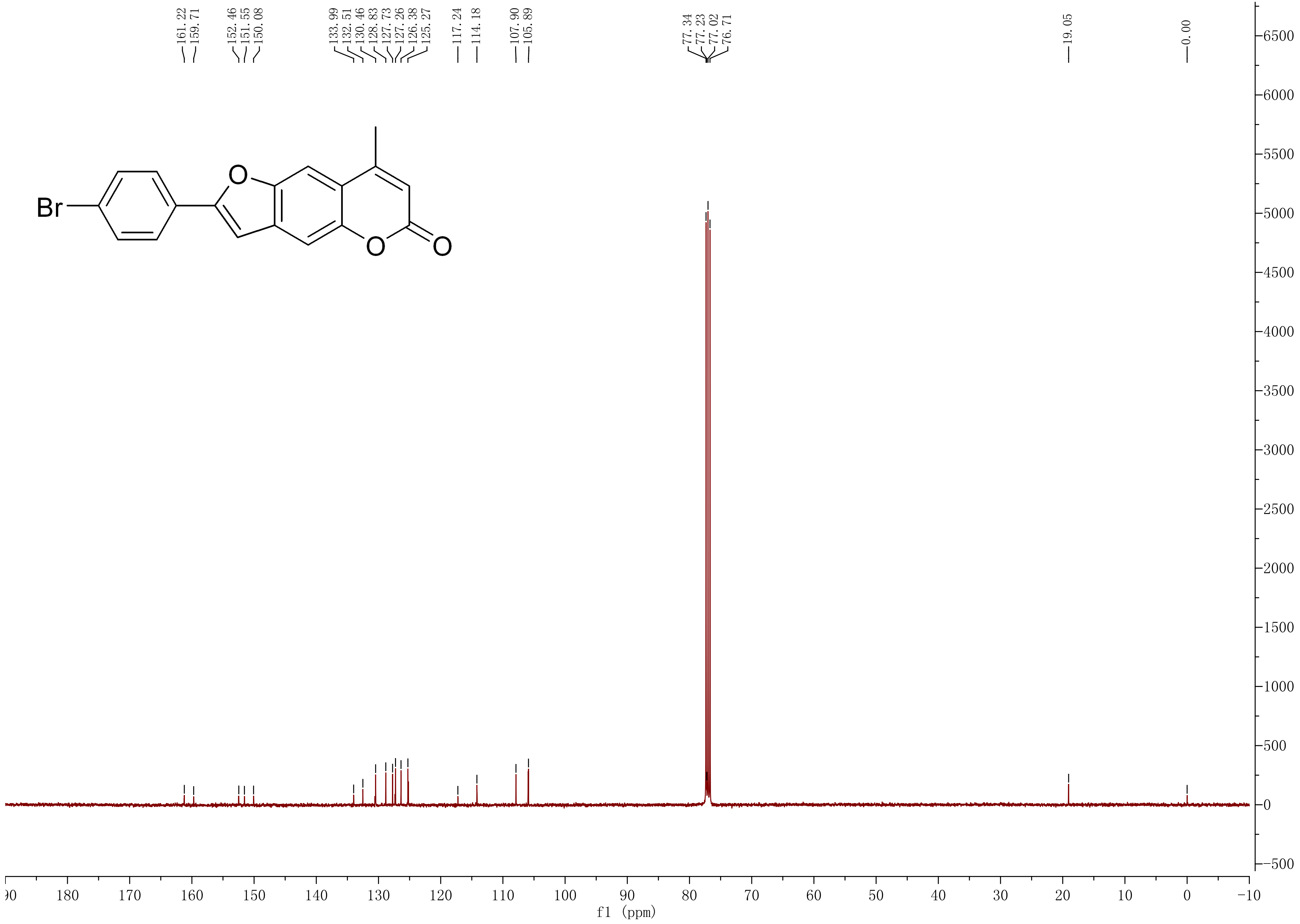
161.22
159.71
152.46
151.55
150.08
133.99
132.51
130.46
128.83
127.73
127.26
126.38
125.27
117.24
114.18
107.90
105.89
77.34
77.23
77.02
76.71
19.05
0.00
Br
O
O
O
f1 (ppm)

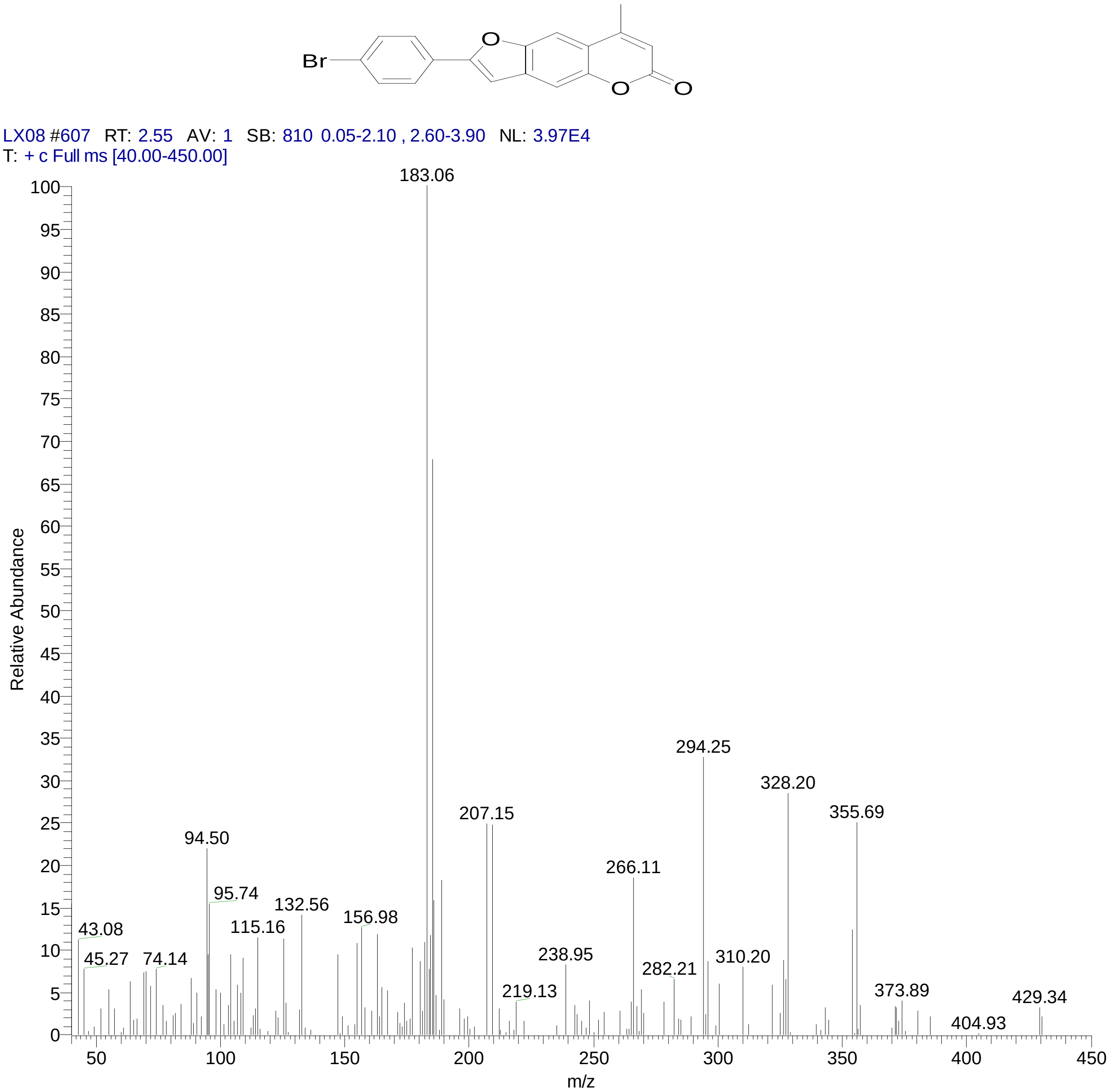
Br
O
O
O
LX08 #607 RT: 2.55 AV: 1 SB: 810 0.05-2.10 , 2.60-3.90 NL: 3.97E4
T: + c Full ms [40.00-450.00]
Relative Abundance
m/z
183.06
294.25
328.20
207.15
355.69
94.50
266.11
95.74
132.56
156.98
115.16
43.08
238.95
45.27
74.14
282.21
310.20
219.13
373.89
429.34
404.93

MS of I15

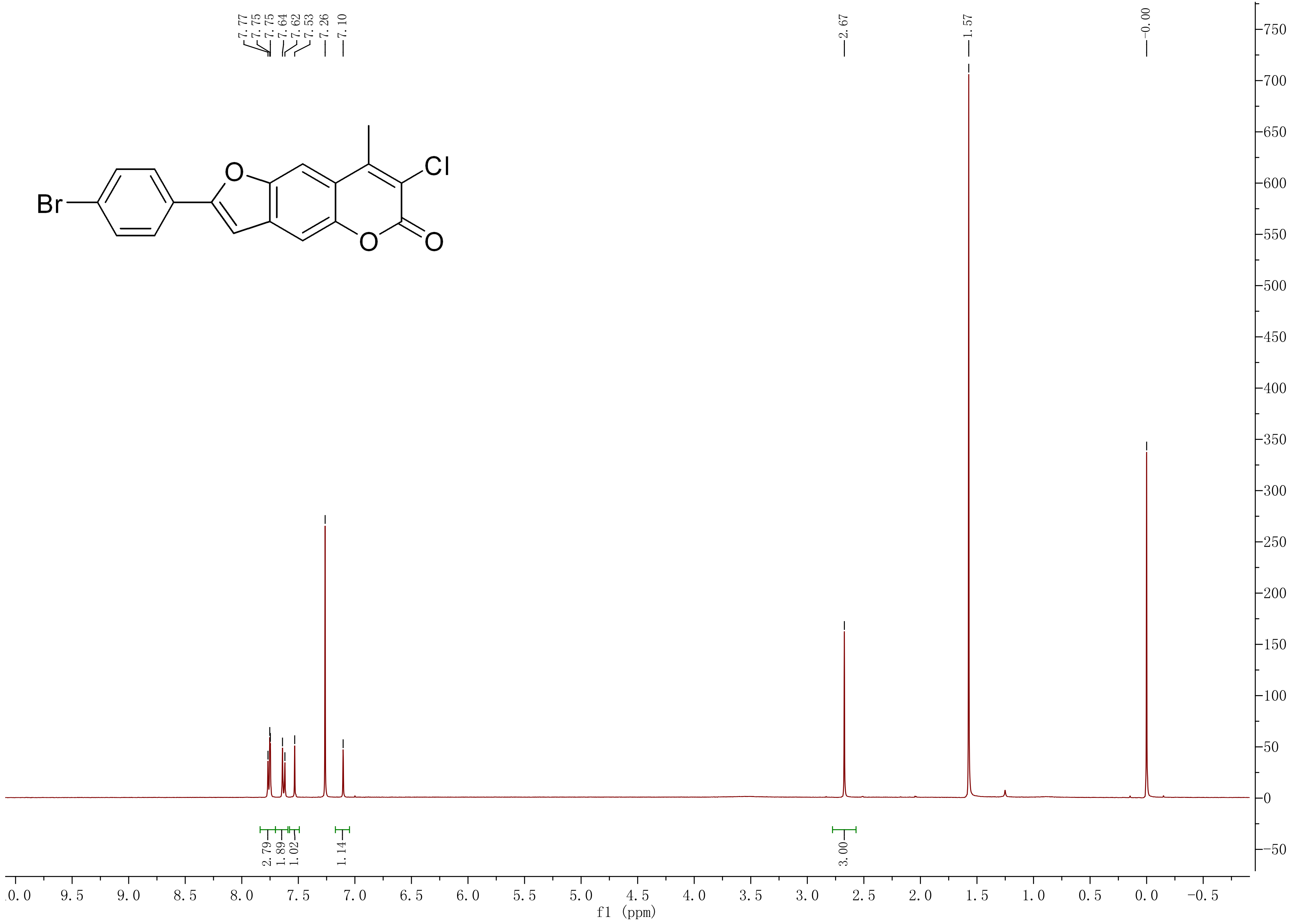

7.77
7.75
7.75
7.64
7.62
7.53
7.26
7.10
2.67
1.57
-0.00
Br
O
Cl
O
O
2.79
1.89
1.02
1.14
3.00
f1 (ppm)

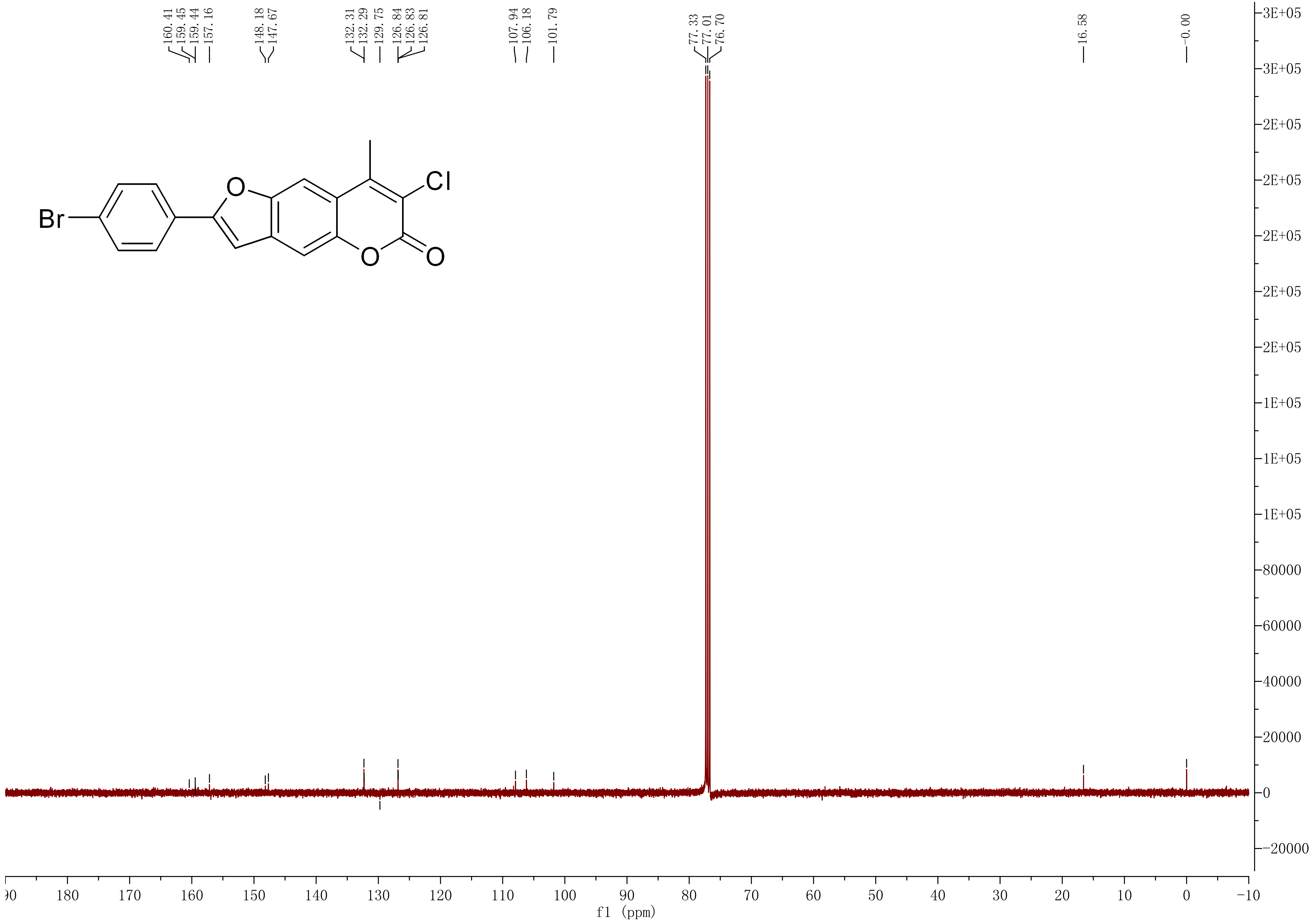
160.41
159.45
159.44
157.16
148.18
147.67
132.31
132.29
129.75
126.84
126.83
126.81
107.94
106.18
101.79
77.33
77.01
76.70
16.58
-0.00
Br
O
Cl
O
O
f1 (ppm)

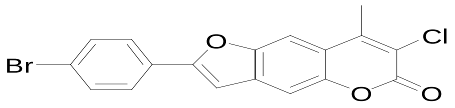
Br
O
Cl
O
O

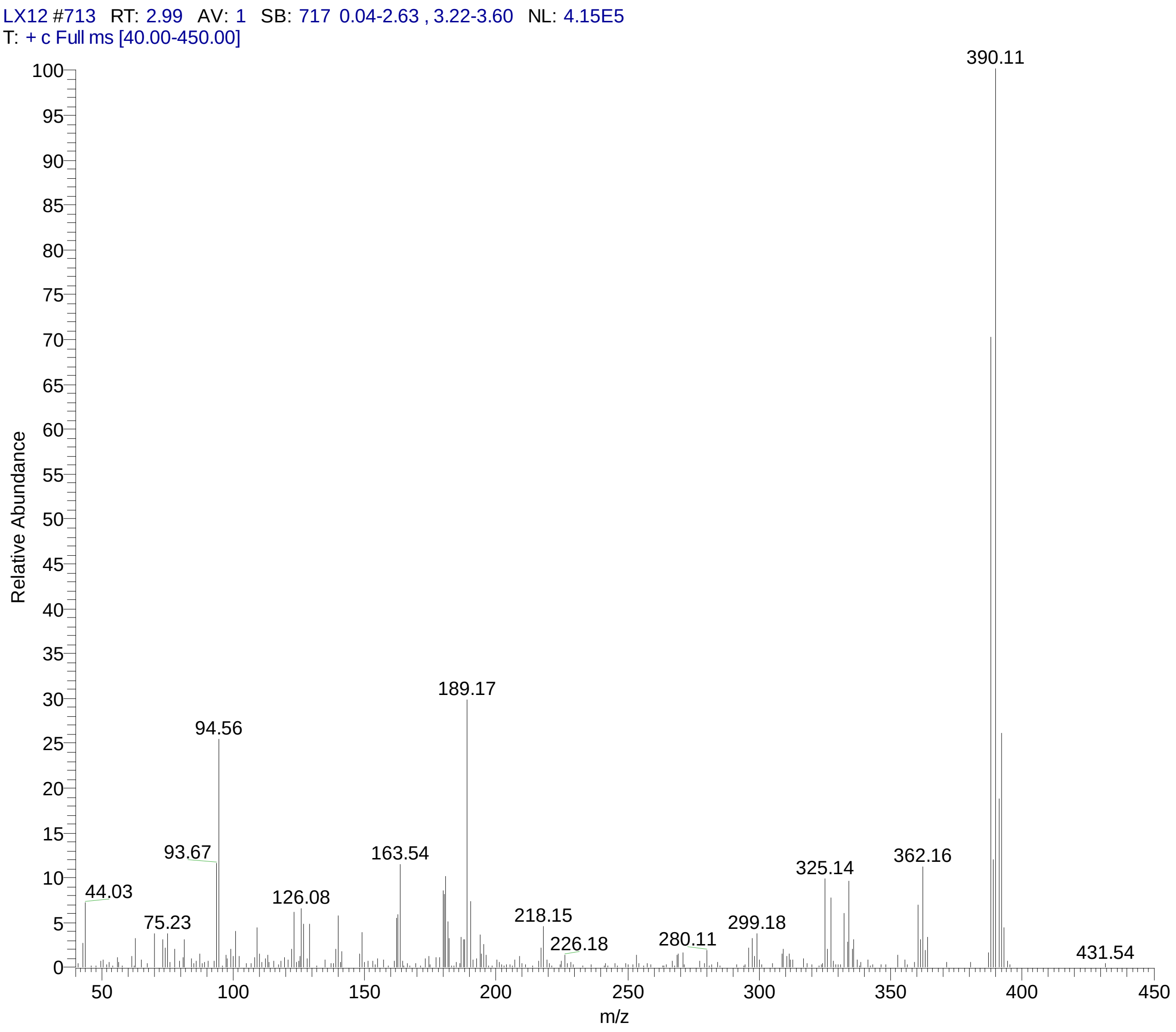
LX12 #713 RT: 2.99 AV: 1 SB: 717 0.04-2.63 , 3.22-3.60 NL: 4.15E5
T: + c Full ms [40.00-450.00]
Relative Abundance
m/z
390.11
189.17
94.56
93.67
163.54
362.16
325.14
44.03
126.08
75.23
218.15
299.18
226.18
280.11
431.54

MS of I16

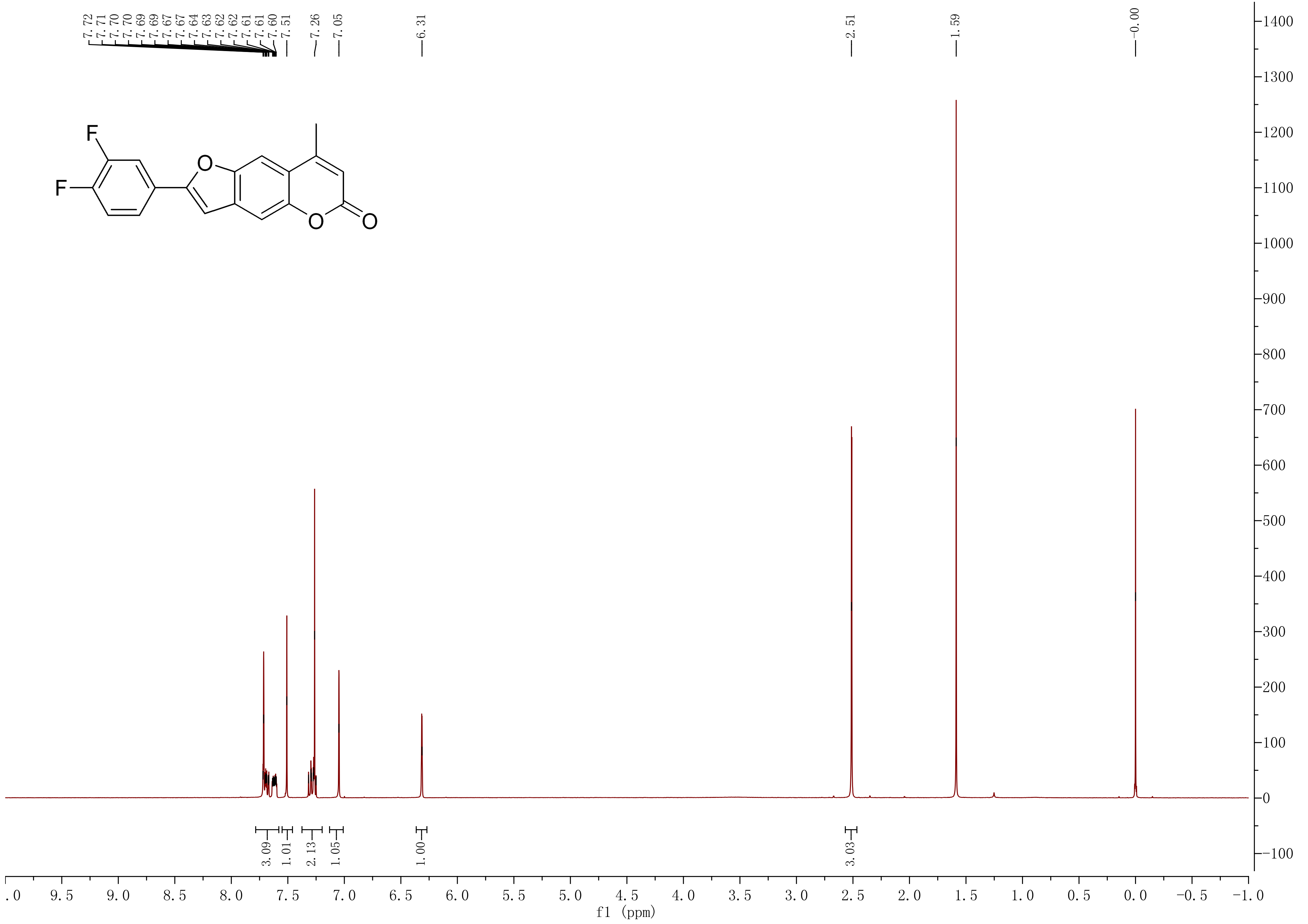

7.72
7.71
7.70
7.70
7.69
7.69
7.67
7.67
7.64
7.63
7.62
7.62
7.61
7.61
7.60
7.51
7.26
7.05
6.31
2.51
1.59
-0.00
F
F
O
O
O
3.09
1.01
2.13
1.05
1.00
3.03
f1 (ppm)

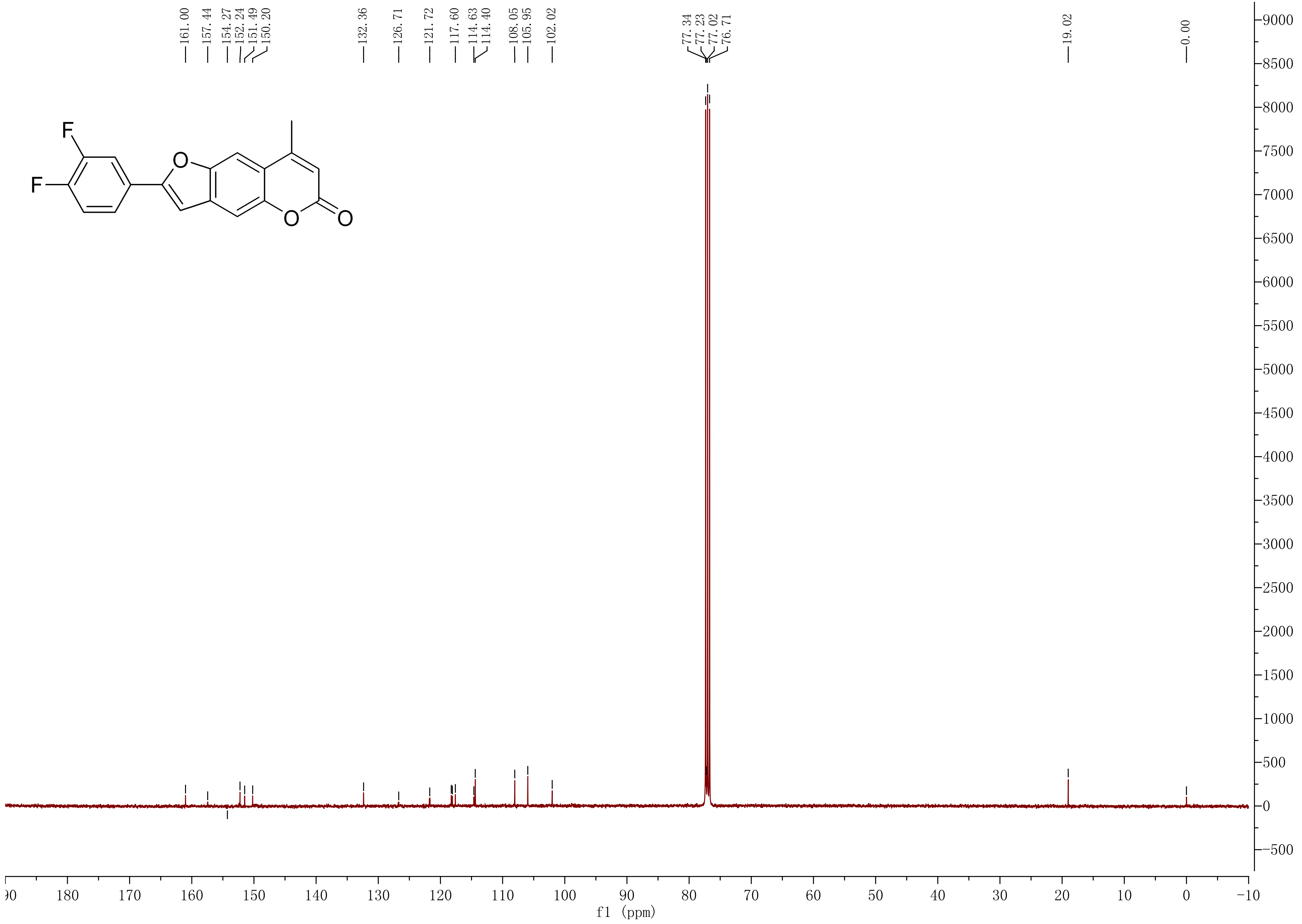

161.00
157.44
154.27
152.24
151.49
150.20
132.36
126.71
121.72
117.60
114.63
114.40
108.05
105.95
102.02
77.34
77.23
77.02
76.71
19.02
0.00
F
F
O
O
O
f1 (ppm)

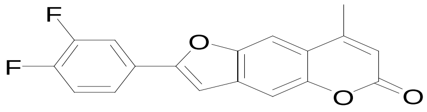

LX91 #652 RT: 2.74 AV: 1 SB: 642 0.04-2.27 , 2.88-3.30 NL: 4.00E5
T: + c Full ms [40.00-450.00]

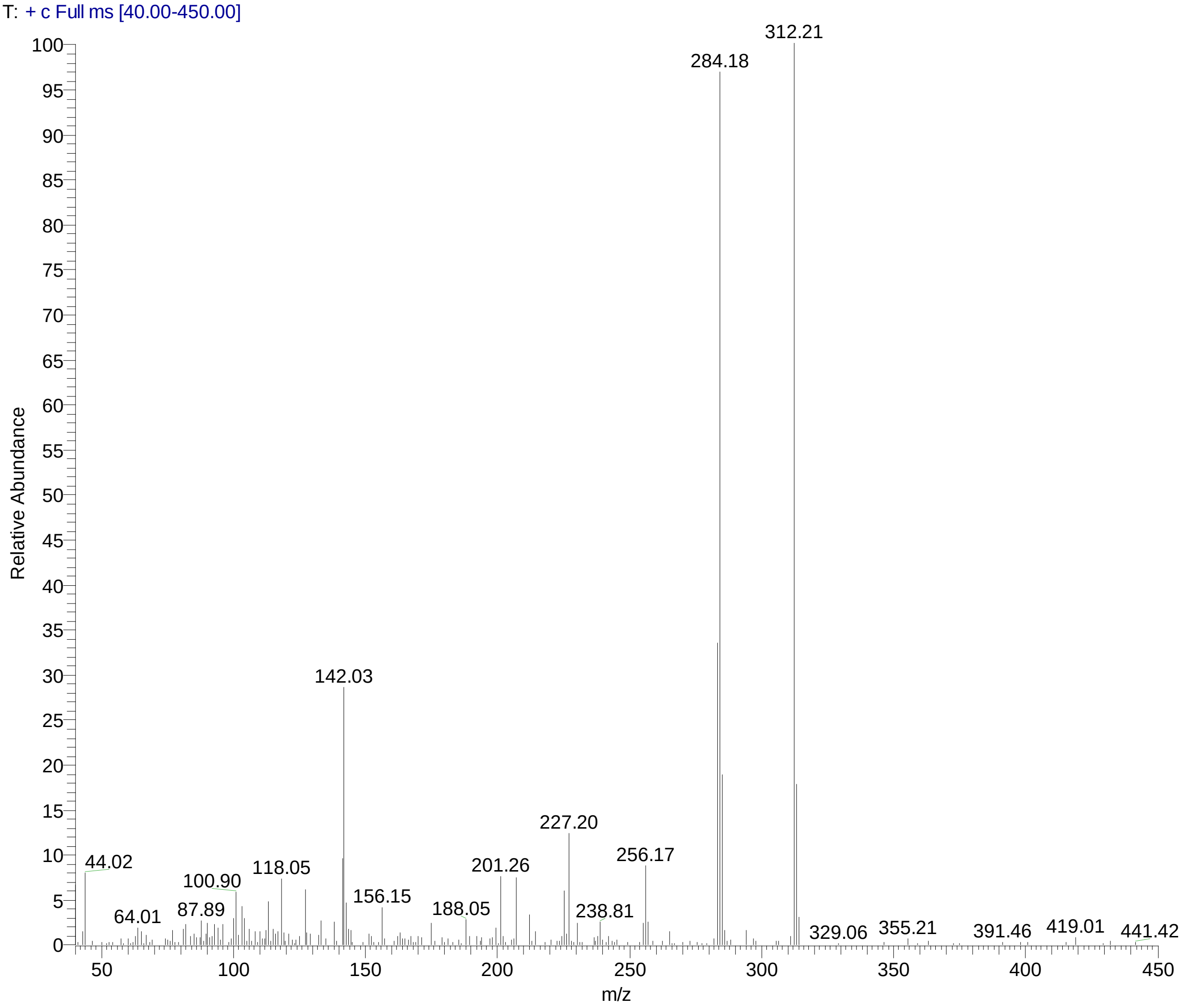

MS of I17

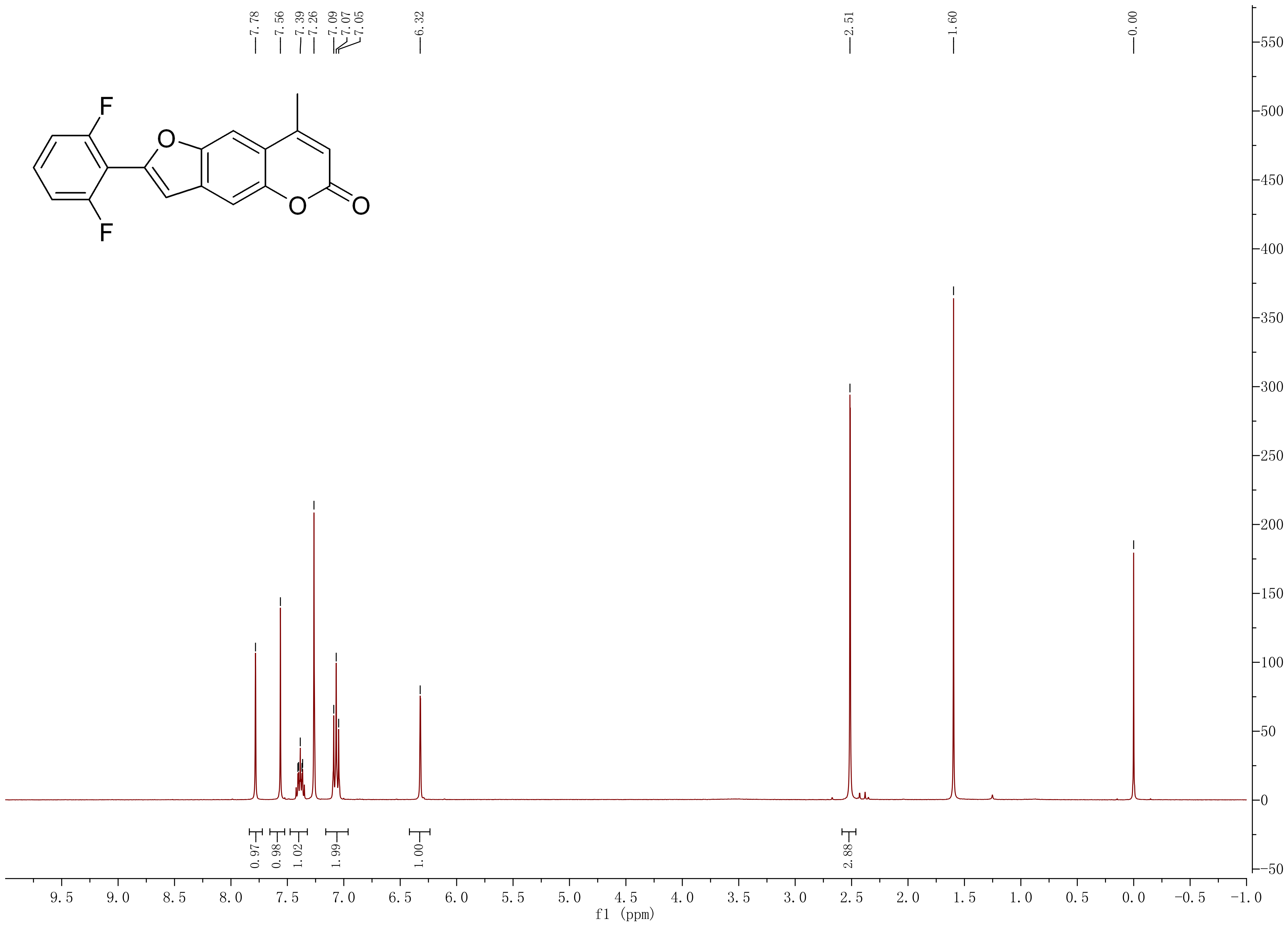

7.78
7.56
7.39
7.26
7.09
7.07
7.05
6.32
2.51
1.60
0.00
F
O
O
O
F
0.97
0.98
1.02
1.99
1.00
2.88
9.5
9.0
8.5
8.0
7.5
7.0
6.5
6.0
5.5
5.0
4.5
4.0
3.5
3.0
2.5
2.0
1.5
1.0
0.5
0.0
-0.5
-1.0
f1 (ppm)
550
500
450
400
350
300
250
200
150
100
50
0
-50

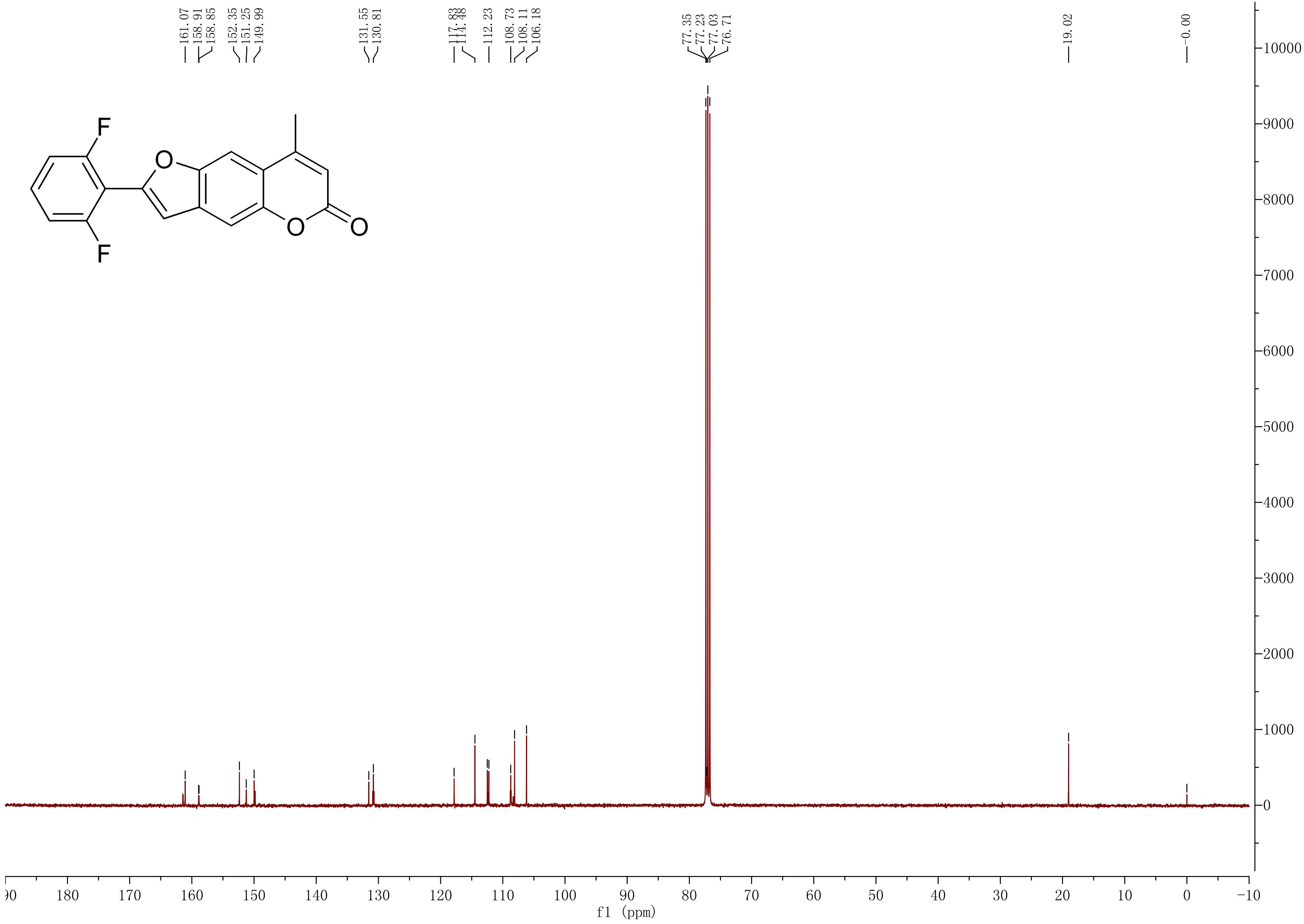
161.07
158.91
158.85
152.35
151.25
149.99
131.55
130.81
117.83
114.48
112.23
108.73
108.11
106.18
77.35
77.23
77.03
76.71
19.02
-0.00
F
F
O
O
O
10000
9000
8000
7000
6000
5000
4000
3000
2000
1000
0
180
170
160
150
140
130
120
110
100
90
80
70
60
50
40
30
20
10
0
-10
f1 (ppm)

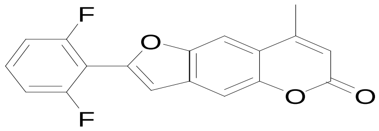
F
O
O
O
F

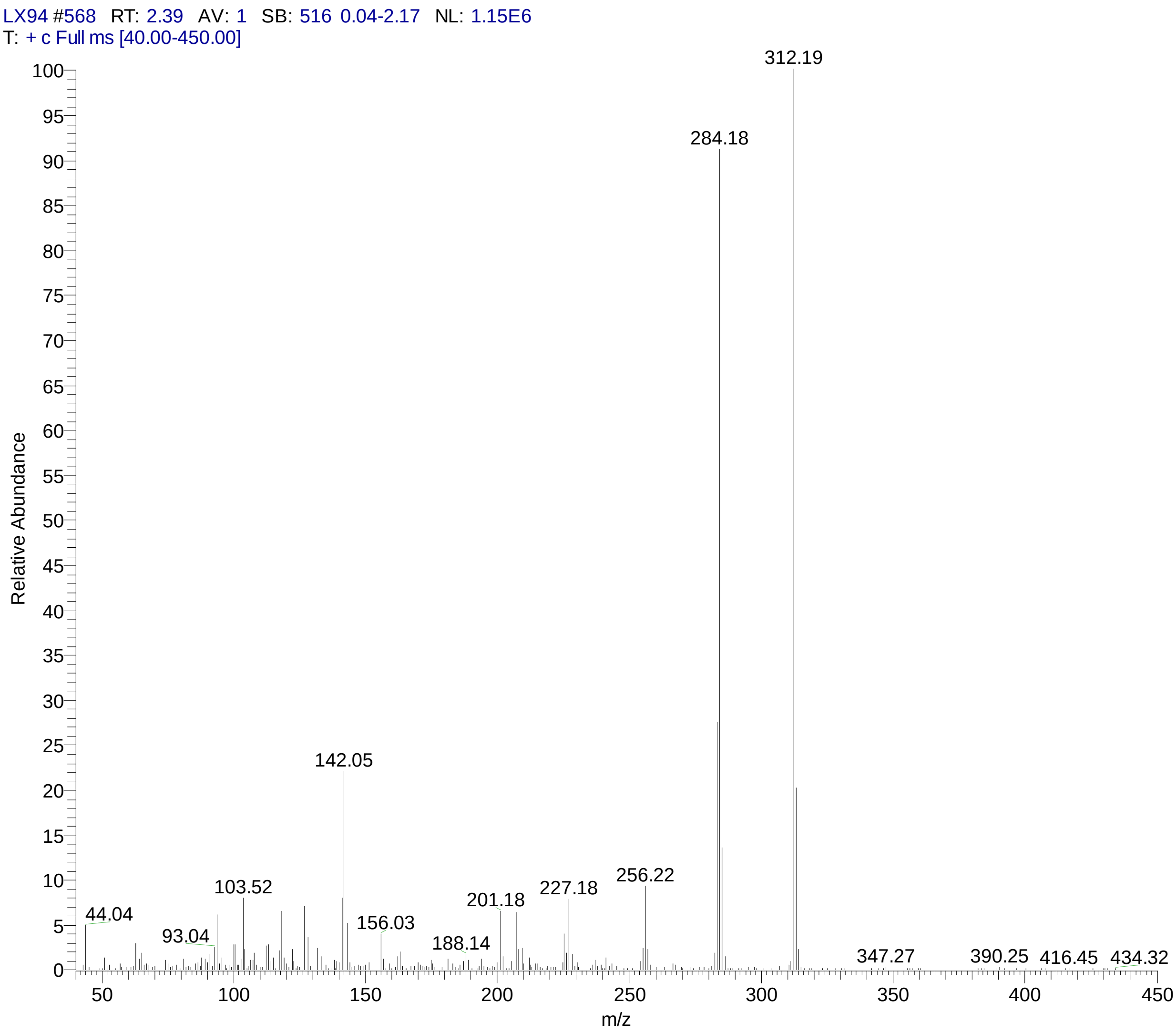
LX94 #568 RT: 2.39 AV: 1 SB: 516 0.04-2.17 NL: 1.15E6
T: + c Full ms [40.00-450.00]
312.19
284.18
142.05
103.52
44.04
93.04
156.03
188.14
201.18
227.18
256.22
347.27
390.25
416.45
434.32
Relative Abundance
m/z

MS of I18

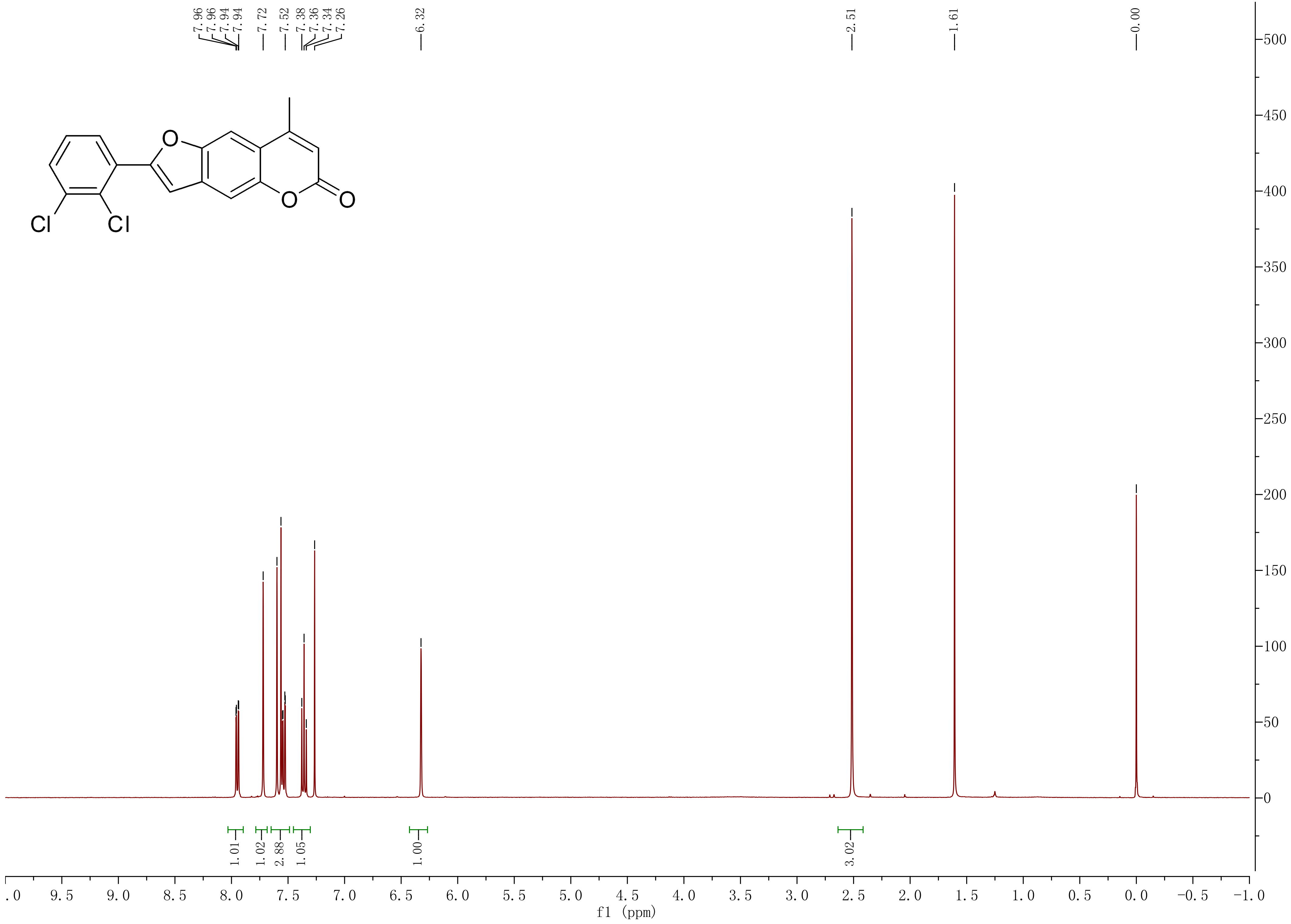

7.96
7.96
7.94
7.94
7.72
7.52
7.38
7.36
7.34
7.26
6.32
2.51
1.61
0.00
O
O
O
Cl
Cl
1.01
1.02
2.88
1.05
1.00
3.02
500
450
400
350
300
250
200
150
100
50
0
.0
9.5
9.0
8.5
8.0
7.5
7.0
6.5
6.0
5.5
5.0
4.5
4.0
3.5
3.0
2.5
2.0
1.5
1.0
0.5
0.0
-0.5
-1.0
f1 (ppm)

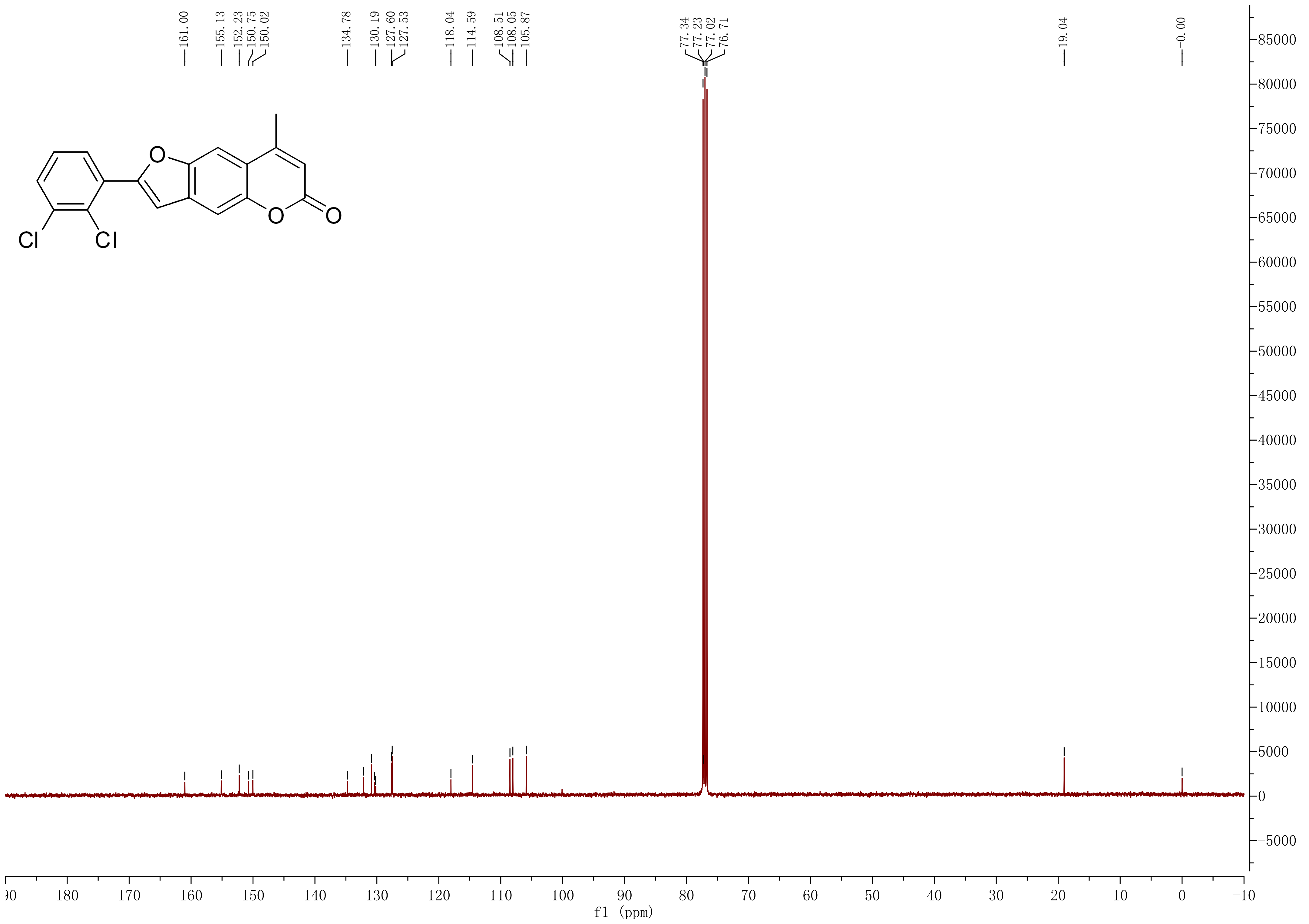
161.00
155.13
152.23
150.75
150.02
134.78
130.19
127.60
127.53
118.04
114.59
108.51
108.05
105.87
77.34
77.23
77.02
76.71
19.04
-0.00
O
O
O
Cl
Cl
f1 (ppm)

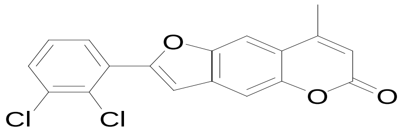
O
O
O
Cl
Cl

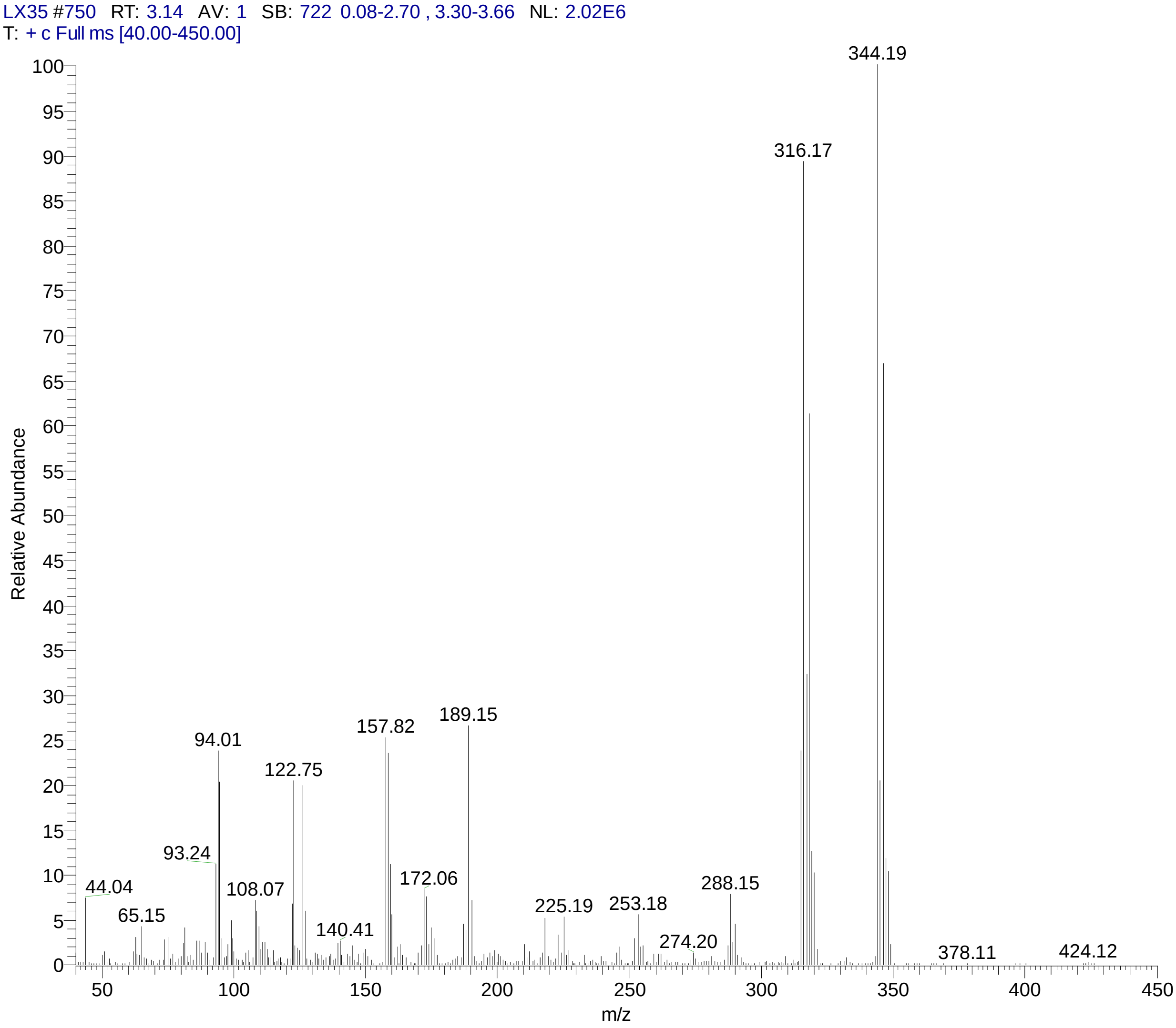
LX35 #750 RT: 3.14 AV: 1 SB: 722 0.08-2.70 , 3.30-3.66 NL: 2.02E6
T: + c Full ms [40.00-450.00]
Relative Abundance
m/z
344.19
316.17
189.15
157.82
94.01
122.75
93.24
172.06
44.04
108.07
65.15
140.41
225.19
253.18
288.15
274.20
378.11
424.12

MS of I19

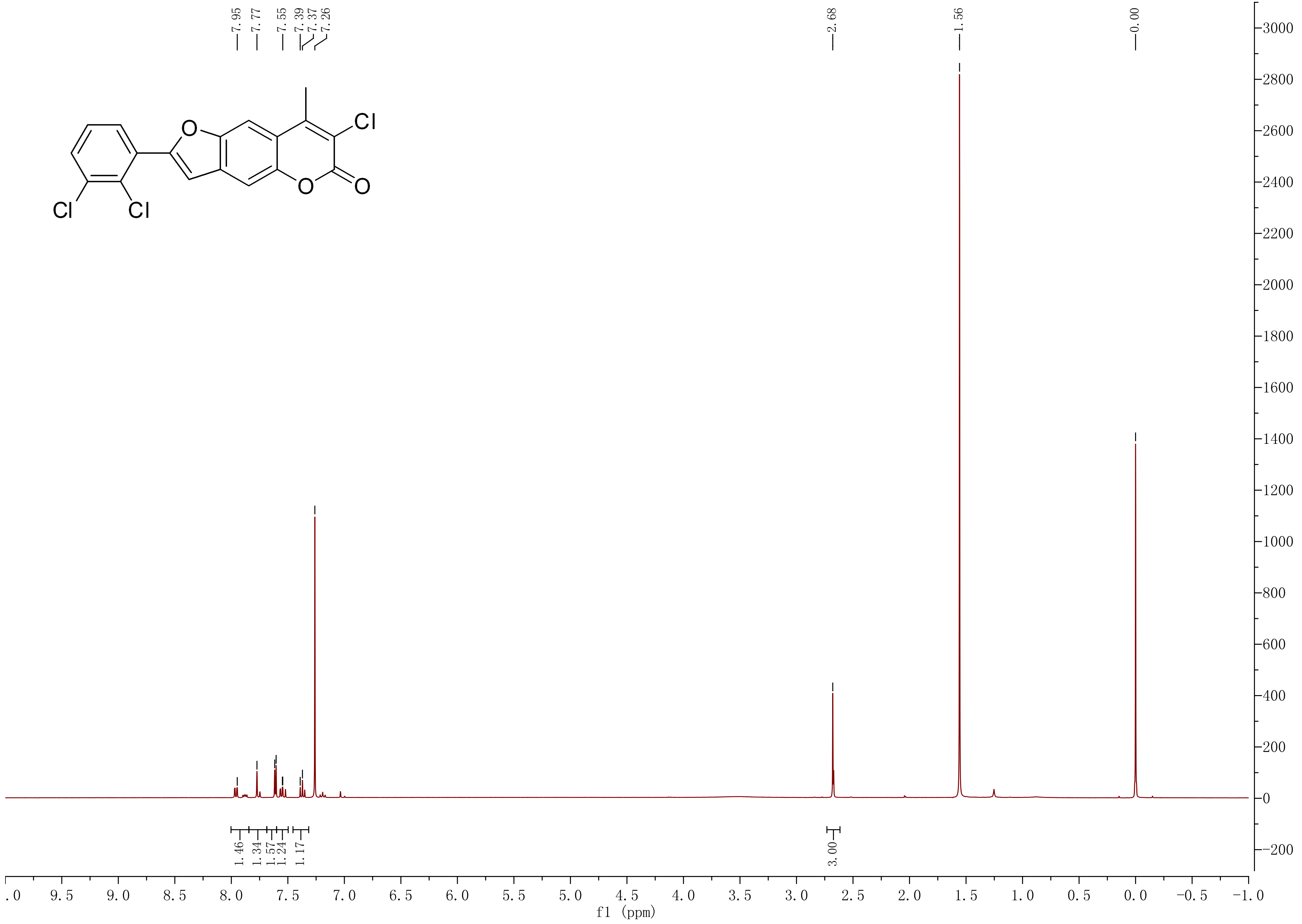

7.95
7.77
7.55
7.39
7.37
7.26
2.68
1.56
0.00
Cl
Cl
Cl
O
O
O
1.46
1.34
1.57
1.24
1.17
3.00
f1 (ppm)

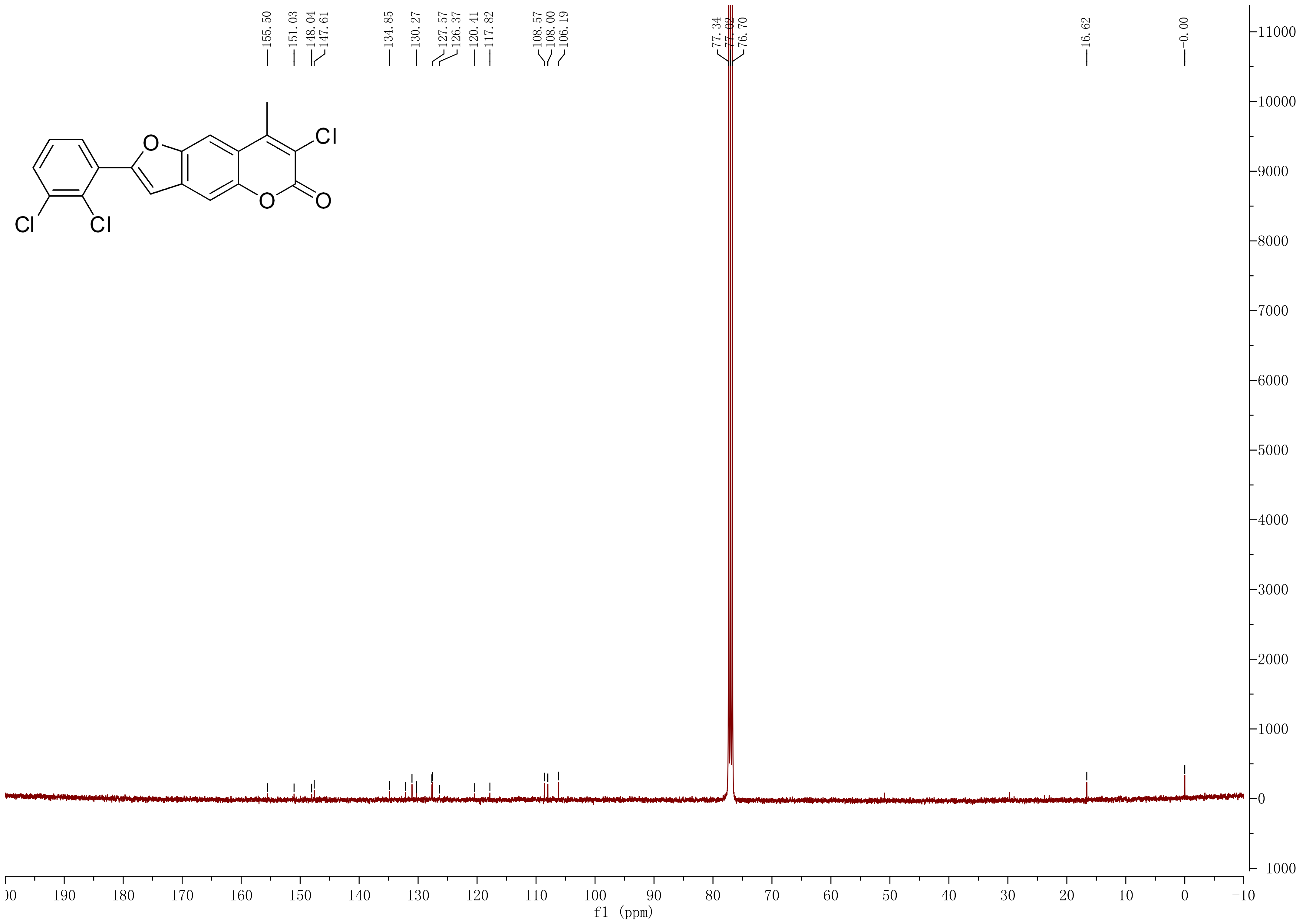

155.50
151.03
148.04
147.61
134.85
130.27
127.57
126.37
120.41
117.82
108.57
108.00
106.19
77.34
77.02
76.70
16.62
-0.00
Cl
Cl
Cl
O
O
O
f1 (ppm)

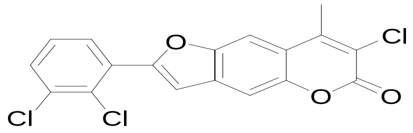
O
Cl
O
O
Cl
Cl

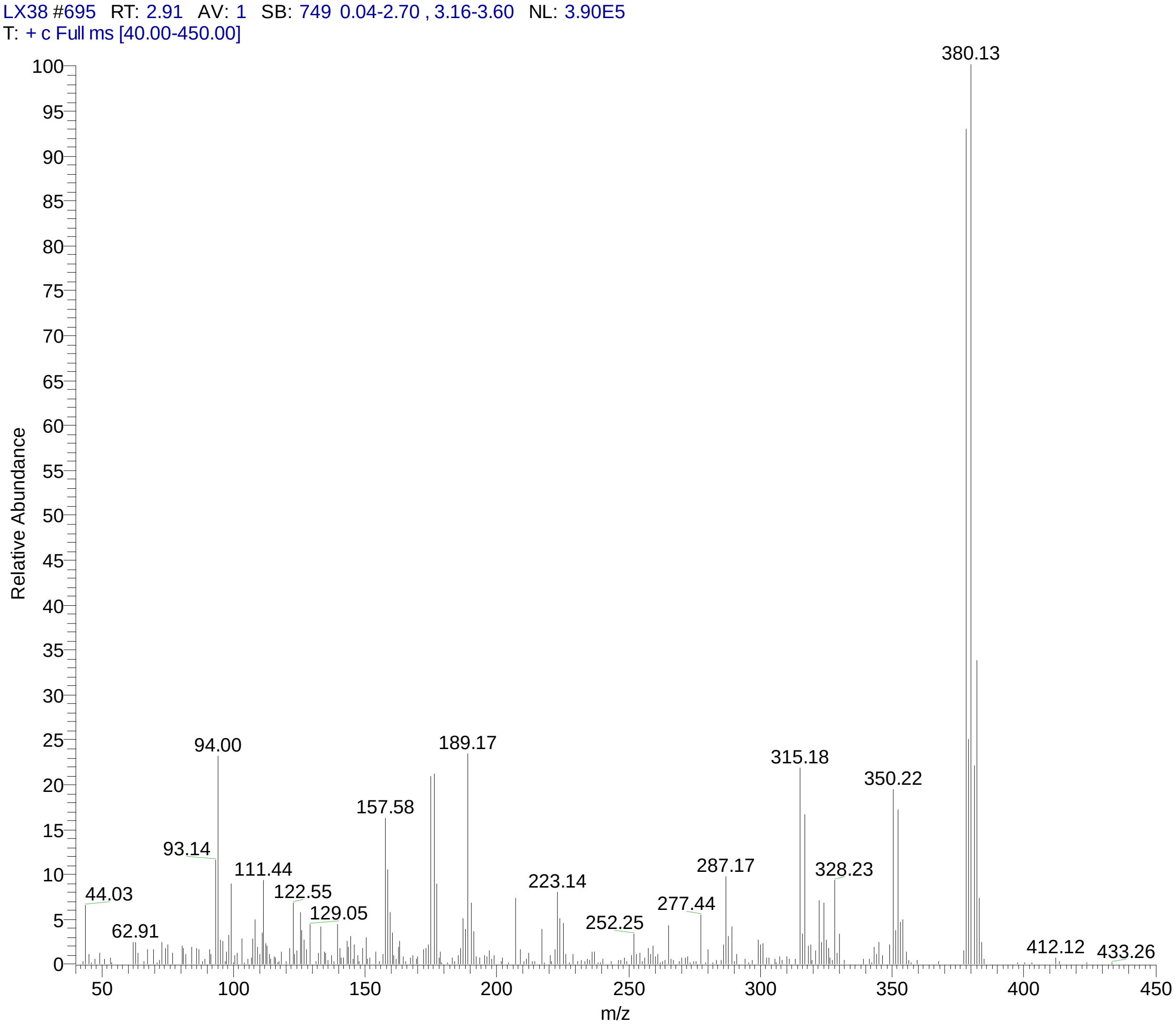
LX38 #695 RT: 2.91 AV: 1 SB: 749 0.04-2.70 , 3.16-3.60 NL: 3.90E5
T: + c Full ms [40.00-450.00]
380.13
94.00
189.17
315.18
350.22
157.58
93.14
111.44
287.17
328.23
223.14
44.03
122.55
277.44
129.05
252.25
62.91
412.12
433.26
Relative Abundance
m/z

MS of I20

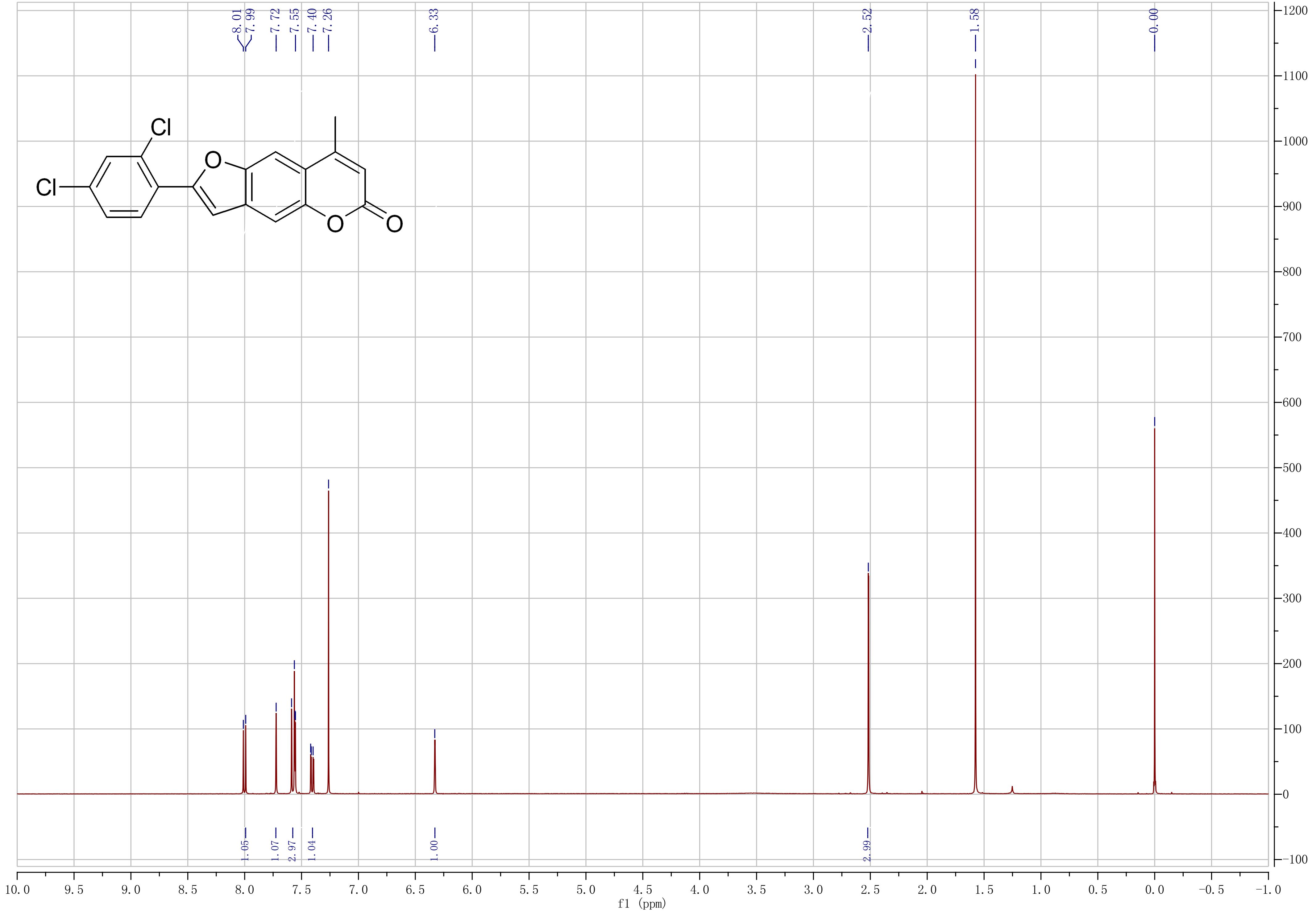

8.01
7.99
7.72
7.55
7.40
7.26
6.33
2.52
1.58
0.00
Cl
Cl
O
O
O
1.05
1.07
2.97
1.04
1.00
2.99
10.0 9.5 9.0 8.5 8.0 7.5 7.0 6.5 6.0 5.5 5.0 4.5 4.0 3.5 3.0 2.5 2.0 1.5 1.0 0.5 0.0 -0.5 -1.0
f1 (ppm)
1200 1100 1000 900 800 700 600 500 400 300 200 100 0 -100

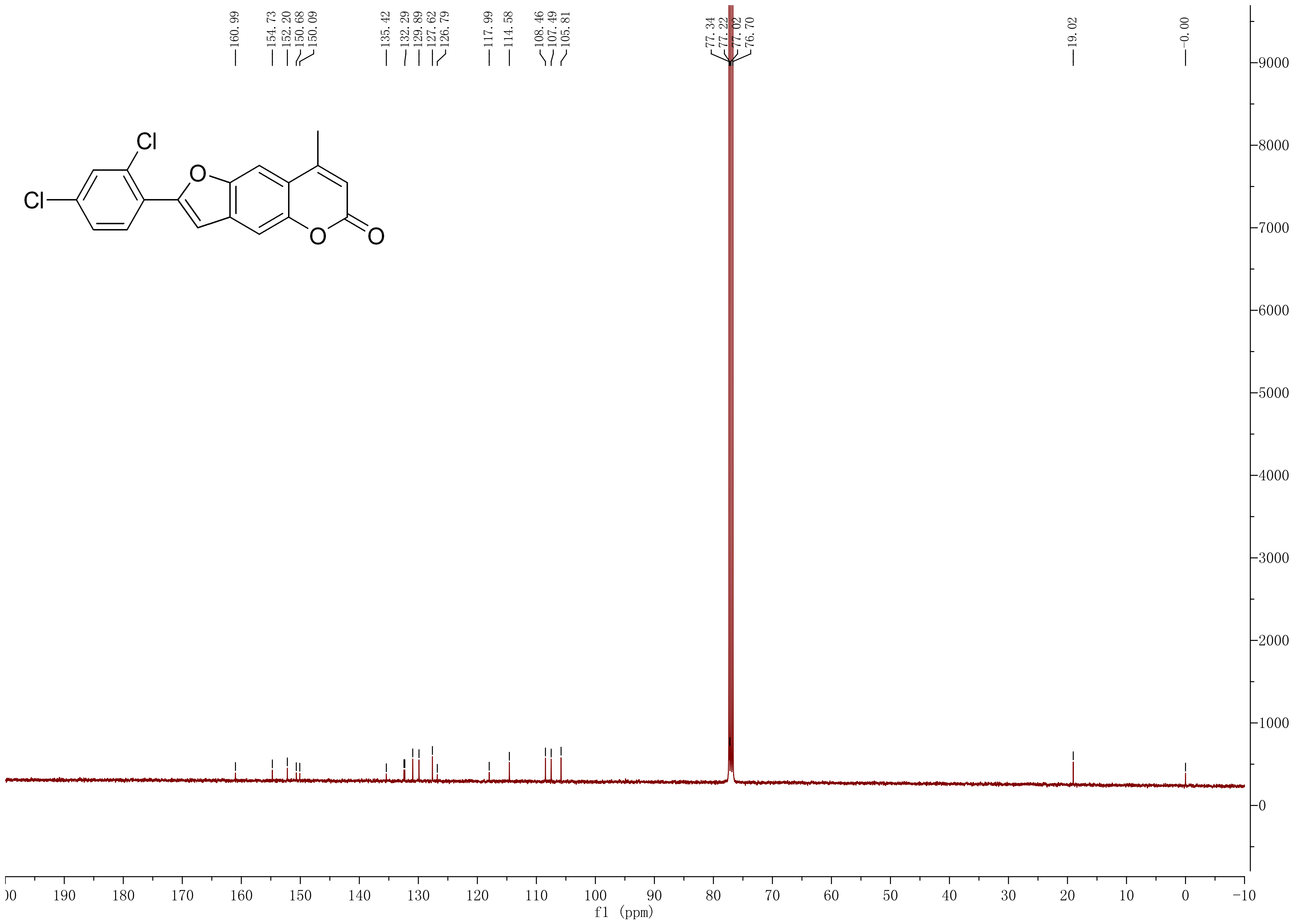

160.99
154.73
152.20
150.68
150.09
135.42
132.29
129.89
127.62
126.79
117.99
114.58
108.46
107.49
105.81
77.34
77.22
77.02
76.70
19.02
-0.00
Cl
Cl
O
O
O
f1 (ppm)

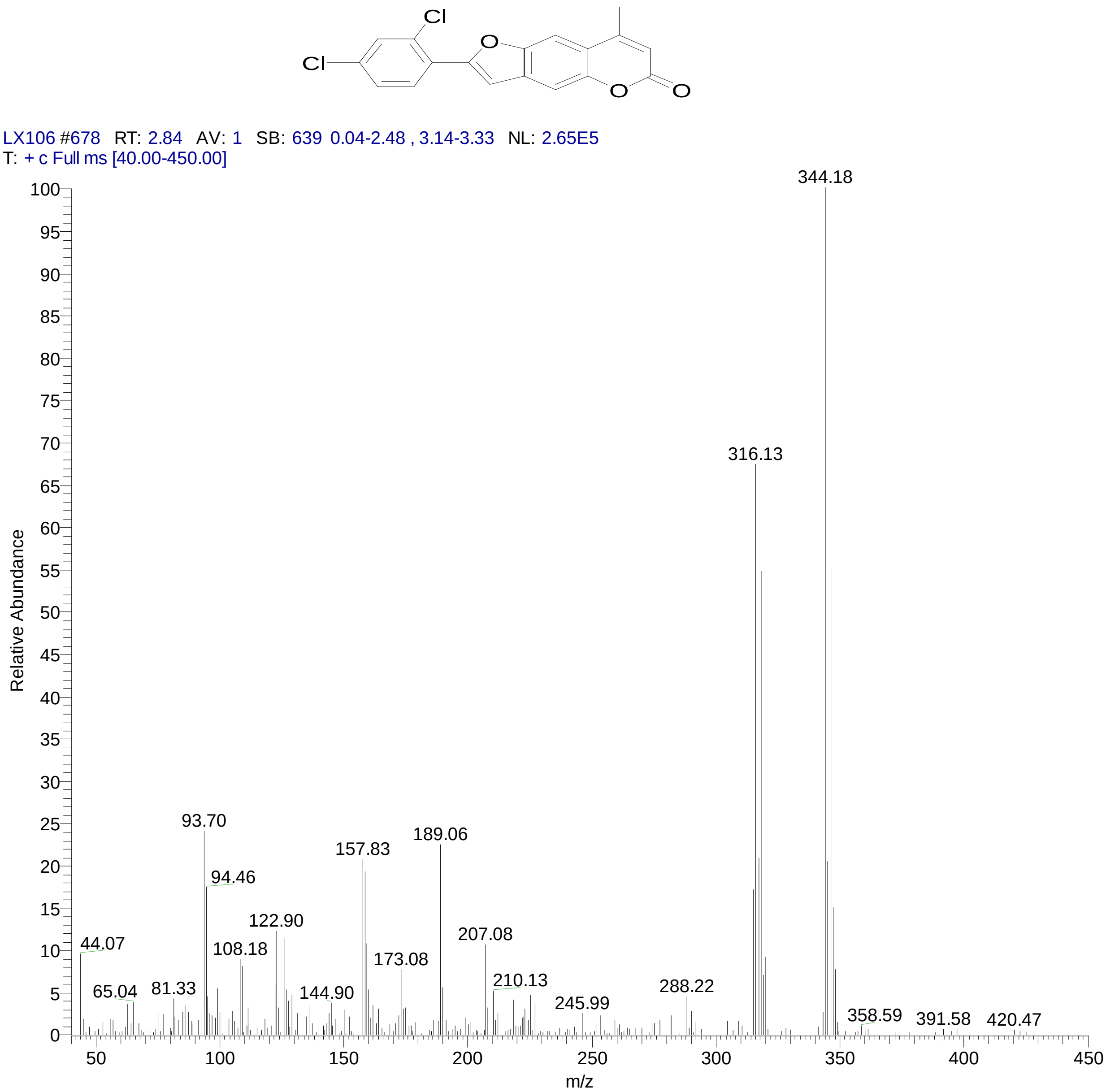
Cl
Cl
O
O
O
LX106 #678 RT: 2.84 AV: 1 SB: 639 0.04-2.48 , 3.14-3.33 NL: 2.65E5
T: + c Full ms [40.00-450.00]
344.18
316.13
93.70
189.06
157.83
94.46
122.90
207.08
44.07
108.18
173.08
210.13
65.04
81.33
144.90
245.99
288.22
358.59
391.58
420.47
Relative Abundance
m/z

MS of I21

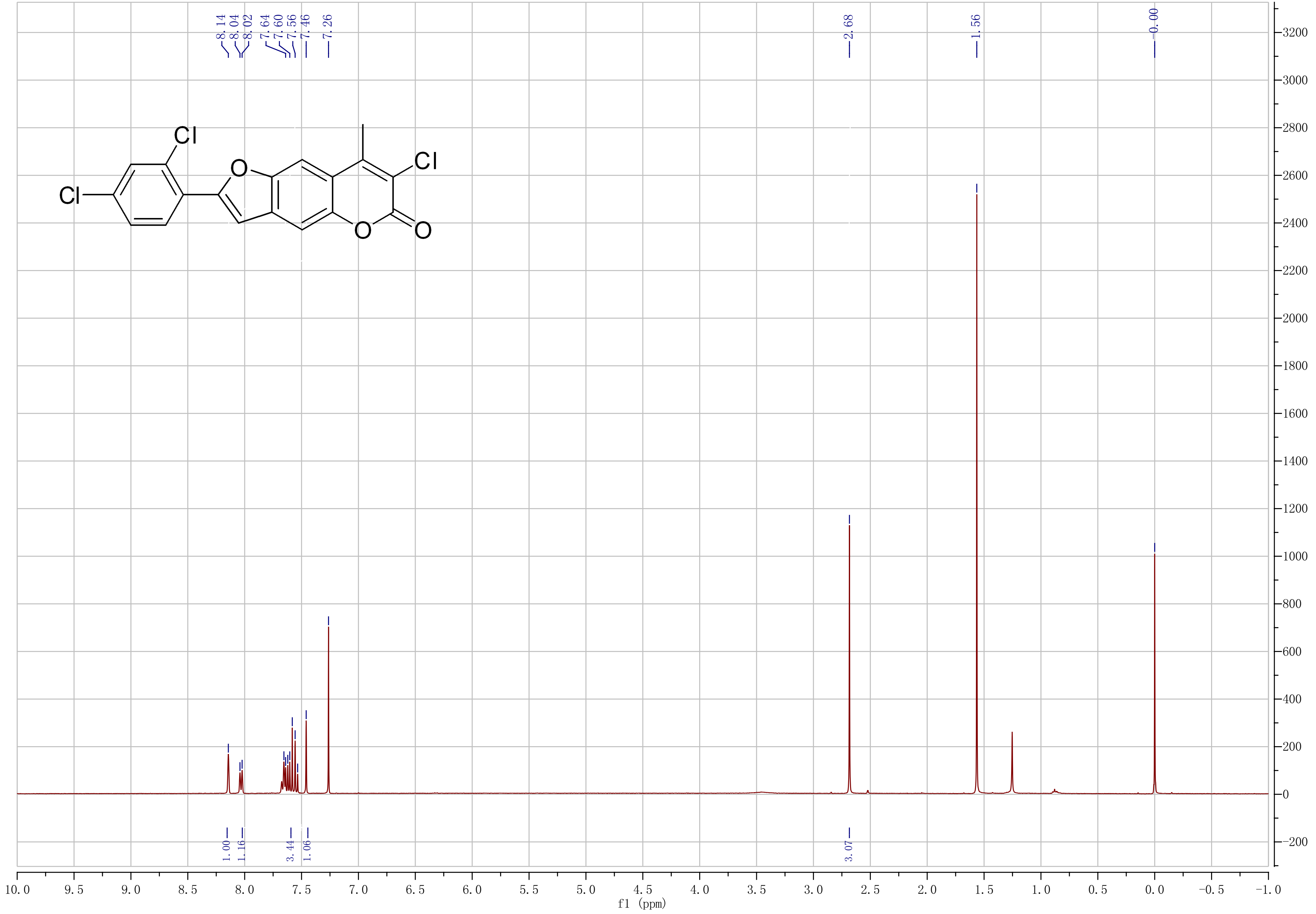

8.14
8.04
8.02
7.64
7.60
7.56
7.46
7.26
2.68
1.56
0.00
Cl
Cl
Cl
O
O
O
1.00
1.16
3.44
1.06
3.07
f1 (ppm)

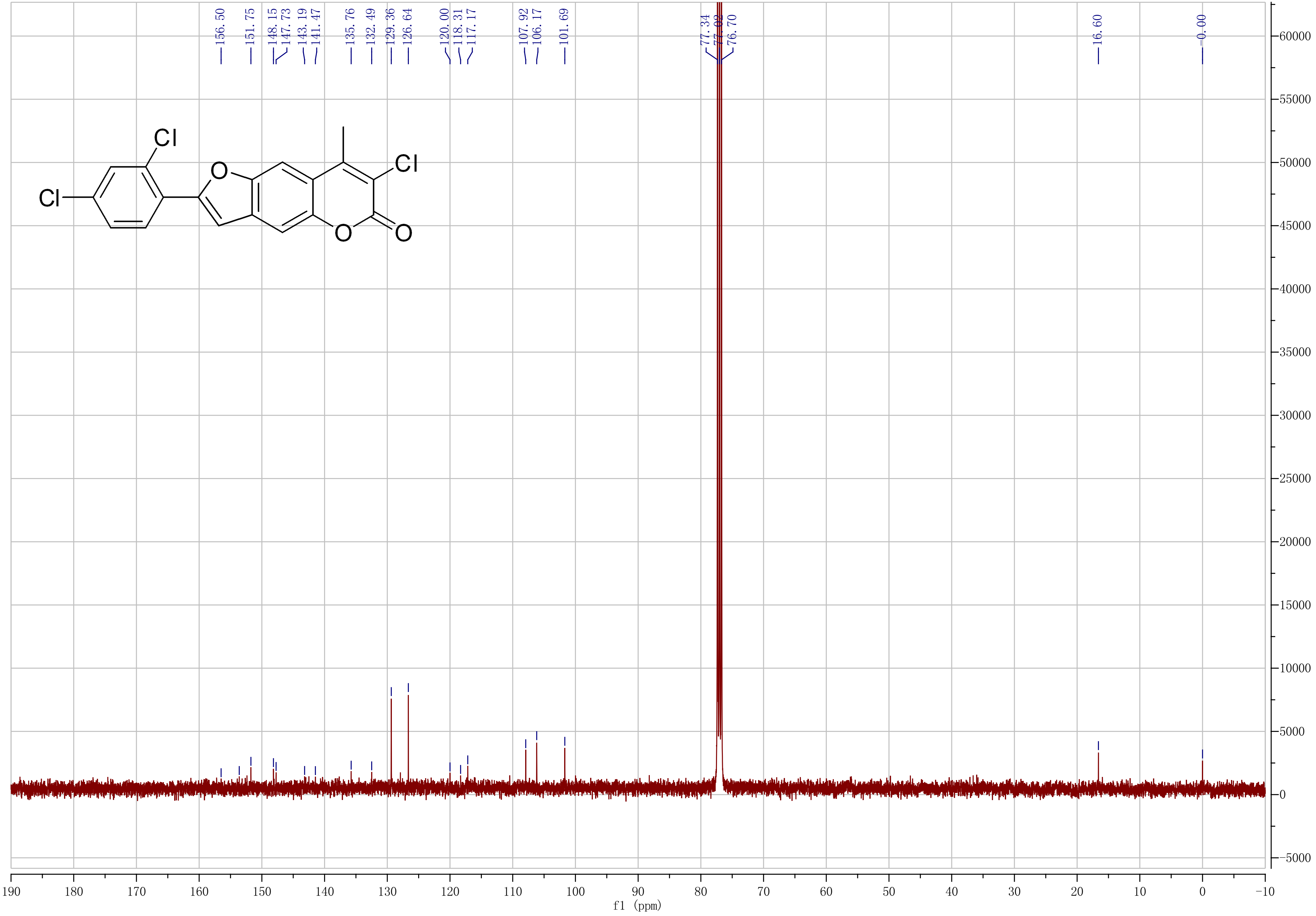
156.50
151.75
148.15
147.73
143.19
141.47
135.76
132.49
129.36
126.64
120.00
118.31
117.17
107.92
106.17
101.69
77.34
77.02
76.70
16.60
0.00
Cl
Cl
Cl
O
O
O
f1 (ppm)

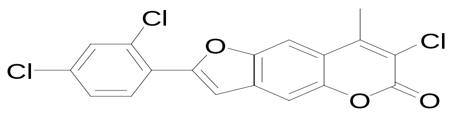

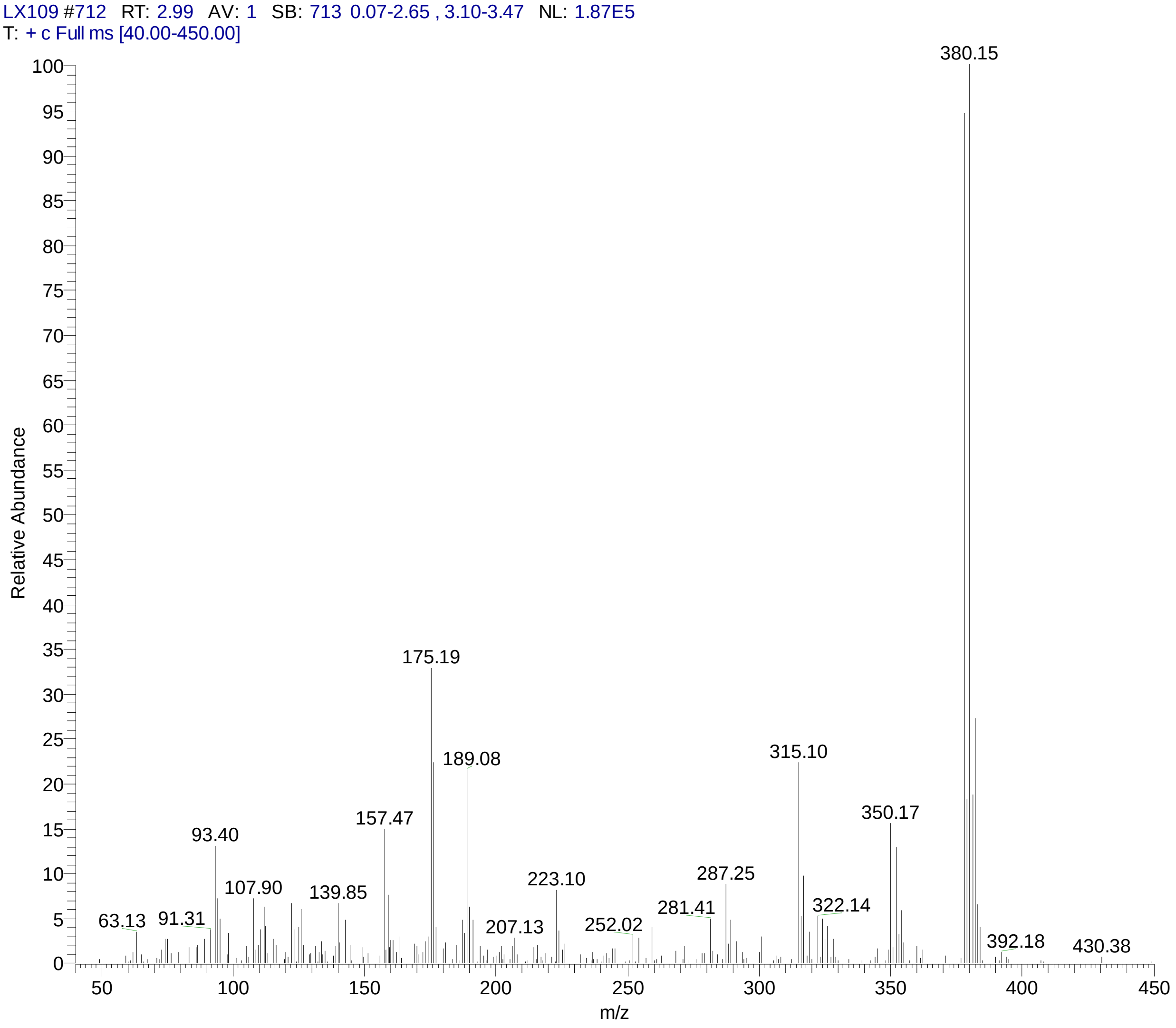

LX109 #712 RT: 2.99 AV: 1 SB: 713 0.07-2.65 , 3.10-3.47 NL: 1.87E5
T: + c Full ms [40.00-450.00]
Relative Abundance
m/z
380.15
175.19
189.08
315.10
350.17
157.47
93.40
287.25
223.10
107.90
139.85
322.14
281.41
63.13
91.31
207.13
252.02
392.18
430.38

MS of I22

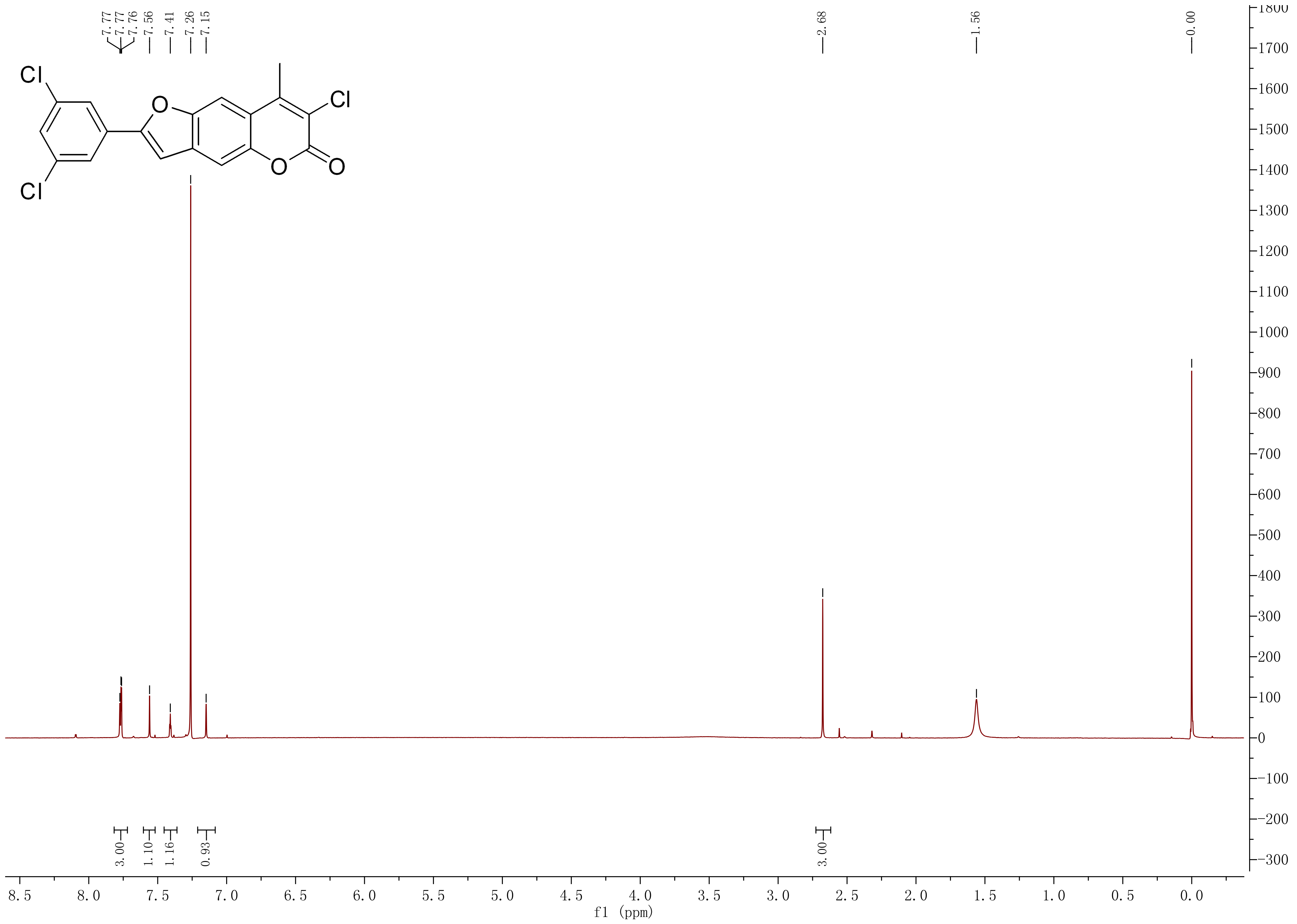

7.77
7.77
7.76
7.56
7.41
7.26
7.15
2.68
1.56
0.00
Cl
O
Cl
Cl
O
O
3.00
1.10
1.16
0.93
3.00
f1 (ppm)

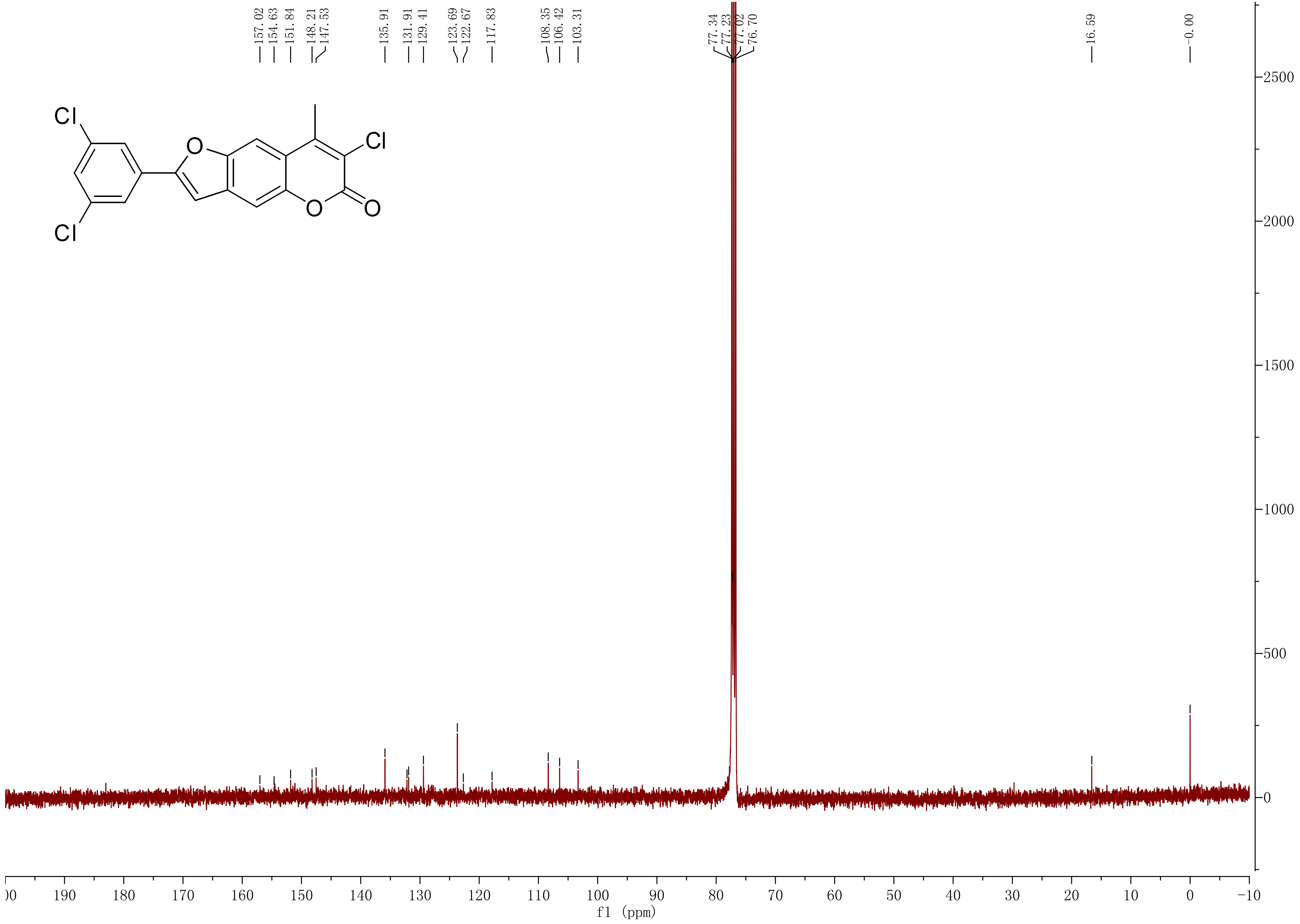
157.02
154.63
151.84
148.21
147.53
135.91
131.91
129.41
123.69
122.67
117.83
108.35
106.42
103.31
77.34
77.23
77.02
76.70
16.59
-0.00
Cl
Cl
O
Cl
O
O
2500
2000
1500
1000
500
0
190
180
170
160
150
140
130
120
110
100
90
80
70
60
50
40
30
20
10
0
-10
f1 (ppm)

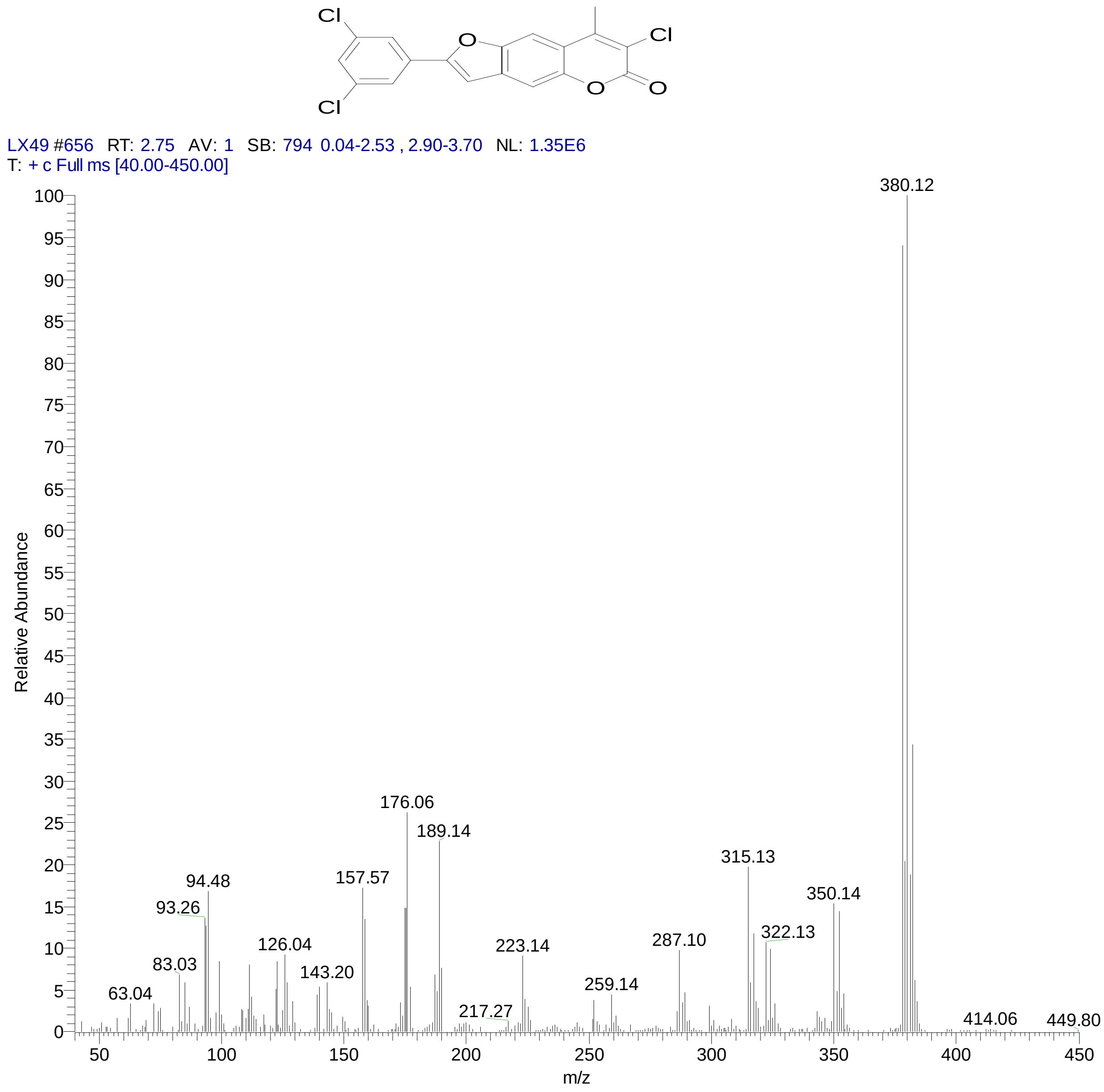

Cl
O
Cl
Cl
O
O
LX49 #656 RT: 2.75 AV: 1 SB: 794 0.04-2.53 , 2.90-3.70 NL: 1.35E6
T: + c Full ms [40.00-450.00]
Relative Abundance
m/z
380.12
176.06
189.14
315.13
350.14
322.13
287.10
259.14
223.14
217.27
157.57
143.20
126.04
94.48
93.26
83.03
63.04
414.06
449.80

MS of I23

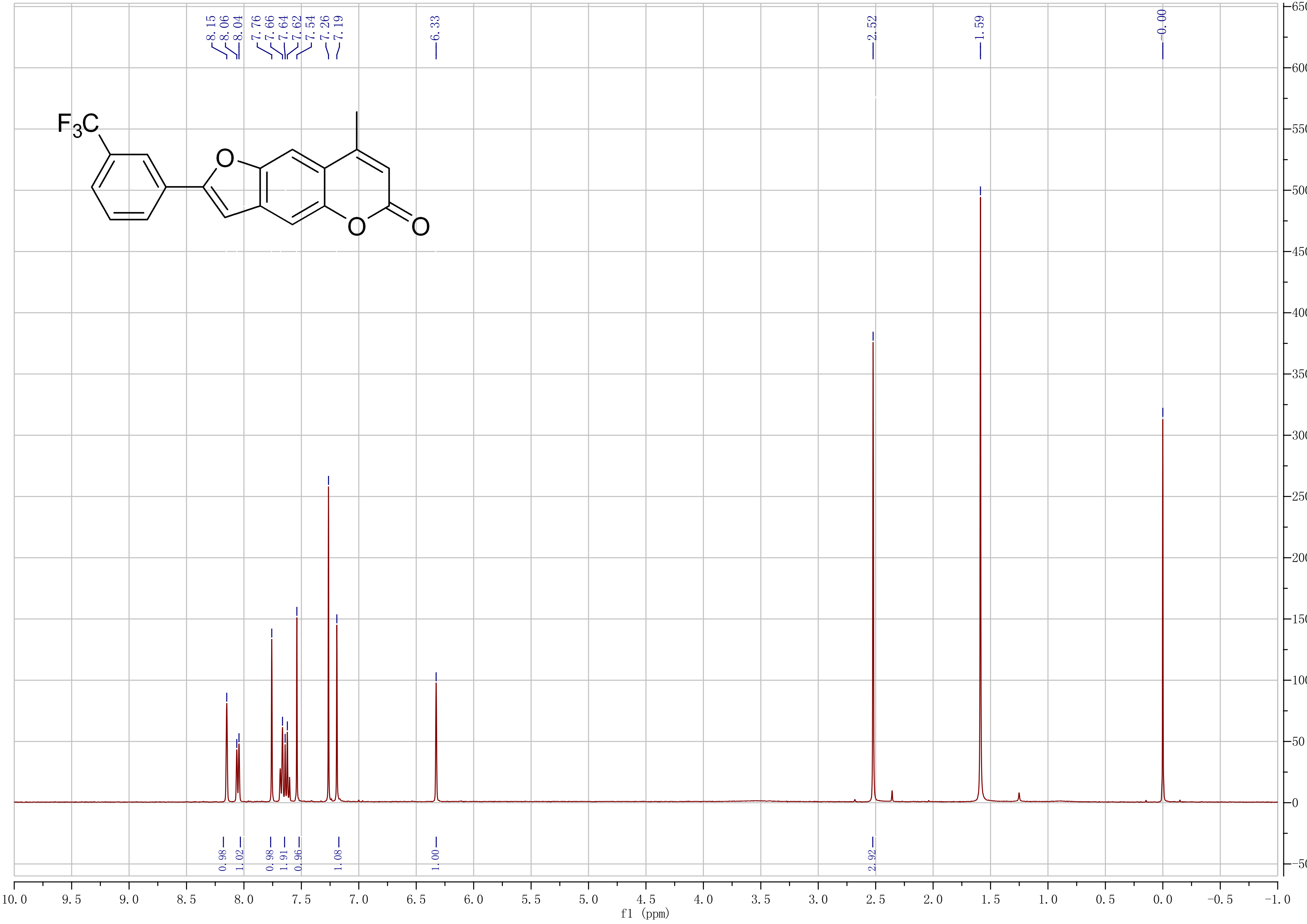

8.15
8.06
8.04
7.76
7.66
7.64
7.62
7.54
7.26
7.19
6.33
2.52
1.59
-0.00
F3C
O
O
O
0.98
1.02
0.98
1.91
0.96
1.08
1.00
2.92
10.0
9.5
9.0
8.5
8.0
7.5
7.0
6.5
6.0
5.5
5.0
4.5
4.0
3.5
3.0
2.5
2.0
1.5
1.0
0.5
0.0
-0.5
-1.0
f1 (ppm)
650
600
550
500
450
400
350
300
250
200
150
100
50
0
-50

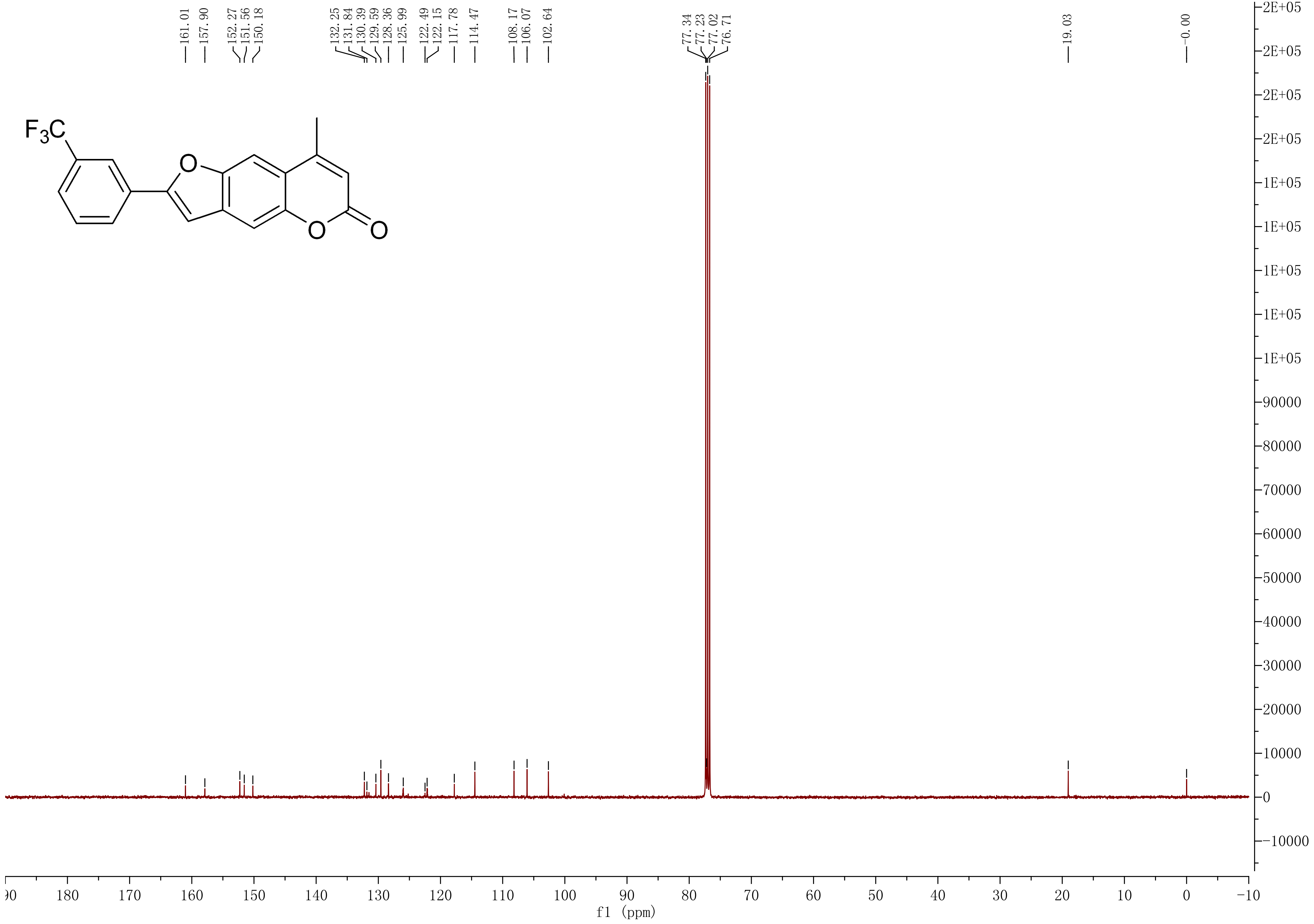
161.01
157.90
152.27
151.56
150.18
132.25
131.84
130.39
129.59
128.36
125.99
122.49
122.15
117.78
114.47
108.17
106.07
102.64
77.34
77.23
77.02
76.71
19.03
-0.00
F3C
O
O
O
f1 (ppm)
2E+05
1E+05
90000
80000
70000
60000
50000
40000
30000
20000
10000
0
-10000
180
170
160
150
140
130
120
110
100
90
80
70
60
50
40
30
20
10
0
-10

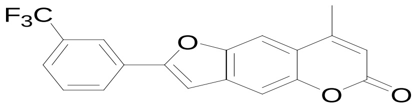
F3C
O
O
O

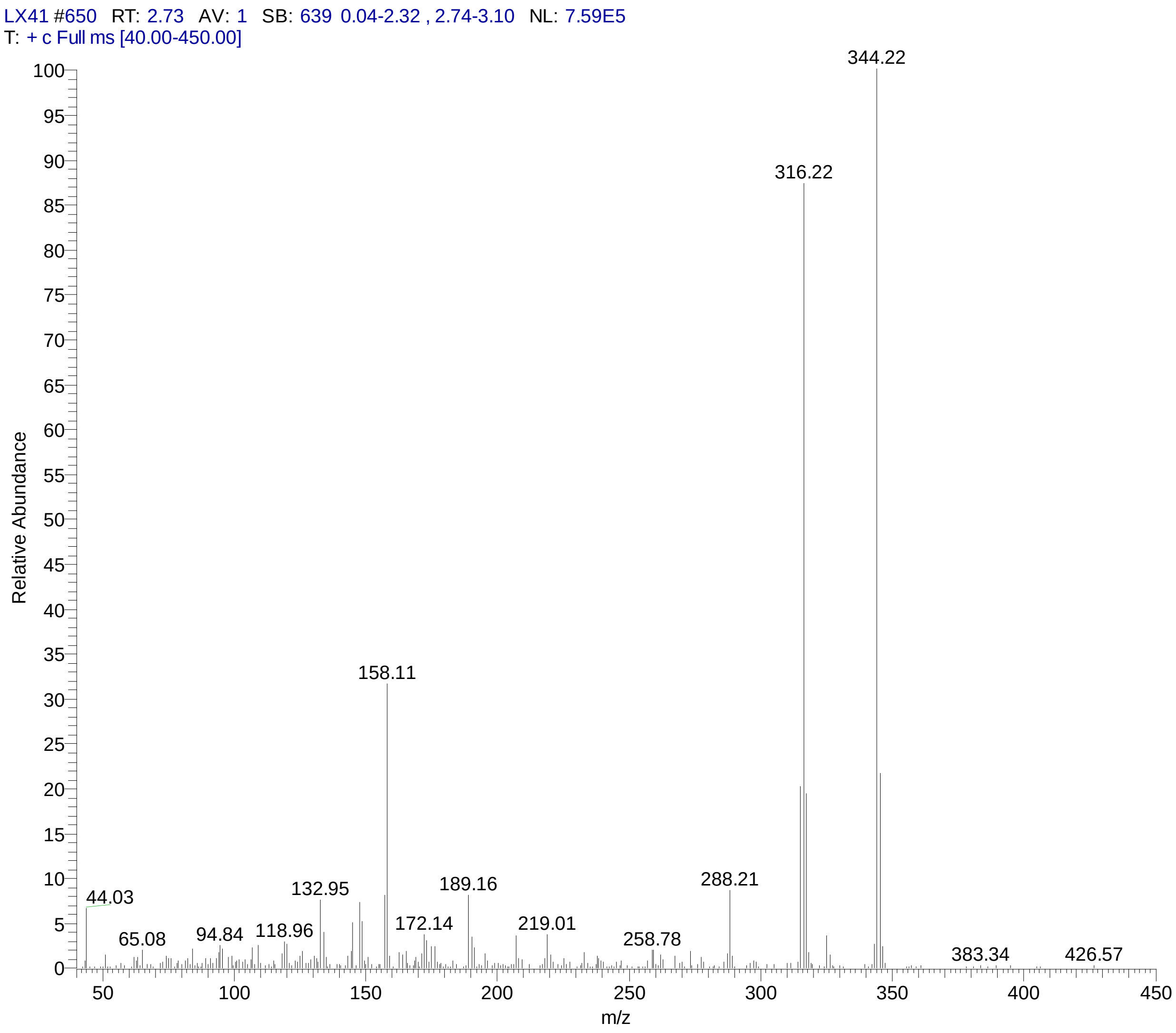
LX41 #650 RT: 2.73 AV: 1 SB: 639 0.04-2.32 , 2.74-3.10 NL: 7.59E5
T: + c Full ms [40.00-450.00]
344.22
316.22
158.11
44.03
65.08
94.84
118.96
132.95
172.14
189.16
219.01
258.78
288.21
383.34
426.57
Relative Abundance
m/z

MS of I24

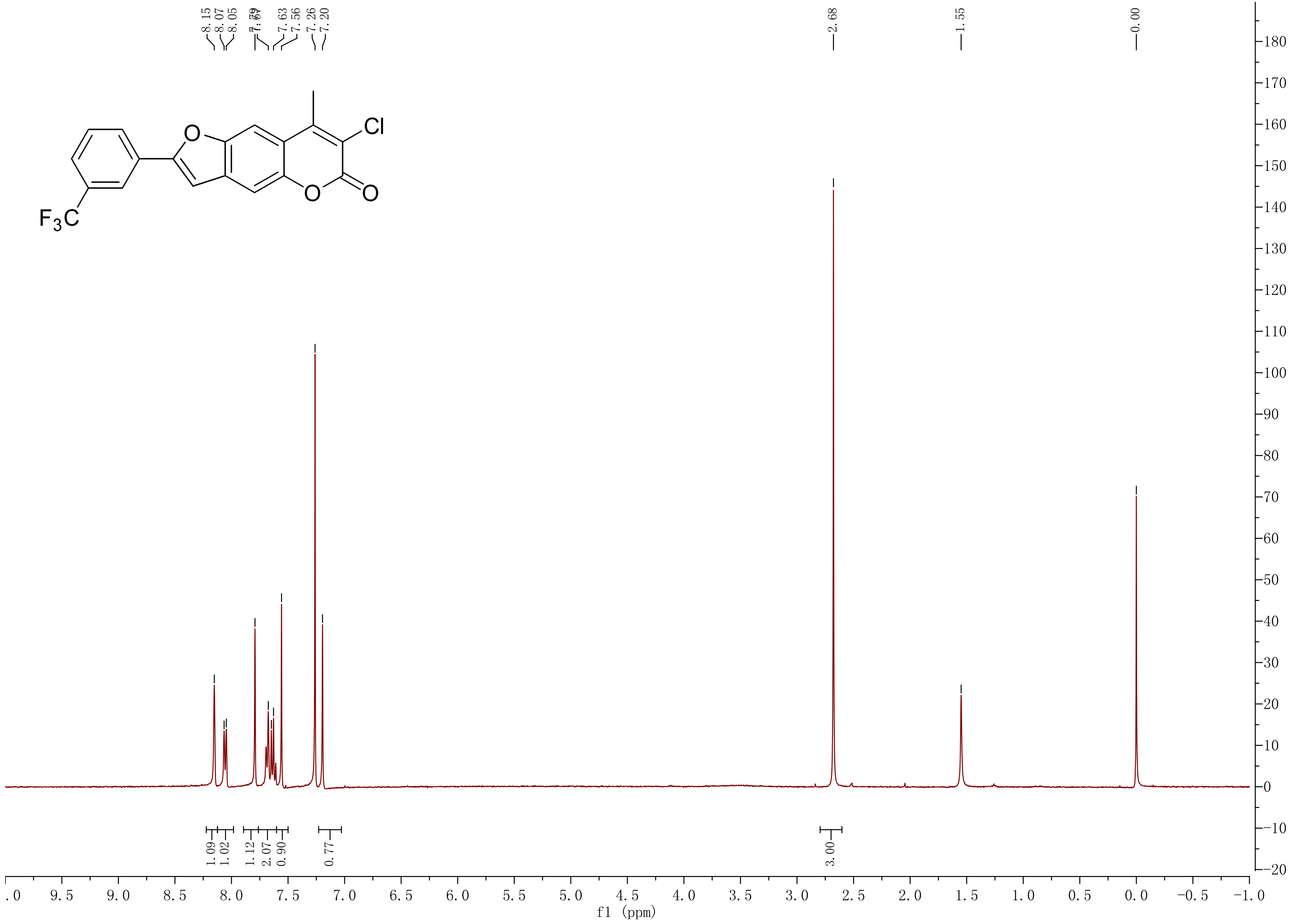

8.15
8.07
8.05
7.79
7.67
7.63
7.56
7.26
7.20
2.68
1.55
0.00
Cl
O
O
O
F3C
1.09
1.02
1.12
2.07
0.90
0.77
3.00
f1 (ppm)

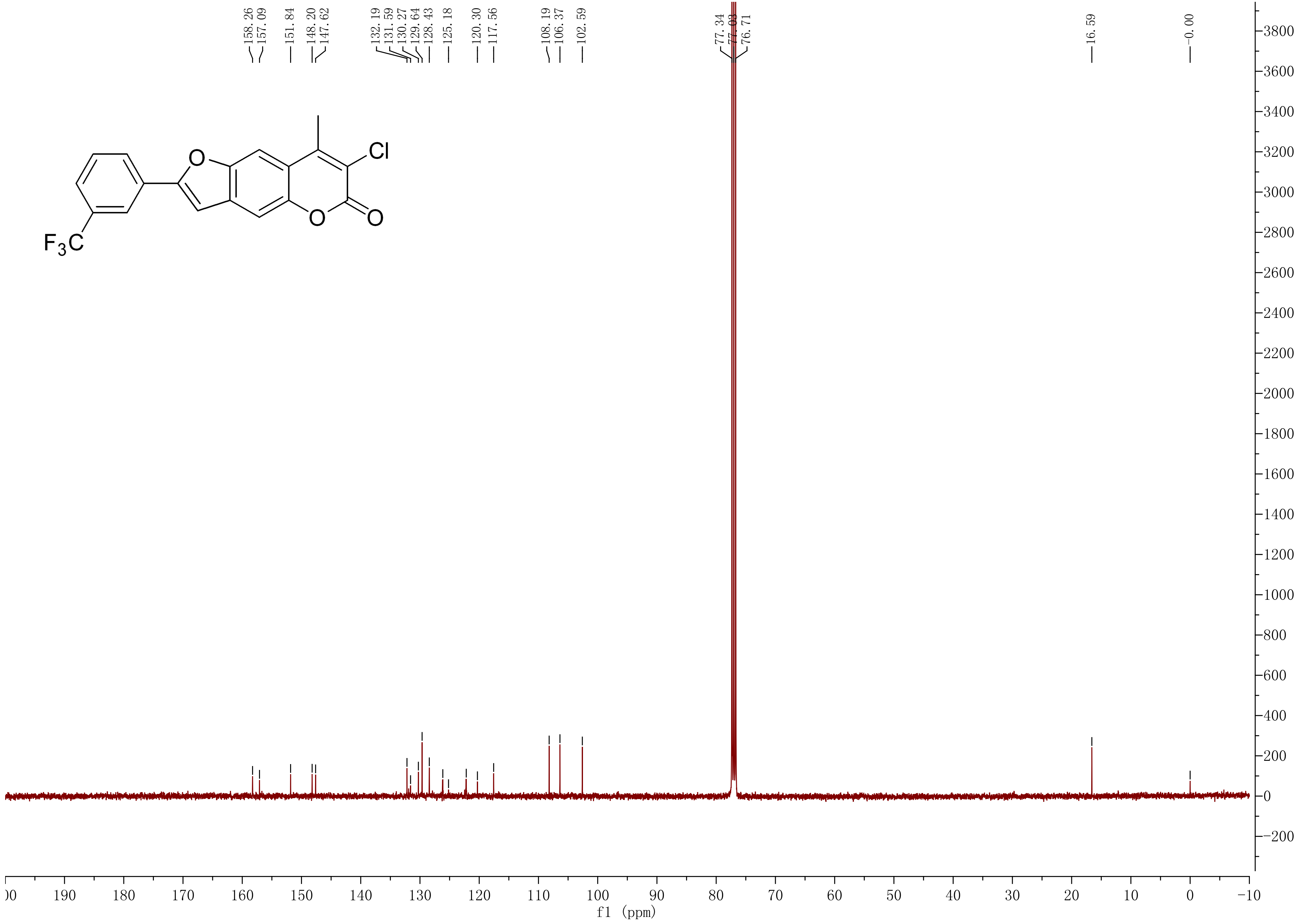

158.26
157.09
151.84
148.20
147.62
132.19
131.59
130.27
129.64
128.43
125.18
120.30
117.56
108.19
106.37
102.59
77.34
77.03
76.71
16.59
-0.00
Cl
O
O
O
F3C
f1 (ppm)

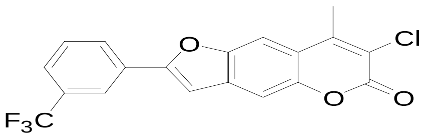
O
Cl
O
O
F3C

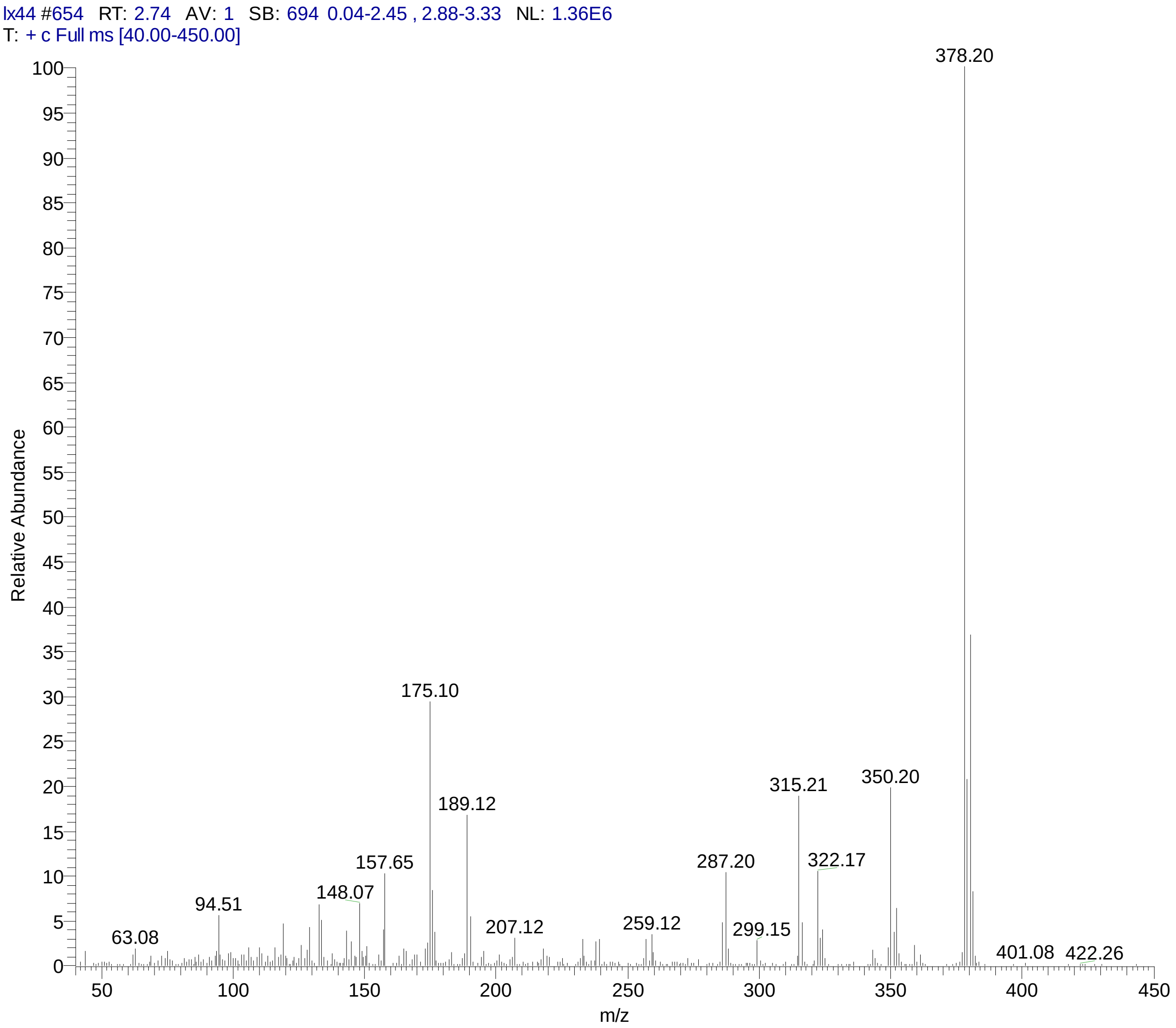
lx44 #654 RT: 2.74 AV: 1 SB: 694 0.04-2.45 , 2.88-3.33 NL: 1.36E6
T: + c Full ms [40.00-450.00]
378.20
175.10
189.12
157.65
148.07
94.51
63.08
207.12
259.12
287.20
299.15
315.21
322.17
350.20
401.08
422.26
Relative Abundance
m/z

MS of I25

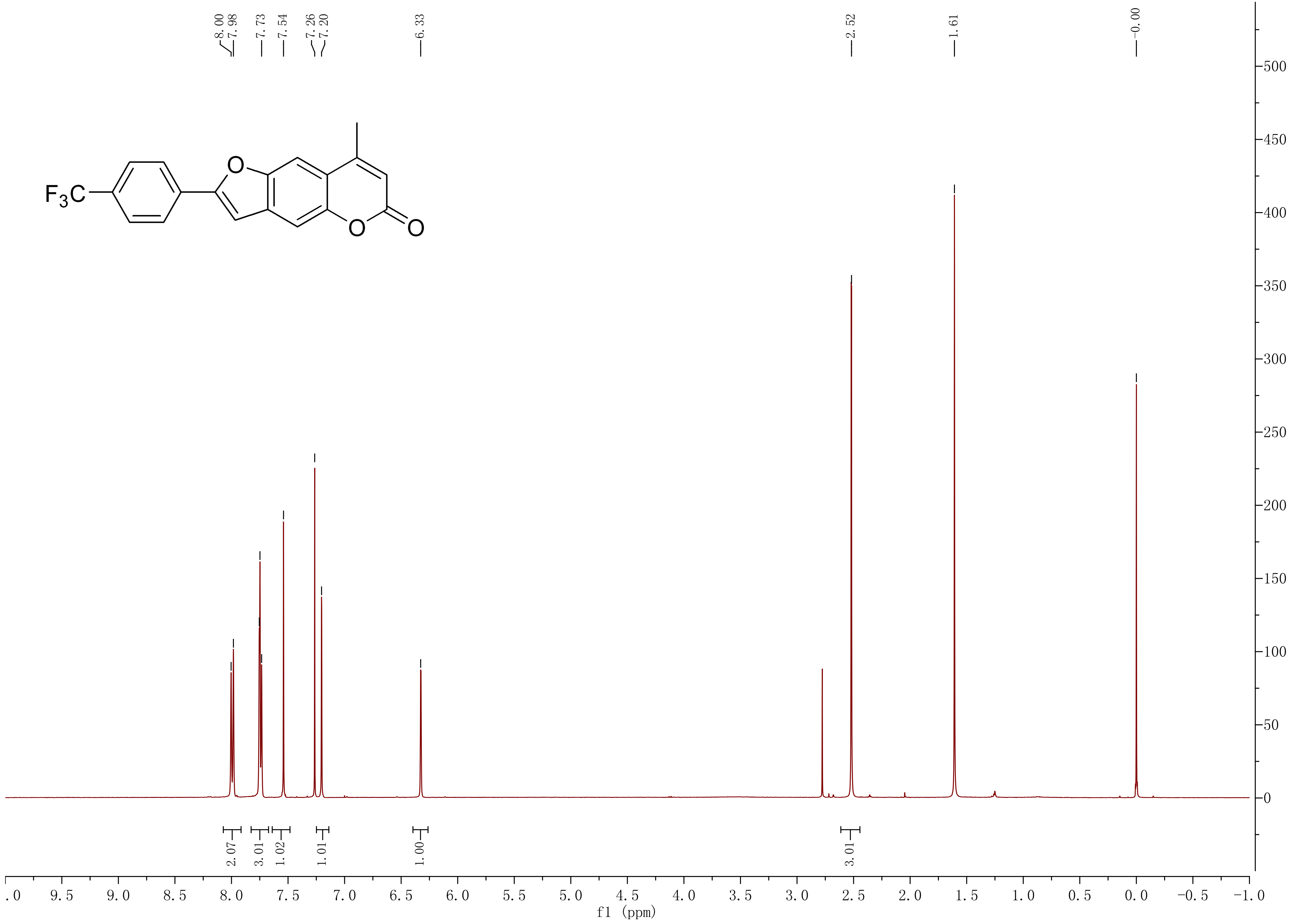

8.00
7.98
7.73
7.54
7.26
7.20
6.33
2.52
1.61
-0.00
F3C
O
O
O
2.07
3.01
1.02
1.01
1.00
3.01
500
450
400
350
300
250
200
150
100
50
0
.0
9.5
9.0
8.5
8.0
7.5
7.0
6.5
6.0
5.5
5.0
4.5
4.0
3.5
3.0
2.5
2.0
1.5
1.0
0.5
0.0
-0.5
-1.0
f1 (ppm)

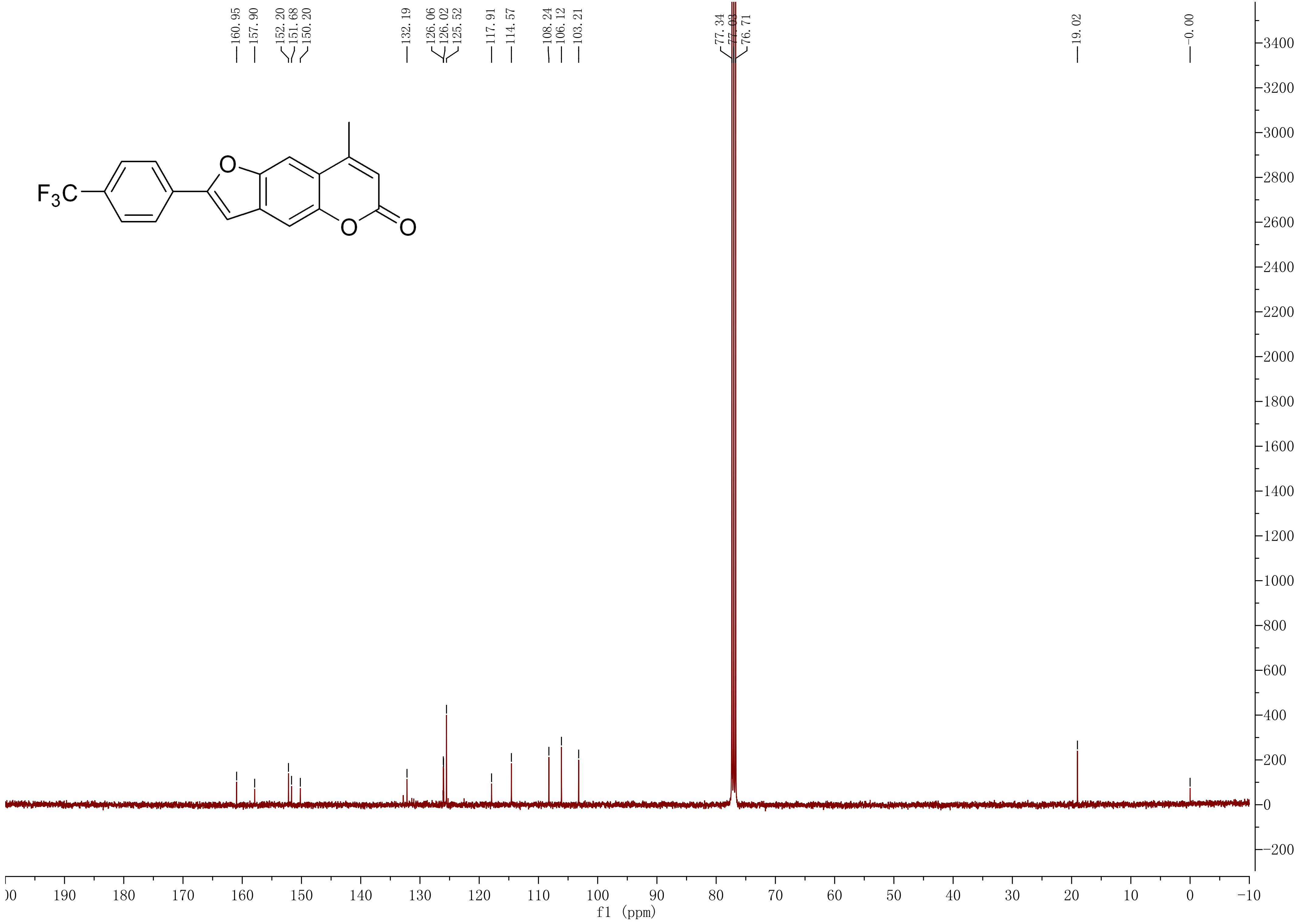
160.95
157.90
152.20
151.68
150.20
132.19
126.06
126.02
125.52
117.91
114.57
108.24
106.12
103.21
77.34
77.03
76.71
19.02
-0.00
F3C
O
O
O
3400
3200
3000
2800
2600
2400
2200
2000
1800
1600
1400
1200
1000
800
600
400
200
0
-200
190
180
170
160
150
140
130
120
110
100
90
80
70
60
50
40
30
20
10
0
-10
f1 (ppm)

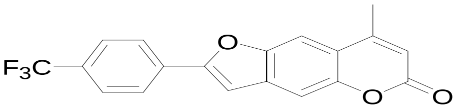

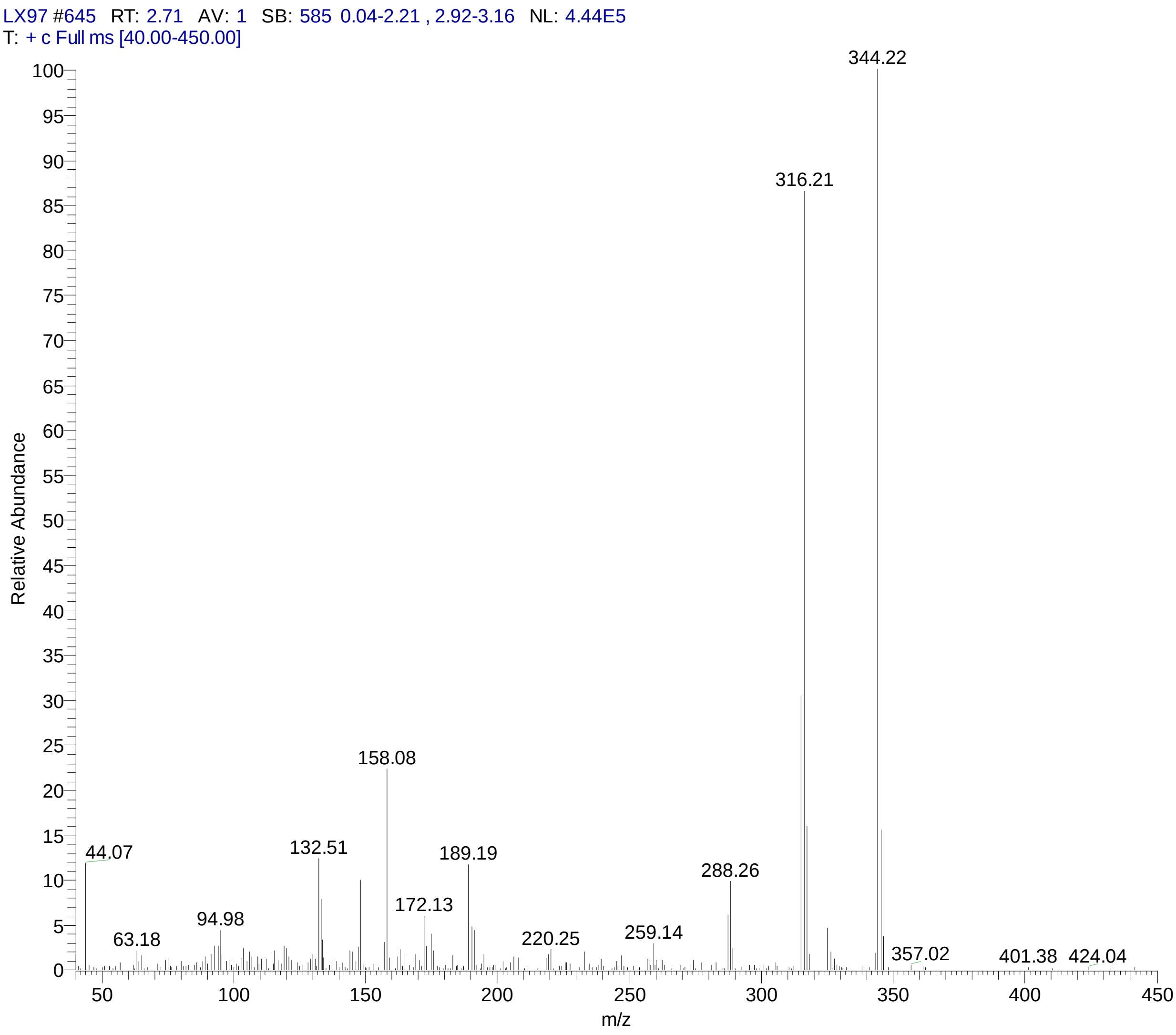
LX97 #645 RT: 2.71 AV: 1 SB: 585 0.04-2.21 , 2.92-3.16 NL: 4.44E5
T: + c Full ms [40.00-450.00]
Relative Abundance
m/z
344.22
316.21
158.08
132.51
189.19
172.13
288.26
44.07
94.98
63.18
220.25
259.14
357.02
401.38
424.04

MS of I26

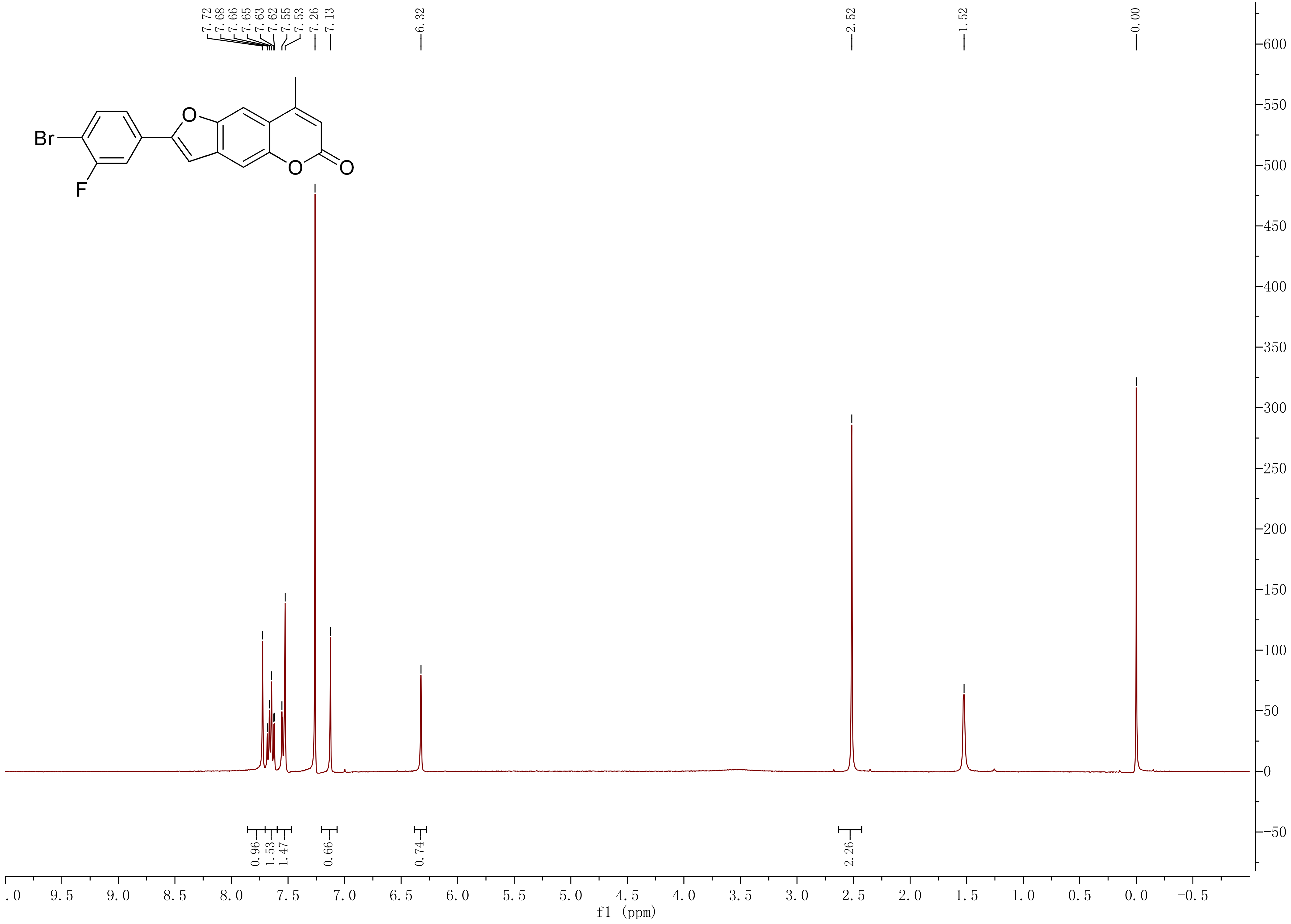

7.72
7.68
7.66
7.65
7.63
7.62
7.55
7.53
7.26
7.13
6.32
2.52
1.52
0.00
Br
F
O
O
O
0.96
1.53
1.47
0.66
0.74
2.26
f1 (ppm)

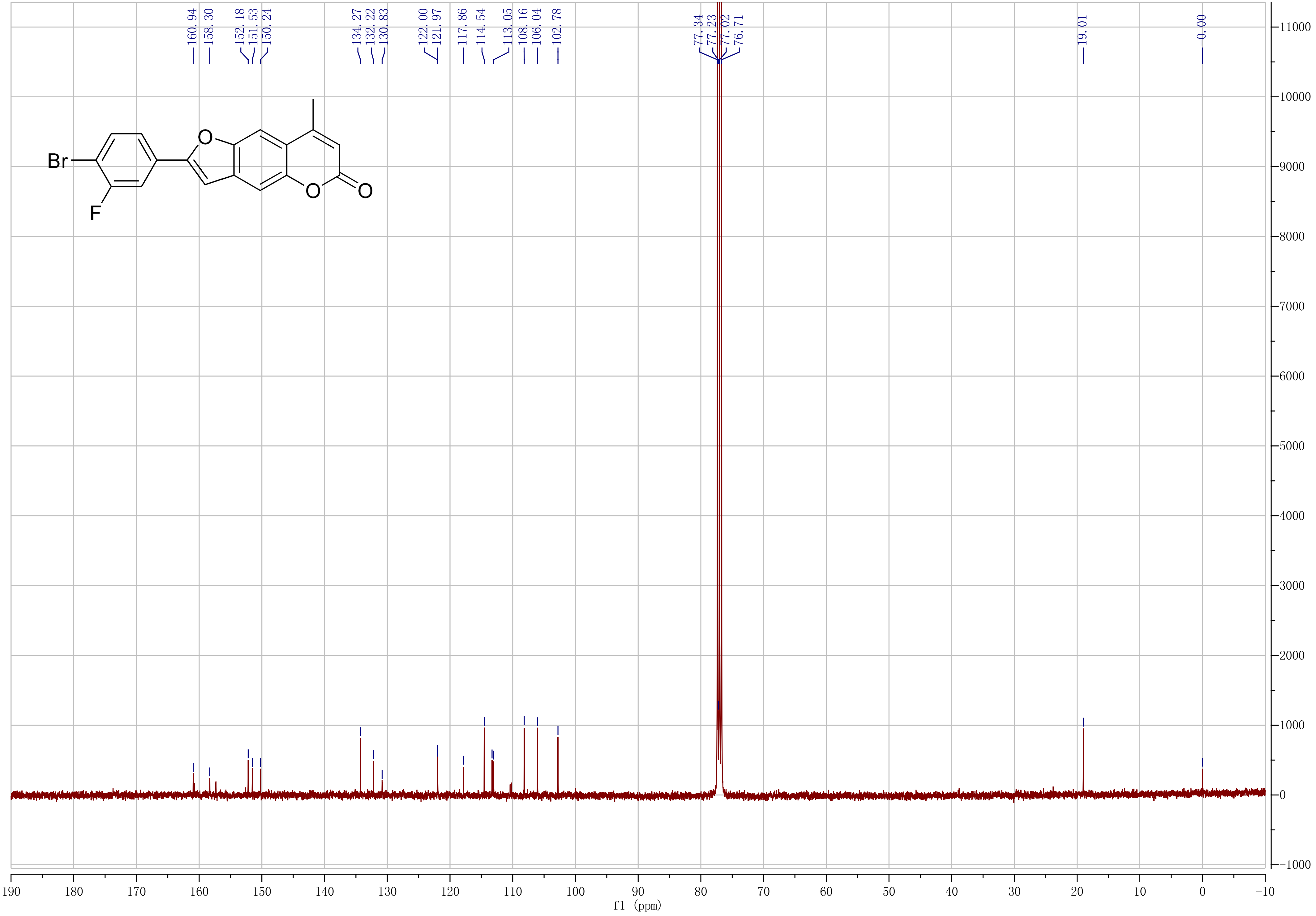
160.94
158.30
152.18
151.53
150.24
134.27
132.22
130.83
122.00
121.97
117.86
114.54
113.05
108.16
106.04
102.78
77.34
77.23
77.02
76.71
19.01
0.00
Br
F
O
O
O
f1 (ppm)

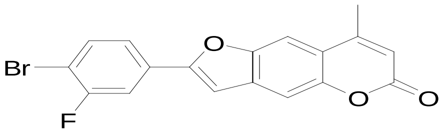

Br
F
O
O
O

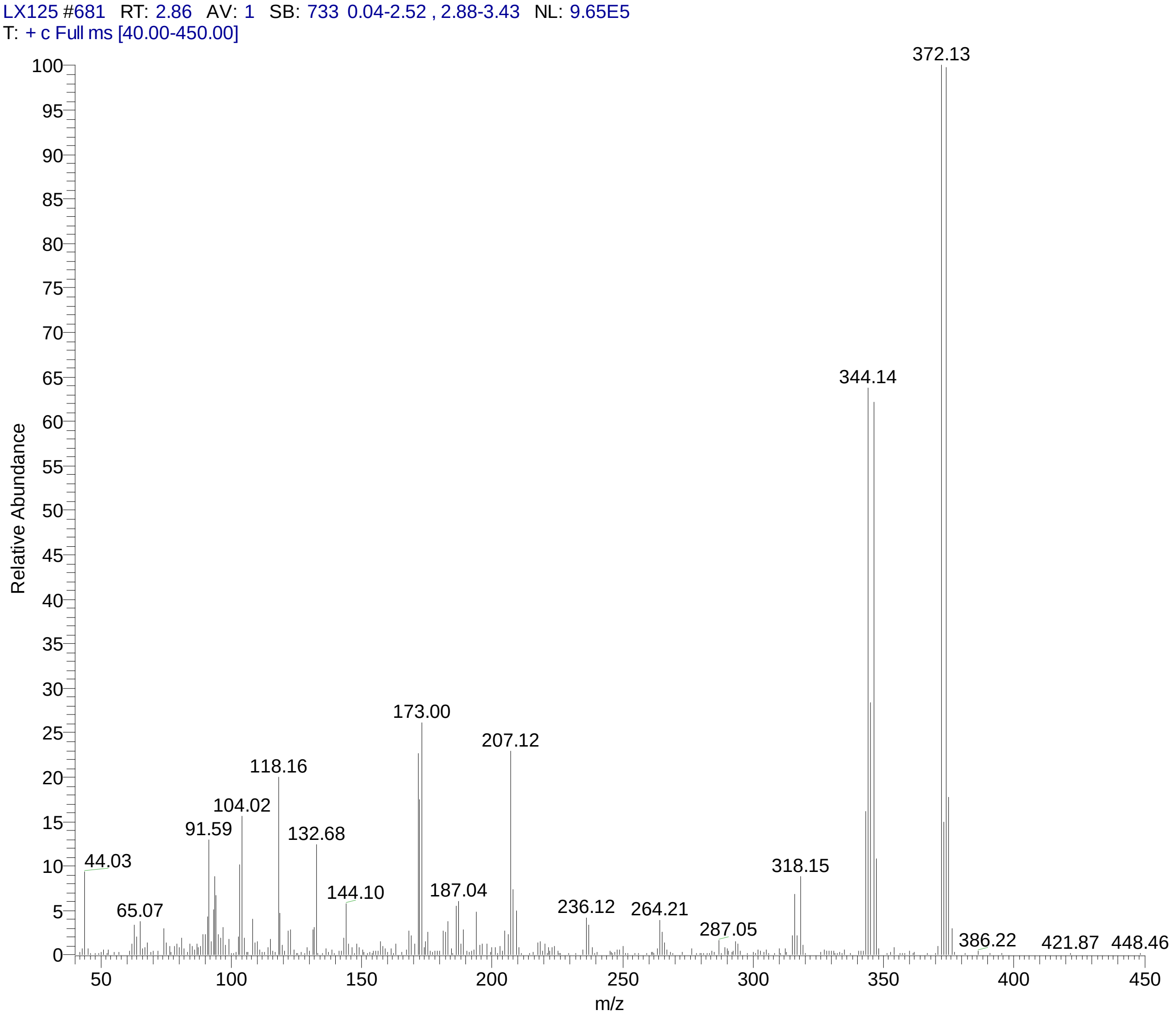

LX125 #681 RT: 2.86 AV: 1 SB: 733 0.04-2.52 , 2.88-3.43 NL: 9.65E5
T: + c Full ms [40.00-450.00]
Relative Abundance
m/z
372.13
344.14
318.15
287.05
264.21
236.12
207.12
187.04
173.00
144.10
132.68
118.16
104.02
91.59
65.07
44.03
386.22
421.87
448.46

MS of I27

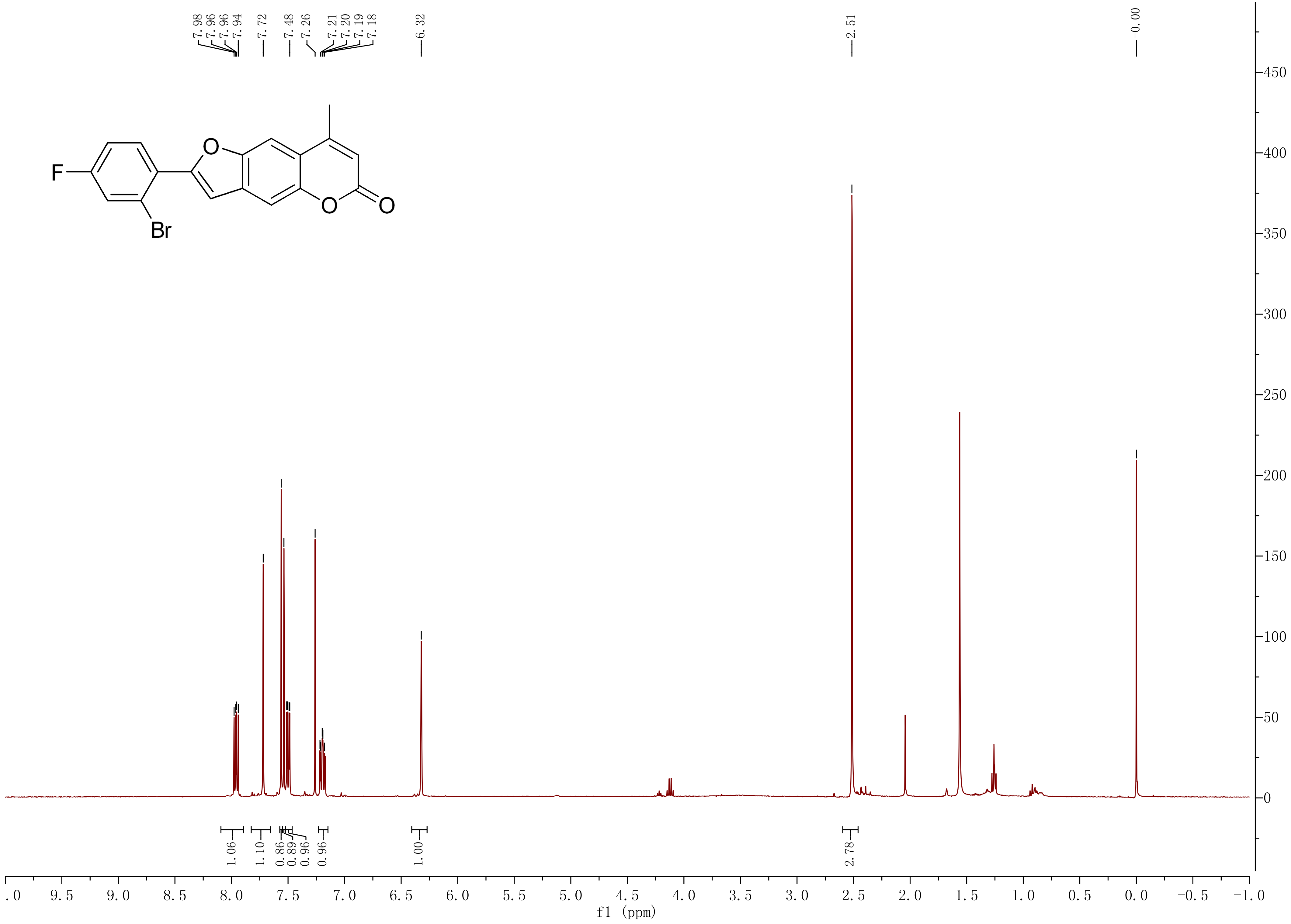
7.98
7.96
7.96
7.94
7.72
7.48
7.26
7.21
7.20
7.19
7.18
6.32
2.51
-0.00
F
O
O
O
Br
1.06
1.10
0.86
0.89
0.96
0.96
1.00
2.78
f1 (ppm)

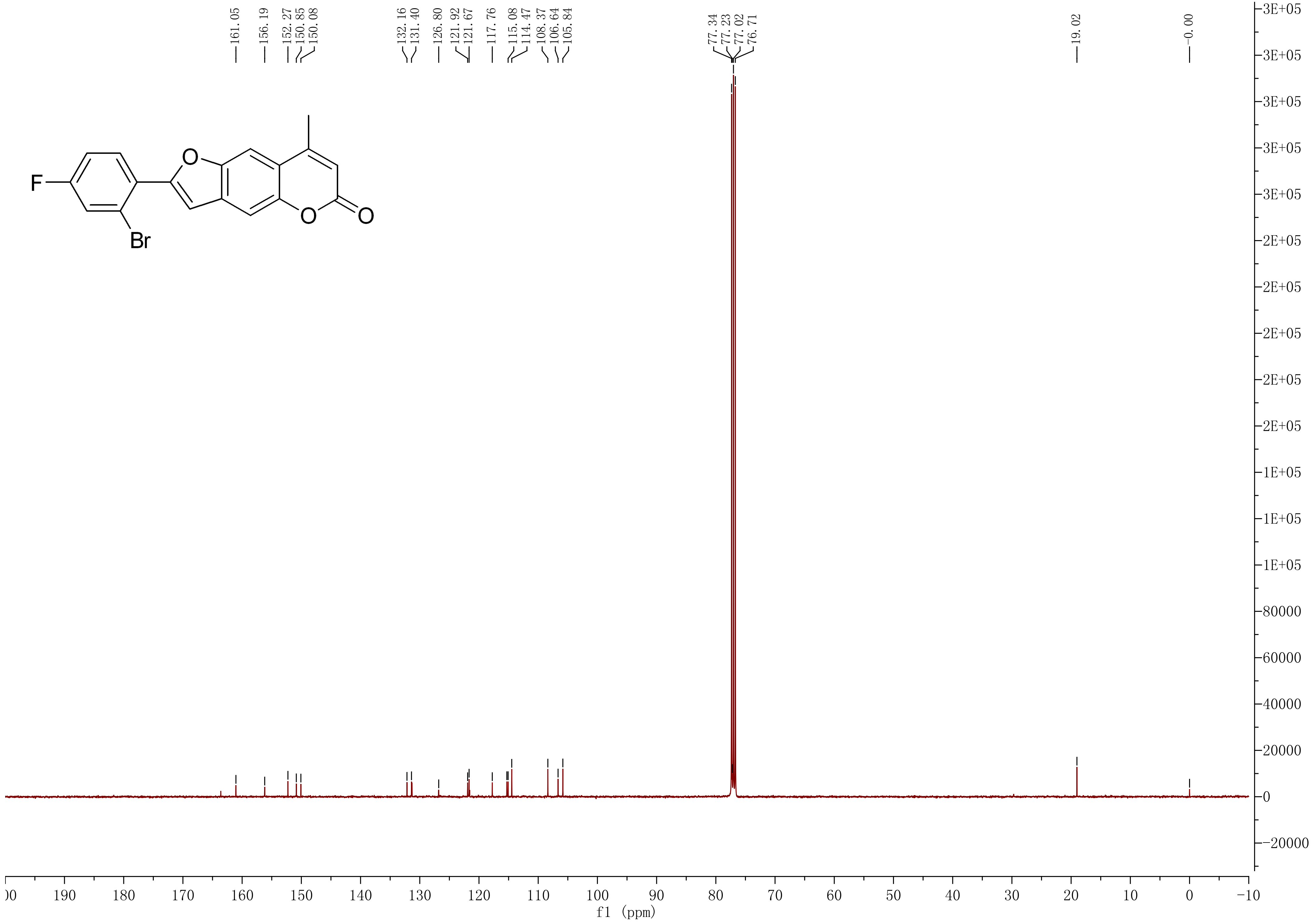
161.05
156.19
152.27
150.85
150.08
132.16
131.40
126.80
121.92
121.67
117.76
115.08
114.47
108.37
106.64
105.84
77.34
77.23
77.02
76.71
19.02
-0.00
F
Br
O
O
O
f1 (ppm)

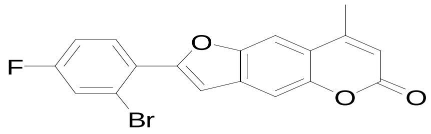
F
O
O
O
Br

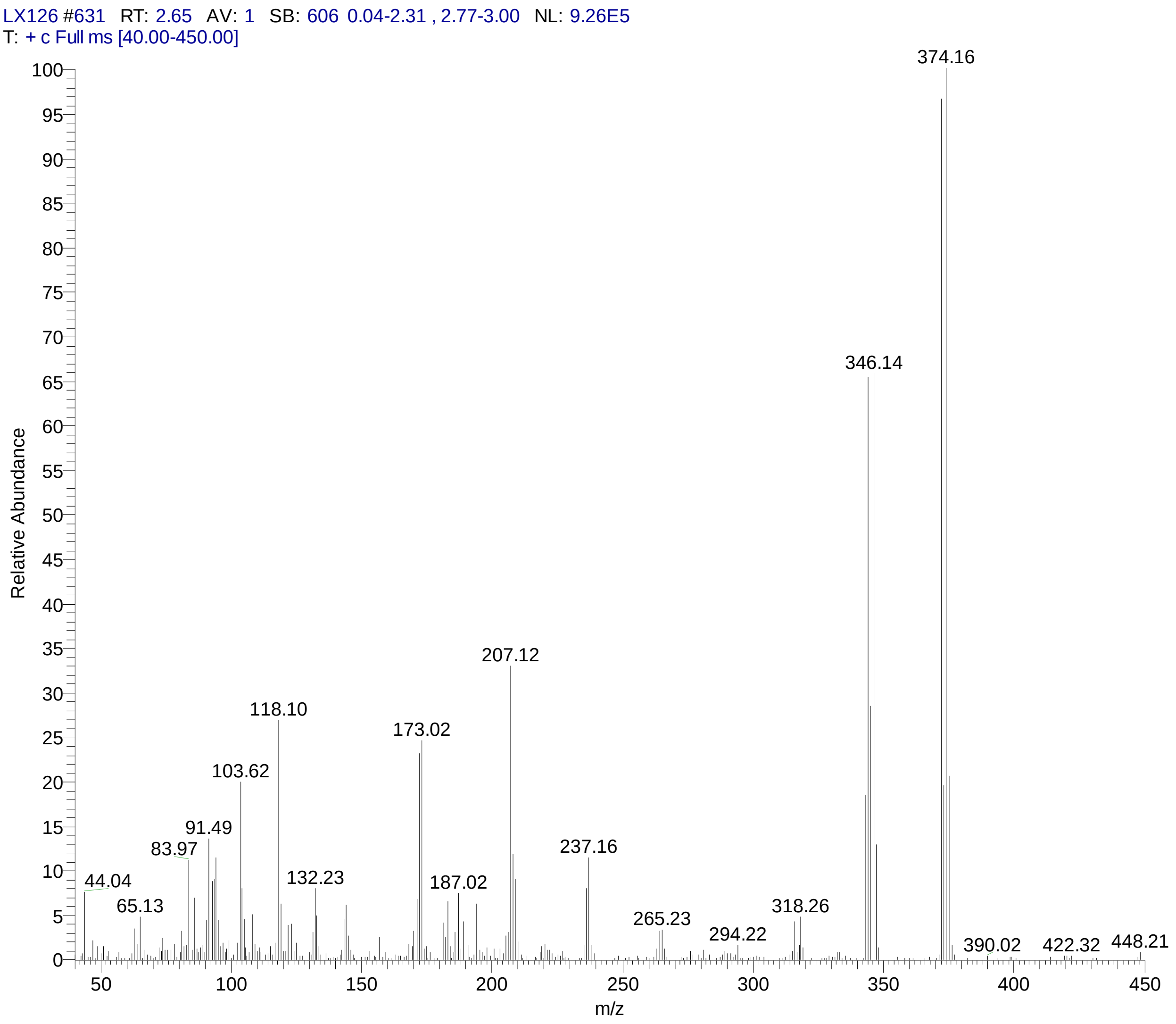
LX126 #631 RT: 2.65 AV: 1 SB: 606 0.04-2.31 , 2.77-3.00 NL: 9.26E5
T: + c Full ms [40.00-450.00]
Relative Abundance
m/z
374.16
346.14
207.12
118.10
173.02
103.62
91.49
83.97
237.16
44.04
132.23
187.02
65.13
265.23
318.26
294.22
390.02
422.32
448.21

MS of I28

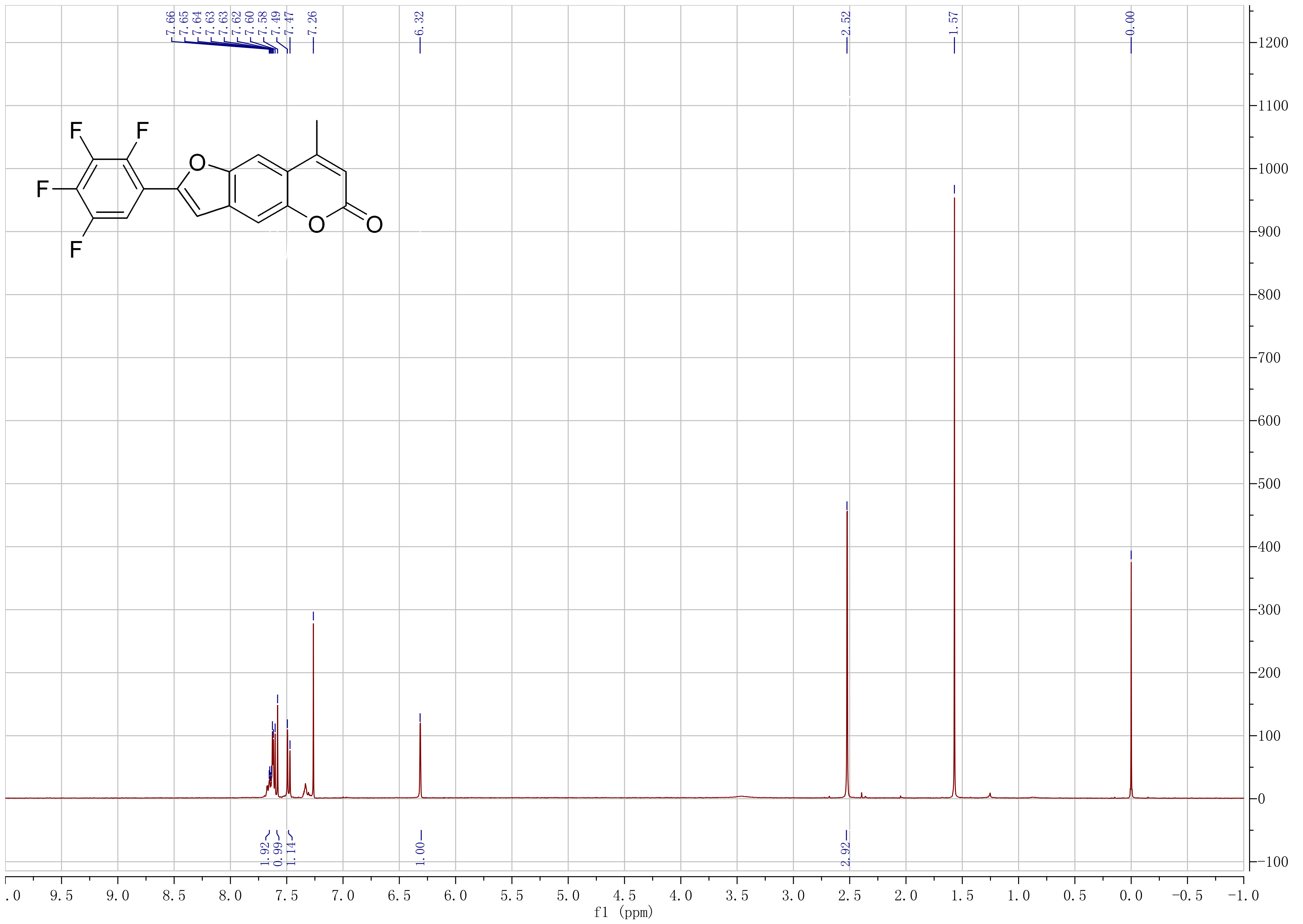

7.66
7.65
7.64
7.63
7.63
7.62
7.60
7.58
7.49
7.47
7.26
6.32
2.52
1.57
0.00
1.92
0.99
1.14
1.00
2.92
f1 (ppm)

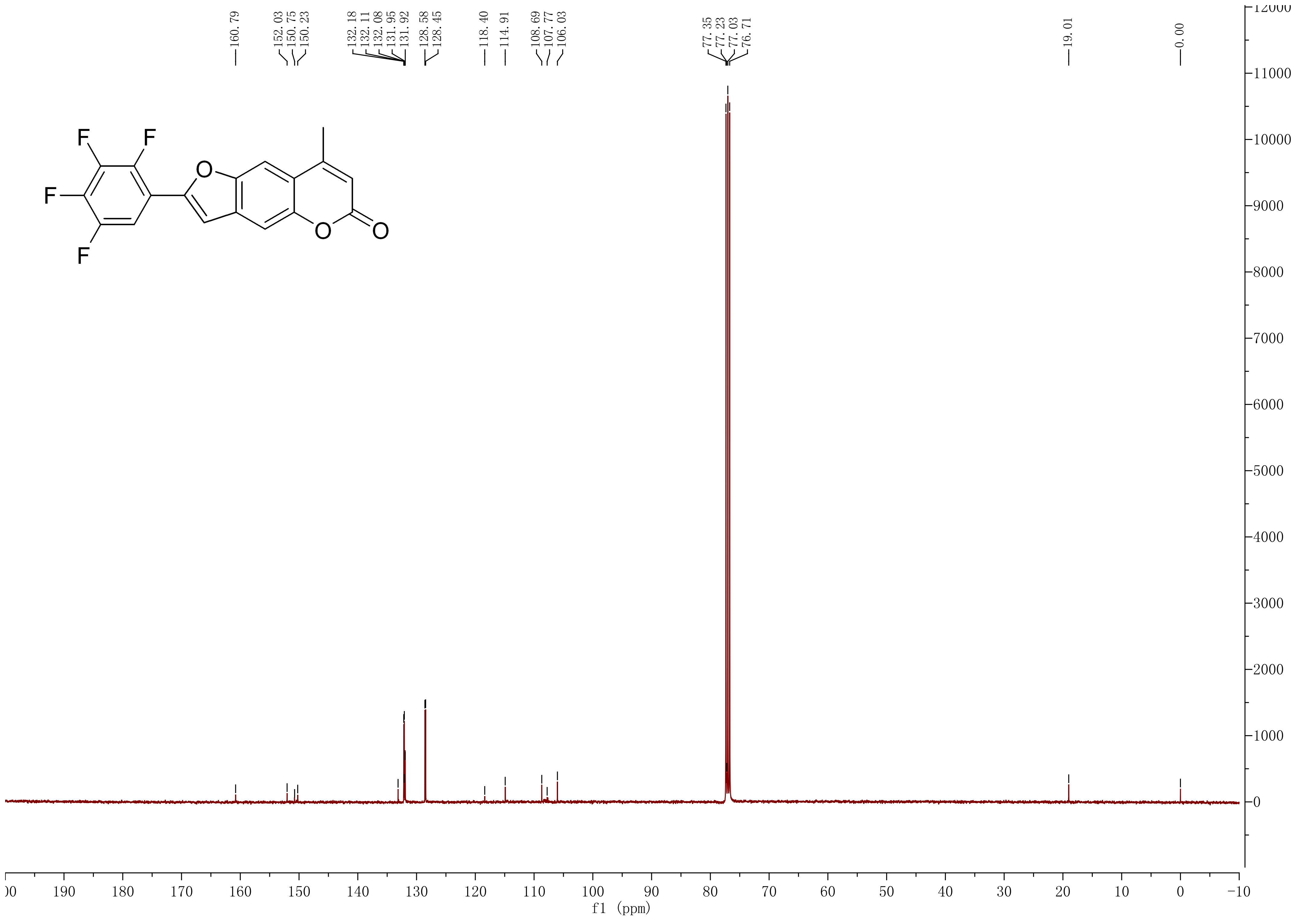
160.79
152.03
150.75
150.23
132.18
132.11
132.08
131.95
131.92
128.58
128.45
118.40
114.91
108.69
107.77
106.03
77.35
77.23
77.03
76.71
19.01
0.00
F
F
F
F
O
O
O
f1 (ppm)

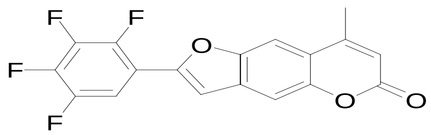

F
F
F
F
O
O
O

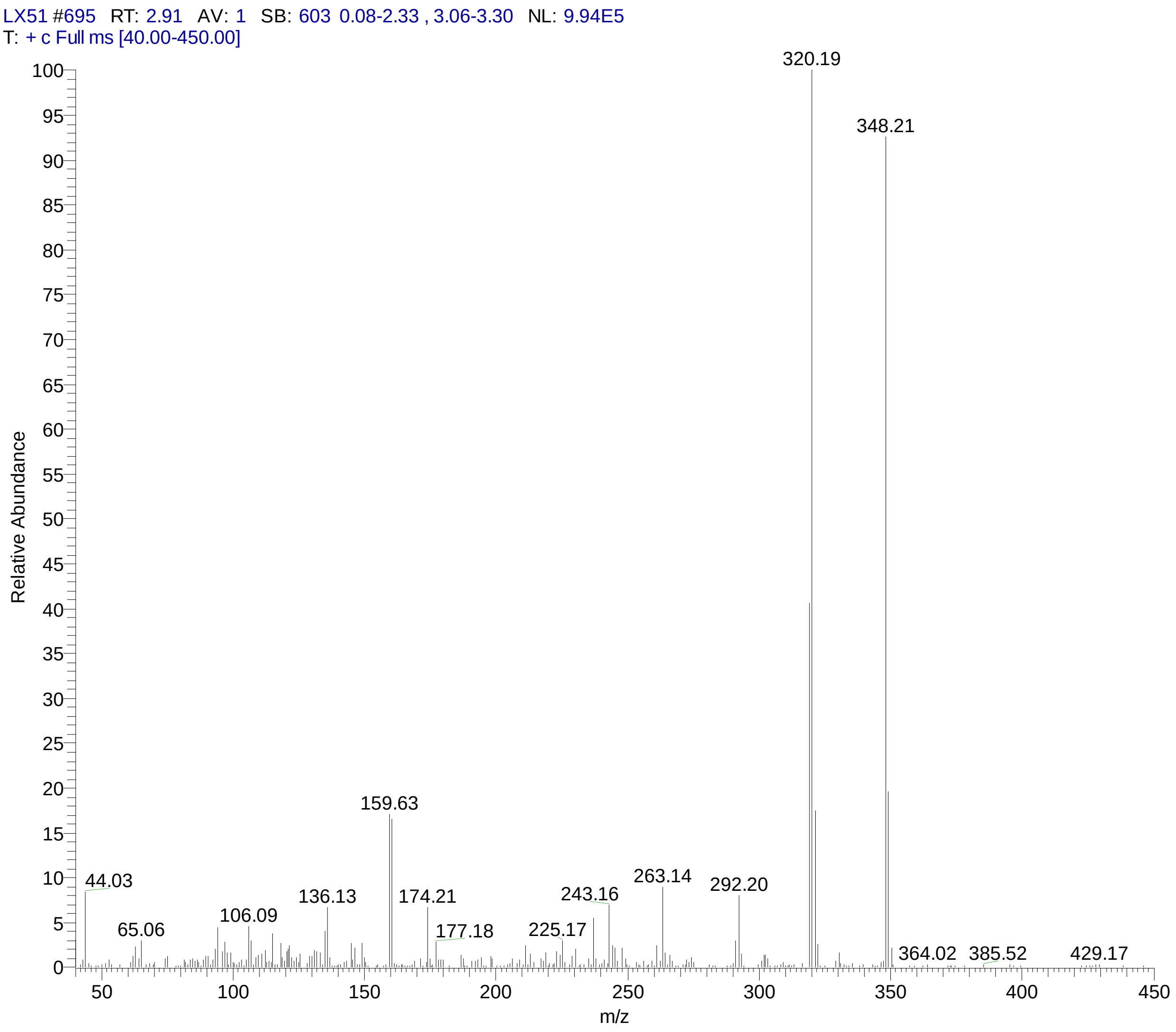

LX51 #695 RT: 2.91 AV: 1 SB: 603 0.08-2.33 , 3.06-3.30 NL: 9.94E5
T: + c Full ms [40.00-450.00]
Relative Abundance
m/z
320.19
348.21
159.63
44.03
65.06
106.09
136.13
174.21
177.18
225.17
243.16
263.14
292.20
364.02
385.52
429.17

MS of I29

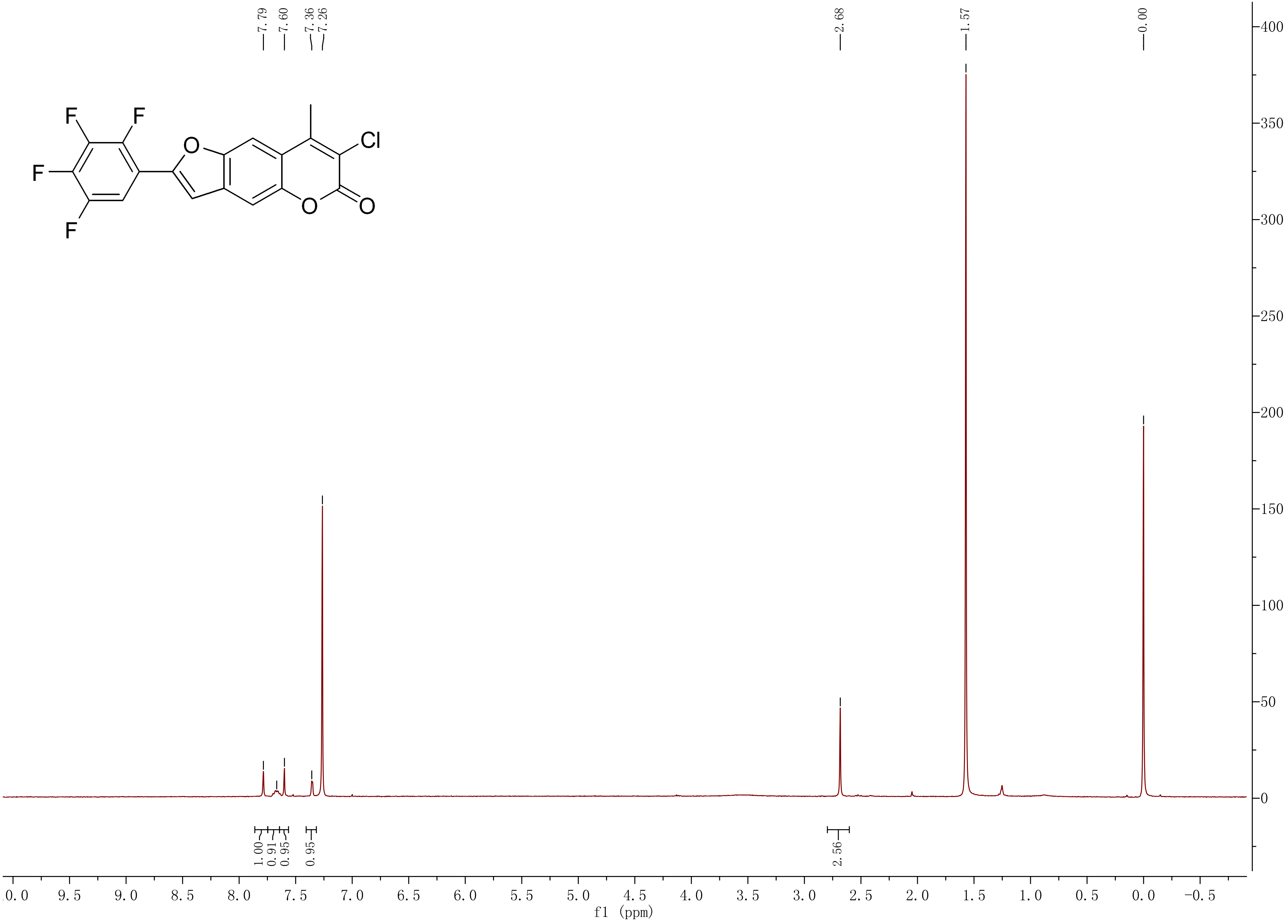

7.79
7.60
7.36
7.26
2.68
1.57
0.00
F
F
F
F
O
Cl
O
O
1.00
0.91
0.95
0.95
2.56
f1 (ppm)

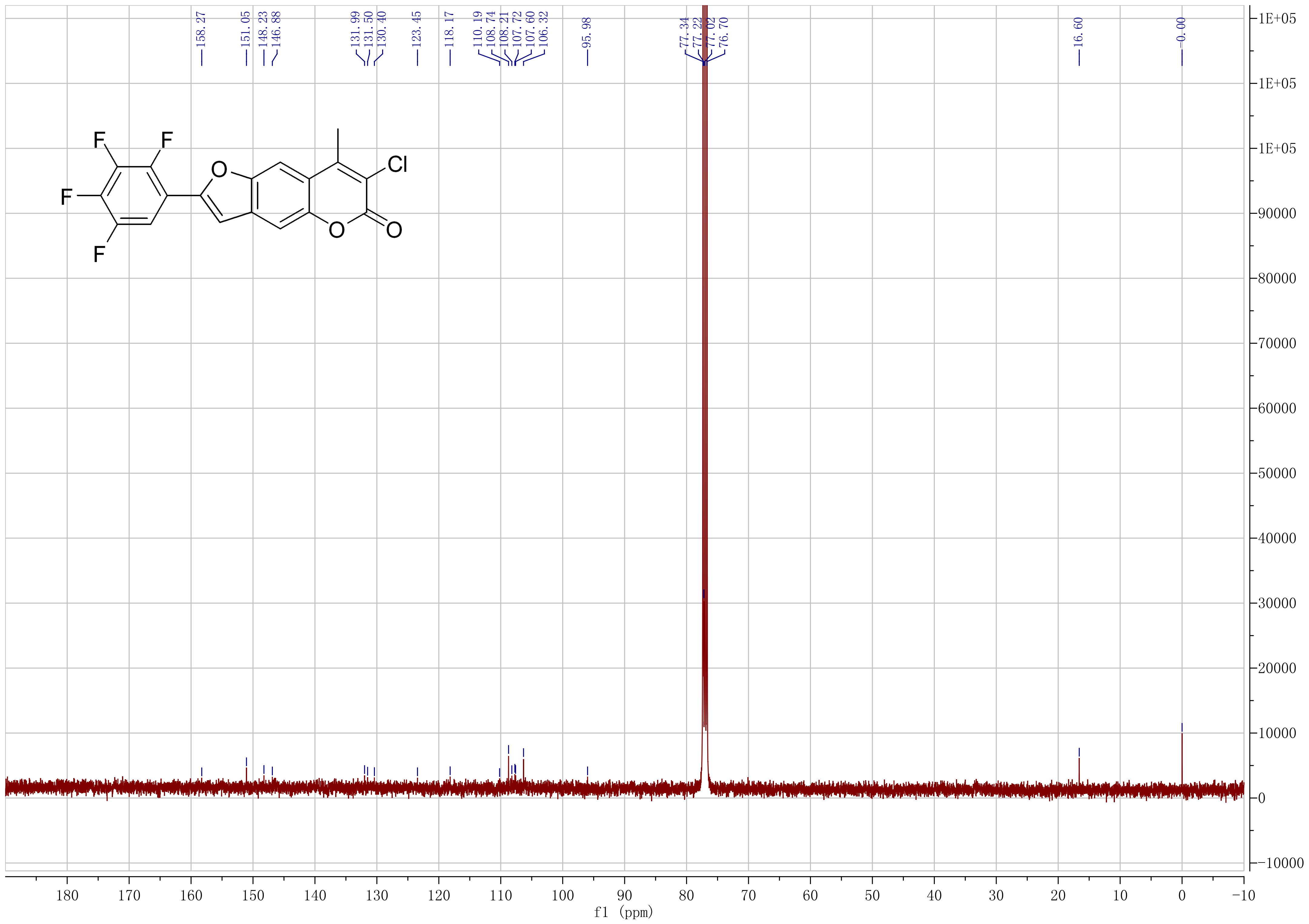
158.27
151.05
148.23
146.88
131.99
131.50
130.40
123.45
118.17
110.19
108.74
108.21
107.72
107.60
106.32
95.98
77.34
77.22
77.02
76.70
16.60
-0.00
F
F
F
F
O
Cl
O
O
f1 (ppm)

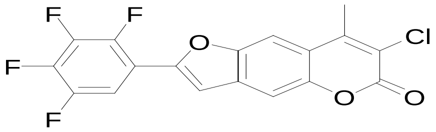

F
F
F
F
O
Cl
O
O

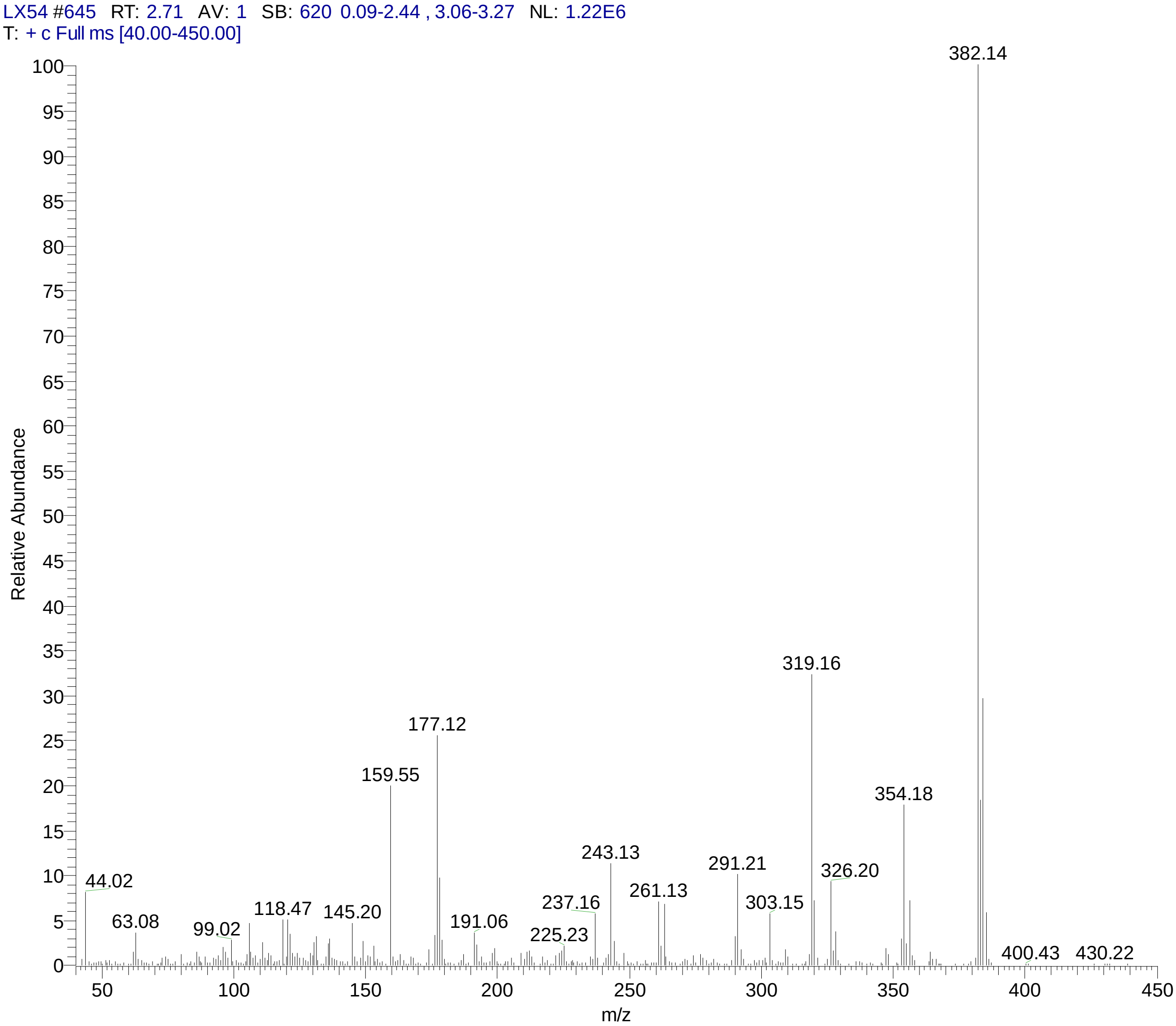

LX54 #645 RT: 2.71 AV: 1 SB: 620 0.09-2.44 , 3.06-3.27 NL: 1.22E6
T: + c Full ms [40.00-450.00]
Relative Abundance
m/z
382.14
319.16
177.12
159.55
354.18
243.13
291.21
326.20
44.02
261.13
237.16
303.15
118.47
145.20
63.08
191.06
99.02
225.23
400.43
430.22

MS of I30

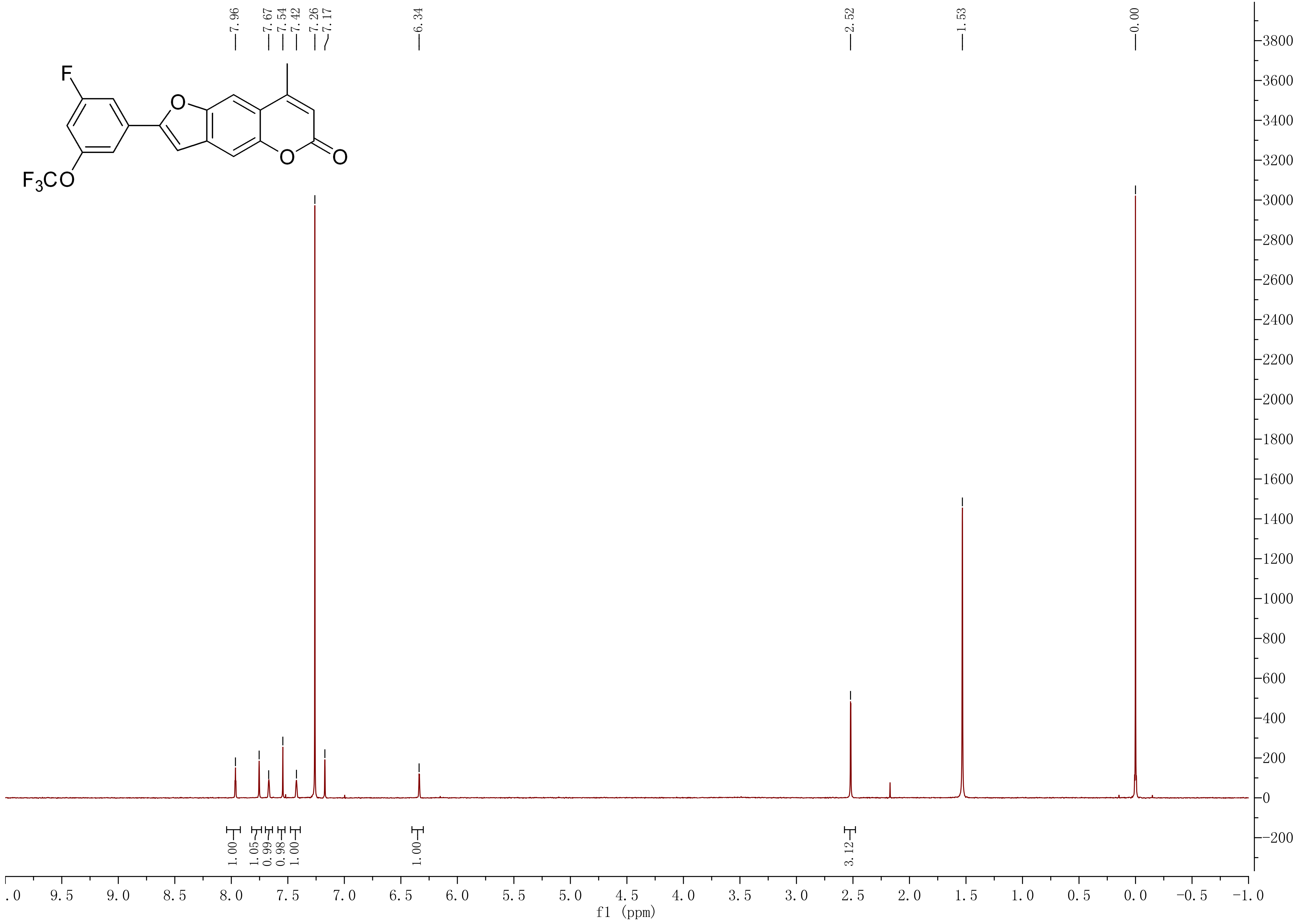

7.96
7.67
7.54
7.42
7.26
7.17
6.34
2.52
1.53
0.00
F
O
O
O
F3CO
1.00
1.05
0.99
0.98
1.00
1.00
3.12
f1 (ppm)

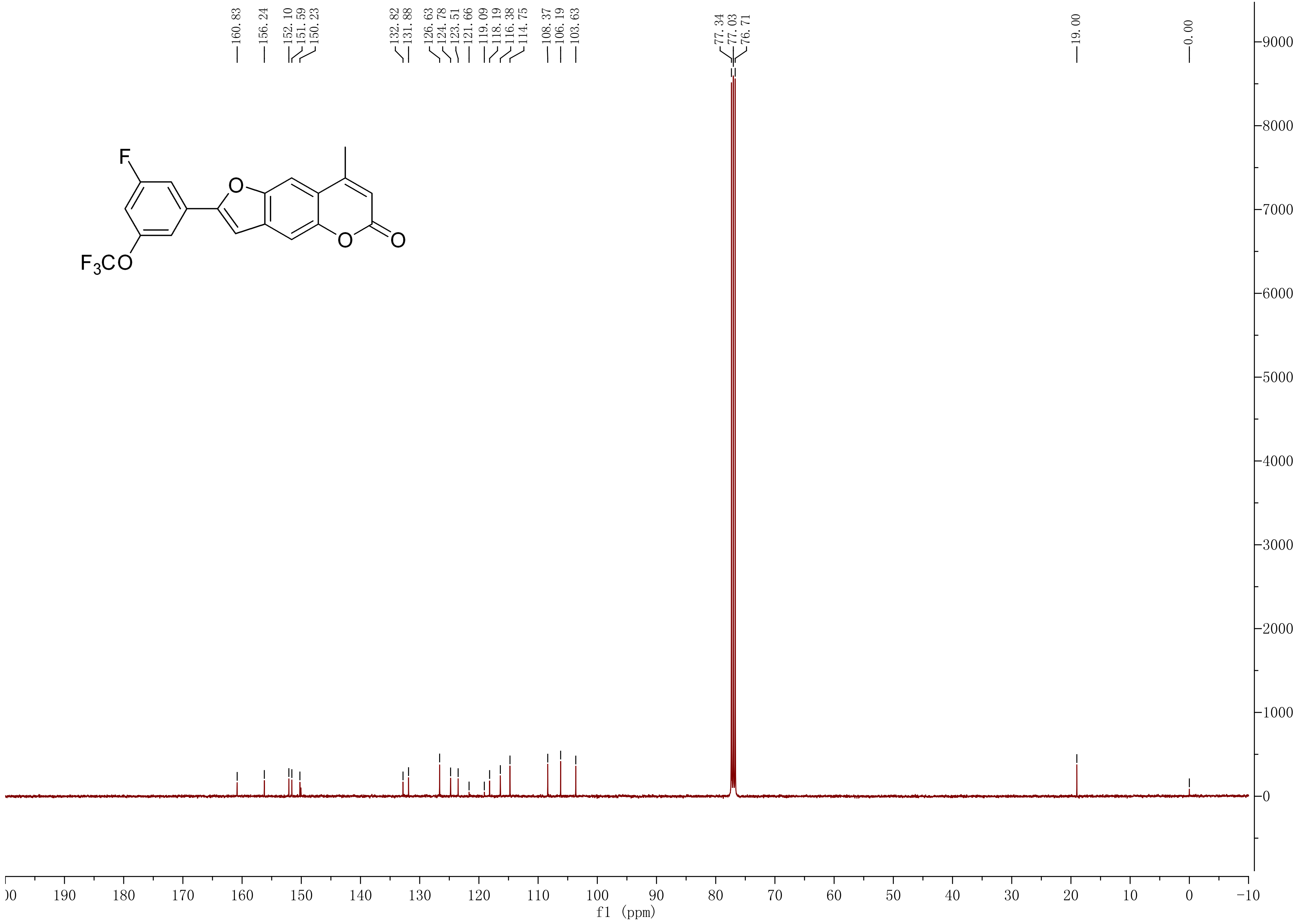
160.83
156.24
152.10
151.59
150.23
132.82
131.88
126.63
124.78
123.51
121.66
119.09
118.19
116.38
114.75
108.37
106.19
103.63
77.34
77.03
76.71
19.00
0.00
F
O
O
O
F3CO
f1 (ppm)

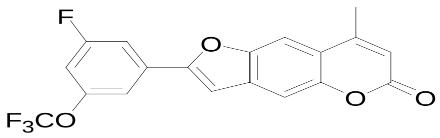
F
F3CO
O
O
O

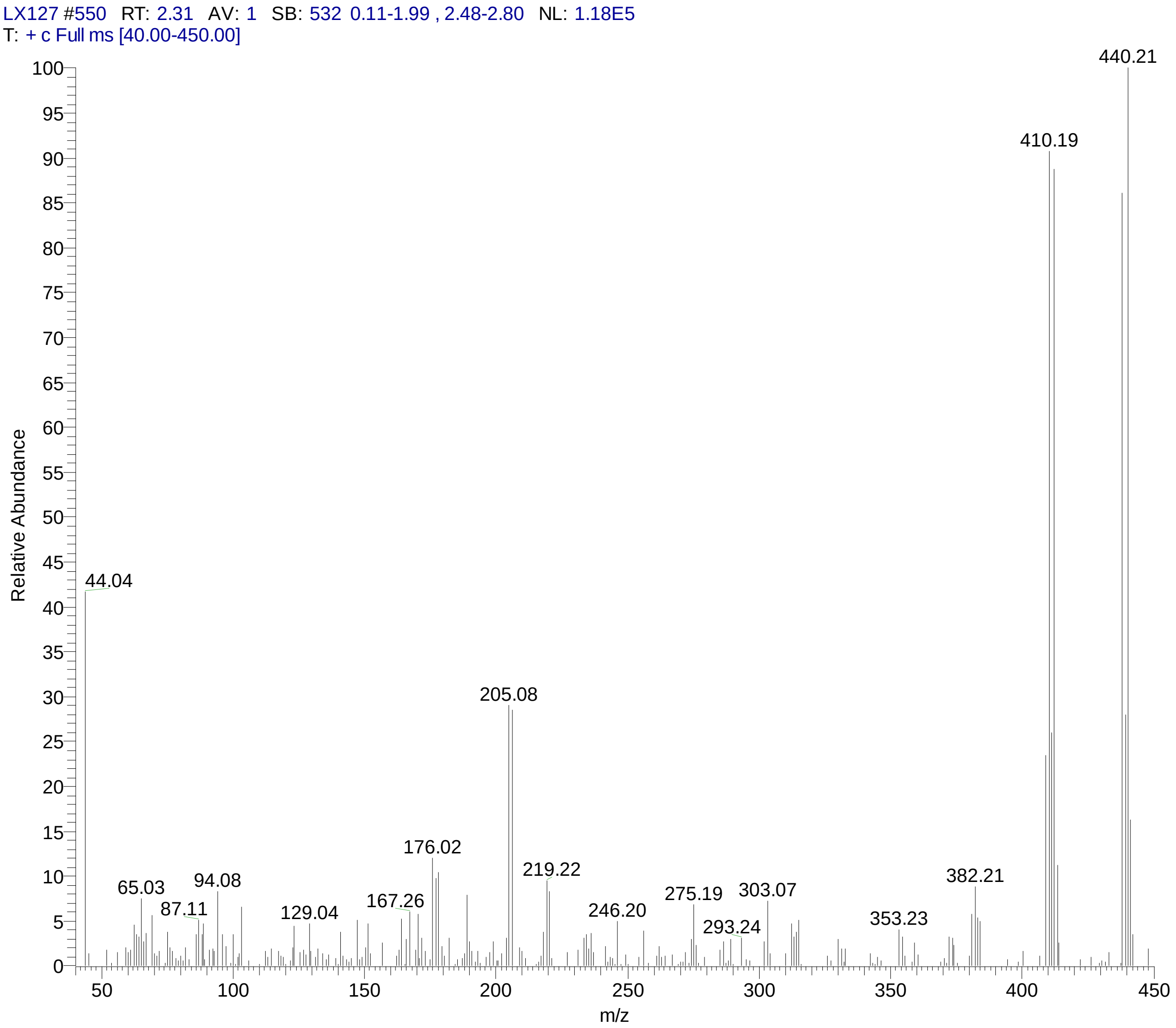
LX127 #550 RT: 2.31 AV: 1 SB: 532 0.11-1.99 , 2.48-2.80 NL: 1.18E5
T: + c Full ms [40.00-450.00]
Relative Abundance
m/z
440.21
410.19
44.04
205.08
176.02
219.22
65.03
94.08
87.11
129.04
167.26
246.20
275.19
303.07
293.24
353.23
382.21

MS of I31

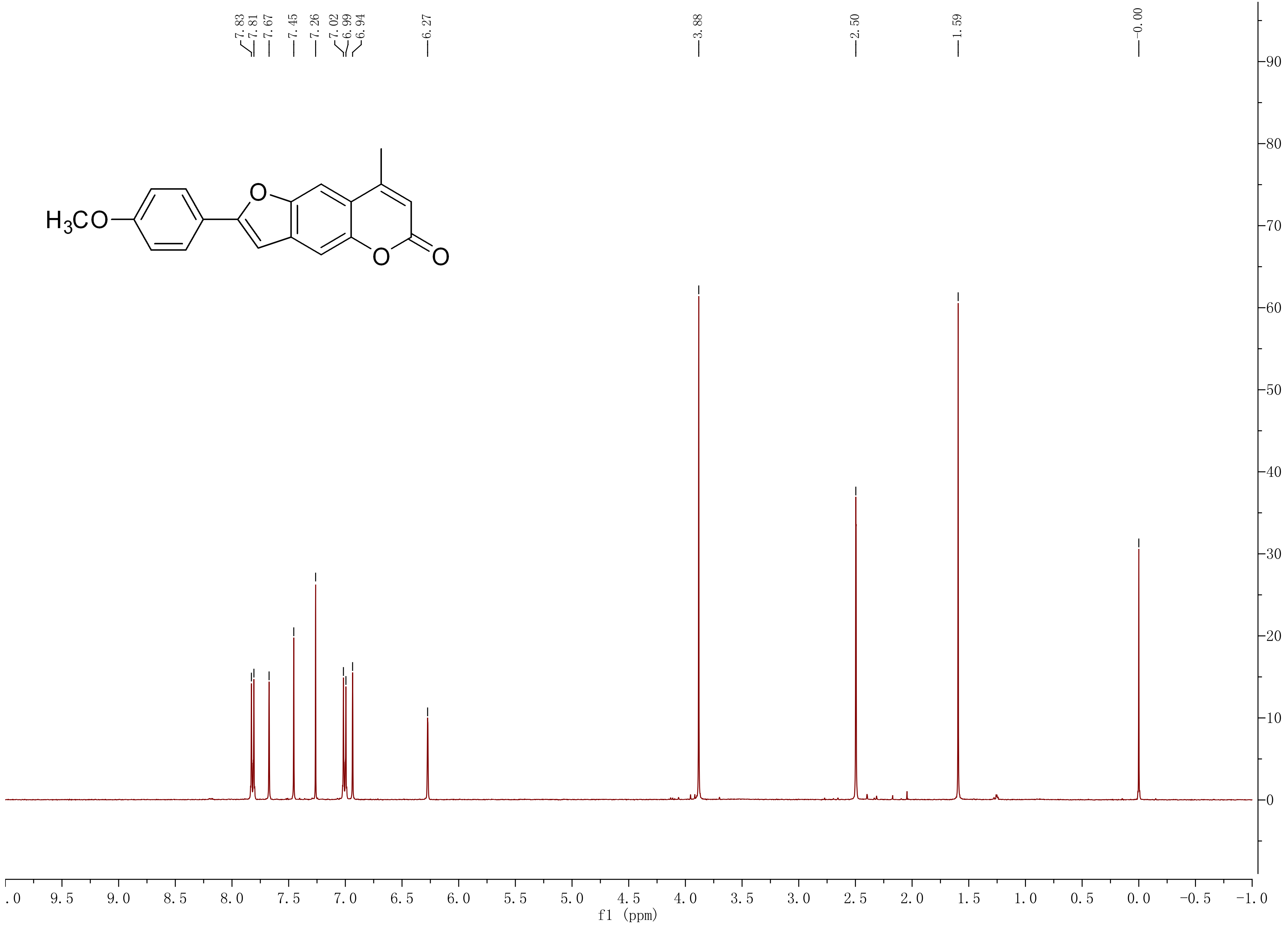
H3CO
O
O
O
7.83
7.81
7.67
7.45
7.26
7.02
6.99
6.94
6.27
3.88
2.50
1.59
-0.00
f1 (ppm)

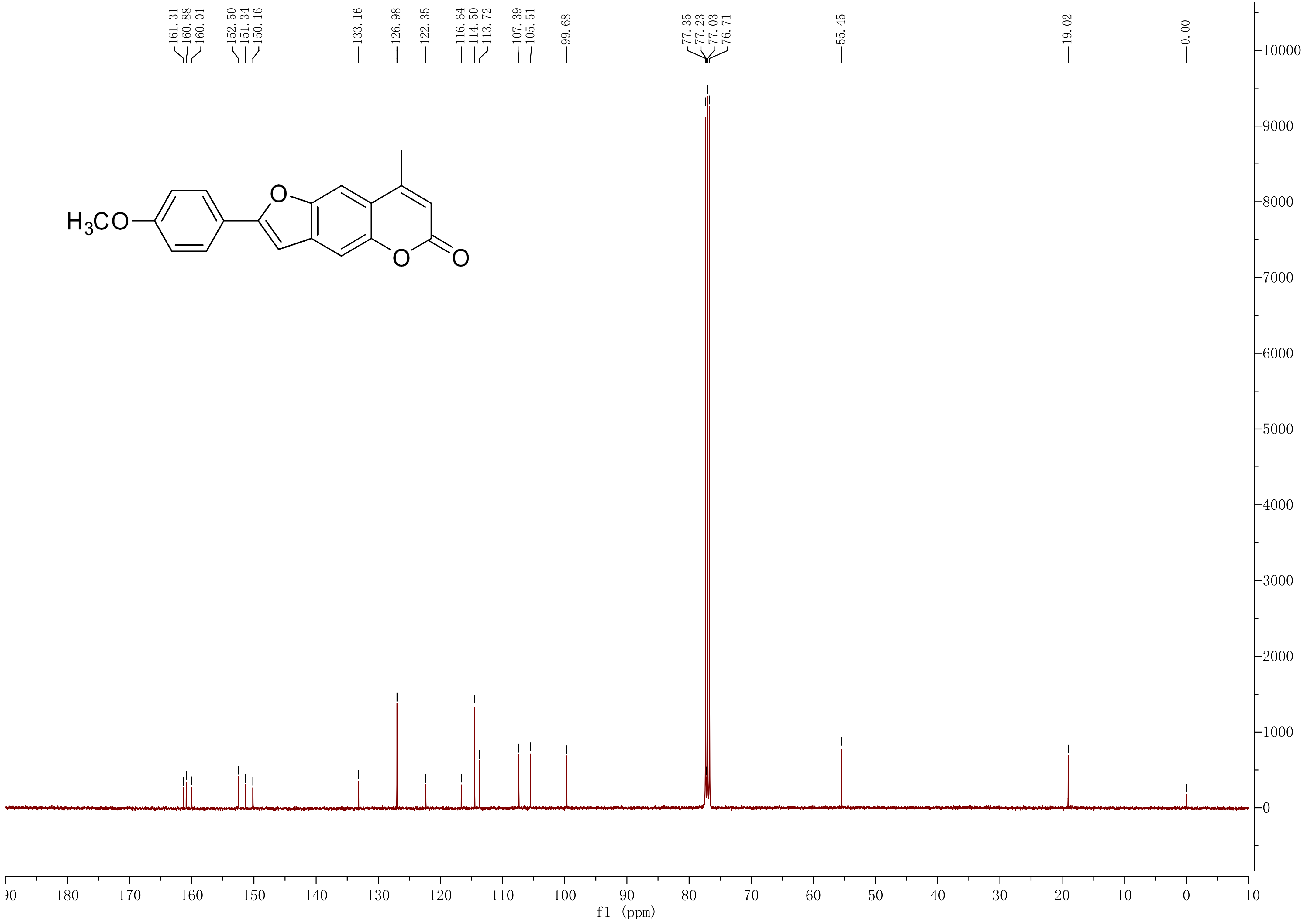

161.31
160.88
160.01
152.50
151.34
150.16
133.16
126.98
122.35
116.64
114.50
113.72
107.39
105.51
99.68
77.35
77.23
77.03
76.71
55.45
19.02
0.00
H3CO
O
O
O
f1 (ppm)

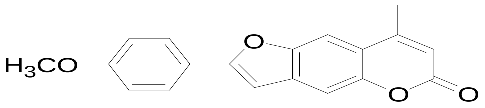

H3CO
O
O
O

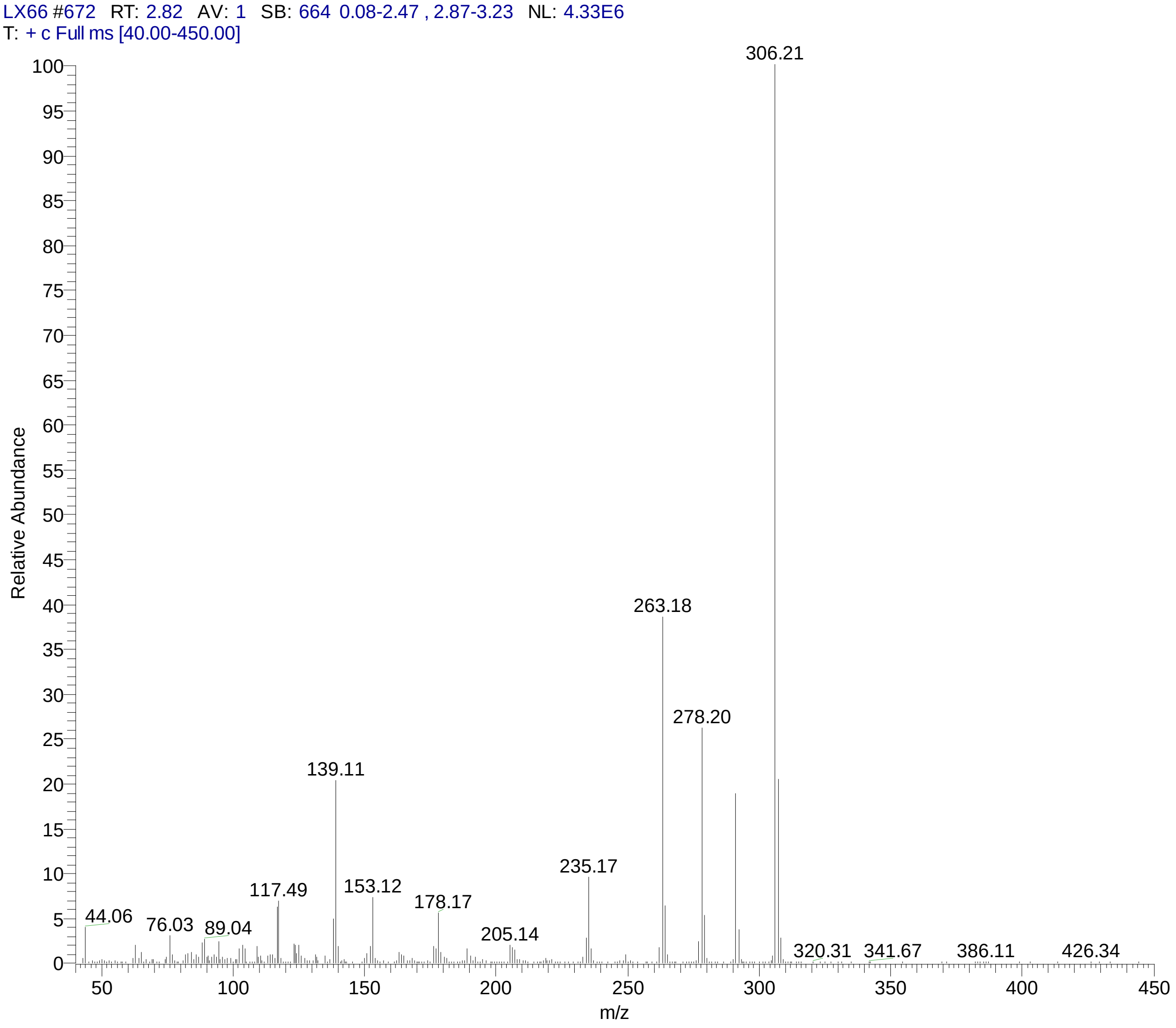

LX66 #672 RT: 2.82 AV: 1 SB: 664 0.08-2.47 , 2.87-3.23 NL: 4.33E6
T: + c Full ms [40.00-450.00]
Relative Abundance
m/z
306.21
263.18
278.20
139.11
235.17
117.49
153.12
178.17
44.06
76.03
89.04
205.14
320.31
341.67
386.11
426.34

MS of I32

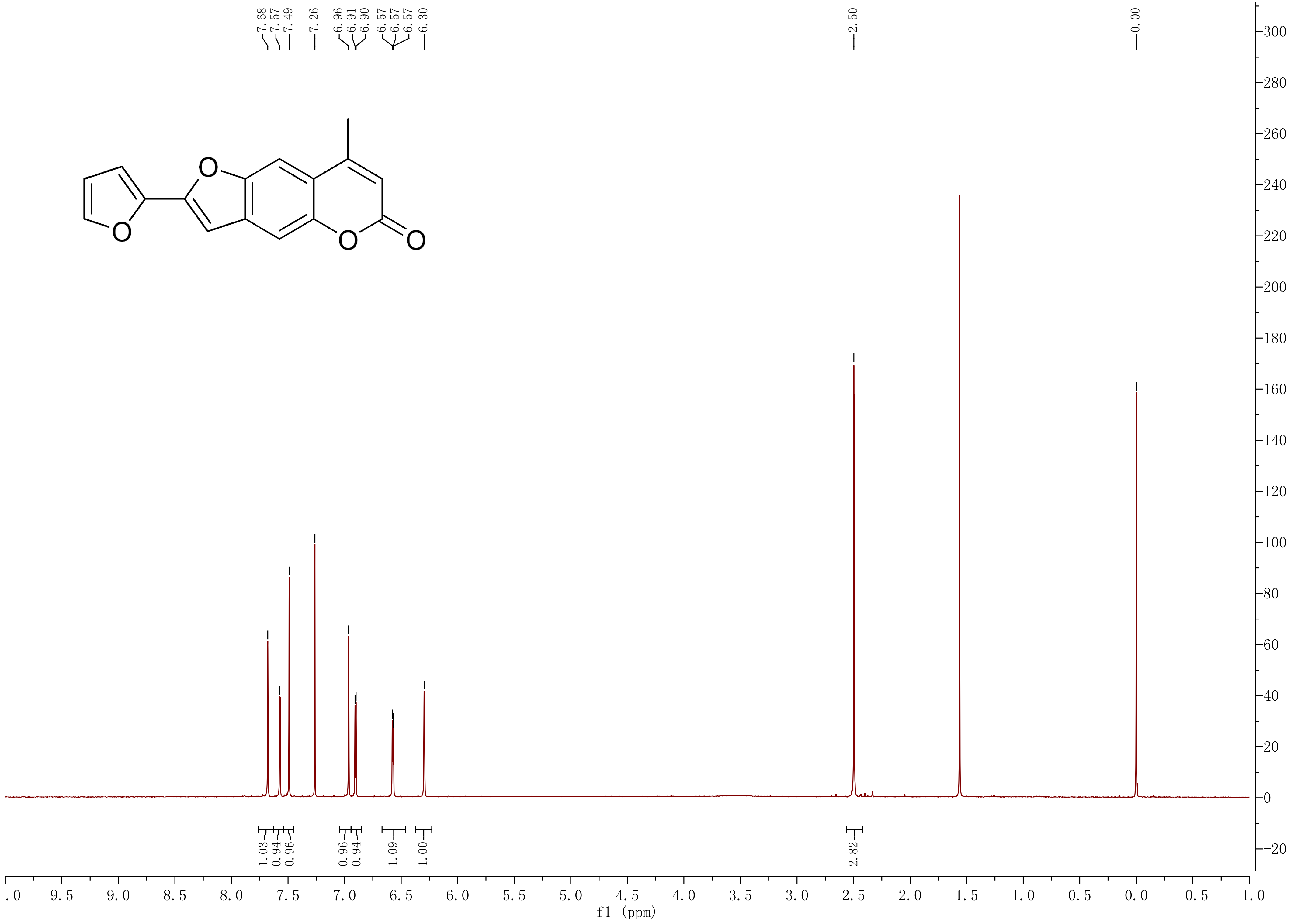
7.68
7.57
7.49
7.26
6.96
6.91
6.90
6.57
6.57
6.57
6.30
2.50
0.00
1.03
0.94
0.96
0.96
0.94
1.09
1.00
2.82
f1 (ppm)

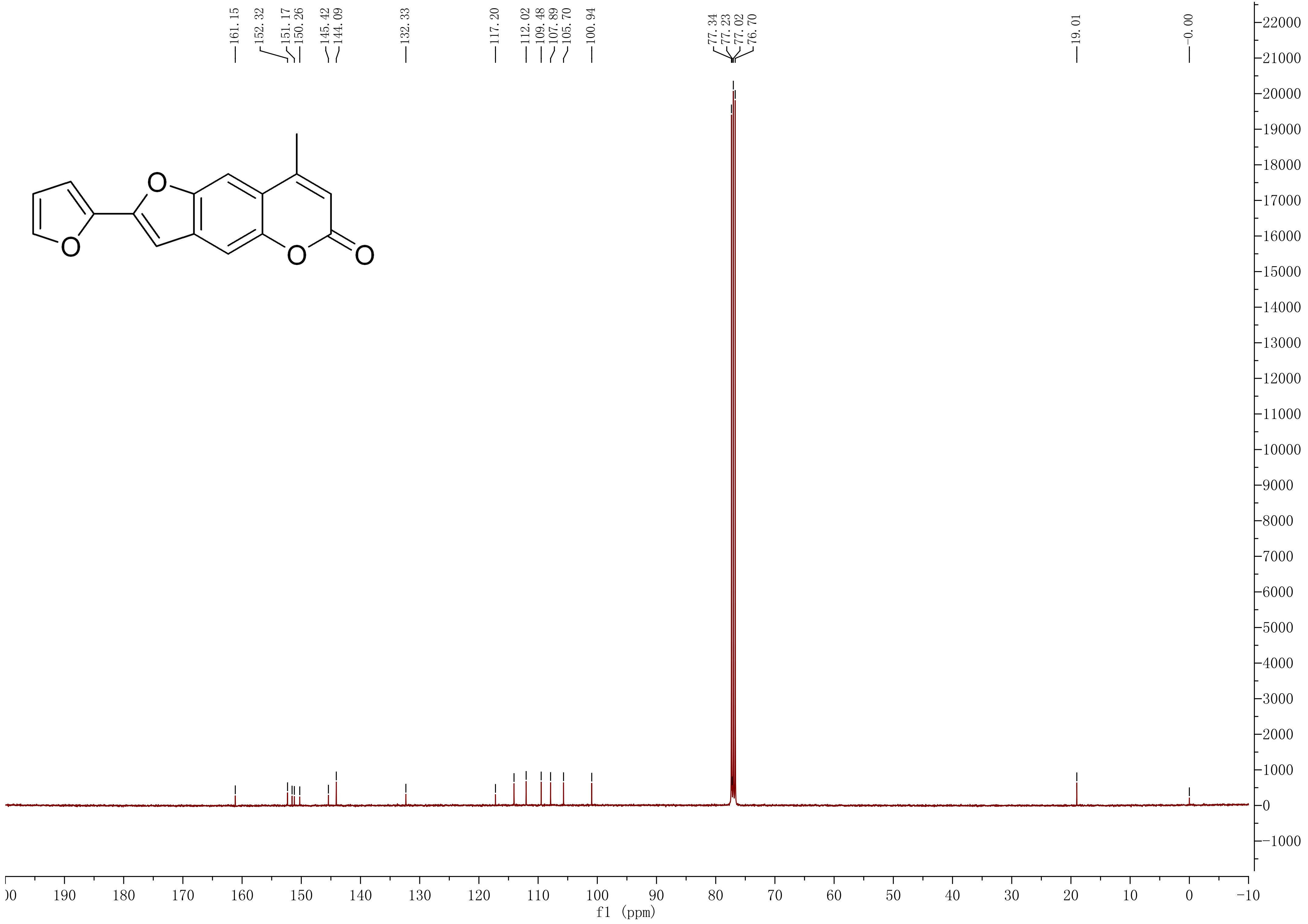

161.15
152.32
151.17
150.26
145.42
144.09
132.33
117.20
112.02
109.48
107.89
105.70
100.94
77.34
77.23
77.02
76.70
19.01
-0.00
O
O
O
O
f1 (ppm)

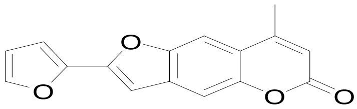

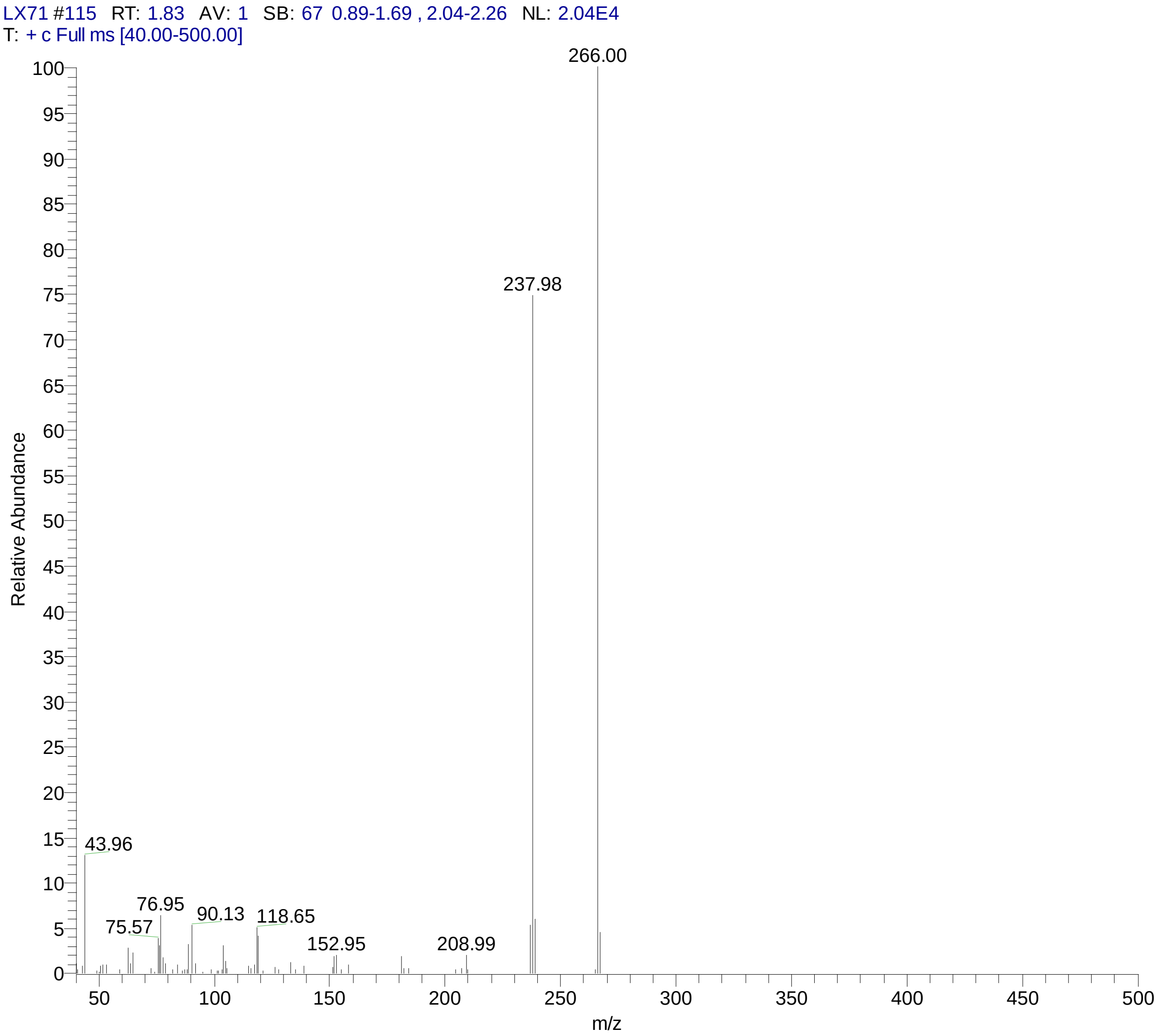
LX71 #115 RT: 1.83 AV: 1 SB: 67 0.89-1.69 , 2.04-2.26 NL: 2.04E4
T: + c Full ms [40.00-500.00]
266.00
237.98
43.96
76.95
75.57
90.13
118.65
152.95
208.99
Relative Abundance
m/z

MS of I33

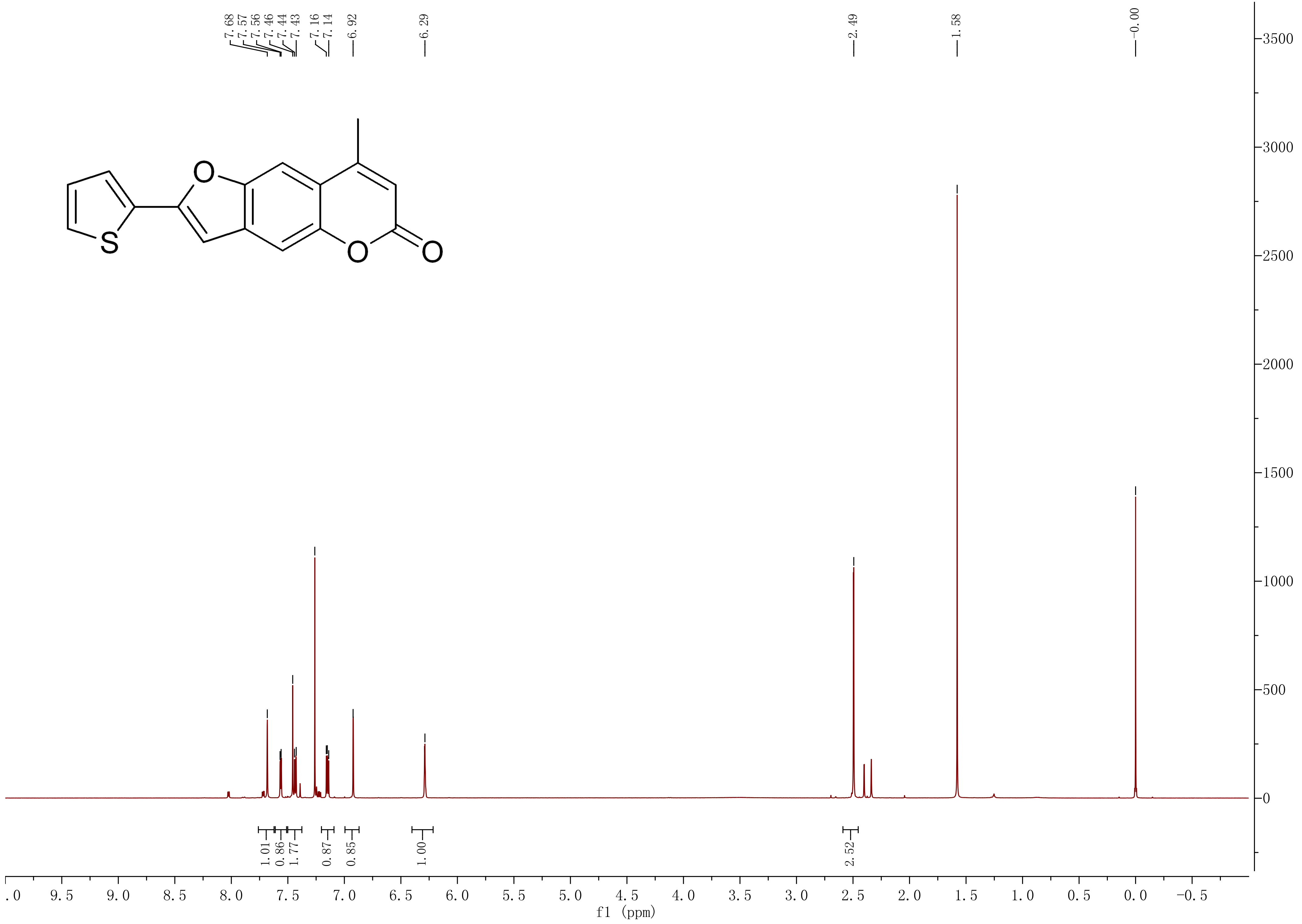

7.68
7.57
7.56
7.46
7.44
7.43
7.16
7.14
6.92
6.29
2.49
1.58
-0.00
3500
3000
2500
2000
1500
1000
500
0
O
O
O
S
1.01
0.86
1.77
0.87
0.85
1.00
2.52
.0
9.5
9.0
8.5
8.0
7.5
7.0
6.5
6.0
5.5
5.0
4.5
4.0
3.5
3.0
2.5
2.0
1.5
1.0
0.5
0.0
-0.5
f1 (ppm)

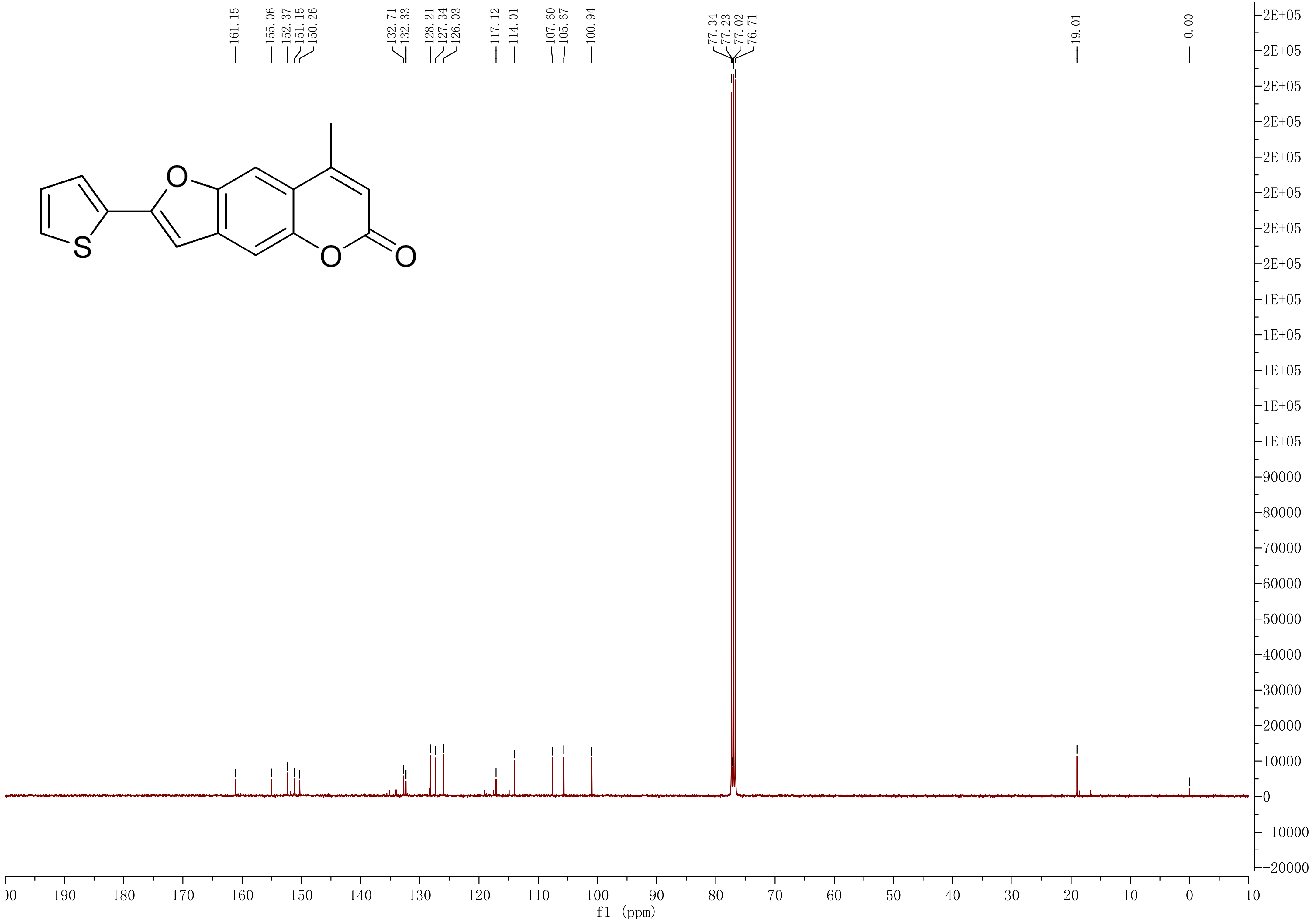
161.15
155.06
152.37
151.15
150.26
132.71
132.33
128.21
127.34
126.03
117.12
114.01
107.60
105.67
100.94
77.34
77.23
77.02
76.71
19.01
-0.00
O
O
O
S
f1 (ppm)
190
180
170
160
150
140
130
120
110
100
90
80
70
60
50
40
30
20
10
0
-10
2E+05
1E+05
90000
80000
70000
60000
50000
40000
30000
20000
10000
0
-10000
-20000

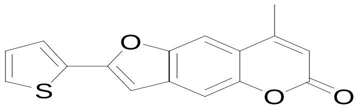

O
S
O
O

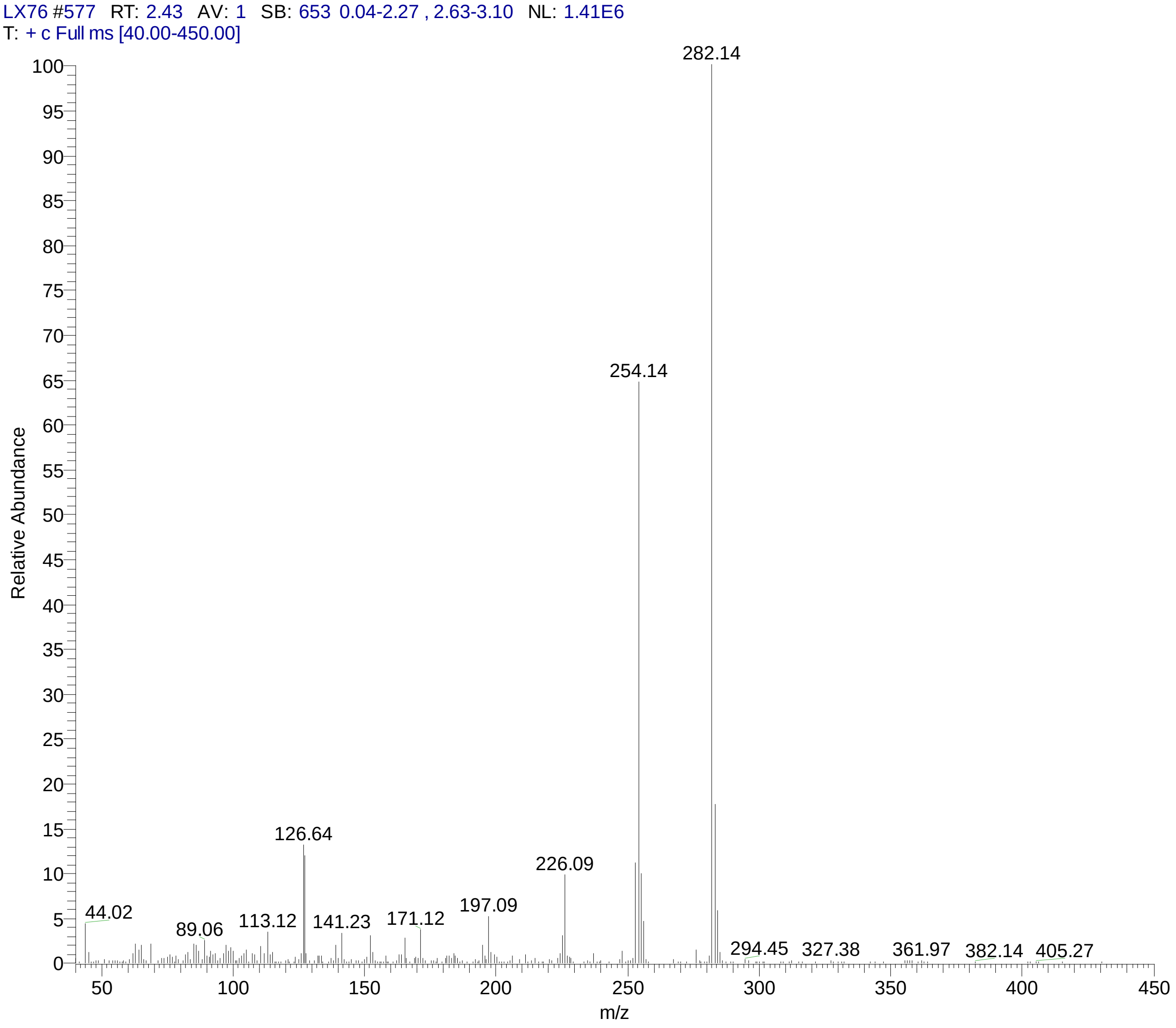

LX76 #577 RT: 2.43 AV: 1 SB: 653 0.04-2.27 , 2.63-3.10 NL: 1.41E6
T: + c Full ms [40.00-450.00]
Relative Abundance
m/z
282.14
254.14
126.64
226.09
44.02
89.06
113.12
141.23
171.12
197.09
294.45
327.38
361.97
382.14
405.27

MS of I34

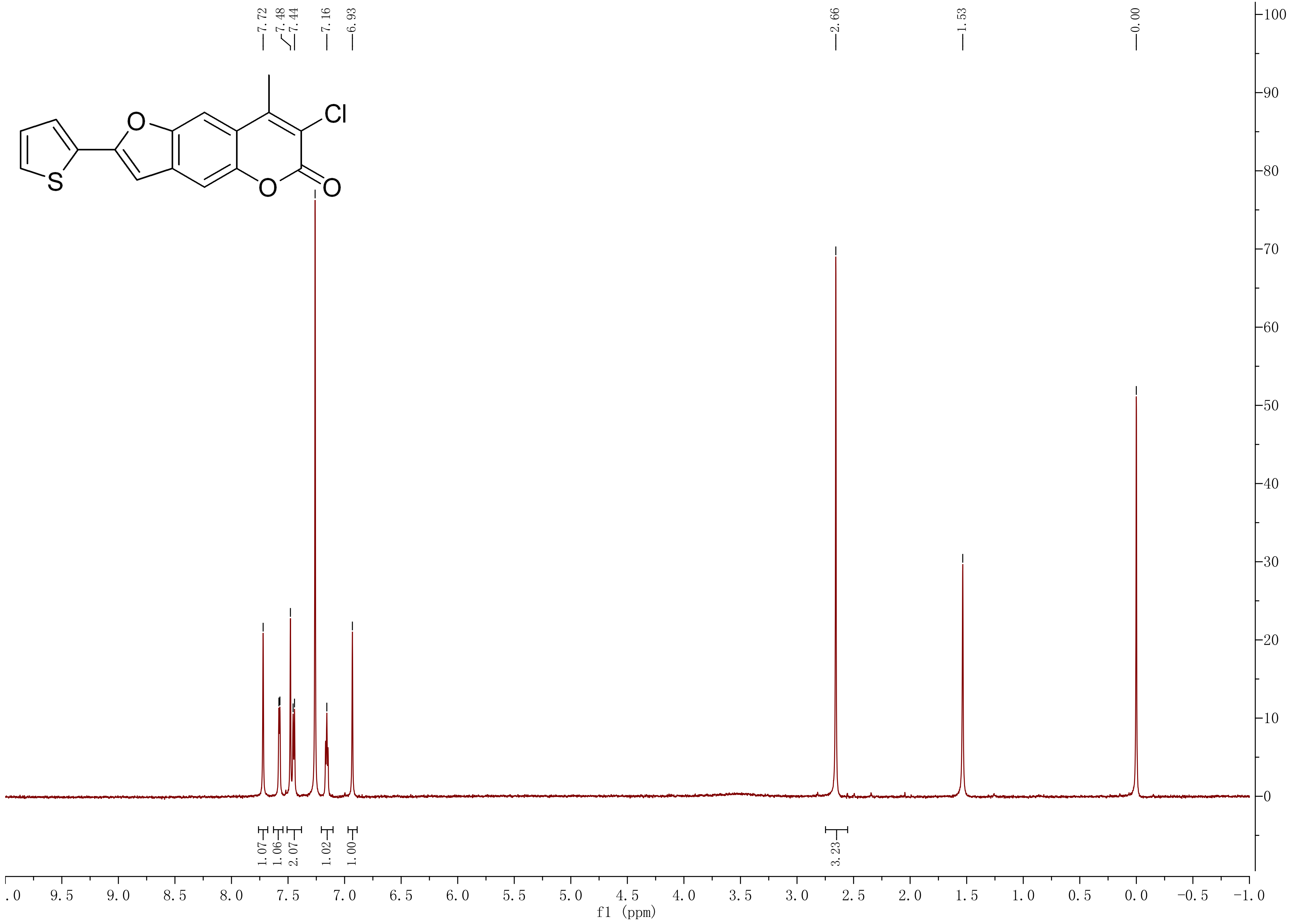

7.72
7.48
7.44
7.16
6.93
2.66
1.53
0.00
Cl
O
O
O
S
1.07
1.06
2.07
1.02
1.00
3.23
f1 (ppm)

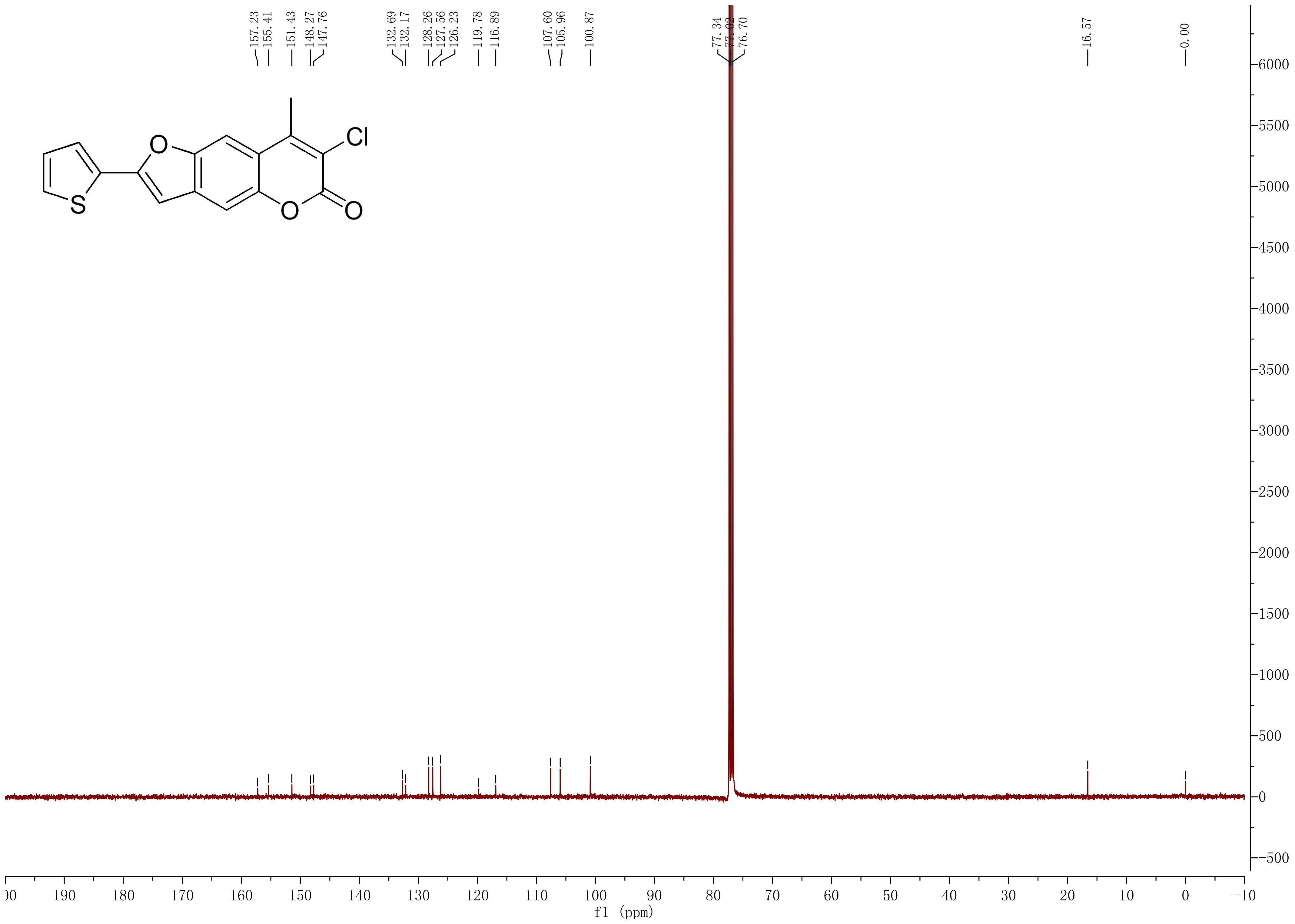
157.23
155.41
151.43
148.27
147.76
132.69
132.17
128.26
127.56
126.23
119.78
116.89
107.60
105.96
100.87
77.34
77.02
76.70
16.57
0.00
Cl
O
S
f1 (ppm)

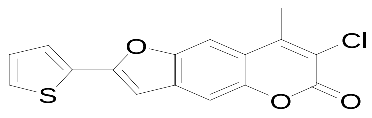
Cl
O
S
O
O

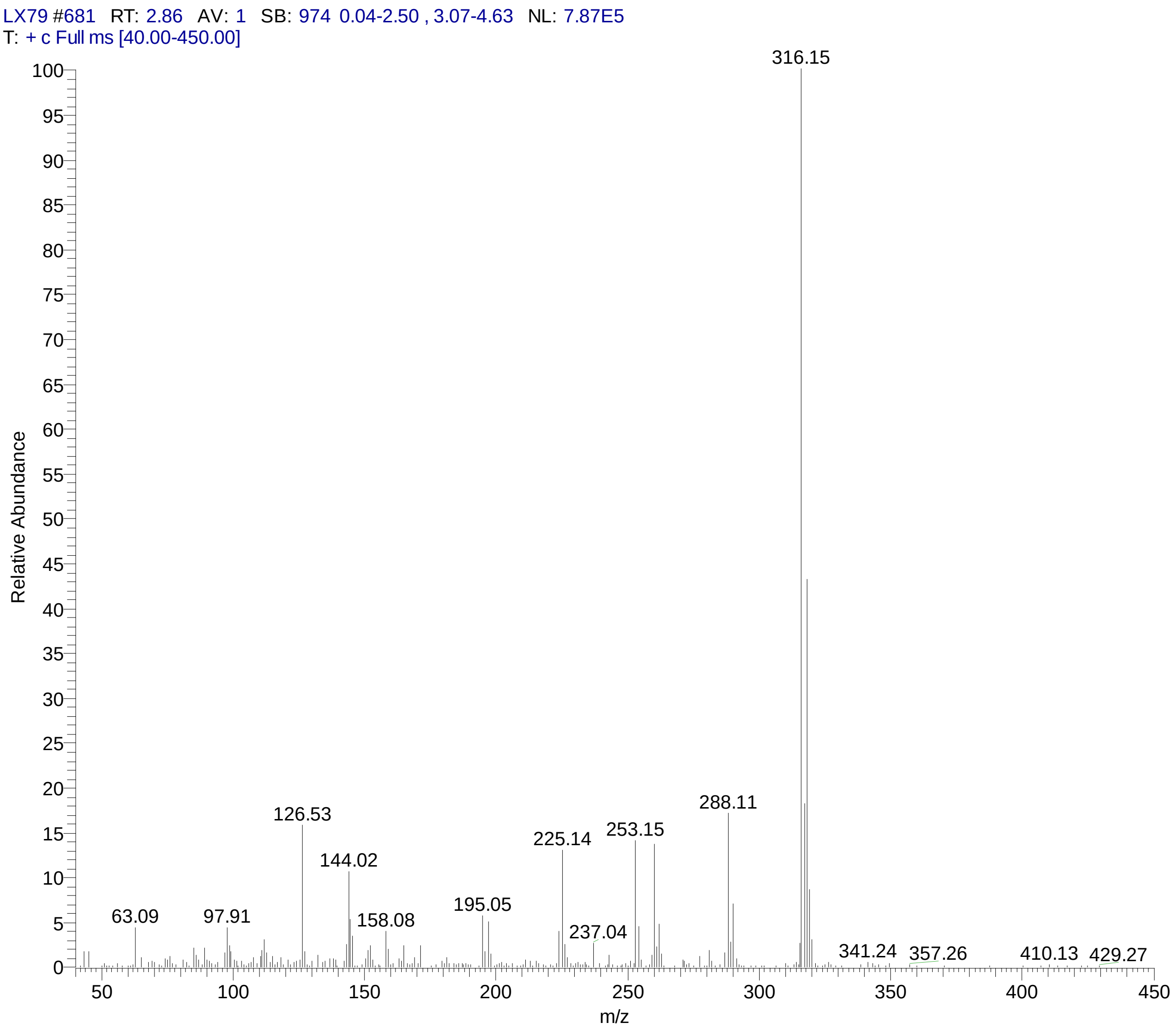
LX79 #681 RT: 2.86 AV: 1 SB: 974 0.04-2.50 , 3.07-4.63 NL: 7.87E5
T: + c Full ms [40.00-450.00]
316.15
288.11
126.53
253.15
225.14
144.02
195.05
158.08
237.04
63.09
97.91
341.24
357.26
410.13
429.27
Relative Abundance
m/z

MS of I35

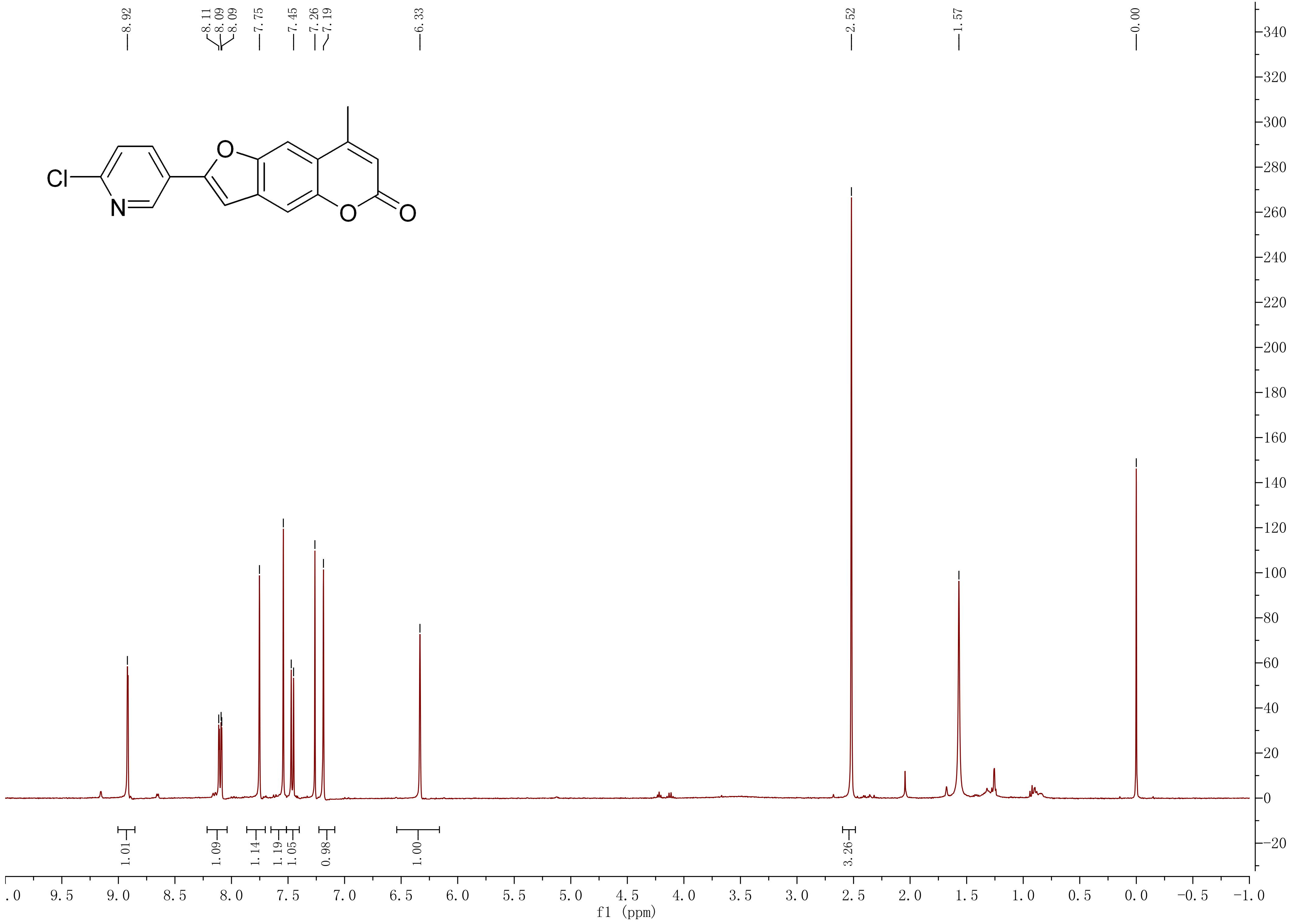
8.92
8.11
8.09
8.09
7.75
7.45
7.26
7.19
6.33
2.52
1.57
0.00
Cl
N
O
O
O
1.01
1.09
1.14
1.19
1.05
0.98
1.00
3.26
f1 (ppm)

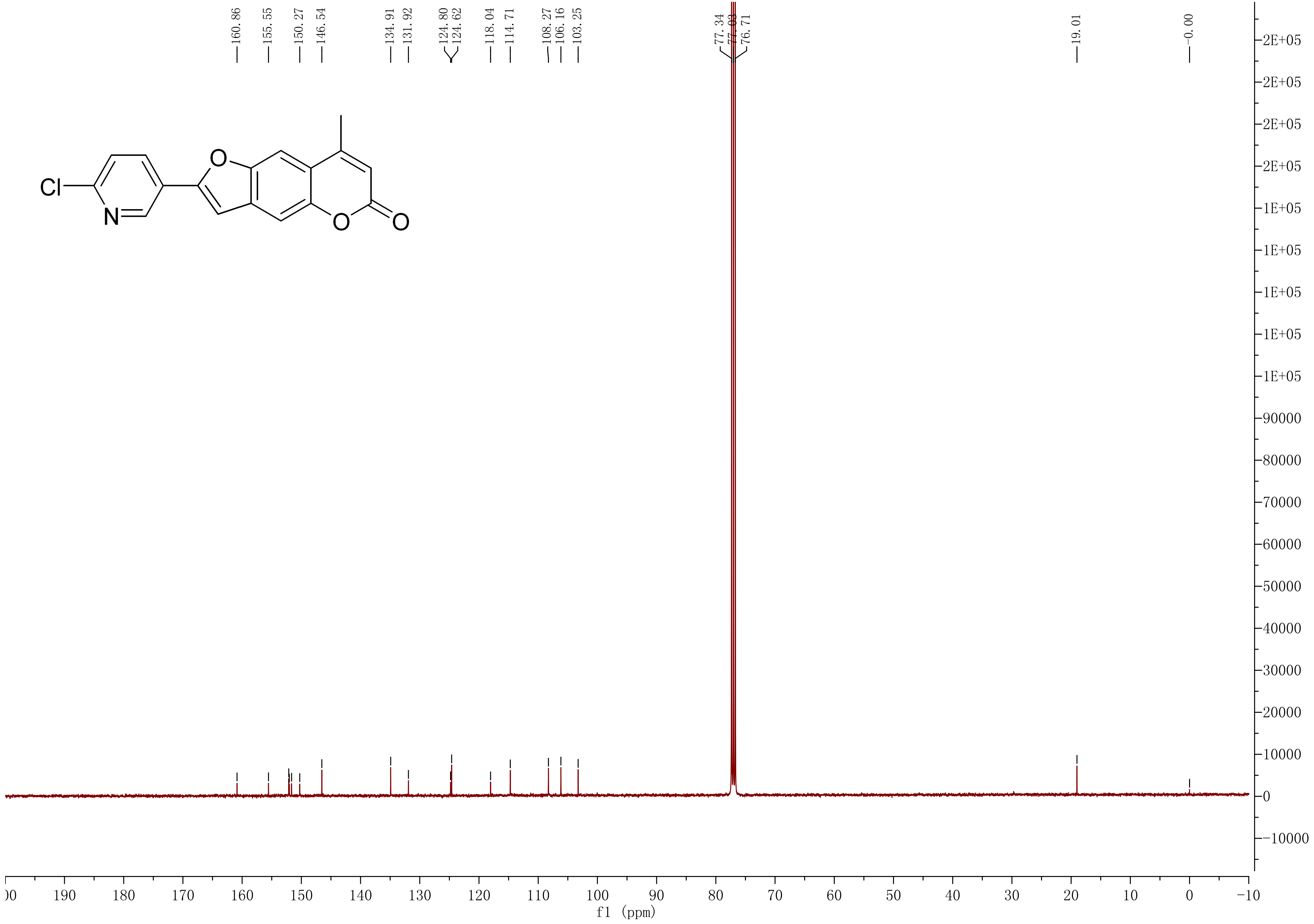
160.86
155.55
150.27
146.54
134.91
131.92
124.80
124.62
118.04
114.71
108.27
106.16
103.25
77.34
77.03
76.71
19.01
-0.00
Cl
N
O
O
O
f1 (ppm)

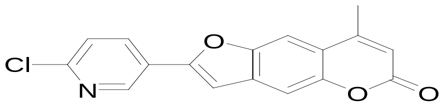
Cl
N
O
O
O

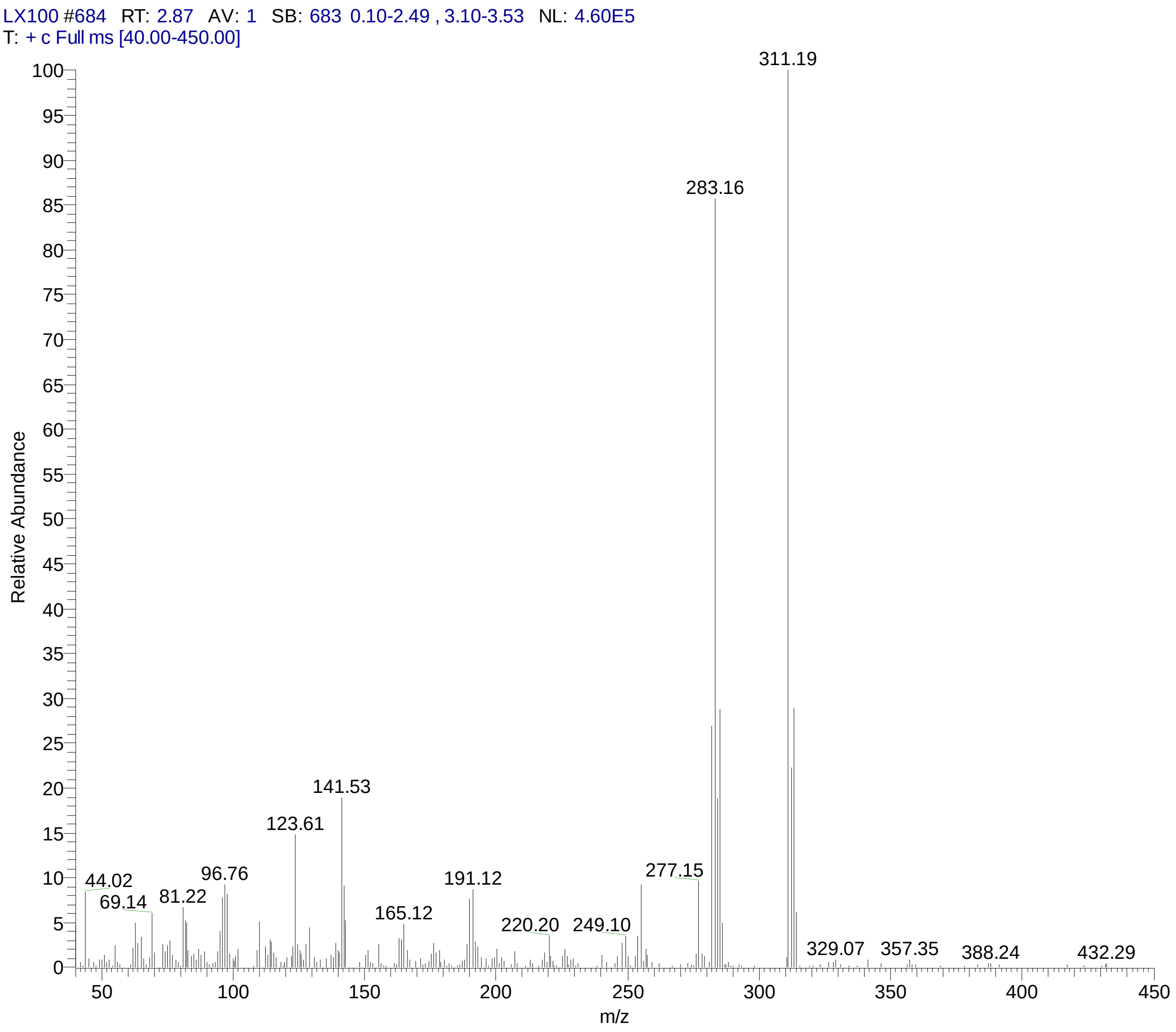
LX100 #684 RT: 2.87 AV: 1 SB: 683 0.10-2.49 , 3.10-3.53 NL: 4.60E5
T: + c Full ms [40.00-450.00]
Relative Abundance
m/z
311.19
283.16
141.53
123.61
44.02
69.14
81.22
96.76
165.12
191.12
220.20
249.10
277.15
329.07
357.35
388.24
432.29

MS of I36

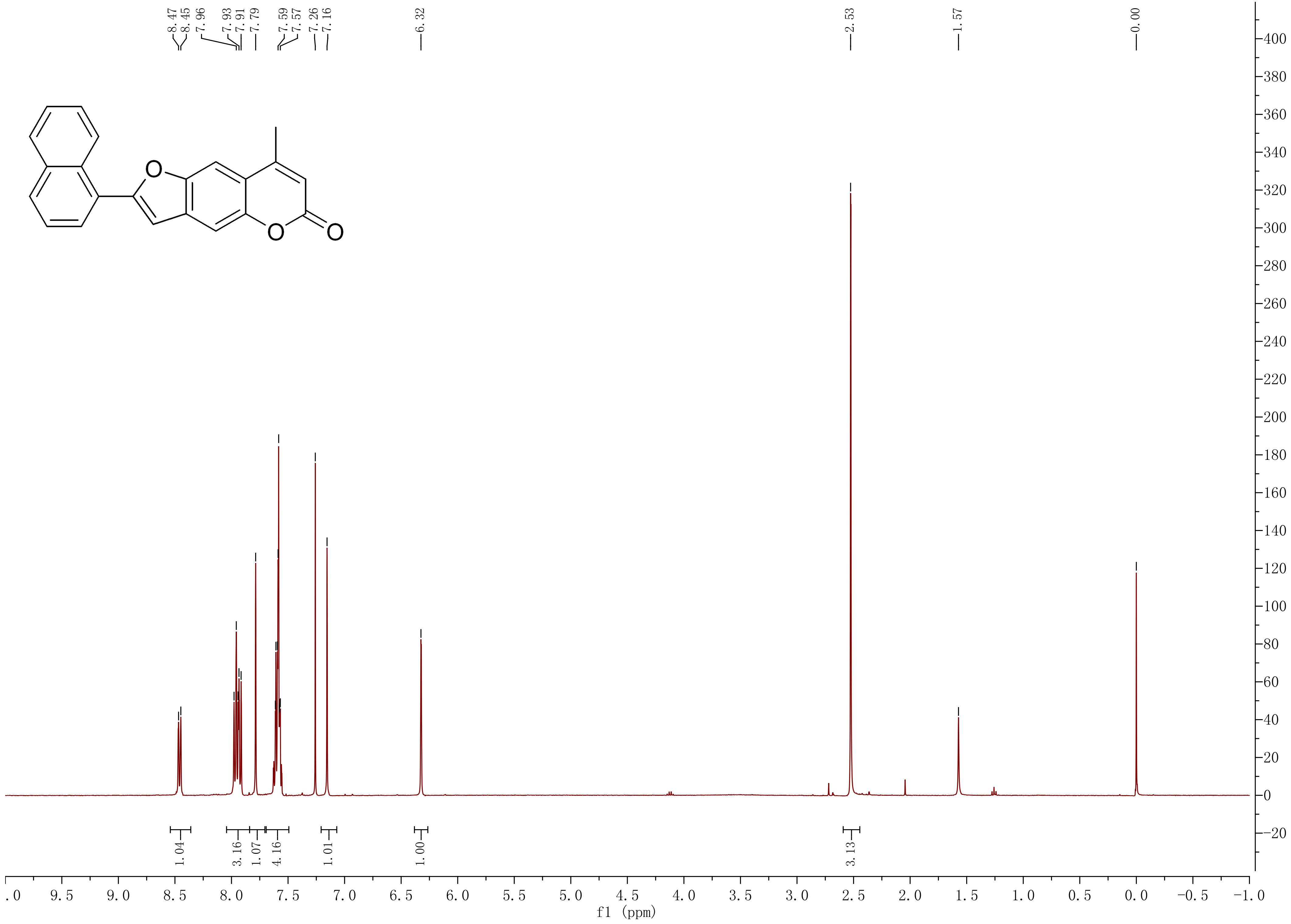
8.47
8.45
7.96
7.93
7.91
7.79
7.59
7.57
7.26
7.16
6.32
2.53
1.57
0.00
1.04
3.16
1.07
4.16
1.01
1.00
3.13
f1 (ppm)

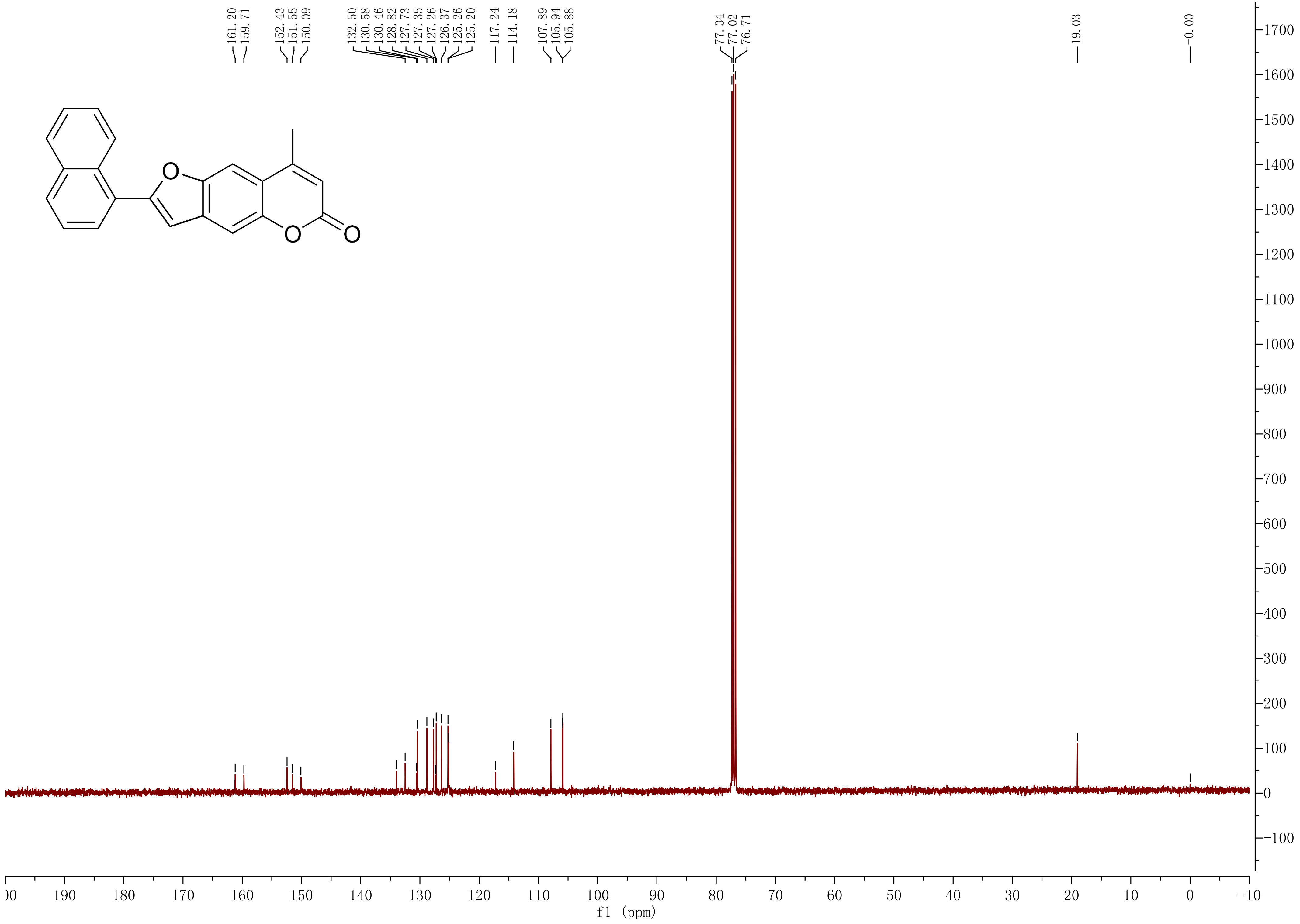

161.20
159.71
152.43
151.55
150.09
132.50
130.58
130.46
128.82
127.73
127.35
127.26
126.37
125.26
125.20
117.24
114.18
107.89
105.94
105.88
77.34
77.02
76.71
19.03
-0.00
O
O
O
f1 (ppm)

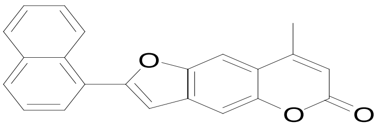

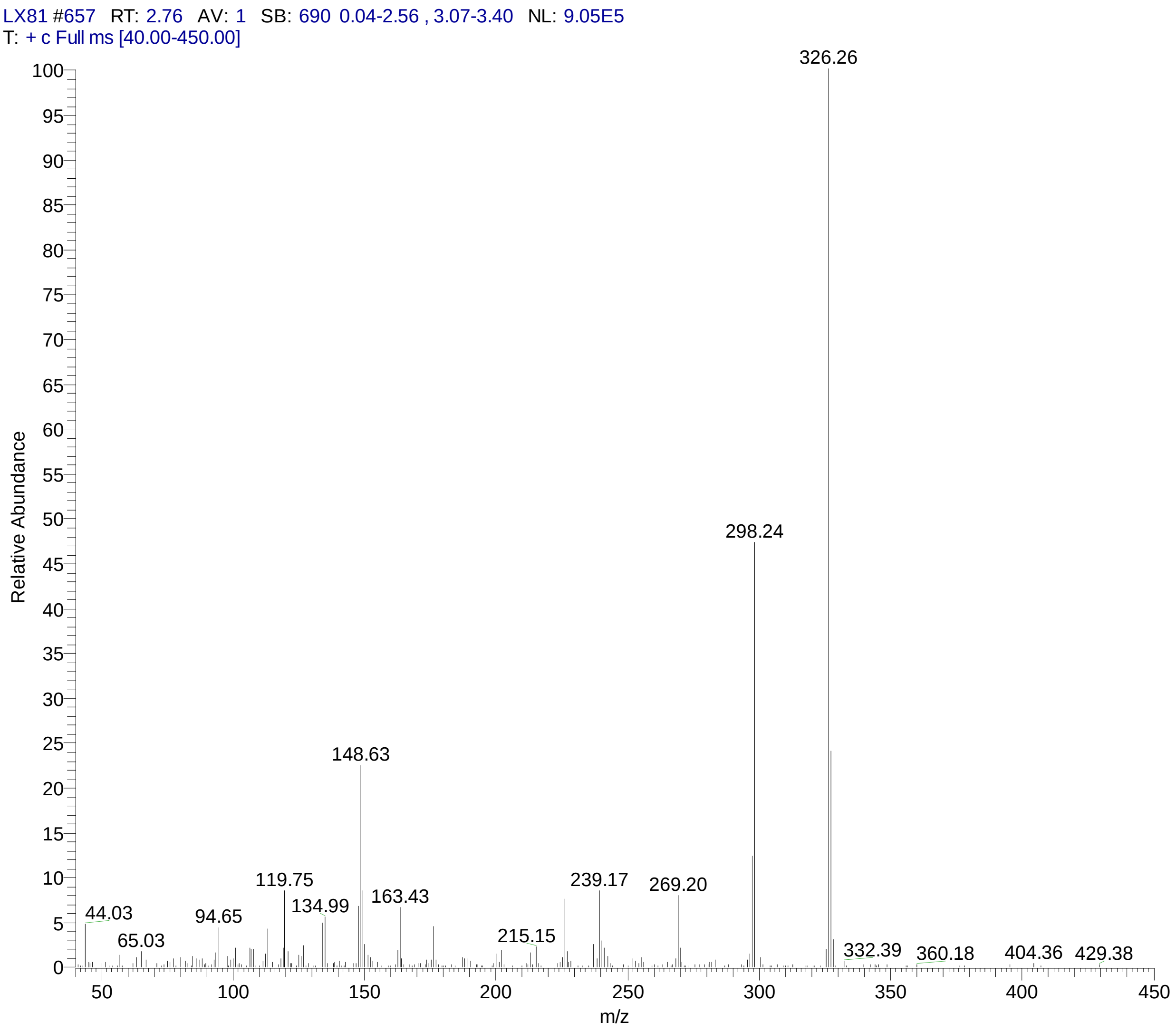
LX81 #657 RT: 2.76 AV: 1 SB: 690 0.04-2.56 , 3.07-3.40 NL: 9.05E5
T: + c Full ms [40.00-450.00]
Relative Abundance
m/z
326.26
298.24
148.63
119.75
134.99
163.43
239.17
269.20
215.15
94.65
44.03
65.03
332.39
360.18
404.36
429.38

MS of I37

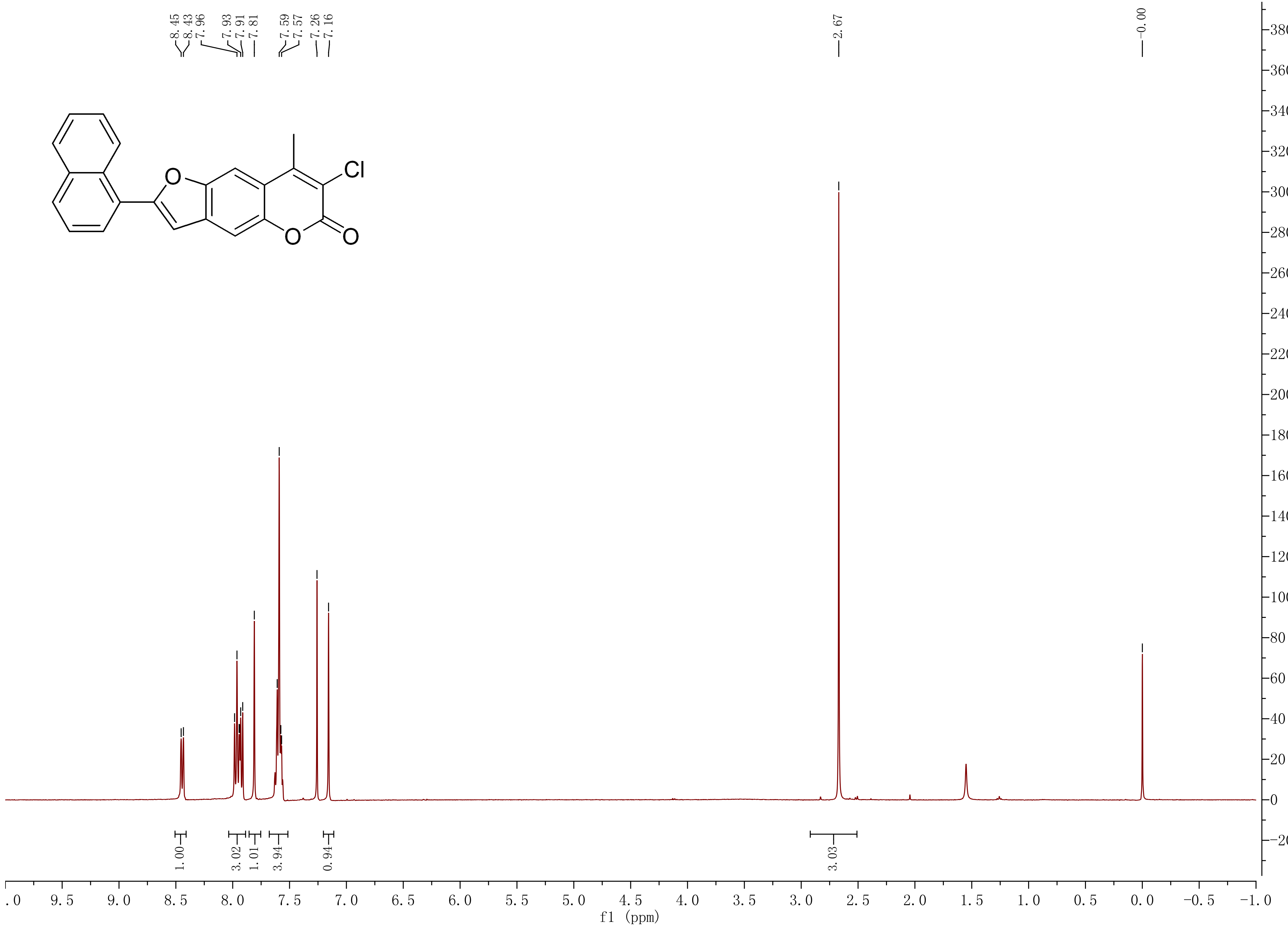

8.45
8.43
7.96
7.93
7.91
7.81
7.59
7.57
7.26
7.16
2.67
0.00
Cl
O
1.00
3.02
1.01
3.94
0.94
3.03
f1 (ppm)

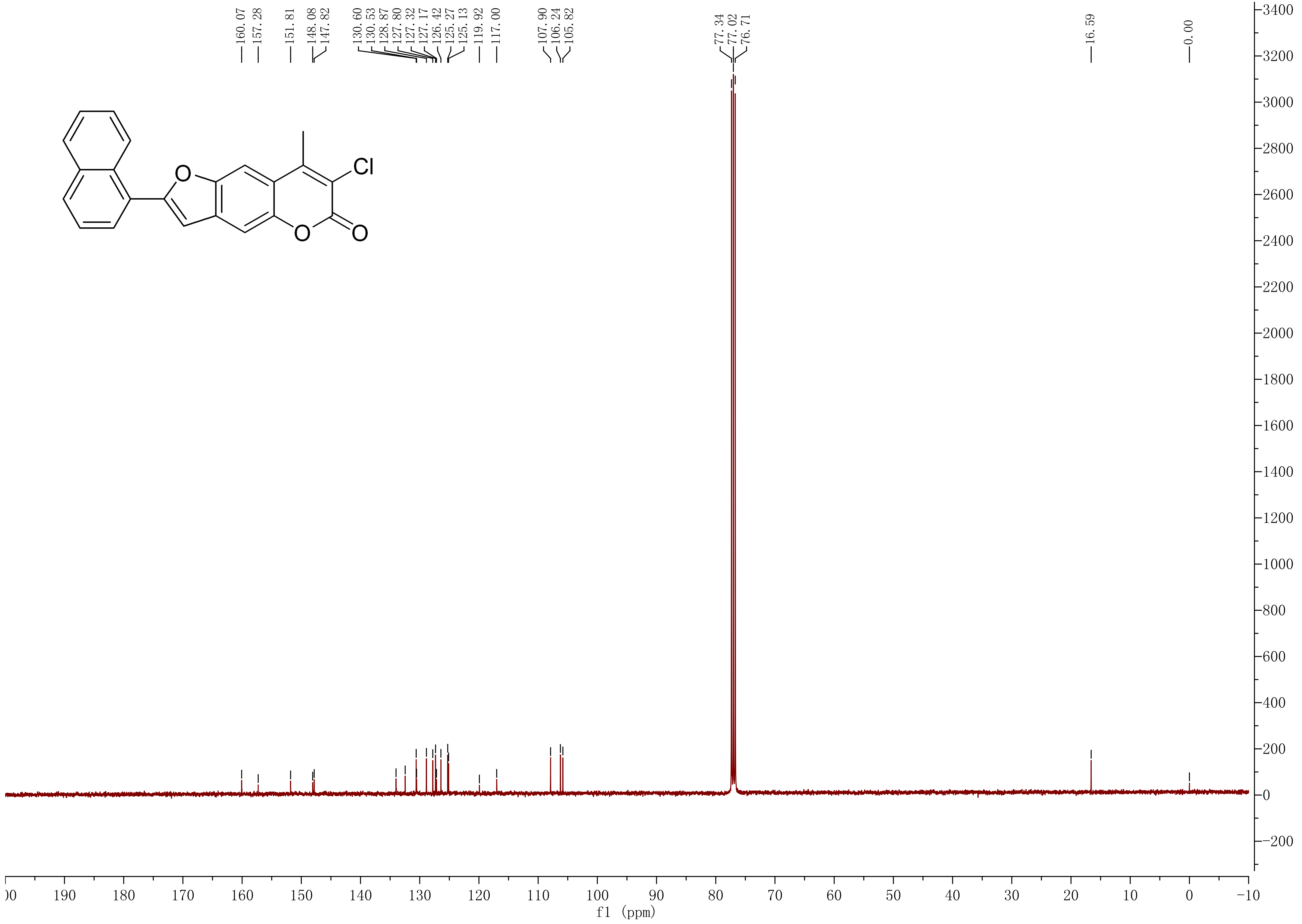

160.07
157.28
151.81
148.08
147.82
130.60
130.53
128.87
127.80
127.32
127.17
126.42
125.27
125.13
119.92
117.00
107.90
106.24
105.82
77.34
77.02
76.71
16.59
0.00
Cl
O
O
O
f1 (ppm)

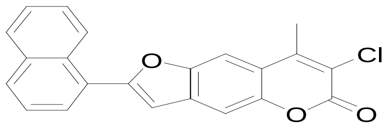
O
Cl
O
O

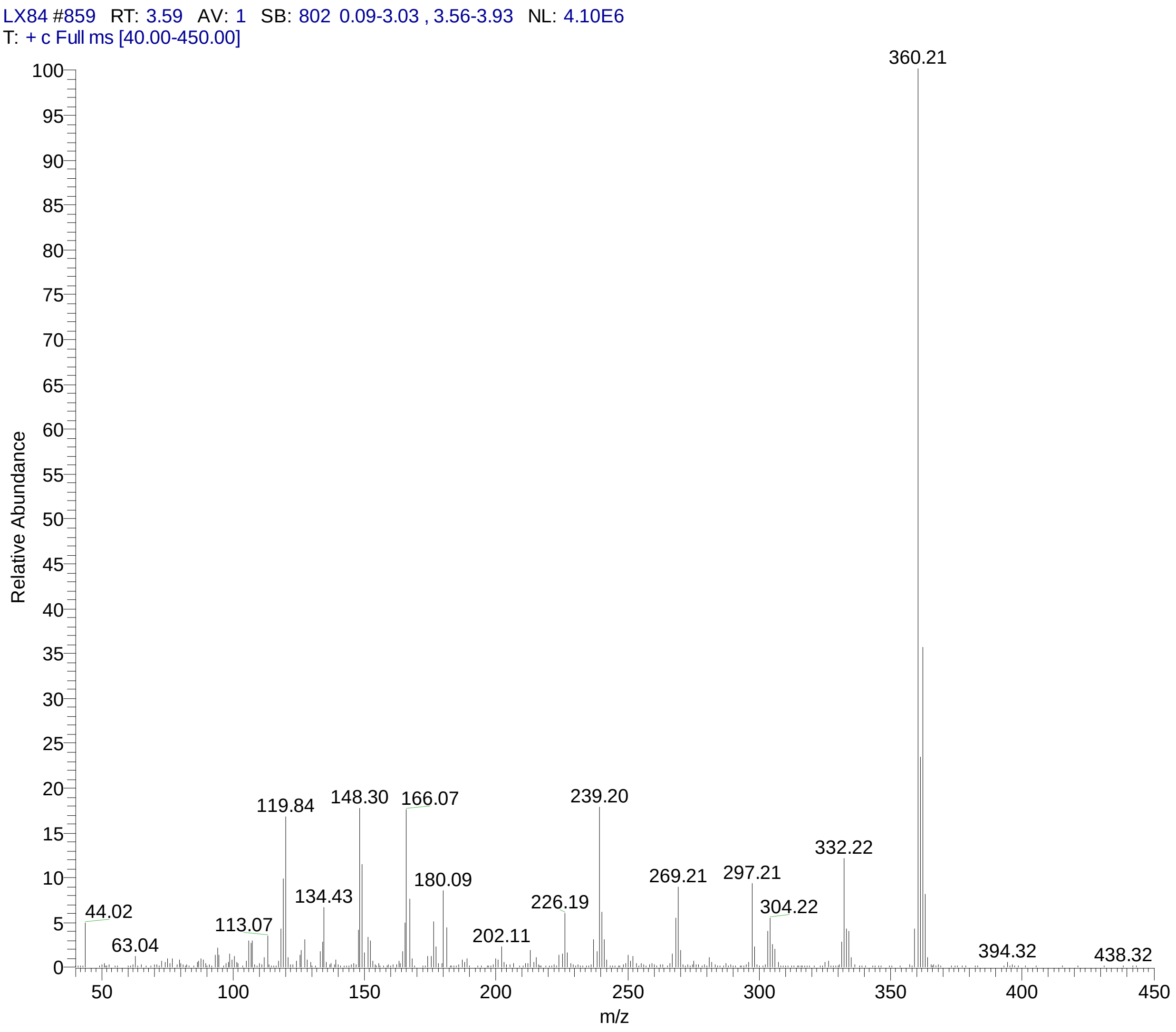
LX84 #859 RT: 3.59 AV: 1 SB: 802 0.09-3.03 , 3.56-3.93 NL: 4.10E6
T: + c Full ms [40.00-450.00]
360.21
119.84
148.30
166.07
239.20
332.22
269.21
297.21
180.09
134.43
226.19
304.22
44.02
113.07
202.11
63.04
394.32
438.32
Relative Abundance
m/z

MS of I38

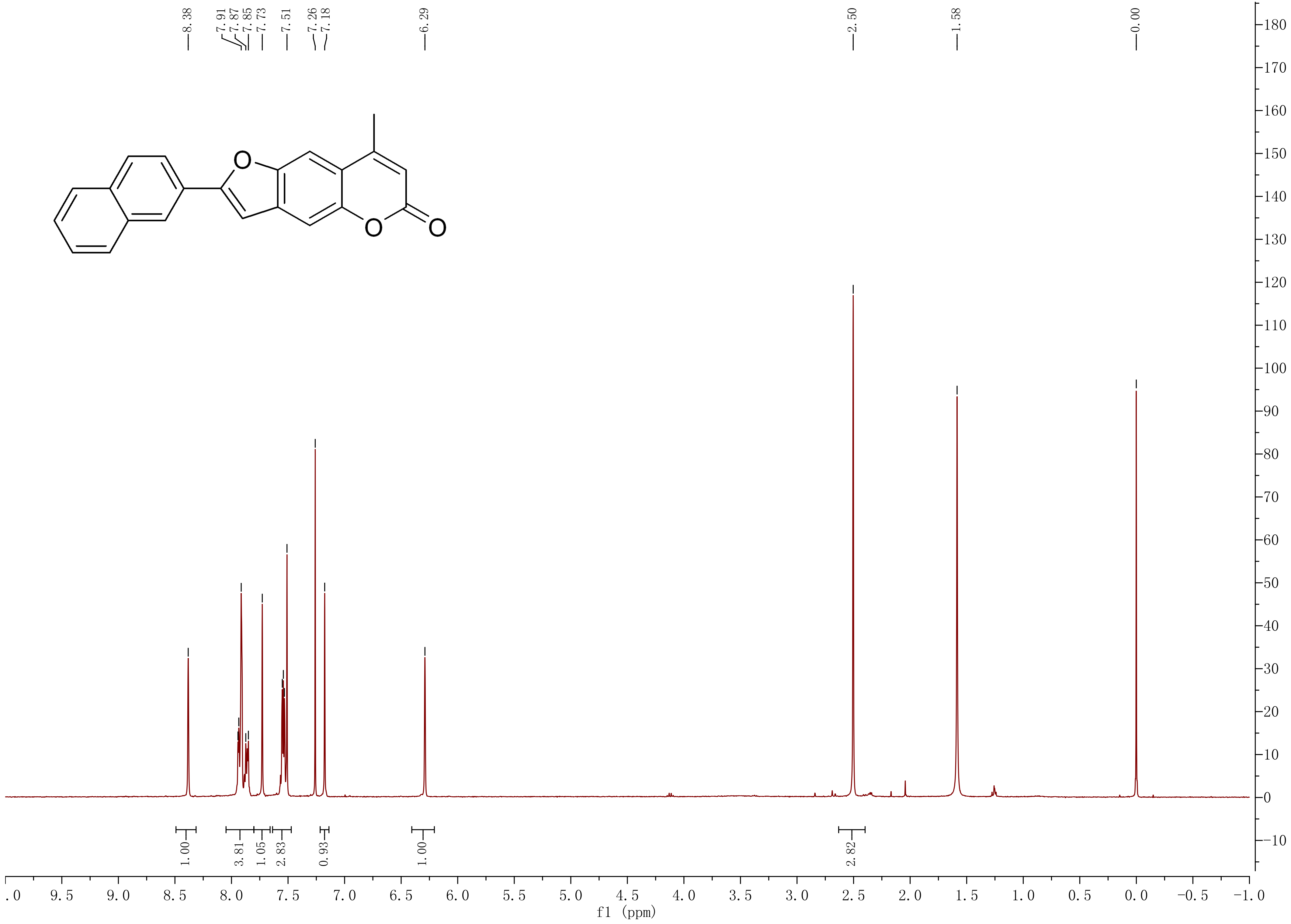
8.38
7.91
7.87
7.85
7.73
7.51
7.26
7.18
6.29
2.50
1.58
0.00
1.00
3.81
1.05
2.83
0.93
1.00
2.82
f1 (ppm)

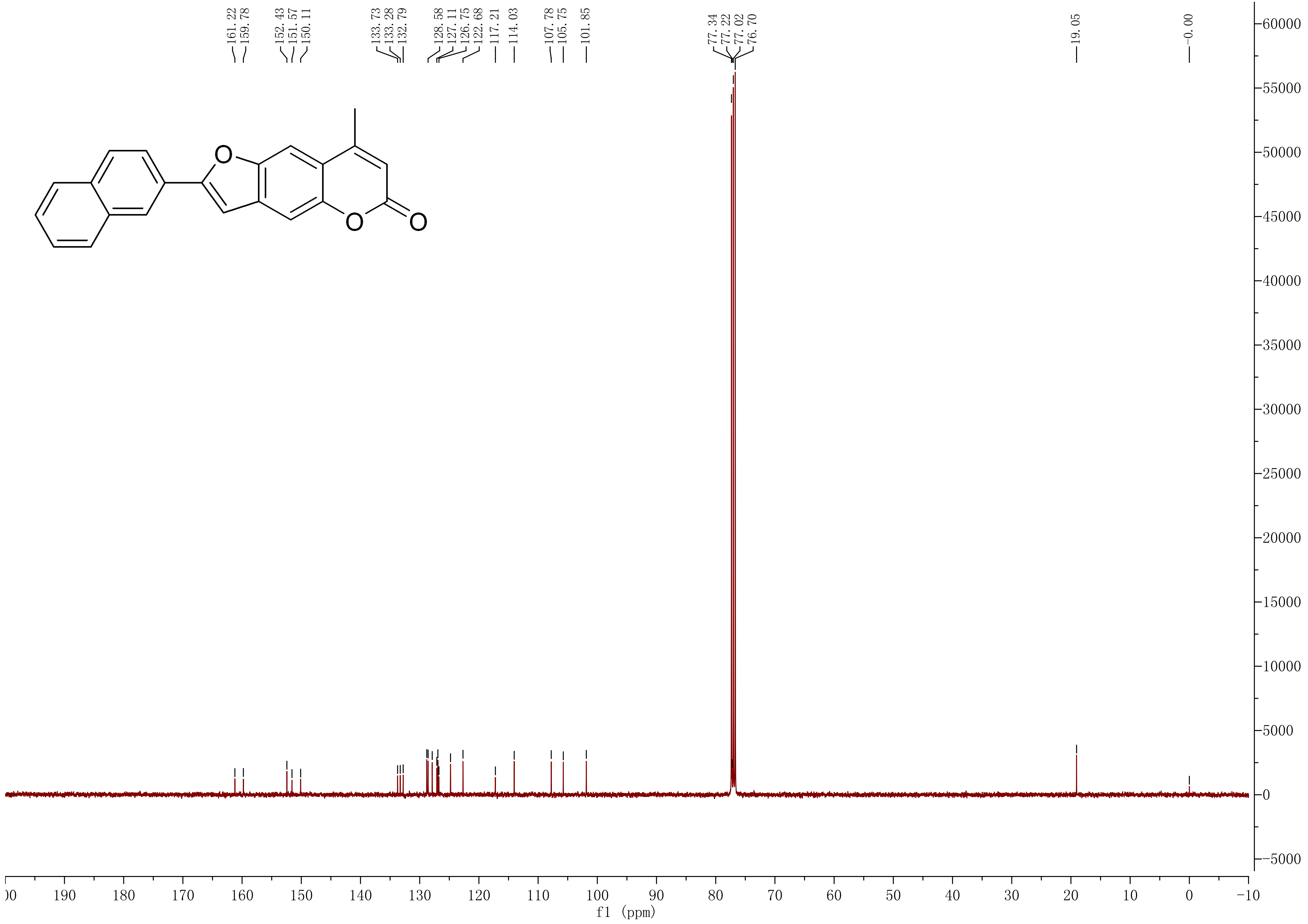
161.22
159.78
152.43
151.57
150.11
133.73
133.28
132.79
128.58
127.11
126.75
122.68
117.21
114.03
107.78
105.75
101.85
77.34
77.22
77.02
76.70
19.05
-0.00
f1 (ppm)

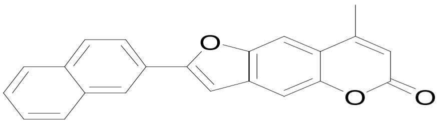
O
O
O

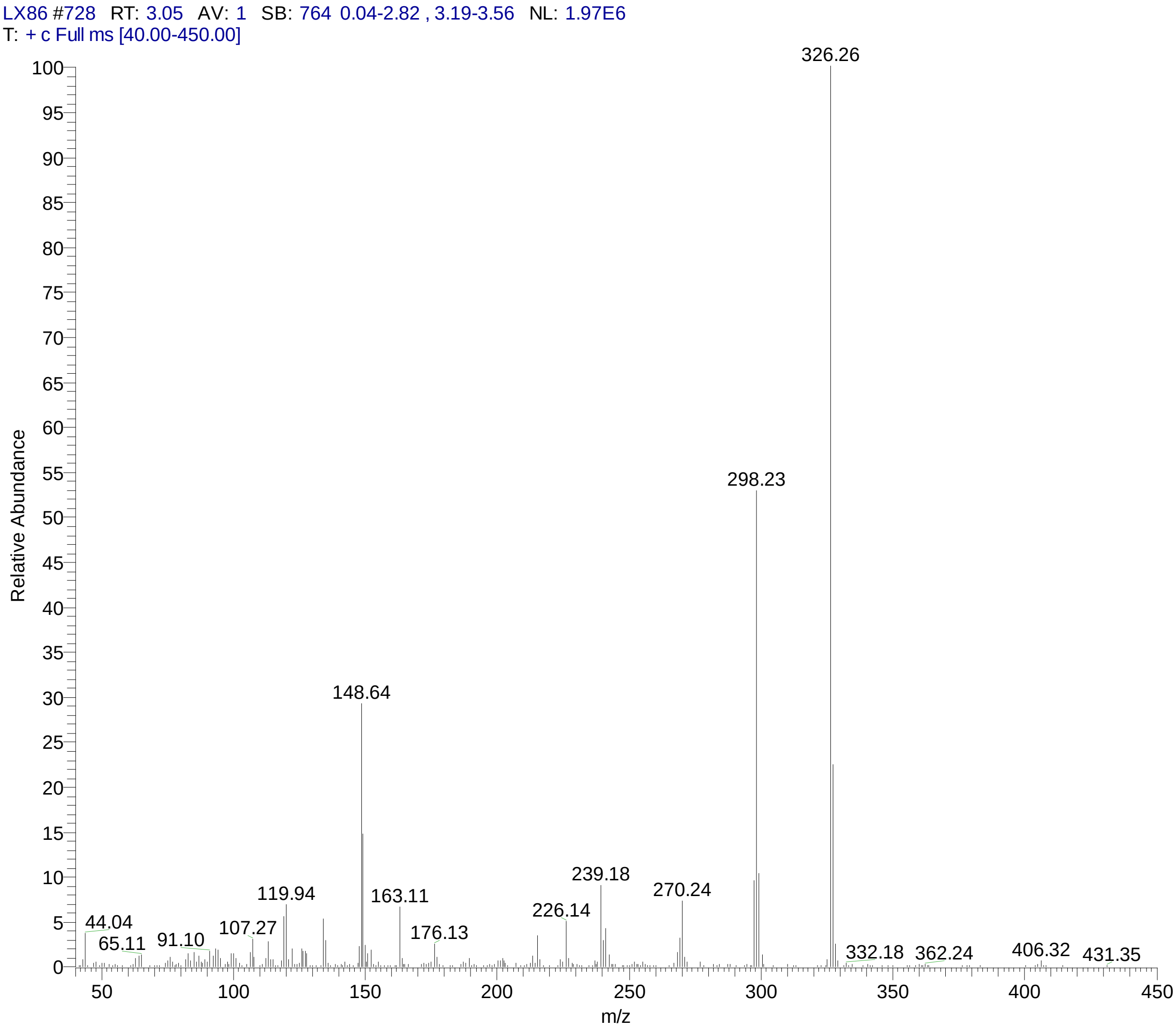
LX86 #728 RT: 3.05 AV: 1 SB: 764 0.04-2.82 , 3.19-3.56 NL: 1.97E6
T: + c Full ms [40.00-450.00]
326.26
298.23
148.64
119.94
163.11
176.13
226.14
239.18
270.24
44.04
65.11
91.10
107.27
332.18
362.24
406.32
431.35
Relative Abundance
m/z

MS of I39
