# Supplementary material for: Potential Biochemical Pesticide—Synthesis of Neofuranocoumarin and Inhibition the Proliferation of Spodoptera frugiperda Cells through Activating the Mitochondrial Pathway
Source: Toxins (Basel). 2022 Sep 29;14(10):677. doi: 10.3390/toxins14100677 (PMC9612269; doi:10.3390/toxins14100677)
Supplement: Supplementary file 1 [file toxins-14-00677-s001.zip › table S1.The photochemical insecticidal activity of target compound I against the fourth instar larva of Culex pipienspallens.pdf]

Table S1 The photochemical insecticidal activity of target compound I against the fourth instar larva of *Culex pipienspallens*

No.	Corrected mortality （%）	No.	Corrected mortality （%）
I1	31.67±0.56 j	I21	—
I2	—	I22	75.00 ±0.80 e
I3	—	I23	—
I4	48.33±1.01 h	I24	—
I5	80.00 ±0.31 d	I25	76.67±1.74 e
I6	—	I26	—
I7	—	I27	—
I8	21.67±1.19 l	I28	28.33±0.52 k
I9	63.33±0.66 f	I29	76.67±1.53 e
I10	—	I30	75.00 ±1.20 e
I11	—	I31	86.67±1.76 c
I12	43.33±0.63 i	I32	80.00 ±1.01 d
I13	51.67±0.32 g	I33	—
I14	18.33±0.25 m	I34	98.33±0.73 a
I15	61.67±0.64 f	I35	—
I16	—	I36	—
I17	—	I37	—
I18	91.67±0.43 b	I38	—
I19	85.00 ±1.24 c	I39	48.33±0.58 h
I20	—	α-T	100±0.00 a

Note: "—" means that the activity is less than 20%, or no insecticidal activity.
