# Supplementary material for: Potential Biochemical Pesticide—Synthesis of Neofuranocoumarin and Inhibition the Proliferation of Spodoptera frugiperda Cells through Activating the Mitochondrial Pathway
Source: Toxins (Basel). 2022 Sep 29;14(10):677. doi: 10.3390/toxins14100677 (PMC9612269; doi:10.3390/toxins14100677)
Supplement: Supplementary file 1 [file toxins-14-00677-s001.zip › Text S1. the data of compounds I.pdf]

8-methyl-2-phenyl-6*H*-furo[2,3-*g*]chromen-6-one(I1)

White crystal, Yield: 96%,m.p. 241-242 °C.^{1}H NMR (400 MHz, $CDCl_3$) δ 7.89 (d, *J* = 7.3 Hz, 2H), 7.72 (s, 1H), 7.48 (dt, *J* = 19.8, 4.6 Hz, 4H), 7.09 (s, 1H), 6.30 (s, 1H), 2.51 (s, 3H). ^{13}C NMR (100 MHz, $CDCl_3$) δ 161.25, 159.77, 152.47, 151.48, 150.09, 132.75, 129.65, 129.56, 129.01, 125.39, 117.14, 114.03, 107.78, 105.81, 101.32, 19.06. MS (EI): m/z (%) 276 ($[M]^+$, 100), 250 (12), 248 (66), 220 (9), 189 (7), 165 (5), 124 (14), 94 (6). Anal. Calcd for $C_{18}H_{12}O_3$: C, 78.25; H, 4.38. Found: C, 78.28; H, 4.37.

7-chloro-8-methyl-2-phenyl-6*H*-furo[2,3-*g*]chromen-6-one(I2)

White crystal, Yield: 90%, m.p. 256-258°C.^{1}H NMR (400 MHz, $CDCl_3$) δ 7.88 (d, *J* = 7.6 Hz, 2H), 7.73 (s, 1H), 7.59-7.37 (m, 4H), 7.07 (s, 1H), 2.65 (s, 3H). ^{13}C NMR (100 MHz, $CDCl_3$) δ 160.13, 157.25, 151.73, 148.10, 147.79, 132.69, 129.80, 129.41, 129.04, 125.43, 119.79, 116.90, 107.76, 106.08, 101.24, 16.56. MS (EI): m/z (%) 312 ($[M]^+$, 33), 310 (100), 282 (14), 254 (22), 247 (12), 219 (15), 189 (13), 155 (5), 141 (14),124 (14), 96 (16), 95 (19). Anal. Calcd for $C_{18}H_{11}ClO_3$: C, 69.58; H, 3.57. Found: C, 69.55; H, 3.52.

2-(2-fluorophenyl)-8-methyl-6*H*-furo[2,3-*g*]chromen-6-one(I3)

Yellow solid, Yield: 91%, m.p. 248-249 °C.^{1}H NMR (400 MHz, $CDCl_3$) δ 8.14-7.93 (m, 1H), 7.72 (s, 1H), 7.53 (s, 1H), 7.47-7.15 (m, 4H), 6.30 (s, 1H), 2.51 (s, 3H). ^{13}C NMR (100 MHz, $CDCl_3$) δ 161.11, 158.67, 153.71, 152.32, 150.75, 150.07, 132.76, 130.68, 127.26, 124.64, 117.55, 116.43, 116.22, 114.29, 108.18, 106.50, 105.72, 19.02. MS (EI): m/z (%) 294 ($[M]^+$, 100), 268 (18), 266 (95), 238 (11), 209 (10), 183 (7), 147 (6), 133 (35), 109 (7), 94 (9), 91 (7). Anal. Calcd for $C_{18}H_{11}FO_3$: C, 73.47; H, 3.77. Found: C, 73.40; H, 3.79.

2-(3-fluorophenyl)-8-methyl-6*H*-furo[2,3-*g*]chromen-6-one(I4)

White crystal, Yield: 89%, m.p. 256-257°C.^{1}H NMR (400 MHz, $CDCl_3$) δ 7.88 (dd, *J* = 8.8, 5.3 Hz, 2H), 7.71 (s, 1H), 7.50 (s, 1H), 7.19 (t, *J* = 8.6 Hz, 2H), 7.03 (s, 1H), 6.31 (s, 1H), 2.51 (s, 3H). ^{13}C NMR (100 MHz, $CDCl_3$) δ 161.14, 158.84, 152.35, 151.47, 150.19, 146.75, 132.72, 127.41, 127.33, 125.94, 117.20, 116.34, 116.12, 114.14, 107.82, 105.80, 101.06, 19.02. MS (EI): m/z (%) 294 ($[M]^+$, 100), 266

(88), 238 (9), 207 (11), 183 (8), 133 (31), 118 (5), 95 (7), 91 (5). Anal. Calcd for $C_{18}H_{11}FO_3$: C, 73.47; H, 3.77. Found: C, 73.50; H, 3.73.

2-(4-fluorophenyl)-8-methyl-6*H*-furo[2,3-*g*]chromen-6-one(I5)

White crystal, Yield: 85%, m.p. 283-284 °C.^{1}H NMR (400 MHz, $CDCl_3$) δ 7.73 (s, 1H), 7.67 (d, J = 7.8 Hz, 1H), 7.58 (d, J = 9.7 Hz, 1H), 7.52 (s, 1H), 7.46 (d, J = 5.8 Hz, 1H), 7.12 (d, J = 3.4 Hz, 2H), 6.32 (s, 1H), 2.52 (s, 3H). ^{13}C NMR (100 MHz, $CDCl_3$) δ 161.09, 152.33, 151.48, 150.13, 132.38, 130.72, 121.06, 117.58, 116.58, 116.37, 114.32, 112.41, 108.04, 105.97, 102.34, 19.04. MS (EI): m/z (%) 294 ([M]$^+$, 100), 266 (99), 238 (11), 207 (10), 183 (7),133 (28), 109 (7), 92 (7). Anal. Calcd for $C_{18}H_{11}FO_3$: C, 73.47; H, 3.77. Found: C, 73.45; H, 3.74.

7-chloro-2-(2-fluorophenyl)-8-methyl-6*H*-furo[2,3-*g*]chromen-6-one(I6)

Yellow solid, Yield: 86%, m.p. 172-173 °C.^{1}H NMR (400 MHz, $CDCl_3$) δ 8.02 (t, *J* = 7.0 Hz, 1H), 7.74 (s, 1H), 7.54 (s, 1H), 7.47-7.38 (m, 1H), 7.26 (tt, *J* = 11.2, 8.0 Hz, 3H), 2.66 (s, 3H). ^{13}C NMR (100 MHz, $CDCl_3$) δ 161.23, 157.18, 154.04, 151.03, 148.09, 147.69, 133.11, 132.73, 130.87, 127.32, 124.69, 117.82, 117.32, 116.48, 116.26, 108.20, 106.44, 106.02, 16.58. MS (EI): m/z (%) 328 ([M]$^+$, 98), 300 (15), 265 (20), 237 (10), 207 (14), 150 (10), 133 (20), 123 (100), 103 (7), 95 (20), 94 (9). Anal. Calcd for $C_{18}H_{10}ClFO_3$: C, 65.77; H, 3.07. Found: C, 65.72; H, 3.11.

7-chloro-2-(3-fluorophenyl)-8-methyl-6*H*-furo[2,3-*g*]chromen-6-one(I7)

Yellow solid, Yield: 89%, m.p. 250-252 °C.^{1}H NMR (400 MHz, $CDCl_3$) δ 7.76 (s, 1H), 7.67 (d, *J* = 7.8 Hz, 1H), 7.58 (d, *J* = 9.5 Hz, 1H), 7.54 (s, 1H), 7.46 (d, *J* = 5.8 Hz, 1H), 7.22-7.02 (m, 2H), 2.67 (s, 3H). ^{13}C NMR (100 MHz, $CDCl_3$) δ 158.69, 157.15, 151.76, 148.16, 147.68, 132.33, 130.78, 121.16, 120.14, 117.36, 116.75, 116.54, 112.48, 112.25, 108.06, 106.27, 102.29, 16.59. MS (EI): m/z (%) 328 ([M]$^+$, 100), 300 (19), 272 (15), 265 (22), 237 (12), 207 (16), 183 (7), 150 (10), 133 (21), 118 (6), 94 (9). Anal. Calcd for $C_{18}H_{10}ClFO_3$: C, 65.77; H, 3.07. Found: C, 65.73; H, 3.04.

7-chloro-2-(4-fluorophenyl)-8-methyl-6*H*-furo[2,3-*g*]chromen-6-one(I8)

White crystal, Yield: 87%, m.p.128-130°C. ^{1}H NMR (400 MHz, $CDCl_3$) δ 7.88 (dd, *J* = 8.9, 5.3 Hz, 2H), 7.74 (s, 1H), 7.52 (s, 1H), 7.19 (t, *J* = 8.7 Hz, 2H), 7.03 (s,

1H), 2.67 (s, 3H). ^{13}C NMR (100 MHz, $CDCl_3$) δ 159.17, 157.26, 151.71, 148.14, 147.78, 132.66, 127.46, 127.38, 119.85, 116.94, 116.38, 116.16, 107.81, 106.09, 100.99, 16.60. MS (EI): m/z (%) 328 ($[M]^+$, 100), 300(16), 265(20), 237(10), 207(12), 150(9), 133(20), 103(7). Anal. Calcd for $C_{18}H_{10}ClFO_3$: C, 65.77; H, 3.07. Found: C, 65.75; H, 3.02.

2-(2-chlorophenyl)-8-methyl-6*H*-furo[2,3-*g*]chromen-6-one(I9)

White crystal, Yield: 97%, m.p. 269-270 °C. ^{1}H NMR (400 MHz, $CDCl_3$) δ 8.04 (d, J = 7.8 Hz, 1H), 7.71 (s, 1H), 7.54 (t, J = 11.7 Hz, 3H), 7.38 (dd, J = 21.1, 7.6 Hz, 2H), 6.31 (s, 1H), 2.51 (s, 3H). ^{13}C NMR (100 MHz, $CDCl_3$) δ 161.06, 155.75, 152.28, 150.70, 150.02, 132.45, 131.89, 131.14, 130.05, 129.21, 128.17, 127.14, 117.73, 114.38, 108.32, 107.22, 105.75, 19.01. MS (EI): m/z (%) 310 ($[M]^+$, 100), 284 (26), 282 (79), 254 (8), 219 (7), 189 (19), 141 (19), 109 (14), 95 (35). Anal. Calcd for $C_{18}H_{11}ClO_3$: C, 69.58; H, 3.57. Found: C, 69.57; H, 3.55.

2-(3-chlorophenyl)-8-methyl-6*H*-furo[2,3-*g*]chromen-6-one(I10)

White crystal, Yield: 87%, m.p. 248-250°C. ^{1}H NMR (400 MHz, $CDCl_3$) δ 7.88 (s, 1H), 7.82-7.69 (m, 2H), 7.51 (s, 1H), 7.47-7.35 (m, 2H), 7.11 (s, 1H), 6.31 (s, 1H), 2.51 (s, 3H). ^{13}C NMR (100 MHz, $CDCl_3$) δ 161.05, 158.09, 152.29, 151.51, 150.15, 135.16, 132.34, 131.28, 130.29, 129.52, 125.38, 123.42, 117.61, 114.35, 108.05, 105.96, 102.36, 19.02. MS (EI): m/z (%) 310 ($[M]^+$, 100), 284 (32), 282 (82), 254 (11), 219 (6), 189 (12), 140 (13), 109 (17), 94 (29). Anal. Calcd for $C_{18}H_{11}ClO_3$: C, 69.58; H, 15.45. Found: C, 69.55; H, 15.47.

2-(4-chlorophenyl)-8-methyl-6*H*-furo[2,3-*g*]chromen-6-one(I11)

White crystal, Yield: 93%, m.p. 275-277 °C. ^{1}H NMR (400 MHz, $CDCl_3$) δ 7.82 (d, J = 8.5 Hz, 2H), 7.72 (s, 1H), 7.59-7.40 (m, 3H), 7.08 (s, 1H), 6.31 (s, 1H), 2.52 (s, 3H). ^{13}C NMR (100 MHz, $CDCl_3$) δ 161.12, 158.58, 152.34, 151.49, 150.16, 135.58, 132.54, 129.32, 128.07, 126.59, 117.41, 114.24, 107.92, 105.88, 101.75, 19.04. MS (EI): m/z (%) 312 ($[M]^+$, 35), 310 (100), 284 (26), 282 (78), 254 (10), 189 (15), 155 (6), 141 (20), 109 (12), 94 (15). Anal. Calcd for $C_{18}H_{11}ClO_3$: C, 69.58; H, 3.57. Found: C, 69.61; H, 3.53.

7-chloro-2-(2-chlorophenyl)-8-methyl-6*H*-furo[2,3-*g*]chromen-6-one(I12)

White crystal, Yield: 92%, m.p. 132-133 °C. ^{1}H NMR (400 MHz, $CDCl_3$) δ 8.06 (dd, *J* = 7.8, 1.7 Hz, 1H), 7.77 (s, 1H), 7.65-7.51 (m, 3H), 7.39 (dd, *J* = 26.9, 1.5 Hz, 2H), 2.68 (s, 3H). ^{13}C NMR (100 MHz, $CDCl_3$) δ 157.18, 147.68, 132.44, 132.39, 132.00, 131.22, 131.16, 130.23, 129.27, 129.22, 127.20, 127.15, 117.54, 108.39, 108.34, 107.19, 106.08, 16.59. MS (EI): m/z (%) 346([M]$^+$, 63), 344 (100), 316 (18), 288 (13), 281 (22), 253 (11), 189 (23), 158 (19), 141 (15), 126 (10), 95 (27), 94 (15). Anal. Calcdfor $C_{18}H_{10}Cl_2O_3$: C, 62.63; H, 2.92. Found: C, 62.67; H, 2.87.

7-chloro-2-(3-chlorophenyl)-8-methyl-6*H*-furo[2,3-*g*]chromen-6-one(I13)

White crystal, Yield: 96%, m.p. 227-229 °C. ^{1}H NMR (400 MHz, $CDCl_3$) δ 7.74-7.61 (m, 3H), 7.54 (dd, *J* = 7.4, 1.6 Hz, 1H), 7.47 (dt, *J* = 5.5, 3.5 Hz, 3H), 2.75 (s, 3H). ^{13}C NMR (100 MHz, $CDCl_3$) δ 160.15, 158.20, 153.09, 150.81, 150.04, 136.58, 133.19, 132.09, 130.78, 129.02, 1236.34, 122.82, 120.55, 115.46, 109.57, 106.03, 103.15, 16.61. MS (EI): m/z (%) 345 ([M]$^+$, 1), 312 (38), 310 (100), 282 (20), 247 (15),219 (13), 189 (17), 141 (23), 124 (23), 109 (6), 95 (23). Anal. Calcd for $C_{18}H_{10}Cl_2O_3$: C, 62.63; H, 2.92. Found: C, 62.59; H, 2.93.

7-chloro-2-(4-chlorophenyl)-8-methyl-6*H*-furo[2,3-*g*]chromen-6-one(I14)

Yellow solid, Yield: 82%, m.p. 288-290°C. ^{1}H NMR (400 MHz, $CDCl_3$) δ 7.82 (d, *J* = 8.6 Hz, 2H), 7.74 (s, 1H), 7.54-7.44 (m, 3H), 7.08 (s, 1H), 2.67 (s, 3H). ^{13}C NMR (100 MHz, $CDCl_3$) δ 158.92, 157.20, 151.73, 148.14, 147.73, 135.76, 132.48, 129.36, 127.90, 126.63, 119.99, 117.16, 107.91, 106.17, 101.68, 16.59 MS (EI): m/z (%) 346([M]$^+$, 73), 344 (100), 316 (16), 288 (10), 281 (19), 253 (12), 189 (24), 172 (8), 158 (15), 141 (25), 126 (12), 95 (15). Anal. Calcd for $C_{18}H_{10}Cl_2O_3$: C, 62.63; H, 2.92. Found: C, 62.64; H, 2.96.

2-(4-bromophenyl)-8-methyl-6*H*-furo[2,3-*g*]chromen-6-one(I15)

Yellow solid, Yield: 89%, m.p. 277-279°C. ^{1}H NMR (400 MHz, $CDCl_3$) δ 7.79-7.66 (m, 3H), 7.62 (d, *J* = 8.4 Hz, 2H), 7.51 (s, 1H), 7.09 (s, 1H), 6.31 (s, 1H), 2.51 (s, 3H). ^{13}C NMR (100 MHz, $CDCl_3$) δ 161.22, 159.71, 152.46, 151.55, 150.08, 133.99, 132.51, 130.46, 128.83, 127.73, 127.26, 126.38, 125.27, 117.24, 114.18, 107.90, 105.89, 19.05. MS (EI): m/z (%) 355([M]$^+$, 27), 353 (13), 328 (29), 310 (10), 294 (33), 282 (7), 266 (19), 239 (9), 207 (25), 185 (68), 183 (100), 157 (13), 133

(15), 115 (12), 96 (16), 95 (22). Anal. Calcd for $C_{18}H_{11}BrO_3$: C, 60.87; H, 3.12. Found: C, 60.83; H, 3.15.

2-(4-bromophenyl)-7-chloro-8-methyl-6*H*-furo[2,3-*g*]chromen-6-one(I16)

White crystal, Yield: 91%, m.p.>300 °C. ^{1}H NMR (400 MHz, $CDCl_3$) δ 7.85-7.71 (m, 3H), 7.64 (s, 2H), 7.53 (s, 1H), 7.10 (s, 1H), 2.67 (s, 3H). ^{13}C NMR (100 MHz, $CDCl_3$) δ 160.41, 159.45, 159.44, 157.16, 148.18, 147.67, 132.31, 132.29, 129.75, 126.84, 126.83, 126.81, 107.94, 106.18, 101.79, 16.58. MS (EI): m/z (%) 390 ($[M]^+$, 100), 388 (72), 362 (13), 325 (11), 218 (5), 189 (30), 164 (12), 126 (7), 95 (26), 94 (12). Anal. Calcd for $C_{18}H_{10}BrClO_3$: C, 55.49; H, 2.59. Found: C, 55.46; H, 2.55.

2-(3,4-difluorophenyl)-8-methyl-6H-furo[2,3-*g*]chromen-6-one(I17)

Yellow solid, Yield: 85%, m.p.187-189°C. ^{1}H NMR (400 MHz, $CDCl_3$) δ 7.78-7.58 (m, 2H), 7.51 (s, 1H), 7.37-7.20 (m, 2H), 7.05 (s, 1H), 6.31 (s, 1H), 2.51 (s, 3H). ^{13}C NMR (100 MHz, $CDCl_3$) δ 161.00, 157.44, 154.27, 152.24, 151.49, 150.20, 132.36, 126.71, 121.72, 118.26, 118.08, 117.60, 114.63, 114.40, 108.05, 105.95, 102.02, 19.02. MS (EI): m/z (%) 312 ($[M]^+$, 100), 284 (98), 256 (9), 227 (13), 201 (8), 142 (29), 118 (8), 111 (7). Anal. Calcdfor $C_{18}H_{10}F_2O_3$: C, 69.23; H, 3.23. Found: C, 69.20; H, 3.21.

2-(2,6-difluorophenyl)-8-methyl-6*H*-furo[2,3-*g*]chromen-6-one(I18)

White crystal, Yield: 82%, m.p. 222-224°C. ^{1}H NMR (400 MHz, $CDCl_3$) δ 7.78 (s, 1H), 7.56 (s, 1H), 7.48-7.32 (m, 2H), 7.07 (t, J = 8.7 Hz, 2H), 6.32 (s, 1H), 2.51 (s, 3H). ^{13}C NMR (100 MHz, $CDCl_3$) δ 161.07, 158.91, 158.85, 152.35, 151.25, 149.99, 131.55, 130.81, 117.83, 114.48, 112.49, 112.23, 108.73, 108.11, 106.18, 19.02. MS (EI): m/z (%) 312 ($[M]^+$, 100), 284 (94), 256 (11), 227 (9), 201 (8), 142 (23), 103 (9). Anal. Calcd for $C_{18}H_{10}F_2O_3$: C, 69.23; H, 3.23. Found: C, 69.25; H, 3.22.

2-(2,3-dichlorophenyl)-8-methyl-6*H*-furo[2,3-*g*]chromen-6-one(I19)

White crystal, Yield: 90%, m.p. 259-260°C. ^{1}H NMR (400 MHz, $CDCl_3$) δ 7.95 (dd, J = 8.0, 1.4 Hz, 1H), 7.72 (s, 1H), 7.65-7.49 (m, 3H), 7.36 (t, J = 8.0 Hz, 1H), 6.32 (s, 1H), 2.51 (s, 3H). ^{13}C NMR (100 MHz, $CDCl_3$) δ 161.00, 155.13, 152.23, 150.75, 150.02, 134.78, 132.15, 130.88, 130.37, 130.19, 127.60, 127.53, 118.04, 114.59, 108.51, 108.05, 105.87, 19.04. MS (EI): m/z (%) 346 ($[M]^+$, 68), 344

(100), 318 (62), 316 (91), 288 (9), 253 (6), 189 (27), 172 (9), 158 (25), 123 (21), 108 (7), 94 (24), 93 (11). Anal. Calcd for $C_{18}H_{10}Cl_2O_3$: C, 62.63; H, 2.92. Found: C, 62.64; H, 2.89.

7-chloro-2-(2,3-dichlorophenyl)-8-methyl-6*H*-furo[2,3-*g*]chromen-6-one(I20)

White crystal, Yield: 93%, m.p. 287-289 °C. ^{1}H NMR (400 MHz, $CDCl_3$) δ 7.95 (s, 1H), 7.77 (s, 1H), 7.61 (d, *J* = 4.9 Hz, 2H), 7.55 (d, *J* = 1.3 Hz, 1H), 7.38 (d, *J* = 8.0 Hz, 1H), 2.68 (s, 3H). ^{13}C NMR (100 MHz, $CDCl_3$) δ 155.50, 151.03, 148.04, 147.61, 134.85, 132.11, 131.04, 130.29, 130.27, 127.66, 127.57, 126.37, 120.41, 117.82, 108.57, 108.00, 106.19, 16.62. MS (EI): m/z (%) 380 ([M]$^+$, 100), 378 (95), 352 (19), 350 (21), 328 (11), 317 (17), 315 (23), 287 (11), 223 (9), 189 (24), 158 (12), 123 (7), 111 (10), 94 (24), 93 (12). Anal. Calcd for $C_{18}H_9Cl_3O_3$: C, 56.95; H, 2.39. Found: C, 56.99; H, 2.36.

2-(2,4-dichlorophenyl)-8-methyl-6*H*-furo[2,3-*g*]chromen-6-one(I21)

White crystal, Yield: 86%, m.p. 264-266 °C. ^{1}H NMR (400 MHz, $CDCl_3$) δ 8.00 (d, *J* = 8.6 Hz, 1H), 7.72 (s, 1H), 7.57 (dd, *J* = 7.8, 6.0 Hz, 3H), 7.41 (dd, *J* = 8.6, 2.1 Hz, 1H), 6.33 (s, 1H), 2.52 (s, 3H). ^{13}C NMR (100 MHz, $CDCl_3$) δ 160.99, 154.73, 152.20, 150.68, 150.09, 135.42, 132.44, 132.29, 130.95, 129.89, 127.62, 126.79, 117.99, 114.58, 108.46, 107.49, 105.81, 19.02. MS (EI): m/z (%) 346 ([M]$^+$, 57), 344 (100), 318 (56), 316 (69), 207 (11), 189 (24), 173 (9), 158 (21), 123 (13),108 (9), 95 (18), 94 (24). Anal. Calcd for $C_{18}H_{10}Cl_2O_3$: C, 62.63; H, 2.92. Found: C, 62.65; H, 2.88.

7-chloro-2-(2,4-dichlorophenyl)-8-methyl-6*H*-furo[2,3-*g*]chromen-6-one(I22)

Yellow solid, Yield: 96%, m.p. 126-127 °C. ^{1}H NMR (400 MHz, $CDCl_3$) δ 8.02-7.73 (m, 2H), 7.56-7.43 (m, 3H), 7.39-7.28 (m, 1H), 2.66 (s, 3H). ^{13}C NMR (100 MHz, $CDCl_3$) δ 161.06, 155.49, 154.32, 151.79, 150.35, 140.19, 136.57, 134.18, 130.34, 129.06, 128.24, 118.09, 117.44, 115.22, 108.16, 106.51, 105.46, 16.56. MS (EI): m/z (%) 380 ([M]$^+$, 100), 378 (95), 350 (17), 322 (6), 315 (23), 287 (10), 223 (9), 189 (22), 177 (23),175 (33), 158 (15), 140 (8), 108 (7), 93 (13). Anal. Calcd for $C_{18}H_9Cl_3O_3$: C, 56.95; H, 2.39. Found: C, 56.90; H, 2.41.

7-chloro-2-(3,5-dichlorophenyl)-8-methyl-6*H*-furo[2,3-*g*]chromen-6-one(I23)

White crystal, Yield: 89%, m.p. 259-271 °C. ^1H NMR (400 MHz, $CDCl_3$) δ 7.82-7.72 (m, 3H), 7.56 (s, 1H), 7.41 (s, 1H), 7.15 (s, 1H), 2.68 (s, 3H). ^{13}C NMR (100 MHz, $CDCl_3$) δ 157.02, 154.63, 151.84, 148.21, 147.53, 135.91, 132.19, 131.91, 129.41, 123.69, 122.67, 117.83, 108.35, 106.42, 103.31, 16.59. MS (EI): m/z (%) 380 ($[M]^+$, 100), 378 (95), 350 (16), 322 (12), 315 (20), 287 (10), 223 (10), 189 (24), 176 (26), 158 (17), 143 (6), 126 (9), 95 (17), 93 (14). Anal. Calcd for $C_{18}H_9Cl_3O_3$: C, 56.95; H, 2.39. Found: C, 56.90; H, 2.40.

8-methyl-2-(3-(trifluoromethyl)phenyl)-6*H*-furo[2,3-*g*]chromen-6-one(I24)

Yellow solid, Yield: 91%, m.p. 282-283 °C. ^1H NMR (400 MHz, $CDCl_3$) δ 8.15 (s, 1H), 8.05 (d, J = 7.7 Hz, 1H), 7.76 (s, 1H), 7.65 (dd, J = 16.9, 7.8 Hz, 2H), 7.54 (s, 1H), 7.19 (s, 1H), 6.33 (s, 1H), 2.52 (s, 3H). ^{13}C NMR (100 MHz, $CDCl_3$) δ 161.01, 157.90, 152.27, 151.56, 150.18, 132.25, 131.84, 130.39, 129.59, 128.36, 125.99, 122.49, 122.15, 117.78, 114.47, 108.17, 106.07, 102.64, 19.03. MS (EI): m/z (%) 344($[M]^+$, 100), 316 (89), 314 (21), 288 (10), 189 (9), 158 (32), 156 (8), 133 (8). Anal. Calcd for $C_{19}H_{11}F_3O_3$: C, 66.28; H, 3.22. Found: C, 66.30; H, 3.19.

7-chloro-8-methyl-2-(3-(trifluoromethyl)phenyl)-6*H*-furo[2,3-*g*]chromen-6-one(I25)

White crystal, Yield: 90%, m.p.>300 °C. ^1H NMR (400 MHz, $CDCl_3$) δ 8.15 (s, 1H), 8.06 (d, J = 7.7 Hz, 1H), 7.79 (s, 1H), 7.66 (dd, J = 18.4, 7.6 Hz, 2H), 7.56 (s, 1H), 7.20 (s, 1H), 2.68 (s, 3H). ^{13}C NMR (100 MHz, $CDCl_3$) δ 158.26, 157.09, 151.84, 148.20, 147.62, 132.19, 131.59, 130.27, 129.64, 128.43, 126.14, 125.18, 122.19, 120.30, 117.56, 108.19, 106.37, 102.59, 16.59. MS (EI): m/z (%) 278 ($[M]^+$, 100), 350 (21), 322 (11), 315 (19), 287 (11), 189 (17), 175 (30), 158 (10), 148 (7), 95 (6). Anal. Calcd for $C_{19}H_{10}ClF_3O_3$: C, 60.26; H, 2.66. Found: C, 60.22; H, 2.63.

8-methyl-2-(4-(trifluoromethyl)phenyl)-6*H*-furo[2,3-*g*]chromen-6-one(I26)

White crystal, Yield: 90%, m.p. 271-273 °C. ^1H NMR (400 MHz, $CDCl_3$) δ 7.99 (d, J = 8.2 Hz, 2H), 7.75 (t, J = 4.1 Hz, 3H), 7.54 (s, 1H), 7.20 (s, 1H), 6.33 (s, 1H), 2.52 (s, 3H). ^{13}C NMR (100 MHz, $CDCl_3$) δ 160.95, 157.90, 152.20, 151.68, 150.20, 132.19, 126.06, 126.02, 125.52, 117.91, 114.57, 108.24, 106.12, 103.21, 19.02. MS (EI): m/z (%) 346 ($[M]^+$, 17), 344 (100), 316 (89), 314 (33), 288 (10), 189 (12), 172

(7), 158 (23), 133 (12). Anal. Calcd for $C_{19}H_{11}F_3O_3$: C, 66.28; H, 3.22. Found: C, 66.32; H, 3.20.

2-(4-bromo-3-fluorophenyl)-8-methyl-6*H*-furo[2,3-*g*]chromen-6-one(I27)

White crystal, Yield: 84%, m.p. 249-251 °C. ^{1}H NMR (400 MHz, $CDCl_3$) δ 7.72 (s, 1H), 7.70-7.60 (m, 2H), 7.54 (d, J = 11.4 Hz, 2H), 7.13 (s, 1H), 6.32 (s, 1H), 2.52 (s, 3H). ^{13}C NMR (100 MHz, $CDCl_3$) δ 160.94, 158.30, 152.18, 151.53, 150.23, 134.27, 132.21, 130.82, 122.00, 121.97, 117.86, 114.54, 113.29, 113.05, 108.16, 106.04, 102.78, 19.01. MS (EI): m/z (%) 374 ($[M]^+$, 100), 372 (100), 346 (63), 344 (65), 318 (9), 207 (24), 187 (7), 173 (27), 171 (23),144 (6), 133 (13), 118 (21), 104 (16), 92 (13). Anal. Calcd for $C_{18}H_{10}BrFO_3$: C, 57.93; H, 2.70. Found: C, C, 57.90; H, 2.72.

2-(2-bromo-4-fluorophenyl)-8-methyl-6*H*-furo[2,3-*g*]chromen-6-one (I28)

White crystal, Yield: 87%, m.p. 261-263°C. ^{1}H NMR (400 MHz, $CDCl_3$) δ 7.96 (dd, J = 8.8, 6.0 Hz, 1H), 7.72 (s, 1H), 7.56 (s, 1H), 7.54 (s, 1H), 7.50 (dd, J = 8.2, 2.6 Hz, 1H), 7.23-7.15 (m, 1H), 6.32 (s, 1H), 2.51 (s, 3H). ^{13}C NMR (100 MHz, $CDCl_3$) δ 161.05, 156.19, 152.27, 150.85, 150.08, 132.16, 131.40, 126.80, 121.92, 121.67, 117.76, 115.29, 115.08, 114.47, 108.37, 106.64, 105.84, 19.02. MS (EI): m/z (%) 376 ($[M]^+$, 23), 374 (100), 372 (97), 346 (68), 344 (67), 237 (13), 207 (34), 187 (8), 173 (25),132 (9), 118 (28),104 (20),92 (14). Anal. Calcd for $C_{18}H_{10}BrFO_3$: C, 57.93; H, 2.70. Found: C, 57.95; H, 2.71.

8-methyl-2-(2,3,4,5-tetrafluorophenyl)-6*H*-furo[2,3-*g*]chromen-6-one(I29)

White crystal, Yield: 79%, m.p. 157-158 °C. ^{1}H NMR (400 MHz, $CDCl_3$) δ 7.74 (s, 1H), 7.72-7.62 (m, 2H), 7.51-7.42 (m, 1H), 6.35 (s, 1H), 2.52 (s, 3H). ^{13}C NMR (100 MHz, $CDCl_3$) δ 160.79, 152.03, 150.77, 150.23, 133.15, 132.18, 132.11, 132.08, 131.95, 131.92, 128.58, 128.45, 118.40, 114.91, 108.69, 107.77, 106.03, 19.01. MS (EI): m/z (%) 348($[M]^+$, 93), 320 (100), 292 (9), 263 (10), 243 (8), 174 (7), 160 (18), 136 (7). Anal. Calcd for $C_{18}H_8F_4O_3$: C, 62.08; H, 2.32. Found: C, 62.04; H, 2.33.

7-chloro-8-methyl-2-(2,3,4,5-tetrafluorophenyl)-6*H*-furo[2,3-*g*]chromen-6-one(I30)

White crystal, Yield: 76%, m.p.>300°C. ^{1}H NMR (400 MHz, $CDCl_3$) δ 7.79 (s,

1H), 7.68 (s, 1H), 7.60 (s, 1H), 7.35 (s, 1H), 2.68 (s, 3H). ^{13}C NMR (100 MHz, $CDCl_3$) δ 158.27, 154.84, 151.05, 148.23, 146.88, 131.99, 131.50, 130.40, 123.44, 118.17, 110.19, 108.74, 108.21, 107.71, 107.60, 106.32, 95.98, 16.60. MS (EI): m/z (%) 382 ([M]$^+$, 100), 354(19), 326 (11), 319 (34), 303 (7), 291 (11), 261 (8), 243 (11), 237 (6), 177 (26), 160 (20), 118 (5). Anal. Calcd for $C_{18}H_7ClF_4O_3$: C, 56.49; H, 1.84. Found: C, 56.52; H, 1.88.

2-(3-fluoro-5-(trifluoromethoxy)phenyl)-8-methyl-6*H*-furo[2,3-*g*]chromen-6-one(I31)

White crystal, Yield: 69%, m.p. 244-246 °C. ^{1}H NMR (400 MHz, $CDCl_3$) δ 7.96 (s, 1H), 7.75 (s, 1H), 7.67 (s, 1H), 7.54 (s, 1H), 7.42 (s, 1H), 7.17 (s, 1H), 6.34 (s, 1H), 2.52 (s, 3H). ^{13}C NMR (100 MHz, $CDCl_3$) δ 160.83, 156.24, 152.10, 151.59, 150.23, 132.82, 131.88, 126.63, 124.78, 123.51, 121.66, 119.09, 118.19, 116.38, 114.75, 108.37, 106.19, 103.63, 19.00. MS (EI): m/z (%) 440 ([M]$^+$, 100), 438 (88), 412 (90), 410 (93), 382 (10), 303 (8), 275 (7), 219 (10), 207 (29), 205 (30), 176 (12), 167 (6), 94 (9). Anal. Calcd for $C_{19}H_{10}F_4O_4$: C, 60.33; H, 2.66. Found: C, 60.29; H, 2.68.

2-(4-methoxyphenyl)-8-methyl-6*H*-furo[2,3-*g*]chromen-6-one(I32)

Yellow solid, Yield: 95%, m.p. 152-153 °C. ^{1}H NMR (400 MHz, $CDCl_3$) δ 7.82 (d, J = 8.8 Hz, 2H), 7.67 (s, 1H), 7.45 (s, 1H), 7.01 (d, J = 8.8 Hz, 2H), 6.94 (s, 1H), 6.27 (s, 1H), 3.88 (s, 3H), 2.50 (s, 3H). ^{13}C NMR (100 MHz, $CDCl_3$) δ 161.31, 160.88, 160.01, 152.50, 151.34, 150.16, 133.16, 126.98, 122.35, 116.64, 114.50, 113.72, 107.39, 105.51, 99.68, 55.45, 19.02. MS (EI): m/z (%) 308 ([M]$^+$, 21), 306 (100), 278 (27), 263 (39), 235 (10), 178 (6), 153 (8), 139 (21), 117 (7). Anal. Calcd for $C_{19}H_{14}O_4$: C, 74.50; H, 4.61. Found: C, 74.52; H, 4.58.

2-(furan-2-yl)-8-methyl-6H-furo[2,3-g]chromen-6-one(I33)

White crystal, Yield: 83%, m.p. 249-250 °C. ^{1}H NMR (400 MHz, $CDCl_3$) δ 7.68 (s, 1H), 7.57 (s, 1H), 7.49 (s, 1H), 6.96 (s, 1H), 6.90 (d, J = 3.4 Hz, 1H), 6.57 (dd, J = 3.4, 1.8 Hz, 1H), 6.30 (s, 1H), 2.50 (s, 3H). ^{13}C NMR (100 MHz, $CDCl_3$) δ 161.15, 152.32, 151.56, 151.17, 150.26, 145.42, 144.09, 132.33, 117.20, 114.07, 112.02, 109.48, 107.89, 105.70, 100.94, 19.01. MS (EI): m/z (%) 266 ([M]$^+$, 100), 238 (75), 209 (2), 181 (2), 153 (2), 133 (1), 119 (5), 104 (3), 90 (5), 77 (6). Anal. Calcd for

$C_{16}H_{10}O_4$: C, 72.18; H, 3.79. Found: C, 72.16; H, 3.82.

8-methyl-2-(thiophen-2-yl)-6*H*-furo[2,3-*g*]chromen-6-one(I34)

White crystal, Yield: 76%, m.p.140-141°C. ^{1}H NMR (400 MHz, $CDCl_3$) δ 7.68 (s, 1H), 7.56 (d, *J* = 3.7 Hz, 1H), 7.50-7.38 (m, 2H), 7.20-7.09 (m, 1H), 6.92 (s, 1H), 6.29 (s, 1H), 2.49 (s, 3H). ^{13}C NMR (100 MHz, $CDCl_3$) δ 161.15, 155.06, 152.37, 151.15, 150.26, 132.71, 132.33, 128.21, 127.34, 126.03, 117.12, 114.01, 107.60, 105.67, 100.94, 19.01. MS (EI): m/z (%) 284 ($[M]^+$, 19), 282 (100), 254 (65), 252 (12), 226 (11), 197 (6), 127 (13). Anal. Calcd for $C_{16}H_{10}O_3S$: C, 68.07; H, 3.57; S, 11.36. Found: C, 68.05; H, 3.60; S, 11.34.

7-chloro-8-methyl-2-(thiophen-2-yl)-6*H*-furo[2,3-*g*]chromen-6-one(I35)

Yellow solid, Yield: 92%, m.p.222-224°C. ^{1}H NMR (400 MHz, $CDCl_3$) δ 7.72 (s, 1H), 7.58 (d, *J* = 3.6 Hz, 1H), 7.51-7.38 (m, 2H), 7.16 (s, 1H), 6.93 (s, 1H), 2.66 (s, 3H). ^{13}C NMR (100 MHz, $CDCl_3$) δ 157.23, 155.41, 151.43, 148.27, 147.76, 132.69, 132.17, 128.26, 127.56, 126.23, 119.78, 116.89, 107.60, 105.96, 100.87, 16.57. MS (EI): m/z (%) 318 ($[M]^+$, 44), 316 (100), 288 (18), 253 (15), 225 (14), 195 (6), 144 (11), 126 (16). Anal. Calcd for $C_{16}H_9ClO_3S$: C, 60.67; H, 2.86; S, 10.12. Found: C, 60.64; H, 2.85; S, 10.09.

2-(6-chloropyridin-3-yl)-8-methyl-6*H*-furo[2,3-*g*]chromen-6-one(I36)

White crystal, Yield: 82%, m.p. 155-157 °C. ^{1}H NMR (400 MHz, $CDCl_3$) δ 8.92 (s, 1H), 8.22-8.04 (m, 1H), 7.75 (s, 1H), 7.54 (s, 1H), 7.46 (d, *J* = 8.4 Hz, 1H), 7.19 (s, 1H), 6.33 (s, 1H), 2.52 (s, 3H). ^{13}C NMR (100 MHz, $CDCl_3$) δ 160.86, 155.55, 152.13, 152.03, 151.65, 150.27, 146.54, 134.91, 131.92, 124.80, 124.62, 118.04, 114.71, 108.27, 106.16, 103.25, 19.01. MS (EI): m/z (%) 313 ($[M]^+$, 30), 311 (100), 285 (30), 283 (87), 281 (28), 277 (11), 191 (9), 143 (10), 142 (19), 124 (15), 97 (9). Anal. Calcd for $C_{17}H_{10}ClNO_3$: C, 65.50; H, 3.23; N, 4.49. Found: C, 65.54; H, 3.19; N, 4.50.

8-methyl-2-(naphthalen-1-yl)-6*H*-furo[2,3-*g*]chromen-6-one(I37)

White crystal, Yield: 87%, m.p. 209-210 °C. ^{1}H NMR (400 MHz, $CDCl_3$) δ 8.46 (d, *J* = 8.0 Hz, 1H), 8.04-7.84 (m, 3H), 7.79 (s, 1H), 7.69-7.49 (m, 4H), 7.16 (s, 1H), 6.32 (s, 1H), 2.53 (s, 3H). ^{13}C NMR (100 MHz, $CDCl_3$) δ 161.20, 159.71, 152.43,

151.55, 150.09, 134.00, 132.50, 130.58, 130.46, 128.82, 127.73, 127.35, 127.26, 126.37, 125.26, 125.20, 117.24, 114.18, 107.89, 105.94, 105.88, 19.03. MS (EI): m/z (%) 328 ($[M]^+$, 25), 326 (100), 300 (12), 298 (48), 269 (9), 239 (9), 163 (7), 149 (23), 135 (6), 120 (9). Anal. Calcd for $C_{22}H_{14}O_3$: C, 80.97; H, 4.32. Found: C, 80.94; H, 4.29.

7-chloro-8-methyl-2-(naphthalen-1-yl)-6*H*-furo[2,3-*g*]chromen-6-one(I38)

Yellow solid, Yield: 85%, m.p. 223-225 °C. ^{1}H NMR (400 MHz, $CDCl_3$) δ 8.44 (d, J = 8.1 Hz, 1H), 8.04-7.89 (m, 3H), 7.81 (s, 1H), 7.59 (dd, J = 10.6, 4.9 Hz, 4H), 7.16 (s, 1H), 2.67 (s, 3H). ^{13}C NMR (100 MHz, $CDCl_3$) δ 160.07, 157.28, 151.81, 148.08, 147.82, 134.00, 132.46, 130.60, 130.53, 128.87, 127.80, 127.32, 127.17, 126.42, 125.27, 125.13, 119.92, 117.00, 107.90, 106.24, 105.82, 16.59. MS (EI): m/z (%) 362 ($[M]^+$, 38), 360 (100), 332 (14), 304 (7), 297 (10), 269 (10), 239 (18), 226 (6), 180 (9), 166 (19), 143 (18), 134 (7), 120 (18). Anal. Calcd for $C_{22}H_{13}ClO_3$: C, 73.24; H, 3.63. Found: C, 73.21; H, 3.62.

8-methyl-2-(naphthalen-2-yl)-6*H*-furo[2,3-*g*]chromen-6-one(I39)

White crystal, Yield: 88%, m.p.139-140°C. ^{1}H NMR (400 MHz, $CDCl_3$) δ 8.38 (s, 1H), 8.04-7.81 (m, 4H), 7.73 (s, 1H), 7.53 (dd, J = 10.7, 6.8 Hz, 3H), 7.17 (s, 1H), 6.29 (s, 1H), 2.50 (s, 3H). ^{13}C NMR (100 MHz, $CDCl_3$) δ 161.22, 159.78, 152.43, 151.57, 150.11, 133.73, 133.28, 132.79, 128.80, 128.58, 127.88, 127.11, 126.93, 126.75, 124.79, 122.68, 117.21, 114.03, 107.78, 105.75, 101.85, 19.05. MS (EI): m/z (%) 328 ($[M]^+$, 23), 326 (100), 300 (12), 298 (54), 270 (8), 239 (10), 226 (5), 163 (7), 149 (30), 120 (7). Anal. Calcd for $C_{22}H_{14}O_3$: C, 80.97; H, 4.32. Found: C, 81.01; H, 4.33.
